# The role of COVID-19 vaccines in preventing post-COVID-19 thromboembolic and cardiovascular complications

**DOI:** 10.1136/heartjnl-2023-323483

**Published:** 2024-03-12

**Authors:** Núria Mercadé-Besora, Xintong Li, Raivo Kolde, Nhung TH Trinh, Maria T Sanchez-Santos, Wai Yi Man, Elena Roel, Carlen Reyes, Antonella Delmestri, Hedvig M E Nordeng, Anneli Uusküla, Talita Duarte-Salles, Clara Prats, Daniel Prieto-Alhambra, Annika M Jödicke, Martí Català

**Affiliations:** 1 Pharmaco- and Device Epidemiology Group, Health Data Sciences, Botnar Research Centre, NDORMS, University of Oxford, Oxford, UK; 2 Department of Physics, Universitat Politècnica de Catalunya, Barcelona, Spain; 3 Fundació Institut Universitari per a la recerca a l'Atenció Primària de Salut Jordi Gol i Gurina (IDIAPJGol), IDIAP Jordi Gol, Barcelona, Catalunya, Spain; 4 Institute of Computer Science, University of Tartu, Tartu, Estonia; 5 Pharmacoepidemiology and Drug Safety Research Group, Department of Pharmacy, Faculty of Mathematics and Natural Sciences, University of Oslo, Oslo, Norway; 6 School of Pharmacy, University of Oslo, Oslo, Norway; 7 Division of Mental Health, Norwegian Institute of Public Health, Oslo, Norway; 8 Department of Family Medicine and Public Health, University of Tartu, Tartu, Estonia; 9 Department of Medical Informatics, Erasmus University Medical Center, Erasmus University Rotterdam, Rotterdam, Zuid-Holland, Netherlands

**Keywords:** COVID-19, Epidemiology, PUBLIC HEALTH, Electronic Health Records

## Abstract

**Objective:**

To study the association between COVID-19 vaccination and the risk of post-COVID-19 cardiac and thromboembolic complications.

**Methods:**

We conducted a staggered cohort study based on national vaccination campaigns using electronic health records from the UK, Spain and Estonia. Vaccine rollout was grouped into four stages with predefined enrolment periods. Each stage included all individuals eligible for vaccination, with no previous SARS-CoV-2 infection or COVID-19 vaccine at the start date. Vaccination status was used as a time-varying exposure. Outcomes included heart failure (HF), venous thromboembolism (VTE) and arterial thrombosis/thromboembolism (ATE) recorded in four time windows after SARS-CoV-2 infection: 0–30, 31–90, 91–180 and 181–365 days. Propensity score overlap weighting and empirical calibration were used to minimise observed and unobserved confounding, respectively.

Fine-Gray models estimated subdistribution hazard ratios (sHR). Random effect meta-analyses were conducted across staggered cohorts and databases.

**Results:**

The study included 10.17 million vaccinated and 10.39 million unvaccinated people. Vaccination was associated with reduced risks of acute (30-day) and post-acute COVID-19 VTE, ATE and HF: for example, meta-analytic sHR of 0.22 (95% CI 0.17 to 0.29), 0.53 (0.44 to 0.63) and 0.45 (0.38 to 0.53), respectively, for 0–30 days after SARS-CoV-2 infection, while in the 91–180 days sHR were 0.53 (0.40 to 0.70), 0.72 (0.58 to 0.88) and 0.61 (0.51 to 0.73), respectively.

**Conclusions:**

COVID-19 vaccination reduced the risk of post-COVID-19 cardiac and thromboembolic outcomes. These effects were more pronounced for acute COVID-19 outcomes, consistent with known reductions in disease severity following breakthrough versus unvaccinated SARS-CoV-2 infection.

**WHAT IS ALREADY KNOWN ON THIS TOPIC**

- COVID-19 vaccines proved to be highly effective in reducing the severity of acute SARS-CoV-2 infection.
- While COVID-19 vaccines were associated with increased risk for cardiac and thromboembolic events, such as myocarditis and thrombosis, the risk of complications was substantially higher due to SARS-CoV-2 infection.

**WHAT THIS STUDY ADDS**

- COVID-19 vaccination reduced the risk of heart failure, venous thromboembolism and arterial thrombosis/thromboembolism in the acute (30 days) and post-acute (31 to 365 days) phase following SARS-CoV-2 infection. This effect was stronger in the acute phase.
- The overall additive effect of vaccination on the risk of post-vaccine and/or post-COVID thromboembolic and cardiac events needs further research.

**HOW THIS STUDY MIGHT AFFECT RESEARCH, PRACTICE OR POLICY**

- COVID-19 vaccines proved to be highly effective in reducing the risk of post-COVID cardiovascular and thromboembolic complications.

## Introduction

COVID-19 vaccines were approved under emergency authorisation in December 2020 and showed high effectiveness against SARS-CoV-2 infection, COVID-19-related hospitalisation and death.^1 2^ However, concerns were raised after spontaneous reports of unusual thromboembolic events following adenovirus-based COVID-19 vaccines, an association that was further assessed in observational studies.^3 4^ More recently, mRNA-based vaccines were found to be associated with a risk of rare myocarditis events.^5 6^

On the other hand, SARS-CoV-2 infection can trigger cardiac and thromboembolic complications.^7 8^ Previous studies showed that, while slowly decreasing over time, the risk for serious complications remain high for up to a year after infection.^9 10^ Although acute and post-acute cardiac and thromboembolic complications following COVID-19 are rare, they present a substantial burden to the affected patients, and the absolute number of cases globally could become substantial.

Recent studies suggest that COVID-19 vaccination could protect against cardiac and thromboembolic complications attributable to COVID-19.^11 12^ However, most studies did not include long-term complications and were conducted among specific populations.

Evidence is still scarce as to whether the combined effects of COVID-19 vaccines protecting against SARS-CoV-2 infection and reducing post-COVID-19 cardiac and thromboembolic outcomes, outweigh any risks of these complications potentially associated with vaccination.

We therefore used large, representative data sources from three European countries to assess the overall effect of COVID-19 vaccines on the risk of acute and post-acute COVID-19 complications including venous thromboembolism (VTE), arterial thrombosis/thromboembolism (ATE) and other cardiac events. Additionally, we studied the comparative effects of ChAdOx1 versus BNT162b2 on the risk of these same outcomes.

## Methods

### Data sources

We used four routinely collected population-based healthcare datasets from three European countries: the UK, Spain and Estonia.

For the UK, we used data from two primary care databases—namely, Clinical Practice Research Datalink, CPRD Aurum^13^ and CPRD Gold.^14^ CPRD Aurum currently covers 13 million people from predominantly English practices, while CPRD Gold comprises 3.1 million active participants mostly from GP practices in Wales and Scotland. Spanish data were provided by the Information System for the Development of Research in Primary Care (SIDIAP),^15^ which encompasses primary care records from 6 million active patients (around 75% of the population in the region of Catalonia) linked to hospital admissions data (Conjunt Mínim Bàsic de Dades d’Alta Hospitalària). Finally, the CORIVA dataset based on national health claims data from Estonia was used. It contains all COVID-19 cases from the first year of the pandemic and ~440 000 randomly selected controls. CORIVA was linked to the death registry and all COVID-19 testing from the national health information system.

Databases included sociodemographic information, diagnoses, measurements, prescriptions and secondary care referrals and were linked to vaccine registries, including records of all administered vaccines from all healthcare settings. Data availability for CPRD Gold ended in December 2021, CPRD Aurum in January 2022, SIDIAP in June 2022 and CORIVA in December 2022.

All databases were mapped to the Observational Medical Outcomes Partnership Common Data Model (OMOP CDM)^16^ to facilitate federated analytics.

### Multinational network staggered cohort study: study design and participants

The study design has been published in detail elsewhere.^17^ Briefly, we used a staggered cohort design considering vaccination as a time-varying exposure. Four staggered cohorts were designed with each cohort representing a country-specific vaccination rollout phase (eg, dates when people became eligible for vaccination, and eligibility criteria).

The source population comprised all adults registered in the respective database for at least 180 days at the start of the study (4 January 2021 for CPRD Gold and Aurum, 20 February 2021 for SIDIAP and 28 January 2021 for CORIVA). Subsequently, each staggered cohort corresponded to an enrolment period: all people eligible for vaccination during this time were included in the cohort and people with a history of SARS-CoV-2 infection or COVID-19 vaccination before the start of the enrolment period were excluded. Across countries, cohort 1 comprised older age groups, whereas cohort 2 comprised individuals at risk for severe COVID-19. Cohort 3 included people aged ≥40 and cohort 4 enrolled people aged ≥18.

In each cohort, people receiving a first vaccine dose during the enrolment period were allocated to the vaccinated group, with their index date being the date of vaccination. Individuals who did not receive a vaccine dose comprised the unvaccinated group and their index date was assigned within the enrolment period, based on the distribution of index dates in the vaccinated group. People with COVID-19 before the index date were excluded.

Follow-up started from the index date until the earliest of end of available data, death, change in exposure status (first vaccine dose for those unvaccinated) or outcome of interest.

### COVID-19 vaccination

All vaccines approved within the study period from January 2021 to July 2021—namely, ChAdOx1 (Oxford/AstraZeneca), BNT162b2 (BioNTech/Pfizer]) Ad26.COV2.S (Janssen) and mRNA-1273 (Moderna), were included for this study.

### Post-COVID-19 outcomes of interest

Outcomes of interest were defined as SARS-CoV-2 infection followed by a predefined thromboembolic or cardiac event of interest within a year after infection, and with no record of the same clinical event in the 6 months before COVID-19. Outcome date was set as the corresponding SARS-CoV-2 infection date.

COVID-19 was identified from either a positive SARS-CoV-2 test (polymerase chain reaction (PCR) or antigen), or a clinical COVID-19 diagnosis, with no record of COVID-19 in the previous 6 weeks. This wash-out period was imposed to exclude re-recordings of the same COVID-19 episode.

Post-COVID-19 outcome events were selected based on previous studies.^11–13^ Events comprised ischaemic stroke (IS), haemorrhagic stroke (HS), transient ischaemic attack (TIA), ventricular arrhythmia/cardiac arrest (VACA), myocarditis/pericarditis (MP), myocardial infarction (MI), heart failure (HF), pulmonary embolism (PE) and deep vein thrombosis (DVT). We used two composite outcomes: (1) VTE, as an aggregate of PE and DVT and (2) ATE, as a composite of IS, TIA and MI. To avoid re-recording of the same complication we imposed a wash-out period of 90 days between records. Phenotypes for these complications were based on previously published studies.^3 4 8 18^

**Figure 1:**
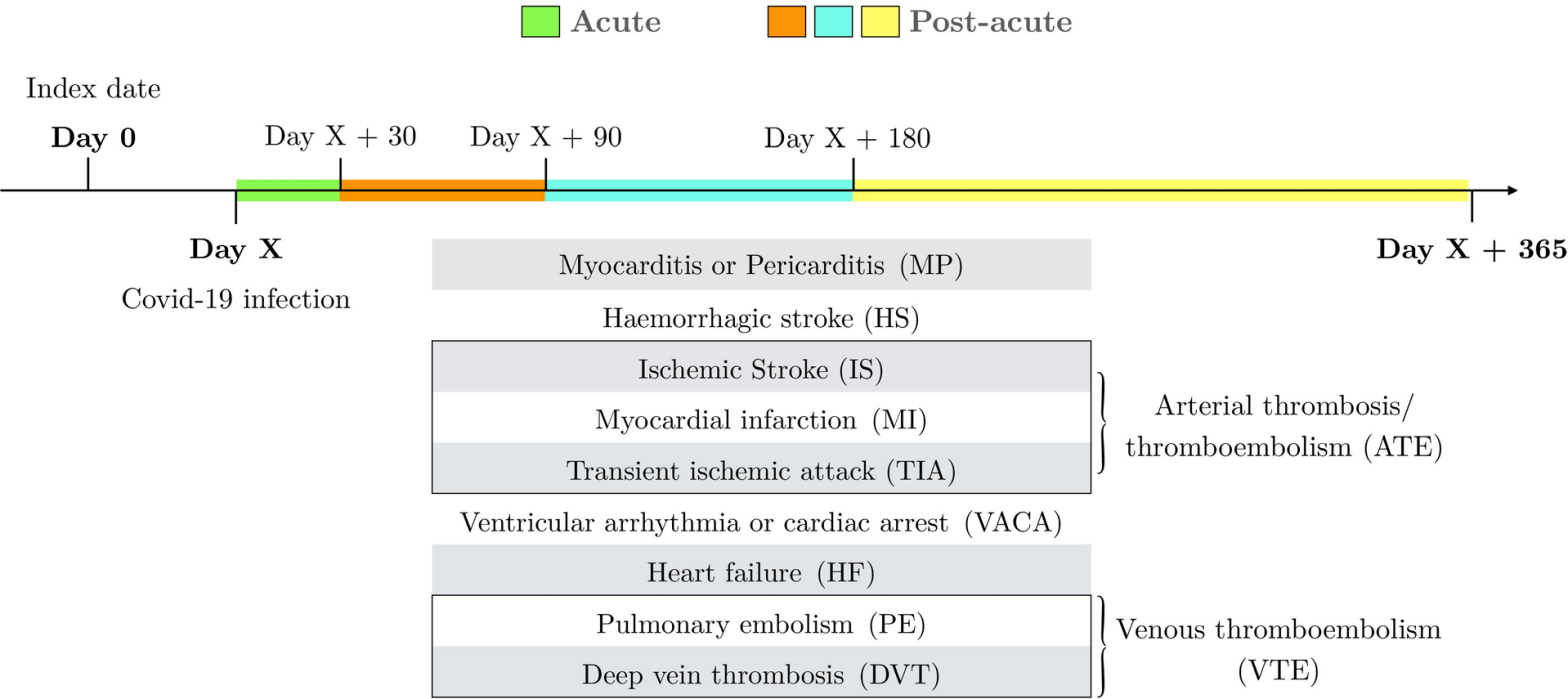
Study outcome design. Study outcomes of interest are defined as a COVID-19 infection followed by one of the complications in the figure, within a year after infection. Outcomes were ascertained in four different time windows after SARS-CoV-2 infection: 0–30 days (namely the acute phase), 31–90 days, 91–180 days and 181–365 days (these last three comprise the post-acute phase).

All outcomes were ascertained in four different time periods following SARS-CoV-2 infection: the first period described the acute infection phase—that is, 0–30 days after COVID-19, whereas the later periods - which are 31–90 days, 91–180 days and 181–365 days, illustrate the post-acute phase (figure 1).

### Negative control outcomes

Negative control outcomes (NCOs) were used to detect residual confounding. NCOs are outcomes which are not believed to be causally associated with the exposure, but share the same bias structure with the exposure and outcome of interest. Therefore, no significant association between exposure and NCO is to be expected. Our study used 43 different NCOs from previous work assessing vaccine effectiveness.^19^

### Statistical analysis

#### Federated network analyses

A template for an analytical script was developed and subsequently tailored to include the country-specific aspects (eg, dates, priority groups) for the vaccination rollout. Analyses were conducted locally for each database. Only aggregated data were shared and person counts <5 were clouded.

#### Propensity score weighting

Large-scale propensity scores (PS) were calculated to estimate the likelihood of a person receiving the vaccine based on their demographic and health-related characteristics (eg, conditions, medications) prior to the index date. PS were then used to minimise observed confounding by creating a weighted population (overlap weighting^20^), in which individuals contributed with a different weight based on their PS and vaccination status.

Prespecified key variables included in the PS comprised age, sex, location, index date, prior observation time in the database, number of previous outpatient visits and previous SARS-CoV-2 PCR/antigen tests. Regional vaccination, testing and COVID-19 incidence rates were also forced into the PS equation for the UK databases^21^ and SIDIAP.^22^ In addition, least absolute shrinkage and selection operator (LASSO) regression, a technique for variable selection, was used to identify additional variables from all recorded conditions and prescriptions within 0–30 days, 31–180 days and 181-any time (conditions only) before the index date that had a prevalence of >0.5% in the study population.

PS were then separately estimated for each staggered cohort and analysis. We considered covariate balance to be achieved if absolute standardised mean differences (ASMDs) were ≤0.1 after weighting. Baseline characteristics such as demographics and comorbidities were reported.

#### Effect estimation

To account for the competing risk of death associated with COVID-19, Fine-and-Grey models^23^ were used to calculate subdistribution hazard ratios (sHRs). Subsequently, sHRs and confidence intervals were empirically calibrated from NCO estimates^24^ to account for unmeasured confounding. To calibrate the estimates, the empirical null distribution was derived from NCO estimates and was used to compute calibrated confidence intervals. For each outcome, sHRs from the four staggered cohorts were pooled using random-effect meta-analysis, both separately for each database and across all four databases.

### Sensitivity analysis

Sensitivity analyses comprised 1) censoring follow-up for vaccinated people at the time when they received their second vaccine dose and 2) considering only the first post-COVID-19 outcome within the year after infection (online supplemental figure S1). In addition, comparative effectiveness analyses were conducted for BNT162b2 versus ChAdOx1.

10.1136/heartjnl-2023-323483.supp1 Supplementary data

### Data and code availability

All analytic code for the study is available in GitHub (https://github.com/oxford-pharmacoepi/vaccineEffectOnPostCovidCardiacThromboembolicEvents), including code lists for vaccines, COVID-19 tests and diagnoses, cardiac and thromboembolic events, NCO and health conditions to prioritise patients for vaccination in each country. We used R version 4.2.3 and statistical packages survival (3.5–3), Empirical Calibration (3.1.1), glmnet (4.1-7), and Hmisc (5.0–1).

### Patient and public involvement

Owing to the nature of the study and the limitations regarding data privacy, the study design, analysis, interpretation of data and revision of the manuscript did not involve any patients or members of the public.

## Results

All aggregated results are available in a web application (https://dpa-pde-oxford.shinyapps.io/PostCovidComplications/).

We included over 10.17 million vaccinated individuals (1 618 395 from CPRD Gold; 5 729 800 from CPRD Aurum; 2 744 821 from SIDIAP and 77 603 from CORIVA) and 10.39 million unvaccinated individuals (1 640 371; 5 860 564; 2 588 518 and 302 267, respectively). Online supplemental figures S2-5 illustrate study inclusion for each database.

Adequate covariate balance was achieved after PS weighting in most studies: CORIVA (all cohorts) and SIDIAP (cohorts 1 and 4) did not contribute to ChAdOx1 subanalyses owing to sample size and covariate imbalance. ASMD results are accessible in the web application.

NCO analyses suggested residual bias after PS weighting, with a majority of NCOs associated positively with vaccination. Therefore, calibrated estimates are reported in this manuscript. Uncalibrated effect estimates and NCO analyses are available in the web interface.

### Population characteristics

Table 1 presents baseline characteristics for the weighted populations in CPRD Aurum, for illustrative purposes. Online supplemental tables S1-25 summarise baseline characteristics for weighted and unweighted populations for each database and comparison. Across databases and cohorts, populations followed similar patterns: cohort 1 represented an older subpopulation (around 80 years old) with a high proportion of women (57%). Median age was lowest in cohort 4 ranging between 30 and 40 years.

**Table 1:** Characteristics of weighted populations in CPRD Aurum database, stratified by staggered cohort and exposure status. Exposure is any COVID-19 vaccine

Characteristics	Cohort 1			Cohort 2			Cohort 3			Cohort 4		
	Unvaccinated	Vaccinated	ASMD	Unvaccinated	Vaccinated	ASMD	Unvaccinated	Vaccinated	ASMD	Unvaccinated	Vaccinated	ASMD
**No (individuals)**	154 864	154 245		420 707	420 931		463 495	462 463		818 917	827 124	
**Age, median (Q25–Q75)**	80 (76–84)	80 (76–84)	0.000	58 (44–67)	58 (44–67)	0.005	50 (41–58)	52 (40–58)	0.003	34 (26–42)	34 (26–42)	0.004
**Sex: female, N (%)**	88 349 (57%)	87 639 (57%)	0.005	248 156 (59%)	249 561 (59%)	0.006	245 248 (53%)	245 600 (53%)	0.004	351 435 (43%)	358 688 (43%)	0.009
Years of prior history*, median (Q25–Q75)	24 (10–35)	24 (10–36)	0.006	18 (8–29)	18 (8–29)	0.003	14 (6–24)	14 (7–24)	0.008	8 (4–17)	7 (3–18)	0.001
**Number of GP visits, median (Q25–Q75)**	10 (5–18)	10 (6–17)		8 (3–15)	8 (5–14)		4 (1–11)	6(3-11)		2 (0–6)	2 (0–6)	
**Number of PCR tests, median (Q25–Q75)**	0 (0–0)	0 (0–0)		0 (0–0)	0 (0–0)		0 (0–0)	0 (0–0)		0 (0–0)	0 (0–0)	
**Comorbidities†, N (%)**												
Anxiety	23 200 (15%)	22 789 (15%)	0.006	94 390 (22%)	91 644 (22%)	0.016	92 820 (20%)	90 807 (20%)	0.010	123 055 (15%)	125 202 (15%)	0.003
Asthma	16 978 (11%)	16 663 (11%)	0.005	95 770 (23%)	94 550 (22%)	0.007	79 642 (17%)	78 266 (17%)	0.007	63 687 (8%)	61 472 (7%)	0.013
Chronic kidney disease	36 149 (23%)	36 046 (23%)	0.001	28 181 (7%)	29 756 (7%)	0.015	10 283 (2%)	10 577 (2%)	0.005	3840 (0%)	3572 (0%)	0.006
COPD	13 385 (9%)	13 181 (9%)	0.003	17 447 (4%)	17 999 (4%)	0.006	6062 (1%)	5754 (1%)	0.006	1901 (0%)	1918 (0%)	0.000
Dementia	9483 (6%)	8517 (6%)	0.026	4182 (1%)	3879 (1%)	0.007	1361 (0%)	1392 (0%)	0.001	276 (0%)	495 (0%)	0.012
Depressive disorder	18 632 (12%)	18 547 (12%)	0.000	85 280 (20%)	81 945 (19%)	0.020	81 891 (18%)	79 804 (17%)	0.011	94 373 (12%)	97 053 (12%)	0.007
Diabetes	29 365 (19%)	28 831 (19%)	0.007	49 408 (12%)	48 562 (12%)	0.006	26 616 (6%)	28 628 (6%)	0.019	12 787 (2%)	12 539 (2%)	0.004
GORD	8718 (6%)	8515 (6%)	0.005	19 907 (5%)	18 924 (4%)	0.011	15 646 (3%)	14 982 (3%)	0.008	13 882 (2%)	13 893 (2%)	0.001
Heart failure	9349 (6%)	8851 (6%)	0.013	7284 (2%)	6502 (2%)	0.015	2660 (1%)	2470 (1%)	0.005	930 (0%)	816 (0%)	0.005
Hypertension	81 563 (53%)	80 806 (52%)	0.006	97 707 (23%)	98 193 (23%)	0.002	54 649 (12%)	55 798 (12%)	0.008	22 925 (3%)	24 450 (3%)	0.009
Hypothyroidism	15 125 (10%)	15 098 (10%)	0.001	25 579 (6%)	25 962 (6%)	0.004	17 162 (4%)	17 580 (4%)	0.005	12 427 (2%)	12 641 (2%)	0.001
Malignant neoplastic disease	33 467 (22%)	33 024 (21%)	0.005	30 194 (7%)	35 085 (8%)	0.043	14 815 (3%)	14 140 (3%)	0.008	6447 (1%)	5766 (1%)	0.011
Myocardial infarction	7824 (5%)	7731 (5%)	0.002	9964 (2%)	11 319 (3%)	0.020	3787 (1%)	3664 (1%)	0.003	1315 (0%)	1069 (0%)	0.008
Osteoporosis	15 275 (10%)	15 373 (10%)	0.003	10 626 (3%)	10 718 (3%)	0.001	4113 (1%)	4131 (1%)	0.001	1376 (0%)	1472 (0%)	0.002
Pneumonia	8573 (6%)	7621 (5%)	0.027	11 355 (3%)	10 691 (3%)	0.010	6651 (1%)	6545 (1%)	0.002	5144 (1%)	5151 (1%)	0.001
Rheumatoid arthritis	3066 (2%)	3092 (2%)	0.002	6198 (1%)	6570 (2%)	0.007	2355 (1%)	3111 (1%)	0.021	1201 (0%)	859 (0%)	0.012
Stroke	7667 (5%)	7047 (5%)	0.018	8041 (2%)	8794 (2%)	0.013	3518 (1%)	3293 (1%)	0.006	1496 (0%)	1305 (0%)	0.006
Venous thromboembolism	9589 (6%)	9241 (6%)	0.008	11 836 (3%)	12 475 (3%)	0.009	6503 (1%)	8075 (2%)	0.028	4661 (1%)	2441 (0%)	0.042

The four cohorts represent vaccine rollout periods.

*Calculated as the days of previous observation in the database before index date.

†Assessed any time before index date.

ASMD, absolute standardised mean difference; COPD, chronic obstructive pulmonary disease; GORD, gastro-oesophageal reflux disease; GP, general practitioner; PCR, polymerase chain reaction.

### COVID-19 vaccination and post-COVID-19 complications

Table 2 shows the incidence of post-COVID-19 VTE, ATE and HF, the three most common post-COVID-19 conditions among the studied outcomes. Outcome counts are presented separately for 0–30, 31–90, 91–180 and 181–365 days after SARS-CoV-2 infection. Online supplemental tables S26-36 include all studied complications, also for the sensitivity and subanalyses. Similar pattern for incidences were observed across all databases: higher outcome rates in the older populations (cohort 1) and decreasing frequency with increasing time after infection in all cohorts.

**Table 2:** Number of records (and risk per 10 000 individuals) for acute and post-acute COVID-19 cardiac and thromboembolic complications, across cohorts and databases for any COVID-19 vaccination

Cohort	Time window	Outcome	CPRD Aurum		CORIVA		CPRD Gold		SIDIAP	
			Unvaccinated	Vaccinated	Unvaccinated	Vaccinated	Unvaccinated	Vaccinated	Unvaccinated	Vaccinated
**Cohort 1**			n=346 674	n=552 602	n=23 982	n=26 736	n=169 100	n=118 507	n=223 962	n=89 941
	**0 to 30 days**	**VTE**	93 (2.68)	117 (2.12)	77 (32.11)	45 (16.83)	8 (0.47)	8 (0.68)	74 (3.30)	96 (10.67)
		**ATE**	22 (0.63)	70 (1.27)	110 (45.87)	81 (30.30)	6 (0.35)	7 (0.59)	77 (3.44)	208 (23.13)
		**HF**	59 (1.70)	198 (3.58)	395 (164.71)	299 (111.83)	10 (0.59)	9 (0.76)	302 (13.48)	640 (71.16)
	**31 to 90 days**	**VTE**	19 (0.55)	40 (0.72)	37 (15.43)	30 (11.22)	< 5	< 5	16 (0.71)	46 (5.11)
		**ATE**	5 (0.14)	43 (0.78)	33 (13.76)	47 (17.58)	< 5	< 5	41 (1.83)	130 (14.45)
		**HF**	30 (0.87)	113 (2.04)	151 (62.96)	170 (63.58)	< 5	8 (0.68)	89 (3.97)	298 (33.13)
	**91 to 180 days**	**VTE**	10 (0.29)	21 (0.38)	21 (8.76)	35 (13.09)	< 5	< 5	20 (0.89)	40 (4.45)
		**ATE**	11 (0.32)	28 (0.51)	31 (12.93)	52 (19.45)	< 5	6 (0.51)	30 (1.34)	112 (12.45)
		**HF**	37 (1.07)	95 (1.72)	162 (67.55)	220 (82.29)	< 5	5 (0.42)	87 (3.88)	252 (28.02)
	**181 to 365 days**	**VTE**	10 (0.29)	11 (0.20)	45 (18.76)	35 (13.09)	< 5	< 5	10 (0.45)	13 (1.45)
		**ATE**	10 (0.29)	23 (0.42)	55 (22.93)	82 (30.67)	< 5	< 5	42 (1.88)	53 (5.89)
		**HF**	40 (1.15)	58 (1.05)	268 (111.75)	321 (120.06)	< 5	6 (0.51)	86 (3.84)	149 (16.57)
**Cohort 2**			n=1 975 726	**n=**1 563 569	n=34 317	n=4572	n=5 83 399	n=4 86 619	n=4 33 151	n=8 19 590
	**0 to 30 days**	**VTE**	241 (1.22)	220 (1.41)	79 (23.02)	7 (15.31)	31 (0.53)	24 (0.49)	258 (5.96)	400 (4.88)
		**ATE**	41 (0.21)	104 (0.67)	110 (32.05)	5 (10.94)	< 5	6 (0.12)	173 (3.99)	669 (8.16)
		**HF**	45 (0.23)	146 (0.93)	364 (106.07)	23 (50.31)	5 (0.09)	13 (0.27)	378 (8.73)	1331 (16.24)
	**31 to 90 days**	**VTE**	43 (0.22)	76 (0.49)	31 (9.03)	5 (10.94)	< 5	9 (0.18)	59 (1.36)	195 (2.38)
		**ATE**	18 (0.09)	93 (0.59)	32 (9.32)	< 5	< 5	9 (0.18)	85 (1.96)	444 (5.42)
		**HF**	27 (0.14)	103 (0.66)	149 (43.42)	19 (41.56)	< 5	7 (0.14)	138 (3.19)	643 (7.85)
	**91 to 180 days**	**VTE**	28 (0.14)	40 (0.26)	26 (7.58)	6 (13.12)	6 (0.10)	< 5	58 (1.34)	125 (1.53)
		**ATE**	17 (0.09)	43 (0.28)	32 (9.32)	< 5	< 5	< 5	91 (2.10)	417 (5.09)
		**HF**	22 (0.11)	69 (0.44)	166 (48.37)	21 (45.93)	< 5	< 5	110 (2.54)	579 (7.06)
	**181 to 365 days**	**VTE**	9 (0.05)	13 (0.08)	44 (12.82)	8 (17.50)	< 5	< 5	16 (0.37)	64 (0.78)
		**ATE**	12 (0.06)	18 (0.12)	53 (15.44)	< 5	< 5	< 5	63 (1.45)	178 (2.17)
		**HF**	20 (0.10)	35 (0.22)	259 (75.47)	33 (72.18)	< 5	< 5	81 (1.87)	246 (3.00)
**Cohort 3**			n=1 510 401	n=1 528 031	n=96 423	n=24 050	n=4 17 996	n=4 62 832	n=8 69 497	n=9 54 232
	**0 to 30 days**	**VTE**	245 (1.62)	142 (0.93)	115 (11.93)	9 (3.74)	27 (0.65)	17 (0.37)	325 (3.74)	180 (1.89)
		**ATE**	29 (0.19)	49 (0.32)	119 (12.34)	12 (4.99)	< 5	12 (0.26)	213 (2.45)	275 (2.88)
		**HF**	31 (0.21)	38 (0.25)	380 (39.41)	23 (9.56)	< 5	< 5	364 (4.19)	256 (2.68)
	**31 to 90 days**	**VTE**	44 (0.29)	46 (0.30)	50 (5.19)	10 (4.16)	< 5	7 (0.15)	85 (0.98)	92 (0.96)
		**ATE**	11 (0.07)	33 (0.22)	48 (4.98)	9 (3.74)	< 5	8 (0.17)	109 (1.25)	210 (2.20)
		**HF**	15 (0.10)	26 (0.17)	180 (18.67)	25 (10.40)	< 5	< 5	137 (1.58)	157 (1.65)
	**91 to 180 days**	**VTE**	24 (0.16)	26 (0.17)	43 (4.46)	11 (4.57)	< 5	< 5	64 (0.74)	101 (1.06)
		**ATE**	< 5	28 (0.18)	44 (4.56)	10 (4.16)	< 5	< 5	113 (1.30)	206 (2.16)
		**HF**	11 (0.07)	14 (0.09)	216 (22.40)	30 (12.47)	< 5	< 5	120 (1.38)	138 (1.45)
	**181 to 365 days**	**VTE**	< 5	11 (0.07)	72 (7.47)	17 (7.07)	< 5	< 5	34 (0.39)	26 (0.27)
		**ATE**	< 5	< 5	80 (8.30)	8 (3.33)	< 5	< 5	51 (0.59)	67 (0.70)
		**HF**	5 (0.03)	< 5	324 (33.60)	37 (15.38)	< 5	< 5	62 (0.71)	44 (0.46)
**Cohort 4**			n=2 027 763	n=2 085 598	n=1 47 545	n=22 245	n=4 69 876	n=5 50 437	n=1 061 634	n=8 80 950
	**0 to 30 days**	**VTE**	334 (1.65)	50 (0.24)	116 (7.86)	< 5	36 (0.77)	11 (0.20)	350 (3.30)	98 (1.11)
		**ATE**	26 (0.13)	8 (0.04)	116 (7.86)	10 (4.50)	< 5	< 5	231 (2.18)	95 (1.08)
		**HF**	28 (0.14)	< 5	364 (24.67)	17 (7.64)	< 5	< 5	362 (3.41)	75 (0.85)
	**31 to 90 days**	**VTE**	58 (0.29)	22 (0.11)	54 (3.66)	< 5	5 (0.11)	< 5	91 (0.86)	49 (0.56)
		**ATE**	12 (0.06)	9 (0.04)	46 (3.12)	5 (2.25)	< 5	< 5	118 (1.11)	76 (0.86)
		**HF**	14 (0.07)	9 (0.04)	176 (11.93)	13 (5.84)	< 5	< 5	142 (1.34)	47 (0.53)
	**91 to 180 days**	**VTE**	26 (0.13)	10 (0.05)	49 (3.32)	5 (2.25)	< 5	< 5	71 (0.67)	60 (0.68)
		**ATE**	< 5	6 (0.03)	41 (2.78)	7 (3.15)	< 5	< 5	128 (1.21)	90 (1.02)
		**HF**	10 (0.05)	< 5	208 (14.10)	18 (8.09)	< 5	< 5	139 (1.31)	55 (0.62)
	**181 to 365 days**	**VTE**	< 5	< 5	77 (5.22)	< 5	< 5	< 5	46 (0.43)	12 (0.14)
		**ATE**	< 5	< 5	73 (4.95)	9 (4.05)	< 5	< 5	54 (0.51)	28 (0.32)
		**HF**	< 5	< 5	301 (20.40)	16 (7.19)	< 5	< 5	57 (0.54)	15 (0.17)

The four cohorts represent vaccine rollout periods.

ATE, arterial thrombosis/thromboembolism (Ischaemic stroke+transient ischaemic attack+myocardial infarction); HF, heart failure; VTE, venous thromboembolism (deep vein thrombosis+pulmonary embolism).

**Figure 2:**
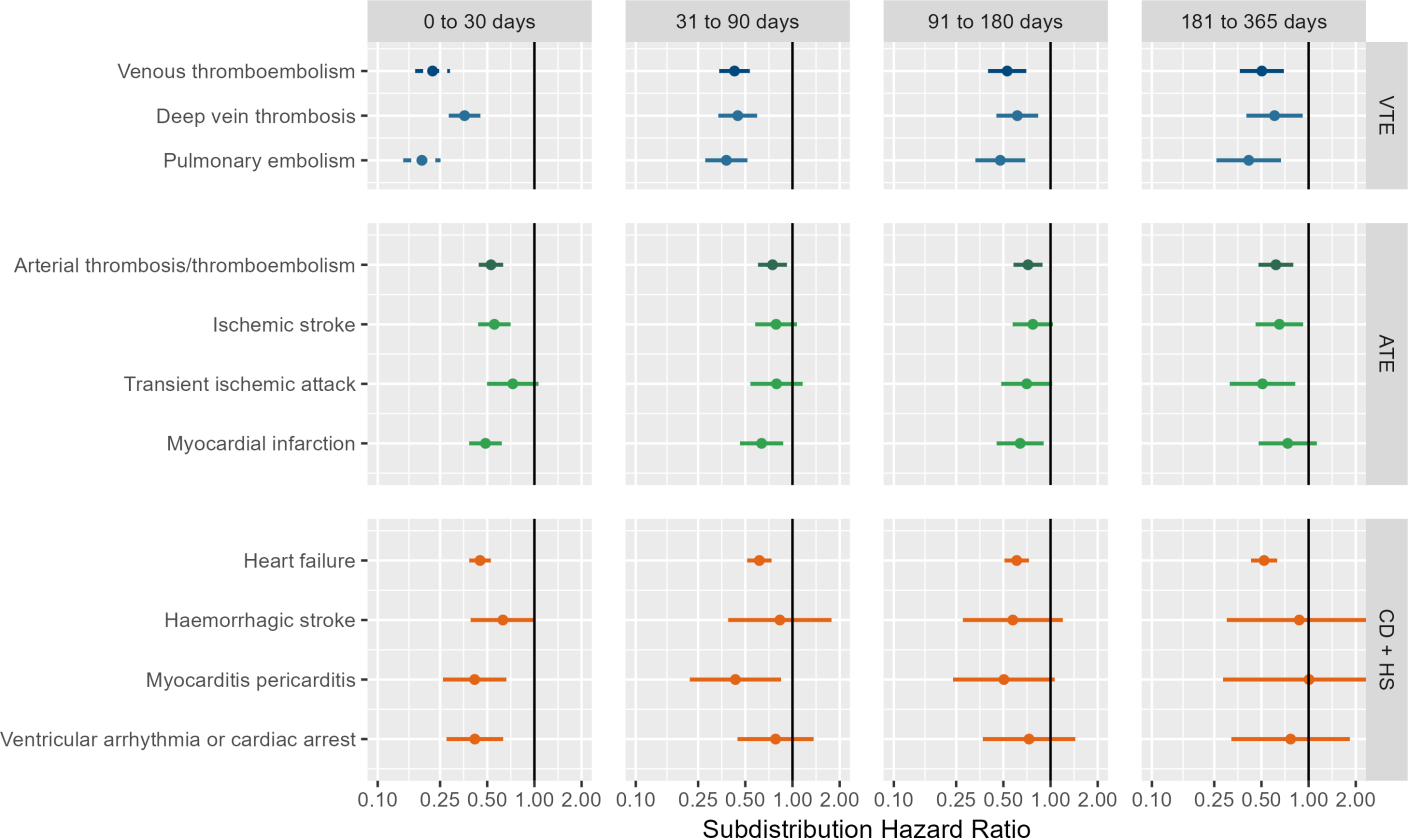
Forest plots for the effect of COVID-19 vaccines on post-COVID-19 cardiac and thromboembolic complications; meta-analysis across cohorts and databases. Dashed line represents a level of heterogeneity I^2^>0.4. ATE, arterial thrombosis/thromboembolism; CD+HS, cardiac diseases and haemorrhagic stroke; VTE, venous thromboembolism.

Results from calibrated estimates pooled in meta-analysis across cohorts and databases are shown in figure 2.

Reduced risk associated with vaccination is observed for acute and post-acute VTE, DVT, and PE: acute meta-analytic sHR are 0.22 (95% CI, 0.17–0.29); 0.36 (0.28–0.45); and 0.19 (0.15–0.25), respectively. For VTE in the post-acute phase, sHR estimates are 0.43 (0.34–0.53), 0.53 (0.40–0.70) and 0.50 (0.36–0.70) for 31–90, 91–180, and 181–365 days post COVID-19, respectively. Reduced risk of VTE outcomes was observed in vaccinated across databases and cohorts, see online supplemental figures S14–22.

Similarly, the risk of ATE, IS and MI in the acute phase after infection was reduced for the vaccinated group, sHR of 0.53 (0.44–0.63), 0.55 (0.43–0.70) and 0.49 (0.38–0.62), respectively. Reduced risk associated with vaccination persisted for post-acute ATE, with sHR of 0.74 (0.60–0.92), 0.72 (0.58–0.88) and 0.62 (0.48–0.80) for 31–90, 91–180 and 181–365 days post-COVID-19, respectively. Risk of post-acute MI remained lower for vaccinated in the 31–90 and 91–180 days after COVID-19, with sHR of 0.64 (0.46–0.87) and 0.64 (0.45–0.90), respectively. Vaccination effect on post-COVID-19 TIA was seen only in the 181–365 days, with sHR of 0.51 (0.31–0.82). Online supplemental figures S23-31 show database-specific and cohort-specific estimates for ATE-related complications.

Risk of post-COVID-19 cardiac complications was reduced in vaccinated individuals. Meta-analytic estimates in the acute phase showed sHR of 0.45 (0.38–0.53) for HF, 0.41 (0.26–0.66) for MP and 0.41 (0.27–0.63) for VACA. Reduced risk persisted for post-acute COVID-19 HF: sHR 0.61 (0.51–0.73) for 31–90 days, 0.61 (0.51–0.73) for 91–180 days and 0.52 (0.43–0.63) for 181–365 days. For post-acute MP, risk was only lowered in the first post-acute window (31–90 days), with sHR of 0.43 (0.21–0.85). Vaccination showed no association with post-COVID-19 HS. Database-specific and cohort-specific results for these cardiac diseases are shown in online supplemental figures S32-40.

Stratified analyses by vaccine showed similar associations, except for ChAdOx1 which was not associated with reduced VTE and ATE risk in the last post-acute window. Sensitivity analyses were consistent with main results (online supplemental figures S6-13).

Figure 3 shows the results of comparative effects of BNT162b2 versus ChAdOx1, based on UK data. Meta-analytic estimates favoured BNT162b2 (sHR of 0.66 (0.46–0.93)) for VTE in the 0–30 days after infection, but no differences were seen for post-acute VTE or for any of the other outcomes. Results from sensitivity analyses, database-specific and cohort-specific estimates were in line with the main findings (online supplemental figures S41-51).

**Figure 3:**
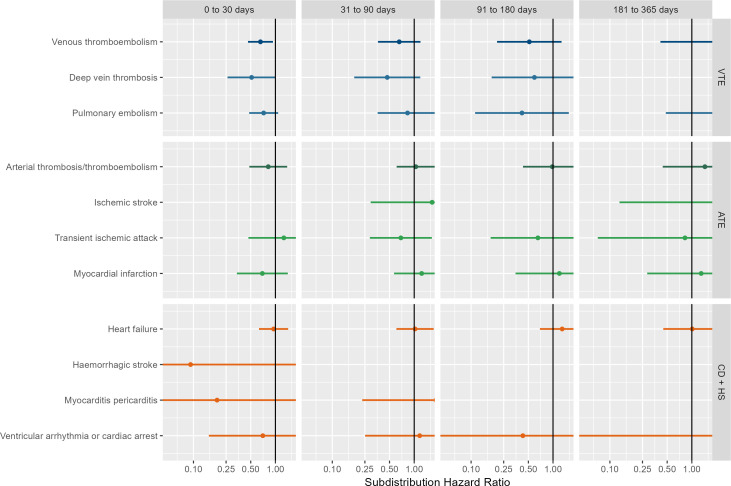
Forest plots for comparative vaccine effect (BNT162b2 vs ChAdOx1); meta-analysis across cohorts and databases. ATE, arterial thrombosis/thromboembolism; CD+HS, cardiac diseases and haemorrhagic stroke; VTE, venous thromboembolism.

## Discussion

### Key findings

Our analyses showed a substantial reduction of risk (45–81%) for thromboembolic and cardiac events in the acute phase of COVID-19 associated with vaccination. This finding was consistent across four databases and three different European countries. Risks for post-acute COVID-19 VTE, ATE and HF were reduced to a lesser extent (24–58%), whereas a reduced risk for post-COVID-19 MP and VACA in vaccinated people was seen only in the acute phase.

### Results in context

The relationship between SARS-CoV-2 infection, COVID-19 vaccines and thromboembolic and/or cardiac complications is tangled. Some large studies report an increased risk of VTE and ATE following both ChAdOx1 and BNT162b2 vaccination,^7^ whereas other studies have not identified such a risk.^25^ Elevated risk of VTE has also been reported among patients with COVID-19 and its occurrence can lead to poor prognosis and mortality.^26 27^ Similarly, several observational studies have found an association between COVID-19 mRNA vaccination and a short-term increased risk of myocarditis, particularly among younger male individuals.^5 6^ For instance, a self-controlled case series study conducted in England revealed about 30% increased risk of hospital admission due to myocarditis within 28 days following both ChAdOx1 and BNT162b2 vaccines. However, this same study also found a ninefold higher risk for myocarditis following a positive SARS-CoV-2 test, clearly offsetting the observed post-vaccine risk.

COVID-19 vaccines have demonstrated high efficacy and effectiveness in preventing infection and reducing the severity of acute-phase infection. However, with the emergence of newer variants of the virus, such as omicron, and the waning protective effect of the vaccine over time, there is a growing interest in understanding whether the vaccine can also reduce the risk of complications after breakthrough infections. Recent studies suggested that COVID-19 vaccination could potentially protect against acute post-COVID-19 cardiac and thromboembolic events.^11 12^ A large prospective cohort study^11^ reports risk of VTE after SARS-CoV-2 infection to be substantially reduced in fully vaccinated ambulatory patients. Likewise, Al-Aly *et al* 12 suggest a reduced risk for post-acute COVID-19 conditions in breakthrough infection versus SARS-CoV-2 infection without prior vaccination. However, the populations were limited to SARS-CoV-2 infected individuals and estimates did not include the effect of the vaccine to prevent COVID-19 in the first place. Other studies on post-acute COVID-19 conditions and symptoms have been conducted,^28 29^ but there has been limited reporting on the condition-specific risks associated with COVID-19, even though the prognosis for different complications can vary significantly.

In line with previous studies, our findings suggest a potential benefit of vaccination in reducing the risk of post-COVID-19 thromboembolic and cardiac complications. We included broader populations, estimated the risk in both acute and post-acute infection phases and replicated these using four large independent observational databases. By pooling results across different settings, we provided the most up-to-date and robust evidence on this topic.

### Strengths and limitations

The study has several strengths. Our multinational study covering different healthcare systems and settings showed consistent results across all databases, which highlights the robustness and replicability of our findings. All databases had complete recordings of vaccination status (date and vaccine) and are representative of the respective general population. Algorithms to identify study outcomes were used in previous published network studies, including regulatory-funded research.^3 4 8 18^ Other strengths are the staggered cohort design which minimises confounding by indication and immortal time bias. PS overlap weighting and NCO empirical calibration have been shown to adequately minimise bias in vaccine effectiveness studies.^19^ Furthermore, our estimates include the vaccine effectiveness against COVID-19, which is crucial in the pathway to experience post-COVID-19 complications.

Our study has some limitations. The use of real-world data comes with inherent limitations including data quality concerns and risk of confounding. To deal with these limitations, we employed state-of-the-art methods, including large-scale propensity score weighting and calibration of effect estimates using NCO.^19 24^ A recent study^30^ has demonstrated that methodologically sound observational studies based on routinely collected data can produce results similar to those of clinical trials. We acknowledge that results from NCO were positively associated with vaccination, and estimates might still be influenced by residual bias despite using calibration. Another limitation is potential under-reporting of post-COVID-19 complications: some asymptomatic and mild COVID-19 infections might have not been recorded. Additionally, post-COVID-19 outcomes of interest might be under-recorded in primary care databases (CPRD Aurum and Gold) without hospital linkage, which represent a large proportion of the data in the study. However, results in SIDIAP and CORIVA, which include secondary care data, were similar. Also, our study included a small number of young men and male teenagers, who were the main population concerned with increased risks of myocarditis/pericarditis following vaccination.

## Conclusions

Vaccination against SARS-CoV-2 substantially reduced the risk of acute post-COVID-19 thromboembolic and cardiac complications, probably through a reduction in the risk of SARS-CoV-2 infection and the severity of COVID-19 disease due to vaccine-induced immunity. Reduced risk in vaccinated people lasted for up to 1 year for post-COVID-19 VTE, ATE and HF, but not clearly for other complications. Findings from this study highlight yet another benefit of COVID-19 vaccination. However, further research is needed on the possible waning of the risk reduction over time and on the impact of booster vaccination.

## Data Availability

Data may be obtained from a third party and are not publicly available. CPRD: CPRD data were obtained under the CPRD multi-study license held by the University of Oxford after Research Data Governance (RDG) approval. Direct data sharing is not allowed. SIDIAP: In accordance with current European and national law, the data used in this study is only available for the researchers participating in this study. Thus, we are not allowed to distribute or make publicly available the data to other parties. However, researchers from public institutions can request data from SIDIAP if they comply with certain requirements. Further information is available online (https://www.sidiap.org/index.php/menu-solicitudesen/application-proccedure) or by contacting SIDIAP (sidiap@idiapjgol.org). CORIVA: CORIVA data were obtained under the approval of Research Ethics Committee of the University of Tartu and the patient level data sharing is not allowed. All analyses in this study were conducted in a federated manner, where analytical code and aggregated (anonymised) results were shared, but no patient-level data was transferred across the collaborating institutions.
