# Supplementary material for: The role of COVID-19 vaccines in preventing post-COVID-19 thromboembolic and cardiovascular complications
Source: Heart. 2024 Mar 12;110(9):635–43. doi: 10.1136/heartjnl-2023-323483 (PMC11041555; doi:10.1136/heartjnl-2023-323483)
Supplement: Supplementary data [file heartjnl-2023-323483supp001.pdf]

SUPPLEMENT

The effectiveness of COVID-19 vaccines to prevent post COVID cardiac and thromboembolic complications.

Table of contents

Figure S1: Follow-up in vaccinated and unvaccinated cohorts. 6

(A) Main analysis, (B) Follow-up ends at first vaccine dose after index date, (C) Post COVID sequelae refers just to the first outcome after infection. 6

Figure S2: Study Inclusion Flowchart CPRD AURUM 7

Figure S3: Study Inclusion Flowchart CPRD GOLD. 8

Figure S4: Study Inclusion Flowchart SIDIAP. 9

Figure S5: Study Inclusion Flowchart CORIVA. 10

Table S1: Characteristics of unweighted populations in CPRD AURUM, database, stratified by staggered cohort and exposure status. Exposure is any COVID-19 vaccine. 11

Table S2: Characteristics of weighted populations in CPRD AURUM, database, stratified by staggered cohort and exposure status. Exposure is ChAdOx1 vaccine. 13

Table S3: Characteristics of unweighted populations in CPRD AURUM, database, stratified by staggered cohort and exposure status. Exposure is ChAdOx1 vaccine. 15

Table S4: Characteristics of weighted populations in CPRD AURUM, database, stratified by staggered cohort and exposure status. Exposure is BNT162b2 vaccine. 17

Table S5: Characteristics of unweighted populations in CPRD AURUM, database, stratified by staggered cohort and exposure status. Exposure is BNT162b2 vaccine. 19

Table S6: Characteristics of weighted populations in CPRD GOLD, database, stratified by staggered cohort and exposure status. Exposure is any COVID-19 vaccine. 21

Table S7: Characteristics of unweighted populations in CPRD GOLD, database, stratified by staggered cohort and exposure status. Exposure is any COVID-19 vaccine. 23

Table S8: Characteristics of weighted populations in CPRD GOLD, database, stratified by staggered cohort and exposure status. Exposure is ChAdOx1 vaccine. 25

Table S9: Characteristics of unweighted populations in CPRD GOLD, database, stratified by staggered cohort and exposure status. Exposure is ChAdOx1 vaccine. 27

Table S10: Characteristics of weighted populations in CPRD GOLD, database, stratified by staggered cohort and exposure status. Exposure is BNT162b2 vaccine. 29

Table S11: Characteristics of unweighted populations in CPRD GOLD, database, stratified by staggered cohort and exposure status. Exposure is BNT162b2 vaccine. 31

Table S12: Characteristics of weighted populations in SIDIAP, database, stratified by staggered cohort and exposure status. Exposure is any COVID-19 vaccine. 33

Table S13: Characteristics of unweighted populations in SIDIAP, database, stratified by staggered cohort and exposure status. Exposure is any COVID-19 vaccine. 35

Table S14: Characteristics of weighted populations in SIDIAP, database, stratified by staggered cohort and exposure status. Exposure is BNT162b2 vaccine. 37

Table S15: Characteristics of unweighted populations in SIDIAP, database, stratified by staggered cohort and exposure status. Exposure is BNT162b2 vaccine. 39

Table S16: Characteristics of weighted populations in SIDIAP, database, stratified by staggered cohort and exposure status. Exposure is ChAdOx1 vaccine. 41

Mercadé-Besora N, *et al. Heart* 2024; 110:635–643. doi: 10.1136/heartjnl-2023-323483

Table S17: Characteristics of unweighted populations in SIDIAP, database, stratified by staggered cohort and exposure status. Exposure is ChAdOx1 vaccine. 43

Table S18: Characteristics of weighted populations in CORIVA, database, stratified by staggered cohort and exposure status. Exposure is any COVID-19 vaccine. 45

Table S19: Characteristics of unweighted populations in CORIVA, database, stratified by staggered cohort and exposure status. Exposure is any COVID-19 vaccine. 47

Table S20: Characteristics of weighted populations in CORIVA, database, stratified by staggered cohort and exposure status. Exposure is BNT162b2 vaccine. 49

Table S21: Characteristics of unweighted populations in CORIVA, database, stratified by staggered cohort and exposure status. Exposure is BNT162b2 vaccine. 51

Table S22: Characteristics of weighted populations in CPRD AURUM database, stratified by staggered cohort and vaccine. 53

Table S23: Characteristics of unweighted populations in CPRD AURUM database, stratified by staggered cohort and vaccine. 55

Table S24: Characteristics of weighted populations in CPRD GOLD database, stratified by staggered cohort and vaccine. 57

Table S25: Characteristics of unweighted populations in CPRD GOLD database, stratified by staggered cohort and vaccine. 59

Table S26: Number of records (and risk per 10,000 individuals) for post COVID-19 cardiac and thromboembolic complications, across cohorts and databases, stratified by exposure status (any COVID-19 vaccine). Follow-up ends at first vaccine dose after index date. 61

Table S27: Number of records (and risk per 10,000 individuals) for post COVID-19 cardiac and thromboembolic complications, across cohorts and databases, stratified by exposure status (any COVID-19 vaccine). Only first outcome after COVID-19 captured. 66

Table S28: Number of records (and risk per 10,000 individuals) for post COVID-19 cardiac and thromboembolic complications, across cohorts and databases, stratified by exposure status (ChAdOx1 vaccine). 71

Table S29: Number of records (and risk per 10,000 individuals) for post COVID-19 cardiac and thromboembolic complications, across cohorts and databases, stratified by exposure status (ChAdOx1 vaccine). Follow-up ends at first vaccine dose after index date. 76

Table S30: Number of records (and risk per 10,000 individuals) for post COVID-19 cardiac and thromboembolic complications, across cohorts and databases, stratified by exposure status (ChAdOx1 vaccine). Only first outcome after COVID-19 captured. 81

Table S31: Number of records (and risk per 10,000 individuals) for post COVID-19 cardiac and thromboembolic complications, across cohorts and databases, stratified by exposure status (BNT162b2 vaccine). 86

Table S32: Number of records (and risk per 10,000 individuals) for post COVID-19 cardiac and thromboembolic complications, across cohorts and databases, stratified by exposure status (BNT162b2 vaccine). Follow-up ends at first vaccine dose after index date. 91

Table S33: Number of records (and risk per 10,000 individuals) for post COVID-19 cardiac and thromboembolic complications, across cohorts and databases, stratified by exposure status (BNT162b2 vaccine). Only first outcome after COVID-19 captured. 96

Table S34: Number of records (and risk per 10,000 individuals) for post COVID-19 cardiac and thromboembolic complications, across cohorts and databases, stratified by vaccine (BNT162b2 - ChAdOx1). 101

Table S35: Number of records (and risk per 10,000 individuals) for post COVID-19 cardiac and thromboembolic complications, across cohorts and databases, stratified by vaccine (BNT162b2 - ChAdOx1). Follow-up ends at first vaccine dose after index date. 106

Table S36: Number of records (and risk per 10,000 individuals) for post COVID-19 cardiac and thromboembolic complications, across cohorts and databases, stratified by vaccine (BNT162b2 - ChAdOx1). Only first outcome after COVID-19 captured. 111

Figure S6: Forest plots for vaccine effect (any COVID-19 vaccine), meta-analysis across cohorts and databases. Follow-up ends at first vaccine dose after index date. Dashed line represents a level of hetereogeneity I2 > 0.4. 116

Figure S7: Forest plots for vaccine effect (any COVID-19 vaccine), meta-analysis across cohorts and databases. Only first outcome after COVID-19 captured. Dashed line represents a level of hetereogeneity I2 > 0.4. 117

Figure S8: Forest plots for vaccine effect (ChAdOx1 vaccine), meta-analysis across cohorts and databases. Dashed line represents a level of hetereogeneity I2 > 0.4. 118

Figure S9: Forest plots for vaccine effect (ChAdOx1 vaccine), meta-analysis across cohorts and databases. Follow-up ends at first vaccine dose after index date. Dashed line represents a level of hetereogeneity I2 > 0.4. 119

Figure S10: Forest plots for vaccine effect (ChAdOx1 vaccine), meta-analysis across cohorts and databases. Only first outcome after COVID-19 captured. Dashed line represents a level of hetereogeneity I2 > 0.4. 120

Figure S11: Forest plots for vaccine effect (BNT162b2 vaccine), meta-analysis across cohorts and databases. Dashed line represents a level of hetereogeneity I2 > 0.4. 121

Figure S12: Forest plots for vaccine effect (BNT162b2 vaccine), meta-analysis across cohorts and databases. Follow-up ends at first vaccine dose after index date. Dashed line represents a level of hetereogeneity I2 > 0.4. 122

Figure S13: Forest plots for vaccine effect (BNT162b2 vaccine), meta-analysis across cohorts and databases. Only first outcome after COVID-19 captured. Dashed line represents a level of hetereogeneity I2 > 0.4. 123

Figure S14: Forest plots for vaccine effect (any COVID-19 vaccine) on preventing venous thromboembolism complications, for each cohort and database (meta-analysis estimates across cohorts in the last panel). Dashed line represents a level of hetereogeneity I2 > 0.4. 124

Figure S15: Forest plots for vaccine effect (any COVID-19 vaccine) on preventing venous thromboembolism complications, for each cohort and database (meta-analysis estimates across cohorts in the last panel).Follow-up ends at first vaccine dose after index date. Dashed line represents a level of hetereogeneity I2 > 0.4. 125

Figure S16: Forest plots for vaccine effect (any COVID-19 vaccine) on preventing venous thromboembolism complications, for each cohort and database (meta-analysis estimates across cohorts in the last panel).Only first outcome after COVID-19 captured. Dashed line represents a level of hetereogeneity I2 > 0.4. 126

Figure S17: Forest plots for vaccine effect (ChAdOx1 vaccine) on preventing venous thromboembolism complications, for each cohort and database (meta-analysis estimates across cohorts in the last panel). Dashed line represents a level of hetereogeneity I2 > 0.4. 127

Figure S18: Forest plots for vaccine effect (ChAdOx1 vaccine) on preventing venous thromboembolism complications, for each cohort and database (meta-analysis estimates across cohorts in the last panel).Follow-up ends at first vaccine dose after index date. Dashed line represents a level of hetereogeneity I2 > 0.4. 128

Figure S19: Forest plots for vaccine effect (ChAdOx1 vaccine) on preventing venous thromboembolism complications, for each cohort and database (meta-analysis estimates across cohorts in the last panel).Only first outcome after COVID-19 captured. Dashed line represents a level of hetereogeneity I2 > 0.4. 129

Figure S20: Forest plots for vaccine effect (BNT162b2 vaccine) on preventing venous thromboembolism complications, for each cohort and database (meta-analysis estimates across cohorts in the last panel). Dashed line represents a level of hetereogeneity I2 > 0.4. 130

Figure S21: Forest plots for vaccine effect (BNT162b2 vaccine) on preventing venous thromboembolism complications, for each cohort and database (meta-analysis estimates across cohorts in the last panel).Follow-up ends at first vaccine dose after index date. Dashed line represents a level of hetereogeneity I2 > 0.4. 131

Figure S22: Forest plots for vaccine effect (BNT162b2 vaccine) on preventing venous thromboembolism complications, for each cohort and database (meta-analysis estimates across cohorts in the last panel).Only first outcome after COVID-19 captured. Dashed line represents a level of hetereogeneity I2 > 0.4. 132

Figure S23: Forest plots for vaccine effect (any COVID-19 vaccine) on preventing arterial thrombosis/thromboembolism complications, for each cohort and database (meta-analysis estimates across cohorts in the last panel). Dashed line represents a level of hetereogeneity I2 > 0.4. 133

Figure S24: Forest plots for vaccine effect (any COVID-19 vaccine) on preventing arterial thrombosis/thromboembolism complications, for each cohort and database (meta-analysis estimates across cohorts in the last panel).Follow-up ends at first vaccine dose after index date. Dashed line represents a level of hetereogeneity I2 > 0.4. 134

Figure S25: Forest plots for vaccine effect (any COVID-19 vaccine) on preventing arterial thrombosis/thromboembolism complications, for each cohort and database (meta-analysis estimates across cohorts in the last panel).Only first outcome after COVID-19 captured. Dashed line represents a level of hetereogeneity I2 > 0.4. 135

Figure S26: Forest plots for vaccine effect (ChAdOx1 vaccine) on preventing arterial thrombosis/thromboembolism complications, for each cohort and database (meta-analysis estimates across cohorts in the last panel). Dashed line represents a level of hetereogeneity I2 > 0.4. 136

Figure S27: Forest plots for vaccine effect (ChAdOx1 vaccine) on preventing arterial thrombosis/thromboembolism complications, for each cohort and database (meta-analysis estimates across cohorts in the last panel).Follow-up ends at first vaccine dose after index date. Dashed line represents a level of hetereogeneity I2 > 0.4. 137

Figure S28: Forest plots for vaccine effect (ChAdOx1 vaccine) on preventing arterial thrombosis/thromboembolism complications, for each cohort and database (meta-analysis estimates across cohorts in the last panel).Only first outcome after COVID-19 captured. Dashed line represents a level of hetereogeneity I2 > 0.4. 138

Figure S29: Forest plots for vaccine effect (BNT162b2 vaccine) on preventing arterial thrombosis/thromboembolism complications, for each cohort and database (meta-analysis estimates across cohorts in the last panel). Dashed line represents a level of hetereogeneity I2 > 0.4. 139

Figure S30: Forest plots for vaccine effect (BNT162b2 vaccine) on preventing arterial thrombosis/thromboembolism complications, for each cohort and database (meta-analysis estimates across cohorts in the last panel).Follow-up ends at first vaccine dose after index date. Dashed line represents a level of hetereogeneity I2 > 0.4. 140

Figure S31: Forest plots for vaccine effect (BNT162b2 vaccine) on preventing arterial thrombosis/thromboembolism complications, for each cohort and database (meta-analysis estimates across cohorts in the last panel).Only first outcome after COVID-19 captured. Dashed line represents a level of hetereogeneity I2 > 0.4. 141

Figure S32: Forest plots for vaccine effect (any COVID-19 vaccine) on preventing cardiac diseases and hemorrhagic stroke, for each cohort and database (meta-analysis estimates across cohorts in the last panel). Dashed line represents a level of hetereogeneity I2 > 0.4. 142

Figure S33: Forest plots for vaccine effect (any COVID-19 vaccine) on preventing cardiac diseases and hemorrhagic stroke, for each cohort and database (meta-analysis estimates across cohorts in the last panel).Follow-up ends at first vaccine dose after index date. Dashed line represents a level of hetereogeneity I2 > 0.4. 143

Figure S34: Forest plots for vaccine effect (any COVID-19 vaccine) on preventing cardiac diseases and hemorrhagic stroke, for each cohort and database (meta-analysis estimates across cohorts in the last panel).Only first outcome after COVID-19 captured. Dashed line represents a level of hetereogeneity I2 > 0.4. 144

Figure S35: Forest plots for vaccine effect (ChAdOx1 vaccine) on preventing cardiac diseases and hemorrhagic stroke, for each cohort and database (meta-analysis estimates across cohorts in the last panel). Dashed line represents a level of hetereogeneity I2 > 0.4. 145

Figure S36: Forest plots for vaccine effect (ChAdOx1 vaccine) on preventing cardiac diseases and hemorrhagic stroke, for each cohort and database (meta-analysis estimates across cohorts in the last panel).Follow-up ends at first vaccine dose after index date. Dashed line represents a level of hetereogeneity I2 > 0.4. 146

Figure S37: Forest plots for vaccine effect (ChAdOx1 vaccine) on preventing cardiac diseases and hemorrhagic stroke, for each cohort and database (meta-analysis estimates across cohorts in the last panel).Only first outcome after COVID-19 captured. Dashed line represents a level of hetereogeneity I2 > 0.4. 147

Figure S38: Forest plots for vaccine effect (BNT162b2 vaccine) on preventing cardiac diseases and hemorrhagic stroke, for each cohort and database (meta-analysis estimates across cohorts in the last panel). Dashed line represents a level of hetereogeneity I2 > 0.4. 148

Figure S39: Forest plots for vaccine effect (BNT162b2 vaccine) on preventing cardiac diseases and hemorrhagic stroke, for each cohort and database (meta-analysis estimates across cohorts in the last panel).Follow-up ends at first vaccine dose after index date. Dashed line represents a level of hetereogeneity I2 > 0.4. 149

Figure S40: Forest plots for vaccine effect (BNT162b2 vaccine) on preventing cardiac diseases and hemorrhagic stroke, for each cohort and database (meta-analysis estimates across cohorts in the last panel).Only first outcome after COVID-19 captured. Dashed line represents a level of hetereogeneity I2 > 0.4. 150

Figure S41: Forest plots for comparative effect, meta-analysis across cohorts and databases. Follow-up ends at first vaccine dose after index date. Dashed line represents a level of hetereogeneity I2 > 0.4. 151

Figure S42: Forest plots for comparative effect, meta-analysis across cohorts and databases. Only first outcome after COVID-19 captured. Dashed line represents a level of hetereogeneity I2 > 0.4. 152

Figure S43: Forest plots for comparative effect on preventing venous thromboembolism complications, for each cohort and database (meta-analysis estimates across cohorts in the last panel). Dashed line represents a level of hetereogeneity I2 > 0.4. 153

Figure S44: Forest plots for comparative effect on preventing venous thromboembolism complications, for each cohort and database (meta-analysis estimates across cohorts in the last panel).Follow-up ends at first vaccine dose after index date. Dashed line represents a level of hetereogeneity I2 > 0.4. 154

Figure S45: Forest plots for comparative effect on preventing venous thromboembolism complications, for each cohort and database (meta-analysis estimates across cohorts in the last panel).Only first outcome after COVID-19 captured. Dashed line represents a level of hetereogeneity I2 > 0.4. 155

Figure S46: Forest plots for comparative effect on preventing arterial thrombosis/thromboembolism complications, for each cohort and database (meta-analysis estimates across cohorts in the last panel). Dashed line represents a level of hetereogeneity I2 > 0.4. 156

Figure S47: Forest plots for comparative effect on preventing arterial thrombosis/thromboembolism complications, for each cohort and database (meta-analysis estimates across cohorts in the last panel).Follow-up ends at first vaccine dose after index date. Dashed line represents a level of hetereogeneity I2 > 0.4. 157

Figure S48: Forest plots for comparative effect on preventing arterial thrombosis/thromboembolism complications, for each cohort and database (meta-analysis estimates across cohorts in the last panel).Only first outcome after COVID-19 captured. Dashed line represents a level of hetereogeneity I2 > 0.4. 158

Figure S49: Forest plots for comparative effect on preventing cardiac diseases and hemorrhagic stroke, for each cohort and database (meta-analysis estimates across cohorts in the last panel). Dashed line represents a level of hetereogeneity I2 > 0.4. 159

Figure S50: Forest plots for comparative effect on preventing cardiac diseases and hemorrhagic stroke, for each cohort and database (meta-analysis estimates across cohorts in the last panel).Follow-up ends at first vaccine dose after index date. Dashed line represents a level of hetereogeneity I2 > 0.4. 160

Figure S51: Forest plots for comparative effect on preventing cardiac diseases and hemorrhagic stroke, for each cohort and database (meta-analysis estimates across cohorts in the last panel).Only first outcome after COVID-19 captured. Dashed line represents a level of hetereogeneity I2 > 0.4. 161

Figure S1: Follow-up in vaccinated and unvaccinated cohorts.

(A) Main analysis, (B) Follow-up ends at first vaccine dose after index date, (C) Post COVID sequelae refers just to the first outcome after infection.

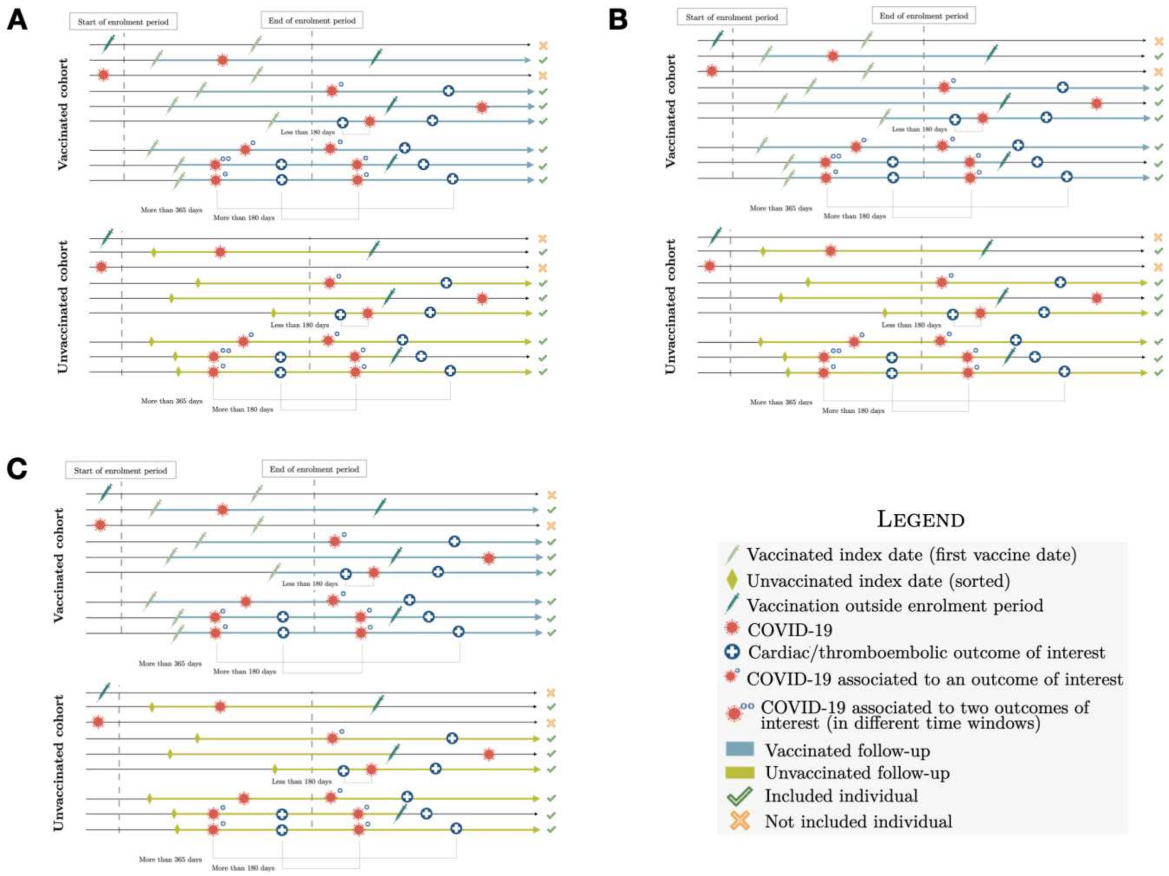

Mercadé-Besora N, *et al. Heart* 2024; 110:635–643. doi: 10.1136/heartjnl-2023-323483

Figure S2: Study Inclusion Flowchart CPRD AURUM

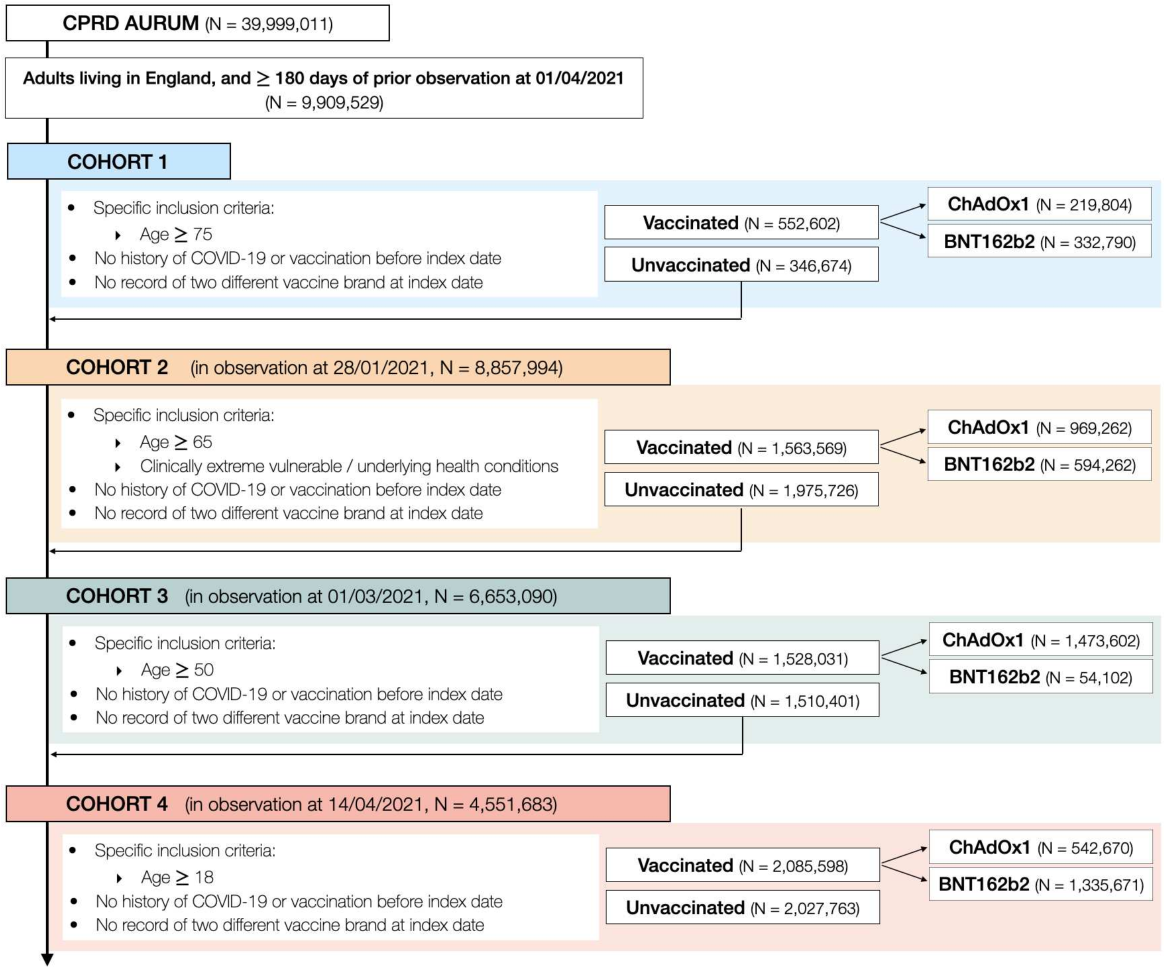

Figure S3: Study Inclusion Flowchart CPRD GOLD.

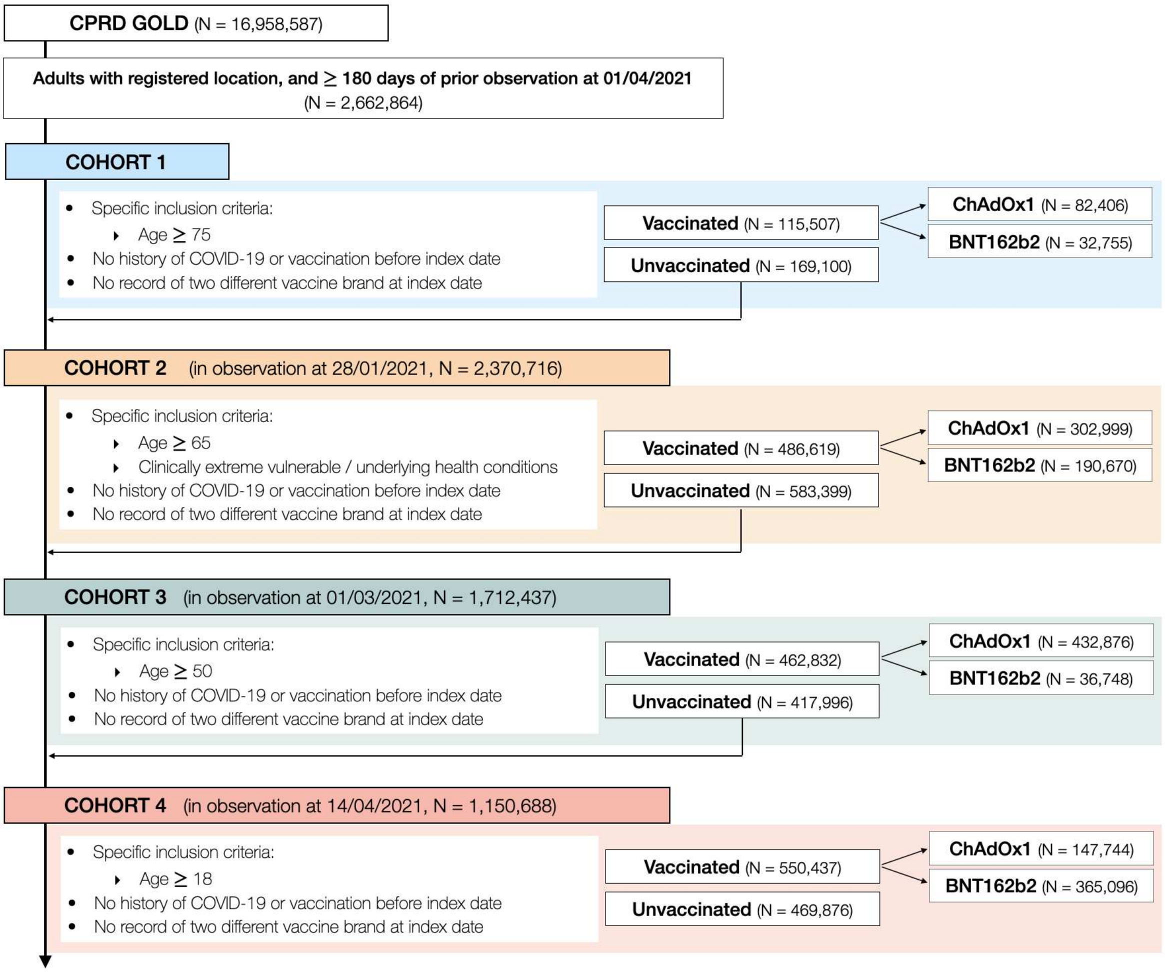

Mercadé-Besora N, *et al. Heart* 2024; 110:635–643. doi: 10.1136/heartjnl-2023-323483

Figure S4: Study Inclusion Flowchart SIDIAP.

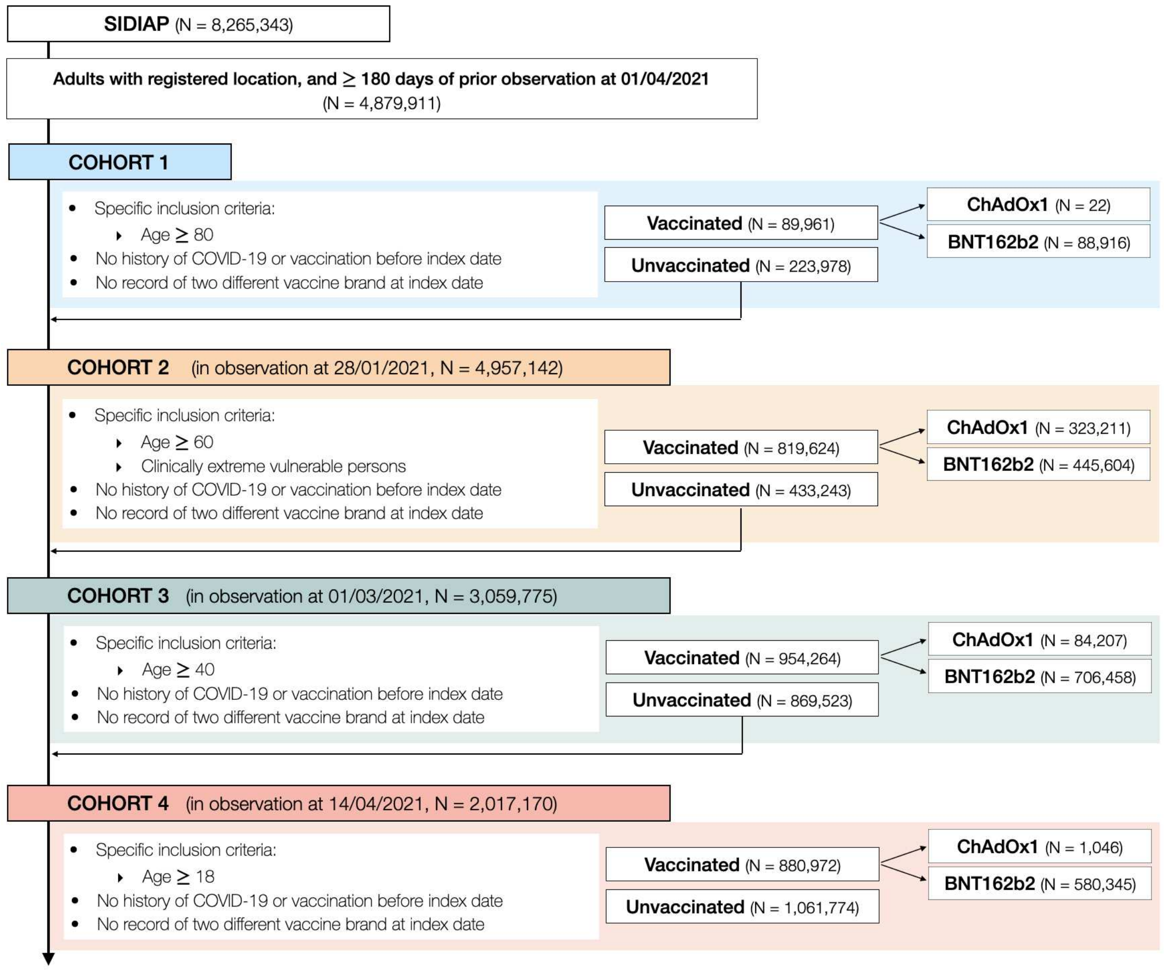

Mercadé-Besora N, *et al. Heart* 2024; 110:635–643. doi: 10.1136/heartjnl-2023-323483

Figure S5: Study Inclusion Flowchart CORIVA.

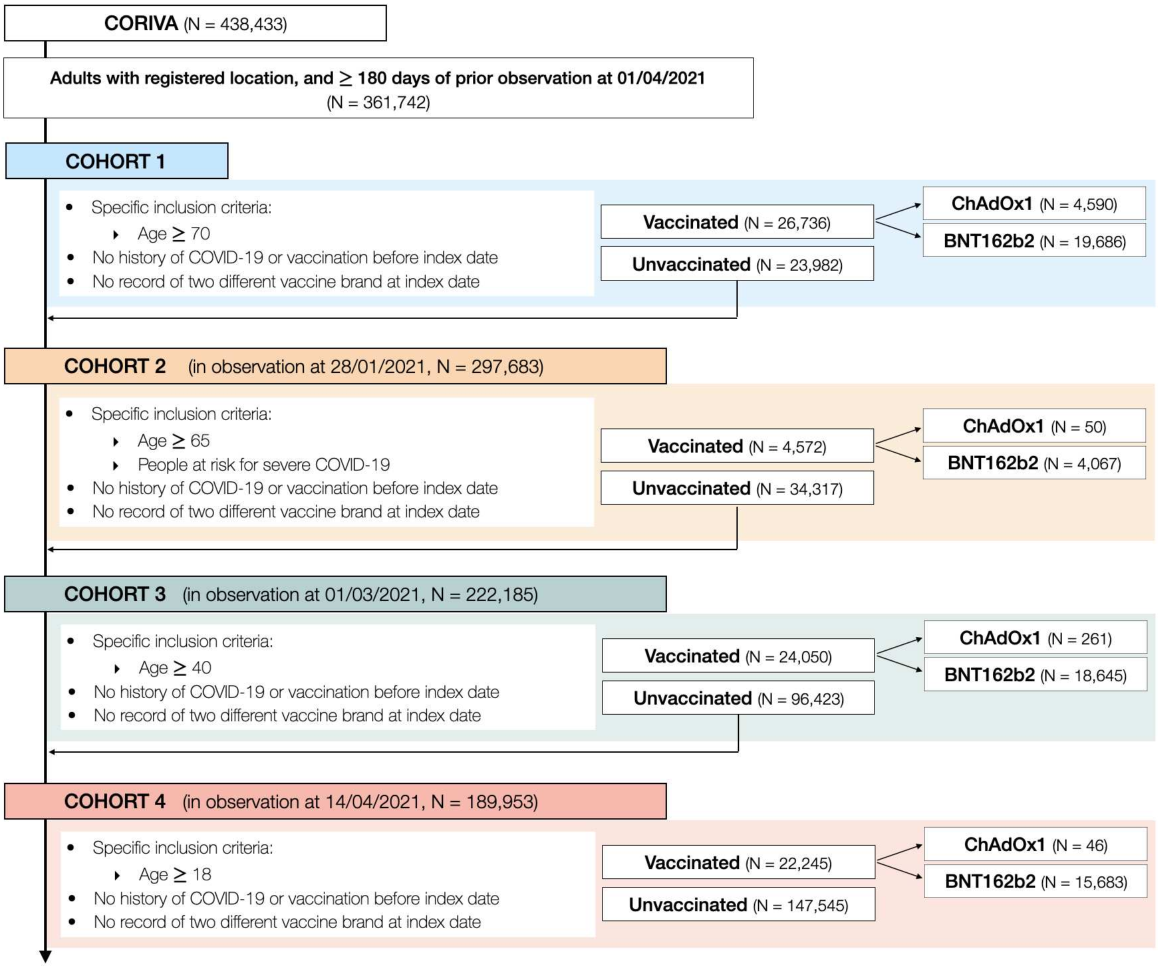

Table S1: Characteristics of unweighted populations in CPRD AURUM, database, stratified by staggered cohort and exposure status. Exposure is any COVID-19 vaccine.

	Cohort 1			Cohort 2			Cohort 3			Cohort 4		
	**Unvaccinated**	**Vaccinated**	**ASMD**	**Unvaccinated**	**Vaccinated**	**ASMD**	**Unvaccinated**	**Vaccinated**	**ASMD**	**Unvaccinated**	**Vaccinated**	**ASMD**
**N (individuals)**	346,674	552,602		1,975,726	1,563,569		1,510,401	1,528,031		2,027,763	2,085,598	
**Age, median [Q25-Q75]**	78 [76-82]	81 [78-86]	0.319	42 [32-55]	68 [60-73]	0.995	41 [31-53]	55 [51-60]	0.610	35 [27-45]	35 [27-43]	0.134
**Sex: Female, N(%)**	192,771 (56%)	314,141 (57%)	0.025	1,244,636 (63%)	852,976 (55%)	0.172	880,404 (58%)	771,929 (51%)	0.157	862,676 (43%)	958,124 (46%)	0.068
**Years of prior history*, median [Q25-Q75]**	24 [10-35]	25 [11-37]	0.056	15 [7-25]	21 [9-32]	0.278	13 [6-22]	17 [8-27]	0.207	8 [4-16]	8 [3-18]	0.032
**Number of GP visits, median [Q25-Q75]**	10 [5-17]	11 [6-19]	0.105	6 [2-11]	10 [6-17]	0.367	4 [1-10]	7 [3-12]	0.180	2 [0-5]	3 [1-7]	0.108
**Number of PCR tests, median [Q25-Q75]**	0[0-0]	0[0-0]	0.103	0[0-0]	0[0-0]	0.065	0[0-0]	0[0-0]	0.007	0[0-0]	0[0-0]	0.078
**Comorbidities**, N(%)**												
Anxiety	51,067 (15%)	82,765 (15%)	0.007	435,770 (22%)	309,567 (20%)	0.056	289,563 (19%)	292,336 (19%)	0.001	265,627 (13%)	338,058 (16%)	0.088
Asthma	36,474 (11%)	61,899 (11%)	0.022	501,209 (25%)	275,768 (18%)	0.189	294,123 (19%)	209,708 (14%)	0.155	138,317 (7%)	173,056 (8%)	0.056
Chronic kidney disease	71,083 (21%)	137,953 (25%)	0.107	55,104 (3%)	154,263 (10%)	0.294	24,340 (2%)	30,896 (2%)	0.031	18,625 (1%)	5,796 (0%)	0.083
COPD	28,665 (8%)	47,422 (9%)	0.011	29,729 (2%)	107,297 (7%)	0.270	13,433 (1%)	15,685 (1%)	0.014	10,543 (1%)	2,535 (0%)	0.071
Dementia	16,610 (5%)	32,934 (6%)	0.052	6,692 (0%)	15,828 (1%)	0.082	3,425 (0%)	3,219 (0%)	0.003	2,691 (0%)	575 (0%)	0.037
Depressive disorder	42,145 (12%)	64,840 (12%)	0.013	367,463 (19%)	283,390 (18%)	0.012	236,584 (16%)	265,876 (17%)	0.047	210,644 (10%)	253,317 (12%)	0.056
Diabetes	63,154 (18%)	101,682 (18%)	0.005	107,425 (5%)	253,026 (16%)	0.351	60,932 (4%)	84,224 (6%)	0.069	49,154 (2%)	22,968 (1%)	0.101
GERD	18,336 (5%)	32,360 (6%)	0.025	68,070 (3%)	84,985 (5%)	0.097	39,594 (3%)	58,961 (4%)	0.070	31,119 (2%)	38,612 (2%)	0.025
Heart failure	17,882 (5%)	33,578 (6%)	0.040	12,275 (1%)	37,396 (2%)	0.146	5,641 (0%)	7,204 (0%)	0.015	4,611 (0%)	1,163 (0%)	0.046
Hypertension	172,799 (50%)	301,294 (55%)	0.094	206,105 (10%)	545,782 (35%)	0.611	110,912 (7%)	240,120 (16%)	0.264	86,435 (4%)	54,357 (3%)	0.091
Hypothyroidism	31,444 (9%)	56,367 (10%)	0.038	78,934 (4%)	118,453 (8%)	0.154	43,892 (3%)	67,686 (4%)	0.081	30,993 (2%)	35,364 (2%)	0.013
Malignant neoplastic	68,984 (20%)	131,635	0.095	60,318 (3%)	207,240	0.379	29,689 (2%)	61,923 (4%)	0.122	22,701 (1%)	13,590 (1%)	0.050

Mercadé-Besora N, *et al. Heart* 2024; 110:635–643. doi: 10.1136/heartjnl-2023-323483

	Cohort 1			Cohort 2			Cohort 3			Cohort 4		
	**Unvaccinated**	**Vaccinated**	**ASMD**	**Unvaccinated**	**Vaccinated**	**ASMD**	**Unvaccinated**	**Vaccinated**	**ASMD**	**Unvaccinated**	**Vaccinated**	**ASMD**
disease		(24%)			(13%)							
Myocardial infarction	16,027 (5%)	29,525 (5%)	0.033	20,259 (1%)	56,161 (4%)	0.172	7,396 (0%)	12,703 (1%)	0.042	5,764 (0%)	1,621 (0%)	0.049
Osteoporosis	29,909 (9%)	59,521 (11%)	0.072	18,263 (1%)	64,108 (4%)	0.204	8,846 (1%)	15,089 (1%)	0.045	6,922 (0%)	2,535 (0%)	0.046
Pneumonia	16,992 (5%)	28,398 (5%)	0.011	31,746 (2%)	50,739 (3%)	0.107	17,785 (1%)	22,064 (1%)	0.023	13,305 (1%)	13,282 (1%)	0.002
Rheumatoid arthritis	6,596 (2%)	11,237 (2%)	0.009	14,811 (1%)	33,173 (2%)	0.116	5,548 (0%)	9,130 (1%)	0.033	3,710 (0%)	1,816 (0%)	0.026
Stroke	15,249 (4%)	26,478 (5%)	0.019	16,942 (1%)	39,907 (3%)	0.131	7,422 (0%)	10,828 (1%)	0.028	6,030 (0%)	2,291 (0%)	0.042
Venous thromboembolism	19,607 (6%)	35,332 (6%)	0.031	28,876 (1%)	63,048 (4%)	0.158	13,608 (1%)	26,299 (2%)	0.072	12,945 (1%)	4,787 (0%)	0.062

The 4 cohorts represent vaccine rollout periods. *Calculated as the days of previous observation in the database before index date. **Assessed anytime before index date. ASMD = Absolute standardized mean difference, GP = General practice, PCR = Polymerase chain reaction, COPD = Chronic obstructive pulmonary disease, GERD = Gastro-Esophageal reflux disease

Mercadé-Besora N, *et al. Heart* 2024; 110:635–643. doi: 10.1136/heartjnl-2023-323483

Table S2: Characteristics of weighted populations in CPRD AURUM, database, stratified by staggered cohort and exposure status. Exposure is ChAdOx1 vaccine.

	Cohort 1			Cohort 2			Cohort 3			Cohort 4		
	**Unvaccinated**	**Vaccinated**	**ASMD**	**Unvaccinated**	**Vaccinated**	**ASMD**	**Unvaccinated**	**Vaccinated**	**ASMD**	**Unvaccinated**	**Vaccinated**	**ASMD**
**N (individuals)**	100,517	99,985		308,608	304,995		448,766	445,397		242,955	243,847	
**Age, median [Q25-Q75]**	78 [76-84]	78 [76-84]	0.001	62 [47-69]	62 [47-69]	0.007	50 [41-57]	52 [41-57]	0.001	44 [41-48]	44 [41-48]	0.003
**Sex: Female, N(%)**	57,756 (57%)	57,174 (57%)	0.006	179,574 (58%)	176,871 (58%)	0.004	236,260 (53%)	234,320 (53%)	0.001	101,805 (42%)	100,846 (41%)	0.011
**Years of prior history*, median [Q25-Q75]**	24 [10-36]	24 [10-36]	0.004	18 [8-29]	18 [8-29]	0.002	14 [6-24]	14 [6-24]	0.002	10 [5-18]	9 [5-18]	0.004
**Number of GP visits, median [Q25-Q75]**	10 [5-18]	10 [6-18]	0.003	8 [3-15]	8 [5-15]	0.003	4 [1-11]	6 [2-10]	0.007	2 [0-7]	2 [1-7]	0.016
**Number of PCR tests, median [Q25-Q75]**	0[0-0]	0[0-0]	0.011	0[0-0]	0[0-0]	0.003	0[0-0]	0[0-0]	0.003	0[0-0]	0[0-0]	0.005
**Comorbidities**, N(%)**												
Anxiety	15,457 (15%)	15,069 (15%)	0.009	67,125 (22%)	64,408 (21%)	0.015	90,806 (20%)	87,243 (20%)	0.016	37,475 (15%)	38,040 (16%)	0.005
Asthma	10,993 (11%)	10,863 (11%)	0.002	65,274 (21%)	62,424 (20%)	0.017	76,417 (17%)	75,841 (17%)	0.000	17,944 (7%)	17,468 (7%)	0.009
Chronic kidney disease	23,223 (23%)	22,872 (23%)	0.005	23,022 (7%)	24,288 (8%)	0.019	8,460 (2%)	8,356 (2%)	0.001	2,419 (1%)	2,043 (1%)	0.017
COPD	8,669 (9%)	8,770 (9%)	0.005	14,411 (5%)	15,226 (5%)	0.015	5,747 (1%)	4,794 (1%)	0.019	1,263 (1%)	1,250 (1%)	0.001
Dementia	6,529 (6%)	6,431 (6%)	0.003	3,880 (1%)	3,541 (1%)	0.009	898 (0%)	1,004 (0%)	0.006	203 (0%)	399 (0%)	0.023
Depressive disorder	12,559 (12%)	12,267 (12%)	0.007	60,745 (20%)	57,903 (19%)	0.018	80,264 (18%)	77,017 (17%)	0.016	32,304 (13%)	33,302 (14%)	0.011
Diabetes	18,750 (19%)	18,602 (19%)	0.001	39,262 (13%)	38,979 (13%)	0.002	25,334 (6%)	26,121 (6%)	0.009	6,679 (3%)	6,315 (3%)	0.010
GERD	5,590 (6%)	5,471 (5%)	0.004	14,645 (5%)	14,339 (5%)	0.002	14,758 (3%)	14,823 (3%)	0.002	5,727 (2%)	6,021 (2%)	0.007
Heart failure	6,181 (6%)	5,763 (6%)	0.016	6,098 (2%)	5,685 (2%)	0.008	2,241 (0%)	1,983 (0%)	0.008	610 (0%)	545 (0%)	0.006
Hypertension	52,737 (52%)	51,796 (52%)	0.013	78,623 (25%)	79,827 (26%)	0.016	51,054 (11%)	51,059 (11%)	0.003	15,350 (6%)	15,670 (6%)	0.004
Hypothyroidism	9,956 (10%)	9,690 (10%)	0.007	19,720 (6%)	19,562 (6%)	0.001	16,480 (4%)	16,361 (4%)	0.000	5,735 (2%)	5,984 (2%)	0.006
Malignant neoplastic	21,691 (22%)	21,468	0.003	25,191 (8%)	27,892 (9%)	0.035	13,140 (3%)	12,870 (3%)	0.002	4,003 (2%)	3,621 (1%)	0.013

13

	Cohort 1			Cohort 2			Cohort 3			Cohort 4		
	**Unvaccinated**	**Vaccinated**	**ASMD**	**Unvaccinated**	**Vaccinated**	**ASMD**	**Unvaccinated**	**Vaccinated**	**ASMD**	**Unvaccinated**	**Vaccinated**	**ASMD**
disease		(21%)										
Myocardial infarction	5,182 (5%)	4,988 (5%)	0.008	7,777 (3%)	8,594 (3%)	0.018	3,415 (1%)	3,228 (1%)	0.004	933 (0%)	636 (0%)	0.022
Osteoporosis	9,975 (10%)	10,018 (10%)	0.003	9,052 (3%)	9,423 (3%)	0.009	3,285 (1%)	3,499 (1%)	0.006	840 (0%)	934 (0%)	0.006
Pneumonia	5,770 (6%)	5,268 (5%)	0.021	9,096 (3%)	8,541 (3%)	0.009	6,196 (1%)	6,077 (1%)	0.001	1,923 (1%)	2,040 (1%)	0.005
Rheumatoid arthritis	2,002 (2%)	2,063 (2%)	0.005	4,856 (2%)	4,971 (2%)	0.004	2,266 (1%)	2,795 (1%)	0.016	665 (0%)	478 (0%)	0.016
Stroke	5,085 (5%)	4,791 (5%)	0.012	6,650 (2%)	6,616 (2%)	0.001	3,150 (1%)	2,942 (1%)	0.005	908 (0%)	773 (0%)	0.010
Venous thromboembolism	6,342 (6%)	6,069 (6%)	0.010	9,208 (3%)	9,850 (3%)	0.014	6,193 (1%)	7,393 (2%)	0.023	2,532 (1%)	1,239 (1%)	0.061

The 4 cohorts represent vaccine rollout periods. *Calculated as the days of previous observation in the database before index date. **Assessed anytime before index date. ASMD = Absolute standardized mean difference, GP = General practice, PCR = Polymerase chain reaction, COPD = Chronic obstructive pulmonary disease, GERD = Gastro-Esophageal reflux disease

Mercadé-Besora N, *et al. Heart* 2024; 110:635–643. doi: 10.1136/heartjnl-2023-323483

Table S3: Characteristics of unweighted populations in CPRD AURUM, database, stratified by staggered cohort and exposure status. Exposure is ChAdOx1 vaccine.

	Cohort 1			Cohort 2			Cohort 3			Cohort 4		
	**Unvaccinated**	**Vaccinated**	**ASMD**	**Unvaccinated**	**Vaccinated**	**ASMD**	**Unvaccinated**	**Vaccinated**	**ASMD**	**Unvaccinated**	**Vaccinated**	**ASMD**
**N (individuals)**	344,687	219,804		1,975,770	969,262		1,510,493	1,473,602		2,066,318	542,670	
**Age, median [Q25-Q75]**	78 [76-82]	80 [77-85]	0.240	42 [32-55]	69 [63-73]	1.059	41 [31-53]	55 [51-60]	0.605	34 [27-45]	45 [42-48]	0.507
**Sex: Female, N(%)**	191,649 (56%)	127,656 (58%)	0.050	1,244,532 (63%)	528,692 (55%)	0.172	880,468 (58%)	739,444 (50%)	0.163	880,225 (43%)	242,758 (45%)	0.043
**Years of prior history*, median [Q25-Q75]**	24 [10-35]	24 [9-36]	0.018	15 [7-25]	21 [9-33]	0.288	13 [6-22]	17 [8-27]	0.203	7 [4-16]	10 [5-18]	0.143
**Number of GP visits, median [Q25-Q75]**	10 [5-17]	12 [6-19]	0.121	6 [2-11]	10 [6-17]	0.359	4 [1-10]	7 [3-12]	0.169	2 [0-5]	3 [1-8]	0.146
**Number of PCR tests, median [Q25-Q75]**	0[0-0]	0[0-0]	0.194	0[0-0]	0[0-0]	0.070	0[0-0]	0[0-0]	0.005	0[0-0]	0[0-0]	0.066
**Comorbidities**, N(%)**												
Anxiety	50,786 (15%)	33,355 (15%)	0.012	435,737 (22%)	187,469 (19%)	0.067	289,618 (19%)	281,846 (19%)	0.001	268,451 (13%)	86,805 (16%)	0.085
Asthma	36,259 (11%)	24,009 (11%)	0.013	501,234 (25%)	159,162 (16%)	0.221	294,156 (19%)	200,995 (14%)	0.157	140,178 (7%)	41,393 (8%)	0.033
Chronic kidney disease	70,604 (20%)	51,053 (23%)	0.066	55,103 (3%)	97,339 (10%)	0.299	24,361 (2%)	24,198 (2%)	0.002	18,798 (1%)	2,879 (1%)	0.045
COPD	28,448 (8%)	19,105 (9%)	0.016	29,730 (2%)	67,700 (7%)	0.274	13,436 (1%)	13,621 (1%)	0.004	10,647 (1%)	1,510 (0%)	0.038
Dementia	16,327 (5%)	16,795 (8%)	0.121	6,689 (0%)	12,106 (1%)	0.103	3,424 (0%)	1,896 (0%)	0.023	2,729 (0%)	447 (0%)	0.015
Depressive disorder	41,905 (12%)	27,009 (12%)	0.004	367,500 (19%)	170,626 (18%)	0.026	236,628 (16%)	256,789 (17%)	0.047	212,626 (10%)	74,210 (14%)	0.104
Diabetes	62,733 (18%)	39,751 (18%)	0.003	107,449 (5%)	154,348 (16%)	0.344	60,968 (4%)	78,394 (5%)	0.061	49,223 (2%)	9,729 (2%)	0.041
GERD	18,253 (5%)	12,156 (6%)	0.010	68,089 (3%)	52,694 (5%)	0.097	39,619 (3%)	56,444 (4%)	0.068	31,367 (2%)	13,807 (3%)	0.073
Heart failure	17,716 (5%)	13,147 (6%)	0.037	12,277 (1%)	24,276 (3%)	0.152	5,638 (0%)	5,673 (0%)	0.002	4,625 (0%)	681 (0%)	0.024
Hypertension	171,846 (50%)	114,922 (52%)	0.049	206,105 (10%)	345,014 (36%)	0.626	110,975 (7%)	224,543 (15%)	0.251	87,010 (4%)	31,330 (6%)	0.072
Hypothyroidism	31,256 (9%)	21,855 (10%)	0.030	78,921 (4%)	73,992 (8%)	0.156	43,887 (3%)	64,026 (4%)	0.077	31,315 (2%)	13,920 (3%)	0.074
Malignant neoplastic disease	68,598 (20%)	49,023 (22%)	0.059	60,355 (3%)	131,135 (14%)	0.387	29,693 (2%)	55,601 (4%)	0.108	22,873 (1%)	7,505 (1%)	0.025

Mercadé-Besora N, *et al. Heart* 2024; 110:635–643. doi: 10.1136/heartjnl-2023-323483

	Cohort 1			Cohort 2			Cohort 3			Cohort 4		
	**Unvaccinated**	**Vaccinated**	**ASMD**	**Unvaccinated**	**Vaccinated**	**ASMD**	**Unvaccinated**	**Vaccinated**	**ASMD**	**Unvaccinated**	**Vaccinated**	**ASMD**
Myocardial infarction	15,938 (5%)	11,236 (5%)	0.023	20,262 (1%)	33,142 (3%)	0.163	7,401 (0%)	11,237 (1%)	0.035	5,781 (0%)	861 (0%)	0.026
Osteoporosis	29,704 (9%)	22,986 (10%)	0.063	18,255 (1%)	42,319 (4%)	0.216	8,856 (1%)	12,455 (1%)	0.031	6,980 (0%)	1,380 (0%)	0.015
Pneumonia	16,837 (5%)	12,101 (6%)	0.028	31,706 (2%)	33,170 (3%)	0.116	17,787 (1%)	20,399 (1%)	0.018	13,425 (1%)	4,297 (1%)	0.017
Rheumatoid arthritis	6,549 (2%)	4,544 (2%)	0.012	14,813 (1%)	20,294 (2%)	0.114	5,536 (0%)	8,463 (1%)	0.030	3,723 (0%)	821 (0%)	0.007
Stroke	15,118 (4%)	10,876 (5%)	0.027	16,944 (1%)	24,851 (3%)	0.132	7,424 (0%)	9,547 (1%)	0.021	6,059 (0%)	1,153 (0%)	0.016
Venous thromboembolism	19,455 (6%)	13,782 (6%)	0.026	28,887 (1%)	39,415 (4%)	0.159	13,609 (1%)	24,312 (2%)	0.067	13,021 (1%)	1,936 (0%)	0.039

The 4 cohorts represent vaccine rollout periods. *Calculated as the days of previous observation in the database before index date. **Assessed anytime before index date. ASMD = Absolute standardized mean difference, GP = General practice, PCR = Polymerase chain reaction, COPD = Chronic obstructive pulmonary disease, GERD = Gastro-Esophageal reflux disease

Mercadé-Besora N, *et al. Heart* 2024; 110:635–643. doi: 10.1136/heartjnl-2023-323483

Table S4: Characteristics of weighted populations in CPRD AURUM, database, stratified by staggered cohort and exposure status. Exposure is BNT162b2 vaccine.

	Cohort 1			Cohort 2			Cohort 3			Cohort 4		
	Unvaccinated	Vaccinated	ASMD	Unvaccinated	Vaccinated	ASMD	Unvaccinated	Vaccinated	ASMD	Unvaccinated	Vaccinated	ASMD
**N (individuals)**	105,853	106,684		255,400	253,164		37,236	37,227		630,449	633,628	
**Age, median [Q25-Q75]**	80 [77-85]	80 [77-85]	0.003	58 [46-67]	58 [46-68]	0.006	54 [45-64]	54 [45-64]	0.016	32 [25-37]	32 [25-37]	0.001
**Sex: Female, N(%)**	60,612 (57%)	60,891 (57%)	0.004	147,020 (58%)	146,572 (58%)	0.007	22,049 (59%)	22,144 (59%)	0.005	278,649 (44%)	282,744 (45%)	0.009
**Years of prior history*, median [Q25-Q75]**	24 [11-36]	24 [11-35]	0.005	18 [8-29]	18 [8-29]	0.007	16 [7-27]	17 [7-27]	0.007	7 [4-16]	7 [3-18]	0.006
**Number of GP visits, median [Q25-Q75]**	10 [5-18]	10 [6-17]	0.002	8 [3-15]	10 [5-15]	0.013	6 [2-15]	8 [4-14]	0.003	2 [0-6]	2 [0-6]	0.011
**Number of PCR tests, median [Q25-Q75]**	0[0-0]	0[0-0]	0.001	0[0-0]	0[0-0]	0.003	0[0-0]	0[0-0]	0.000	0[0-0]	0[0-0]	0.010
**Comorbidities**, N(%)**												
Anxiety	15,706 (15%)	15,578 (15%)	0.007	57,951 (23%)	55,594 (22%)	0.018	7,672 (21%)	7,363 (20%)	0.021	96,918 (15%)	96,528 (15%)	0.004
Asthma	11,563 (11%)	11,733 (11%)	0.002	60,265 (24%)	58,010 (23%)	0.016	6,397 (17%)	6,215 (17%)	0.013	50,849 (8%)	48,590 (8%)	0.015
Chronic kidney disease	25,741 (24%)	26,243 (25%)	0.007	17,219 (7%)	18,864 (7%)	0.028	2,790 (7%)	2,621 (7%)	0.017	2,035 (0%)	1,846 (0%)	0.006
COPD	9,240 (9%)	8,999 (8%)	0.011	11,625 (5%)	12,410 (5%)	0.017	1,224 (3%)	886 (2%)	0.055	859 (0%)	807 (0%)	0.002
Dementia	6,683 (6%)	5,727 (5%)	0.040	2,225 (1%)	1,309 (1%)	0.043	618 (2%)	529 (1%)	0.019	112 (0%)	107 (0%)	0.001
Depressive disorder	12,529 (12%)	12,450 (12%)	0.005	52,883 (21%)	50,765 (20%)	0.016	6,588 (18%)	6,431 (17%)	0.011	71,832 (11%)	71,139 (11%)	0.005
Diabetes	20,227 (19%)	20,182 (19%)	0.005	32,557 (13%)	32,989 (13%)	0.008	3,153 (8%)	3,212 (9%)	0.006	8,183 (1%)	7,675 (1%)	0.008
GERD	6,148 (6%)	5,937 (6%)	0.011	12,436 (5%)	12,160 (5%)	0.003	1,552 (4%)	1,509 (4%)	0.006	9,586 (2%)	10,173 (2%)	0.007
Heart failure	6,643 (6%)	6,329 (6%)	0.014	4,618 (2%)	3,987 (2%)	0.018	767 (2%)	552 (1%)	0.044	474 (0%)	344 (0%)	0.008
Hypertension	56,732 (54%)	57,023 (53%)	0.003	62,628 (25%)	63,500 (25%)	0.013	7,802 (21%)	7,697 (21%)	0.007	11,603 (2%)	12,098 (2%)	0.005
Hypothyroidism	10,528 (10%)	10,633 (10%)	0.001	16,084 (6%)	16,368 (6%)	0.007	2,046 (5%)	2,032 (5%)	0.002	8,722 (1%)	8,355 (1%)	0.006
Malignant neoplastic disease	23,367 (22%)	23,618 (22%)	0.002	19,432 (8%)	22,945 (9%)	0.053	2,799 (8%)	2,742 (7%)	0.006	3,432 (1%)	2,913 (0%)	0.012

Mercadé-Besora N, *et al. Heart* 2024; 110:635–643. doi: 10.1136/heartjnl-2023-323483

	Cohort 1			Cohort 2			Cohort 3			Cohort 4		
	Unvaccinated	Vaccinated	ASMD	Unvaccinated	Vaccinated	ASMD	Unvaccinated	Vaccinated	ASMD	Unvaccinated	Vaccinated	ASMD
Myocardial infarction	5,451 (5%)	5,473 (5%)	0.001	6,811 (3%)	7,856 (3%)	0.026	704 (2%)	637 (2%)	0.013	607 (0%)	569 (0%)	0.002
Osteoporosis	10,923 (10%)	10,997 (10%)	0.000	6,664 (3%)	6,208 (2%)	0.010	1,166 (3%)	1,014 (3%)	0.024	653 (0%)	702 (0%)	0.002
Pneumonia	5,814 (5%)	5,303 (5%)	0.023	7,059 (3%)	6,337 (3%)	0.016	991 (3%)	831 (2%)	0.028	3,698 (1%)	3,579 (1%)	0.003
Rheumatoid arthritis	2,115 (2%)	2,076 (2%)	0.004	4,061 (2%)	4,486 (2%)	0.014	330 (1%)	382 (1%)	0.014	723 (0%)	523 (0%)	0.010
Stroke	5,304 (5%)	4,911 (5%)	0.019	5,139 (2%)	5,592 (2%)	0.014	657 (2%)	560 (2%)	0.021	828 (0%)	669 (0%)	0.007
Venous thromboembolism	6,564 (6%)	6,603 (6%)	0.000	7,760 (3%)	7,984 (3%)	0.007	980 (3%)	965 (3%)	0.003	2,860 (0%)	1,602 (0%)	0.034

The 4 cohorts represent vaccine rollout periods. *Calculated as the days of previous observation in the database before index date. **Assessed anytime before index date. ASMD = Absolute standardized mean difference, GP = General practice, PCR = Polymerase chain reaction, COPD = Chronic obstructive pulmonary disease, GERD = Gastro-Esophageal reflux disease

Mercadé-Besora N, *et al. Heart* 2024; 110:635–643. doi: 10.1136/heartjnl-2023-323483

Table S5: Characteristics of unweighted populations in CPRD AURUM, database, stratified by staggered cohort and exposure status. Exposure is BNT162b2 vaccine.

	Cohort 1			Cohort 2			Cohort 3			Cohort 4		
	**Unvaccinated**	**Vaccinated**	**ASMD**	**Unvaccinated**	**Vaccinated**	**ASMD**	**Unvaccinated**	**Vaccinated**	**ASMD**	**Unvaccinated**	**Vaccinated**	**ASMD**
**N (individuals)**	348,052	332,790		1,976,163	594,262		1,510,323	54,102		2,014,161	1,335,671	
**Age, median [Q25-Q75]**	78 [76-82]	82 [79-86]	0.375	42 [32-55]	67 [56-73]	0.899	41 [31-53]	58 [50-82]	0.777	35 [27-45]	31 [25-37]	0.377
**Sex: Female, N(%)**	193,520 (56%)	186,481 (56%)	0.009	1,244,798 (63%)	324,259 (55%)	0.172	880,418 (58%)	32,310 (60%)	0.029	856,596 (43%)	625,195 (47%)	0.086
**Years of prior history*, median [Q25-Q75]**	23 [10-35]	26 [13-38]	0.081	15 [7-25]	21 [9-32]	0.261	13 [6-22]	19 [8-30]	0.308	8 [4-16]	7 [3-19]	0.012
**Number of GP visits, median [Q25-Q75]**	10 [5-17]	11 [6-18]	0.094	6 [2-11]	11 [6-17]	0.381	4 [1-10]	10 [5-17]	0.436	2 [0-5]	3 [1-7]	0.103
**Number of PCR tests, median [Q25-Q75]**	0[0-0]	0[0-0]	0.013	0[0-0]	0[0-0]	0.056	0[0-0]	0[0-0]	0.165	0[0-0]	0[0-0]	0.082
**Comorbidities**, N(%)**												
Anxiety	51,245 (15%)	49,410 (15%)	0.003	435,705 (22%)	122,087 (21%)	0.037	289,623 (19%)	10,441 (19%)	0.003	264,577 (13%)	218,144 (16%)	0.090
Asthma	36,625 (11%)	37,889 (11%)	0.028	501,289 (25%)	116,596 (20%)	0.138	294,101 (19%)	8,668 (16%)	0.090	137,548 (7%)	114,378 (9%)	0.065
Chronic kidney disease	71,413 (21%)	86,899 (26%)	0.133	55,136 (3%)	56,923 (10%)	0.285	24,372 (2%)	6,692 (12%)	0.431	18,579 (1%)	2,664 (0%)	0.097
COPD	28,829 (8%)	28,317 (9%)	0.008	29,793 (2%)	39,595 (7%)	0.263	13,430 (1%)	2,063 (4%)	0.194	10,501 (1%)	942 (0%)	0.083
Dementia	16,828 (5%)	16,138 (5%)	0.001	6,731 (0%)	3,720 (1%)	0.041	3,424 (0%)	1,323 (2%)	0.194	2,673 (0%)	119 (0%)	0.047
Depressive disorder	42,297 (12%)	37,831 (11%)	0.024	367,534 (19%)	112,753 (19%)	0.010	236,577 (16%)	9,046 (17%)	0.029	209,905 (10%)	155,037 (12%)	0.038
Diabetes	63,483 (18%)	61,929 (19%)	0.010	107,471 (5%)	98,676 (17%)	0.362	60,945 (4%)	5,817 (11%)	0.259	49,143 (2%)	12,040 (1%)	0.120
GERD	18,404 (5%)	20,204 (6%)	0.034	68,110 (3%)	32,288 (5%)	0.097	39,607 (3%)	2,506 (5%)	0.108	31,062 (2%)	21,599 (2%)	0.006
Heart failure	17,980 (5%)	20,430 (6%)	0.042	12,311 (1%)	13,120 (2%)	0.134	5,627 (0%)	1,529 (3%)	0.197	4,614 (0%)	448 (0%)	0.054
Hypertension	173,422 (50%)	186,367 (56%)	0.124	206,136 (10%)	200,762 (34%)	0.586	110,969 (7%)	15,536 (29%)	0.579	86,231 (4%)	19,649 (1%)	0.169
Hypothyroidism	31,595 (9%)	34,512 (10%)	0.044	78,935 (4%)	44,456 (7%)	0.150	43,876 (3%)	3,642 (7%)	0.179	30,913 (2%)	18,516 (1%)	0.012
Malignant neoplastic disease	69,247 (20%)	82,610 (25%)	0.118	60,370 (3%)	76,103 (13%)	0.367	29,693 (2%)	6,311 (12%)	0.392	22,630 (1%)	5,104 (0%)	0.086

	Cohort 1			Cohort 2			Cohort 3			Cohort 4		
	**Unvaccinated**	**Vaccinated**	**ASMD**	**Unvaccinated**	**Vaccinated**	**ASMD**	**Unvaccinated**	**Vaccinated**	**ASMD**	**Unvaccinated**	**Vaccinated**	**ASMD**
Myocardial infarction	16,111 (5%)	18,289 (5%)	0.040	20,304 (1%)	23,019 (4%)	0.185	7,409 (0%)	1,464 (3%)	0.177	5,738 (0%)	715 (0%)	0.056
Osteoporosis	30,053 (9%)	36,535 (11%)	0.079	18,282 (1%)	21,789 (4%)	0.184	8,852 (1%)	2,632 (5%)	0.265	6,904 (0%)	1,028 (0%)	0.058
Pneumonia	17,134 (5%)	16,297 (5%)	0.001	31,724 (2%)	17,567 (3%)	0.091	17,791 (1%)	1,658 (3%)	0.131	13,263 (1%)	7,793 (1%)	0.010
Rheumatoid arthritis	6,613 (2%)	6,693 (2%)	0.008	14,823 (1%)	12,878 (2%)	0.118	5,533 (0%)	667 (1%)	0.097	3,692 (0%)	891 (0%)	0.033
Stroke	15,325 (4%)	15,601 (5%)	0.014	16,962 (1%)	15,056 (3%)	0.130	7,431 (0%)	1,281 (2%)	0.158	6,019 (0%)	1,022 (0%)	0.051
Venous thromboembolism	19,679 (6%)	21,549 (6%)	0.034	28,882 (1%)	23,632 (4%)	0.155	13,639 (1%)	1,980 (4%)	0.185	12,941 (1%)	2,541 (0%)	0.070

The 4 cohorts represent vaccine rollout periods. *Calculated as the days of previous observation in the database before index date. **Assessed anytime before index date. ASMD = Absolute standardized mean difference, GP = General practice, PCR = Polymerase chain reaction, COPD = Chronic obstructive pulmonary disease, GERD = Gastro-Esophageal reflux disease

Table S6: Characteristics of weighted populations in CPRD GOLD, database, stratified by staggered cohort and exposure status. Exposure is any COVID-19 vaccine.

	Cohort 1			Cohort 2			Cohort 3			Cohort 4		
	Unvaccinated	Vaccinated	ASMD	Unvaccinated	Vaccinated	ASMD	Unvaccinated	Vaccinated	ASMD	Unvaccinated	Vaccinated	ASMD
**N (individuals)**	40,084	40,014		111,674	111,393		124,883	124,955		196,235	195,302	
**Age, median [Q25-Q75]**	82 [78-86]	82 [78-86]	0.004	62 [50-69]	62 [50-69]	0.005	50 [41-58]	52 [41-58]	0.000	34 [26-42]	34 [26-43]	0.004
**Sex: Female, N(%)**	23,206 (58%)	23,175 (58%)	0.000	63,290 (57%)	63,079 (57%)	0.001	63,572 (51%)	63,955 (51%)	0.006	84,762 (43%)	85,083 (44%)	0.007
**Years of prior history*, median [Q25-Q75]**	17 [13-20]	18 [13-20]	0.001	17 [11-19]	17 [11-19]	0.005	17 [9-19]	17 [9-19]	0.000	13 [6-18]	14 [5-18]	0.006
**Number of GP visits, median [Q25-Q75]**	12 [7-20]	12 [8-20]	0.000	8 [3-15]	10 [5-15]	0.000	4 [0-10]	4 [1-10]	0.009	0 [0-5]	2 [0-5]	0.004
**Number of PCR tests, median [Q25-Q75]**	0[0-0]	0[0-0]	0.002	0[0-0]	0[0-0]	0.009	0[0-0]	0[0-0]	0.003	0[0-0]	0[0-0]	0.000
**Comorbidities**, N(%)**												
Anxiety	4,933 (12%)	4,920 (12%)	0.000	22,884 (20%)	22,299 (20%)	0.012	24,993 (20%)	24,695 (20%)	0.006	31,360 (16%)	30,689 (16%)	0.007
Asthma	3,911 (10%)	3,931 (10%)	0.002	18,514 (17%)	18,209 (16%)	0.006	17,431 (14%)	17,388 (14%)	0.001	14,817 (8%)	14,596 (7%)	0.003
Chronic kidney disease	8,998 (22%)	8,953 (22%)	0.002	6,848 (6%)	7,431 (7%)	0.022	2,120 (2%)	2,287 (2%)	0.010	922 (0%)	807 (0%)	0.009
COPD	3,439 (9%)	3,341 (8%)	0.008	5,972 (5%)	6,496 (6%)	0.021	2,089 (2%)	1,938 (2%)	0.010	734 (0%)	688 (0%)	0.004
Dementia	2,634 (7%)	2,332 (6%)	0.031	1,383 (1%)	865 (1%)	0.046	283 (0%)	313 (0%)	0.005	78 (0%)	134 (0%)	0.013
Depressive disorder	3,783 (9%)	3,705 (9%)	0.006	20,874 (19%)	20,246 (18%)	0.013	22,015 (18%)	21,682 (17%)	0.007	23,010 (12%)	22,630 (12%)	0.004
Diabetes	5,928 (15%)	5,802 (14%)	0.008	11,000 (10%)	11,657 (10%)	0.020	5,345 (4%)	5,651 (5%)	0.012	2,586 (1%)	2,366 (1%)	0.009
GERD	1,930 (5%)	1,970 (5%)	0.005	4,521 (4%)	4,479 (4%)	0.001	3,343 (3%)	3,346 (3%)	0.000	2,657 (1%)	2,614 (1%)	0.001
Heart failure	2,417 (6%)	2,205 (6%)	0.022	2,002 (2%)	2,029 (2%)	0.002	639 (1%)	614 (0%)	0.003	250 (0%)	207 (0%)	0.006
Hypertension	14,288 (36%)	14,282 (36%)	0.001	22,488 (20%)	22,695 (20%)	0.006	11,108 (9%)	11,313 (9%)	0.006	4,318 (2%)	4,619 (2%)	0.011
Hypothyroidism	3,151 (8%)	3,155 (8%)	0.001	6,321 (6%)	6,361 (6%)	0.002	4,134 (3%)	4,055 (3%)	0.004	2,475 (1%)	2,621 (1%)	0.007
Malignant neoplastic disease	8,302 (21%)	8,283 (21%)	0.000	8,661 (8%)	10,177 (9%)	0.050	3,813 (3%)	3,777 (3%)	0.002	1,614 (1%)	1,676 (1%)	0.004

Mercadé-Besora N, *et al. Heart* 2024; 110:635–643. doi: 10.1136/heartjnl-2023-323483

	Cohort 1			Cohort 2			Cohort 3			Cohort 4		
	Unvaccinated	Vaccinated	ASMD	Unvaccinated	Vaccinated	ASMD	Unvaccinated	Vaccinated	ASMD	Unvaccinated	Vaccinated	ASMD
Myocardial infarction	2,119 (5%)	1,984 (5%)	0.015	3,308 (3%)	3,740 (3%)	0.023	1,193 (1%)	1,267 (1%)	0.006	430 (0%)	343 (0%)	0.010
Osteoporosis	3,702 (9%)	3,670 (9%)	0.002	3,154 (3%)	3,179 (3%)	0.002	1,038 (1%)	1,030 (1%)	0.001	363 (0%)	375 (0%)	0.002
Pneumonia	1,654 (4%)	1,452 (4%)	0.026	2,424 (2%)	2,211 (2%)	0.013	1,401 (1%)	1,314 (1%)	0.007	1,035 (1%)	1,055 (1%)	0.002
Rheumatoid arthritis	637 (2%)	624 (2%)	0.002	1,618 (1%)	1,818 (2%)	0.015	551 (0%)	760 (1%)	0.023	280 (0%)	157 (0%)	0.019
Stroke	2,103 (5%)	1,866 (5%)	0.027	2,468 (2%)	2,512 (2%)	0.003	916 (1%)	981 (1%)	0.006	385 (0%)	356 (0%)	0.003
Venous thromboembolism	1,732 (4%)	1,648 (4%)	0.010	2,639 (2%)	2,596 (2%)	0.002	1,466 (1%)	1,638 (1%)	0.012	959 (0%)	518 (0%)	0.036

The 4 cohorts represent vaccine rollout periods. *Calculated as the days of previous observation in the database before index date. **Assessed anytime before index date. ASMD = Absolute standardized mean difference, GP = General practice, PCR = Polymerase chain reaction, COPD = Chronic obstructive pulmonary disease, GERD = Gastro-Esophageal reflux disease

Mercadé-Besora N, *et al. Heart* 2024; 110:635–643. doi: 10.1136/heartjnl-2023-323483

Table S7: Characteristics of unweighted populations in CPRD GOLD, database, stratified by staggered cohort and exposure status. Exposure is any COVID-19 vaccine.

	Cohort 1			Cohort 2			Cohort 3			Cohort 4		
	**Unvaccinated**	**Vaccinated**	**ASMD**	**Unvaccinated**	**Vaccinated**	**ASMD**	**Unvaccinated**	**Vaccinated**	**ASMD**	**Unvaccinated**	**Vaccinated**	**ASMD**
**N (individuals)**	169,100	118,507		583,399	486,619		417,996	462,832		469,876	550,437	
**Age, median [Q25-Q75]**	78 [76-82]	84 [81-87]	0.550	45 [33-58]	71 [66-75]	1.117	42 [31-55]	56 [52-61]	0.546	37 [28-49]	35 [27-43]	0.250
**Sex: Female, N(%)**	93,719 (55%)	68,258 (58%)	0.044	344,747 (59%)	262,113 (54%)	0.106	230,567 (55%)	230,957 (50%)	0.105	208,184 (44%)	249,603 (45%)	0.021
**Years of prior history*, median [Q25-Q75]**	17 [13-20]	18 [14-20]	0.023	17 [10-19]	17 [13-20]	0.085	17 [9-19]	17 [10-19]	0.055	13 [6-18]	14 [6-18]	0.020
**Number of GP visits, median [Q25-Q75]**	11 [6-18]	14 [9-21]	0.180	5 [1-11]	11 [6-17]	0.414	3 [0-8]	6 [2-12]	0.239	0 [0-4]	2 [0-6]	0.115
**Number of PCR tests, median [Q25-Q75]**	0[0-0]	0[0-0]	0.052	0[0-0]	0[0-0]	0.069	0[0-0]	0[0-0]	0.016	0[0-0]	0[0-0]	0.044
**Comorbidities**, N(%)**												
Anxiety	20,097 (12%)	14,878 (13%)	0.020	132,282 (23%)	78,442 (16%)	0.166	83,022 (20%)	83,629 (18%)	0.046	65,306 (14%)	88,512 (16%)	0.061
Asthma	15,573 (9%)	12,090 (10%)	0.034	119,520 (20%)	61,821 (13%)	0.210	68,746 (16%)	52,312 (11%)	0.149	30,811 (7%)	46,774 (8%)	0.074
Chronic kidney disease	29,343 (17%)	29,934 (25%)	0.194	15,484 (3%)	49,550 (10%)	0.311	6,676 (2%)	8,145 (2%)	0.013	4,746 (1%)	1,376 (0%)	0.096
COPD	14,248 (8%)	9,602 (8%)	0.012	12,247 (2%)	39,371 (8%)	0.275	5,114 (1%)	6,699 (1%)	0.020	3,716 (1%)	953 (0%)	0.089
Dementia	6,678 (4%)	6,860 (6%)	0.086	1,986 (0%)	6,557 (1%)	0.110	1,188 (0%)	636 (0%)	0.032	774 (0%)	159 (0%)	0.044
Depressive disorder	16,339 (10%)	10,720 (9%)	0.021	111,568 (19%)	72,063 (15%)	0.115	66,705 (16%)	77,560 (17%)	0.022	50,341 (11%)	64,215 (12%)	0.030
Diabetes	23,579 (14%)	17,895 (15%)	0.033	29,125 (5%)	66,688 (14%)	0.303	12,407 (3%)	25,465 (6%)	0.126	9,225 (2%)	5,012 (1%)	0.089
GERD	7,610 (5%)	6,174 (5%)	0.033	18,053 (3%)	22,884 (5%)	0.083	8,864 (2%)	15,586 (3%)	0.076	5,920 (1%)	8,482 (2%)	0.024
Heart failure	7,475 (4%)	7,118 (6%)	0.071	4,013 (1%)	12,939 (3%)	0.154	1,732 (0%)	2,410 (1%)	0.016	1,274 (0%)	293 (0%)	0.054
Hypertension	56,298 (33%)	44,709 (38%)	0.093	58,662 (10%)	139,644 (29%)	0.485	24,457 (6%)	64,451 (14%)	0.273	16,440 (3%)	11,991 (2%)	0.080
Hypothyroidism	12,126 (7%)	9,825 (8%)	0.042	22,026 (4%)	31,923 (7%)	0.126	10,616 (3%)	19,001 (4%)	0.087	6,676 (1%)	7,790 (1%)	0.000
Malignant neoplastic disease	30,687 (18%)	26,725 (23%)	0.110	19,781 (3%)	65,779 (14%)	0.370	8,870 (2%)	19,474 (4%)	0.119	5,916 (1%)	4,164 (1%)	0.050

	Cohort 1			Cohort 2			Cohort 3			Cohort 4		
	**Unvaccinated**	**Vaccinated**	**ASMD**	**Unvaccinated**	**Vaccinated**	**ASMD**	**Unvaccinated**	**Vaccinated**	**ASMD**	**Unvaccinated**	**Vaccinated**	**ASMD**
Myocardial infarction	7,707 (5%)	6,298 (5%)	0.035	8,409 (1%)	20,200 (4%)	0.165	2,665 (1%)	5,556 (1%)	0.059	2,003 (0%)	501 (0%)	0.066
Osteoporosis	12,736 (8%)	11,677 (10%)	0.082	5,836 (1%)	23,843 (5%)	0.232	2,580 (1%)	4,797 (1%)	0.046	1,855 (0%)	637 (0%)	0.055
Pneumonia	5,401 (3%)	4,381 (4%)	0.028	7,250 (1%)	12,120 (2%)	0.092	3,822 (1%)	4,889 (1%)	0.014	2,522 (1%)	2,797 (1%)	0.004
Rheumatoid arthritis	2,565 (2%)	1,833 (2%)	0.002	4,013 (1%)	9,000 (2%)	0.104	1,294 (0%)	2,566 (1%)	0.037	877 (0%)	316 (0%)	0.037
Stroke	7,214 (4%)	5,843 (5%)	0.032	5,886 (1%)	14,921 (3%)	0.146	2,170 (1%)	3,998 (1%)	0.042	1,591 (0%)	620 (0%)	0.048
Venous thromboembolism	6,188 (4%)	5,096 (4%)	0.033	7,342 (1%)	14,077 (3%)	0.115	3,067 (1%)	6,732 (1%)	0.069	2,611 (1%)	1,018 (0%)	0.061

The 4 cohorts represent vaccine rollout periods. *Calculated as the days of previous observation in the database before index date. **Assessed anytime before index date. ASMD = Absolute standardized mean difference, GP = General practice, PCR = Polymerase chain reaction, COPD = Chronic obstructive pulmonary disease, GERD = Gastro-Esophageal reflux disease

Table S8: Characteristics of weighted populations in CPRD GOLD, database, stratified by staggered cohort and exposure status. Exposure is ChAdOx1 vaccine.

	Cohort 1			Cohort 2			Cohort 3			Cohort 4		
	Unvaccinated	Vaccinated	ASMD	Unvaccinated	Vaccinated	ASMD	Unvaccinated	Vaccinated	ASMD	Unvaccinated	Vaccinated	ASMD
**N (individuals)**	29,163	29,082		82,091	82,226		116,090	115,912		70,312	70,823	
**Age, median [Q25-Q75]**	82 [79-87]	82 [80-87]	0.002	64 [53-71]	64 [53-70]	0.001	52 [42-58]	52 [42-58]	0.003	42 [39-48]	42 [38-48]	0.000
**Sex: Female, N(%)**	17,126 (59%)	17,076 (59%)	0.000	45,269 (55%)	45,487 (55%)	0.003	58,599 (50%)	58,799 (51%)	0.005	29,480 (42%)	29,458 (42%)	0.007
**Years of prior history*, median [Q25-Q75]**	17 [13-20]	17 [14-20]	0.001	17 [11-19]	17 [11-19]	0.002	17 [9-19]	17 [8-19]	0.001	14 [7-18]	14 [6-18]	0.001
**Number of GP visits, median [Q25-Q75]**	12 [7-21]	12 [8-20]	0.005	8 [3-16]	10 [5-16]	0.002	4 [0-10]	4 [1-10]	0.014	0 [0-6]	2 [0-6]	0.003
**Number of PCR tests, median [Q25-Q75]**	0[0-0]	0[0-0]	0.005	0[0-0]	0[0-0]	0.005	0[0-0]	0[0-0]	0.004	0[0-0]	0[0-0]	0.000
**Comorbidities**, N(%)**												
Anxiety	3,481 (12%)	3,496 (12%)	0.003	16,202 (20%)	15,970 (19%)	0.008	23,083 (20%)	22,695 (20%)	0.008	12,154 (17%)	11,947 (17%)	0.011
Asthma	2,863 (10%)	2,870 (10%)	0.002	13,358 (16%)	13,192 (16%)	0.006	16,057 (14%)	16,029 (14%)	0.000	4,767 (7%)	4,744 (7%)	0.003
Chronic kidney disease	6,959 (24%)	6,928 (24%)	0.001	5,850 (7%)	6,384 (8%)	0.024	2,031 (2%)	2,104 (2%)	0.005	616 (1%)	517 (1%)	0.016
COPD	2,556 (9%)	2,485 (9%)	0.008	5,069 (6%)	5,523 (7%)	0.022	1,969 (2%)	1,874 (2%)	0.006	539 (1%)	478 (1%)	0.011
Dementia	2,042 (7%)	1,794 (6%)	0.034	1,319 (2%)	826 (1%)	0.053	263 (0%)	265 (0%)	0.000	59 (0%)	111 (0%)	0.021
Depressive disorder	2,641 (9%)	2,608 (9%)	0.003	14,875 (18%)	14,558 (18%)	0.011	20,497 (18%)	20,298 (18%)	0.004	10,492 (15%)	10,218 (14%)	0.014
Diabetes	4,346 (15%)	4,273 (15%)	0.006	8,933 (11%)	9,524 (12%)	0.022	5,139 (4%)	5,350 (5%)	0.009	1,459 (2%)	1,421 (2%)	0.005
GERD	1,366 (5%)	1,380 (5%)	0.003	3,514 (4%)	3,414 (4%)	0.006	3,153 (3%)	3,136 (3%)	0.001	1,258 (2%)	1,297 (2%)	0.003
Heart failure	1,844 (6%)	1,699 (6%)	0.020	1,793 (2%)	1,720 (2%)	0.006	613 (1%)	601 (1%)	0.001	177 (0%)	151 (0%)	0.008
Hypertension	10,466 (36%)	10,581 (36%)	0.010	17,843 (22%)	18,115 (22%)	0.007	10,489 (9%)	10,714 (9%)	0.007	3,119 (4%)	3,233 (5%)	0.006
Hypothyroidism	2,338 (8%)	2,360 (8%)	0.004	4,857 (6%)	4,837 (6%)	0.001	3,897 (3%)	3,742 (3%)	0.007	1,432 (2%)	1,412 (2%)	0.003
Malignant neoplastic disease	6,210 (21%)	6,164 (21%)	0.002	7,090 (9%)	8,270 (10%)	0.049	3,588 (3%)	3,590 (3%)	0.000	1,059 (2%)	1,117 (2%)	0.006
Myocardial infarction	1,569 (5%)	1,500 (5%)	0.010	2,720 (3%)	3,083 (4%)	0.024	1,155 (1%)	1,202 (1%)	0.004	319 (0%)	242 (0%)	0.018

	Cohort 1			Cohort 2			Cohort 3			Cohort 4		
	Unvaccinated	Vaccinated	ASMD	Unvaccinated	Vaccinated	ASMD	Unvaccinated	Vaccinated	ASMD	Unvaccinated	Vaccinated	ASMD
Osteoporosis	2,838 (10%)	2,846 (10%)	0.002	2,715 (3%)	2,668 (3%)	0.004	944 (1%)	986 (1%)	0.004	252 (0%)	281 (0%)	0.006
Pneumonia	1,287 (4%)	1,147 (4%)	0.024	1,997 (2%)	1,883 (2%)	0.009	1,307 (1%)	1,245 (1%)	0.005	487 (1%)	473 (1%)	0.003
Rheumatoid arthritis	464 (2%)	445 (2%)	0.005	1,280 (2%)	1,491 (2%)	0.020	514 (0%)	743 (1%)	0.027	184 (0%)	120 (0%)	0.020
Stroke	1,600 (5%)	1,421 (5%)	0.027	2,073 (3%)	2,122 (3%)	0.003	873 (1%)	963 (1%)	0.009	263 (0%)	257 (0%)	0.002
Venous thromboembolism	1,339 (5%)	1,191 (4%)	0.024	2,112 (3%)	2,219 (3%)	0.008	1,407 (1%)	1,572 (1%)	0.013	590 (1%)	297 (0%)	0.053

The 4 cohorts represent vaccine rollout periods. *Calculated as the days of previous observation in the database before index date. **Assessed anytime before index date. ASMD = Absolute standardized mean difference, GP = General practice, PCR = Polymerase chain reaction, COPD = Chronic obstructive pulmonary disease, GERD = Gastro-Esophageal reflux disease

Mercadé-Besora N, *et al. Heart* 2024; 110:635–643. doi: 10.1136/heartjnl-2023-323483

Table S9: Characteristics of unweighted populations in CPRD GOLD, database, stratified by staggered cohort and exposure status. Exposure is ChAdOx1 vaccine.

	Cohort 1			Cohort 2			Cohort 3			Cohort 4		
	**Unvaccinated**	**Vaccinated**	**ASMD**	**Unvaccinated**	**Vaccinated**	**ASMD**	**Unvaccinated**	**Vaccinated**	**ASMD**	**Unvaccinated**	**Vaccinated**	**ASMD**
**N (individuals)**	168,972	82,406		582,791	302,999		418,184	423,876		485,154	147,744	
**Age, median [Q25-Q75]**	78 [76-82]	84 [82-88]	0.657	45 [33-58]	71 [66-76]	1.153	42 [31-55]	56 [52-61]	0.560	37 [28-49]	44 [41-48]	0.262
**Sex: Female, N(%)**	93,648 (55%)	47,915 (58%)	0.055	344,408 (59%)	162,753 (54%)	0.109	230,680 (55%)	210,283 (50%)	0.111	215,080 (44%)	64,999 (44%)	0.007
**Years of prior history*, median [Q25-Q75]**	17 [13-20]	17 [14-20]	0.016	17 [10-19]	17 [13-19]	0.090	17 [9-19]	17 [10-19]	0.047	13 [6-18]	14 [7-18]	0.054
**Number of GP visits, median [Q25-Q75]**	11 [6-18]	14 [9-21]	0.177	5 [1-11]	11 [7-18]	0.451	3 [0-8]	6 [2-12]	0.245	0 [0-4]	3 [0-7]	0.174
**Number of PCR tests, median [Q25-Q75]**	0[0-0]	0[0-0]	0.053	0[0-0]	0[0-0]	0.074	0[0-0]	0[0-0]	0.018	0[0-0]	0[0-0]	0.029
**Comorbidities**, N(%)**												
Anxiety	20,087 (12%)	9,923 (12%)	0.005	132,145 (23%)	49,010 (16%)	0.165	83,054 (20%)	75,258 (18%)	0.054	66,918 (14%)	24,601 (17%)	0.080
Asthma	15,567 (9%)	8,491 (10%)	0.037	119,442 (20%)	39,988 (13%)	0.196	68,792 (16%)	48,034 (11%)	0.148	31,538 (7%)	10,517 (7%)	0.025
Chronic kidney disease	29,280 (17%)	21,564 (26%)	0.216	15,435 (3%)	34,168 (11%)	0.344	6,675 (2%)	7,553 (2%)	0.014	4,947 (1%)	739 (1%)	0.060
COPD	14,234 (8%)	6,837 (8%)	0.005	12,242 (2%)	27,472 (9%)	0.307	5,122 (1%)	6,334 (1%)	0.023	3,834 (1%)	640 (0%)	0.046
Dementia	6,664 (4%)	4,773 (6%)	0.086	1,998 (0%)	5,124 (2%)	0.135	1,201 (0%)	548 (0%)	0.035	831 (0%)	126 (0%)	0.024
Depressive disorder	16,333 (10%)	6,998 (8%)	0.041	111,526 (19%)	45,154 (15%)	0.113	66,763 (16%)	70,703 (17%)	0.019	51,687 (11%)	21,711 (15%)	0.122
Diabetes	23,547 (14%)	12,387 (15%)	0.031	29,089 (5%)	43,647 (14%)	0.322	12,392 (3%)	24,219 (6%)	0.135	9,455 (2%)	2,254 (2%)	0.032
GERD	7,613 (5%)	4,054 (5%)	0.020	18,044 (3%)	14,294 (5%)	0.084	8,869 (2%)	14,447 (3%)	0.079	6,051 (1%)	2,883 (2%)	0.056
Heart failure	7,468 (4%)	5,058 (6%)	0.077	4,016 (1%)	9,180 (3%)	0.174	1,738 (0%)	2,308 (1%)	0.019	1,316 (0%)	191 (0%)	0.032
Hypertension	56,262 (33%)	31,217 (38%)	0.096	58,578 (10%)	88,833 (29%)	0.499	24,450 (6%)	59,554 (14%)	0.277	16,971 (3%)	6,761 (5%)	0.055
Hypothyroidism	12,132 (7%)	7,042 (9%)	0.051	21,999 (4%)	20,521 (7%)	0.134	10,622 (3%)	17,382 (4%)	0.087	6,848 (1%)	3,245 (2%)	0.059
Malignant neoplastic disease	30,670 (18%)	18,950 (23%)	0.120	19,707 (3%)	42,687 (14%)	0.386	8,893 (2%)	17,903 (4%)	0.120	6,143 (1%)	2,192 (1%)	0.019

Mercadé-Besora N, *et al. Heart* 2024; 110:635–643. doi: 10.1136/heartjnl-2023-323483

	Cohort 1			Cohort 2			Cohort 3			Cohort 4		
	**Unvaccinated**	**Vaccinated**	**ASMD**	**Unvaccinated**	**Vaccinated**	**ASMD**	**Unvaccinated**	**Vaccinated**	**ASMD**	**Unvaccinated**	**Vaccinated**	**ASMD**
Myocardial infarction	7,704 (5%)	4,500 (5%)	0.041	8,413 (1%)	13,456 (4%)	0.178	2,660 (1%)	5,280 (1%)	0.063	2,057 (0%)	330 (0%)	0.035
Osteoporosis	12,732 (8%)	8,564 (10%)	0.100	5,847 (1%)	16,117 (5%)	0.249	2,576 (1%)	4,330 (1%)	0.045	1,923 (0%)	408 (0%)	0.021
Pneumonia	5,401 (3%)	3,231 (4%)	0.039	7,241 (1%)	8,604 (3%)	0.113	3,814 (1%)	4,527 (1%)	0.016	2,615 (1%)	921 (1%)	0.011
Rheumatoid arthritis	2,559 (2%)	1,257 (2%)	0.001	4,017 (1%)	5,999 (2%)	0.113	1,286 (0%)	2,405 (1%)	0.039	918 (0%)	191 (0%)	0.015
Stroke	7,200 (4%)	4,193 (5%)	0.039	5,885 (1%)	10,213 (3%)	0.162	2,167 (1%)	3,789 (1%)	0.045	1,625 (0%)	369 (0%)	0.016
Venous thromboembolism	6,185 (4%)	3,550 (4%)	0.033	7,335 (1%)	9,466 (3%)	0.128	3,072 (1%)	6,339 (1%)	0.073	2,712 (1%)	460 (0%)	0.038

The 4 cohorts represent vaccine rollout periods. *Calculated as the days of previous observation in the database before index date. **Assessed anytime before index date. ASMD = Absolute standardized mean difference, GP = General practice, PCR = Polymerase chain reaction, COPD = Chronic obstructive pulmonary disease, GERD = Gastro-Esophageal reflux disease

Mercadé-Besora N, *et al. Heart* 2024; 110:635–643. doi: 10.1136/heartjnl-2023-323483

Table S10: Characteristics of weighted populations in CPRD GOLD, database, stratified by staggered cohort and exposure status. Exposure is BNT162b2 vaccine.

	Cohort 1			Cohort 2			Cohort 3			Cohort 4		
	**Unvaccinated**	**Vaccinated**	**ASMD**	**Unvaccinated**	**Vaccinated**	**ASMD**	**Unvaccinated**	**Vaccinated**	**ASMD**	**Unvaccinated**	**Vaccinated**	**ASMD**
**N (individuals)**	15,295	15,304		60,813	61,113		24,008	24,157		151,713	152,100	
**Age, median [Q25-Q75]**	80 [77-85]	80 [77-85]	0.000	64 [53-70]	66 [52-70]	0.008	52 [44-58]	52 [44-58]	0.004	32 [24-38]	32 [24-38]	0.003
**Sex: Female, N(%)**	8,644 (57%)	8,657 (57%)	0.001	34,612 (57%)	34,776 (57%)	0.000	12,846 (54%)	12,993 (54%)	0.006	66,947 (44%)	67,555 (44%)	0.006
**Years of prior history*, median [Q25-Q75]**	17 [12-20]	18 [12-21]	0.001	17 [12-19]	17 [11-19]	0.003	17 [11-19]	17 [11-19]	0.004	13 [6-18]	14 [5-18]	0.001
**Number of GP visits, median [Q25-Q75]**	12 [7-20]	12 [8-20]	0.001	8 [3-15]	8 [4-15]	0.009	4 [0-10]	4 [1-10]	0.006	0 [0-5]	2 [0-5]	0.007
**Number of PCR tests, median [Q25-Q75]**	0[0-0]	0[0-0]	0.006	0[0-0]	0[0-0]	0.004	0[0-0]	0[0-0]	0.001	0[0-0]	0[0-0]	0.013
**Comorbidities**, N(%)**												
Anxiety	1,969 (13%)	1,940 (13%)	0.006	11,790 (19%)	11,914 (19%)	0.003	5,342 (22%)	5,235 (22%)	0.014	24,455 (16%)	23,928 (16%)	0.011
Asthma	1,498 (10%)	1,488 (10%)	0.002	9,126 (15%)	9,093 (15%)	0.004	2,926 (12%)	2,985 (12%)	0.005	12,195 (8%)	11,929 (8%)	0.007
Chronic kidney disease	3,112 (20%)	3,057 (20%)	0.009	3,563 (6%)	3,764 (6%)	0.013	398 (2%)	407 (2%)	0.002	468 (0%)	346 (0%)	0.016
COPD	1,190 (8%)	1,151 (8%)	0.010	3,222 (5%)	3,237 (5%)	0.000	293 (1%)	274 (1%)	0.008	315 (0%)	250 (0%)	0.010
Dementia	1,087 (7%)	864 (6%)	0.060	646 (1%)	355 (1%)	0.053	55 (0%)	80 (0%)	0.019	21 (0%)	28 (0%)	0.004
Depressive disorder	1,527 (10%)	1,496 (10%)	0.007	10,709 (18%)	10,912 (18%)	0.006	4,382 (18%)	4,343 (18%)	0.007	17,112 (11%)	16,594 (11%)	0.012
Diabetes	2,193 (14%)	2,173 (14%)	0.004	6,057 (10%)	6,199 (10%)	0.006	883 (4%)	858 (4%)	0.007	1,609 (1%)	1,271 (1%)	0.023
GERD	880 (6%)	872 (6%)	0.003	2,440 (4%)	2,505 (4%)	0.004	659 (3%)	706 (3%)	0.011	1,935 (1%)	1,961 (1%)	0.001
Heart failure	857 (6%)	748 (5%)	0.032	1,097 (2%)	933 (2%)	0.022	119 (0%)	77 (0%)	0.027	107 (0%)	77 (0%)	0.008
Hypertension	5,338 (35%)	5,312 (35%)	0.004	12,828 (21%)	12,901 (21%)	0.000	2,597 (11%)	2,645 (11%)	0.004	2,260 (1%)	2,369 (2%)	0.006
Hypothyroidism	1,129 (7%)	1,067 (7%)	0.016	3,476 (6%)	3,416 (6%)	0.005	935 (4%)	954 (4%)	0.003	1,772 (1%)	1,653 (1%)	0.008
Malignant neoplastic disease	3,126 (20%)	3,097 (20%)	0.005	5,084 (8%)	5,529 (9%)	0.024	864 (4%)	922 (4%)	0.011	877 (1%)	902 (1%)	0.002
Myocardial infarction	764 (5%)	681 (4%)	0.026	1,797 (3%)	1,853 (3%)	0.005	230 (1%)	186 (1%)	0.020	189 (0%)	128 (0%)	0.013

Mercadé-Besora N, *et al. Heart* 2024; 110:635–643. doi: 10.1136/heartjnl-2023-323483

	Cohort 1			Cohort 2			Cohort 3			Cohort 4		
	**Unvaccinated**	**Vaccinated**	**ASMD**	**Unvaccinated**	**Vaccinated**	**ASMD**	**Unvaccinated**	**Vaccinated**	**ASMD**	**Unvaccinated**	**Vaccinated**	**ASMD**
Osteoporosis	1,312 (9%)	1,225 (8%)	0.021	1,775 (3%)	1,805 (3%)	0.002	251 (1%)	282 (1%)	0.012	154 (0%)	135 (0%)	0.004
Pneumonia	573 (4%)	475 (3%)	0.035	1,232 (2%)	1,107 (2%)	0.016	256 (1%)	244 (1%)	0.005	744 (0%)	698 (0%)	0.005
Rheumatoid arthritis	248 (2%)	234 (2%)	0.008	854 (1%)	945 (2%)	0.012	113 (0%)	102 (0%)	0.007	165 (0%)	62 (0%)	0.025
Stroke	739 (5%)	650 (4%)	0.028	1,291 (2%)	1,297 (2%)	0.000	172 (1%)	129 (1%)	0.023	204 (0%)	138 (0%)	0.013
Venous thromboembolism	640 (4%)	622 (4%)	0.006	1,355 (2%)	1,347 (2%)	0.002	273 (1%)	256 (1%)	0.008	604 (0%)	288 (0%)	0.039

The 4 cohorts represent vaccine rollout periods. *Calculated as the days of previous observation in the database before index date. **Assessed anytime before index date. ASMD = Absolute standardized mean difference, GP = General practice, PCR = Polymerase chain reaction, COPD = Chronic obstructive pulmonary disease, GERD = Gastro-Esophageal reflux disease

Mercadé-Besora N, *et al. Heart* 2024; 110:635–643. doi: 10.1136/heartjnl-2023-323483

Table S11: Characteristics of unweighted populations in CPRD GOLD, database, stratified by staggered cohort and exposure status. Exposure is BNT162b2 vaccine.

	Cohort 1			Cohort 2			Cohort 3			Cohort 4		
	**Unvaccinated**	**Vaccinated**	**ASMD**	**Unvaccinated**	**Vaccinated**	**ASMD**	**Unvaccinated**	**Vaccinated**	**ASMD**	**Unvaccinated**	**Vaccinated**	**ASMD**
**N (individuals)**	169,459	32,755		584,309	180,670		416,549	36,748		465,326	365,096	
**Age, median [Q25-Q75]**	78 [76-82]	81 [79-86]	0.306	45 [33-58]	70 [66-74]	1.059	42 [31-55]	54 [50-60]	0.407	37 [28-49]	31 [24-37]	0.445
**Sex: Female, N(%)**	93,939 (55%)	18,415 (56%)	0.016	345,249 (59%)	97,747 (54%)	0.101	229,807 (55%)	19,649 (53%)	0.034	206,080 (44%)	167,510 (46%)	0.032
**Years of prior history*, median [Q25-Q75]**	17 [13-20]	18 [13-21]	0.017	17 [10-19]	17 [12-19]	0.076	17 [9-19]	18 [12-20]	0.153	13 [6-18]	15 [6-18]	0.018
**Number of GP visits, median [Q25-Q75]**	11 [6-18]	14 [9-21]	0.172	5 [1-11]	10 [6-16]	0.348	3 [0-8]	6 [2-11]	0.183	0 [0-4]	2 [0-6]	0.092
**Number of PCR tests, median [Q25-Q75]**	0[0-0]	0[0-0]	0.046	0[0-0]	0[0-0]	0.061	0[0-0]	0[0-0]	0.005	0[0-0]	0[0-0]	0.045
**Comorbidities**, N(%)**												
Anxiety	20,145 (12%)	4,336 (13%)	0.041	132,397 (23%)	28,697 (16%)	0.172	82,752 (20%)	8,004 (22%)	0.047	64,849 (14%)	59,094 (16%)	0.063
Asthma	15,611 (9%)	3,250 (10%)	0.024	119,679 (20%)	21,396 (12%)	0.236	68,556 (16%)	4,053 (11%)	0.158	30,579 (7%)	33,240 (9%)	0.094
Chronic kidney disease	29,378 (17%)	7,430 (23%)	0.134	15,504 (3%)	15,033 (8%)	0.251	6,636 (2%)	566 (2%)	0.004	4,692 (1%)	578 (0%)	0.112
COPD	14,265 (8%)	2,417 (7%)	0.039	12,276 (2%)	11,545 (6%)	0.214	5,085 (1%)	348 (1%)	0.026	3,657 (1%)	285 (0%)	0.108
Dementia	6,705 (4%)	1,895 (6%)	0.085	2,008 (0%)	1,375 (1%)	0.056	1,168 (0%)	87 (0%)	0.009	746 (0%)	29 (0%)	0.053
Depressive disorder	16,385 (10%)	3,310 (10%)	0.015	111,708 (19%)	26,294 (15%)	0.122	66,510 (16%)	6,569 (18%)	0.051	49,969 (11%)	39,181 (11%)	0.000
Diabetes	23,614 (14%)	4,882 (15%)	0.028	29,200 (5%)	22,552 (12%)	0.267	12,338 (3%)	1,191 (3%)	0.016	9,143 (2%)	2,507 (1%)	0.112
GERD	7,655 (5%)	2,012 (6%)	0.072	18,069 (3%)	8,525 (5%)	0.084	8,839 (2%)	1,093 (3%)	0.054	5,864 (1%)	5,213 (1%)	0.015
Heart failure	7,502 (4%)	1,782 (5%)	0.047	4,026 (1%)	3,669 (2%)	0.116	1,715 (0%)	100 (0%)	0.024	1,236 (0%)	98 (0%)	0.063
Hypertension	56,411 (33%)	11,995 (37%)	0.070	58,749 (10%)	49,763 (28%)	0.459	24,301 (6%)	4,679 (13%)	0.239	16,363 (4%)	4,776 (1%)	0.144
Hypothyroidism	12,163 (7%)	2,373 (7%)	0.003	22,061 (4%)	11,106 (6%)	0.109	10,565 (3%)	1,553 (4%)	0.094	6,617 (1%)	4,180 (1%)	0.025
Malignant neoplastic disease	30,728 (18%)	6,972 (21%)	0.079	19,832 (3%)	22,617 (13%)	0.342	8,827 (2%)	1,522 (4%)	0.116	5,804 (1%)	1,796 (0%)	0.081

Mercadé-Besora N, *et al. Heart* 2024; 110:635–643. doi: 10.1136/heartjnl-2023-323483

	Cohort 1			Cohort 2			Cohort 3			Cohort 4		
	**Unvaccinated**	**Vaccinated**	**ASMD**	**Unvaccinated**	**Vaccinated**	**ASMD**	**Unvaccinated**	**Vaccinated**	**ASMD**	**Unvaccinated**	**Vaccinated**	**ASMD**
Myocardial infarction	7,716 (5%)	1,603 (5%)	0.016	8,437 (1%)	6,621 (4%)	0.141	2,644 (1%)	271 (1%)	0.012	1,985 (0%)	154 (0%)	0.080
Osteoporosis	12,776 (8%)	2,770 (8%)	0.034	5,867 (1%)	7,531 (4%)	0.200	2,558 (1%)	458 (1%)	0.066	1,841 (0%)	205 (0%)	0.072
Pneumonia	5,415 (3%)	1,032 (3%)	0.003	7,266 (1%)	3,418 (2%)	0.052	3,790 (1%)	345 (1%)	0.003	2,512 (1%)	1,696 (0%)	0.011
Rheumatoid arthritis	2,566 (2%)	512 (2%)	0.004	4,018 (1%)	2,929 (2%)	0.087	1,276 (0%)	154 (0%)	0.019	881 (0%)	111 (0%)	0.048
Stroke	7,203 (4%)	1,490 (5%)	0.015	5,907 (1%)	4,632 (3%)	0.117	2,152 (1%)	199 (1%)	0.003	1,578 (0%)	225 (0%)	0.062
Venous thromboembolism	6,198 (4%)	1,389 (4%)	0.030	7,333 (1%)	4,490 (2%)	0.091	3,056 (1%)	370 (1%)	0.029	2,585 (1%)	496 (0%)	0.072

The 4 cohorts represent vaccine rollout periods. *Calculated as the days of previous observation in the database before index date. **Assessed anytime before index date. ASMD = Absolute standardized mean difference, GP = General practice, PCR = Polymerase chain reaction, COPD = Chronic obstructive pulmonary disease, GERD = Gastro-Esophageal reflux disease

Table S12: Characteristics of weighted populations in SIDIAP, database, stratified by staggered cohort and exposure status. Exposure is any COVID-19 vaccine.

	Cohort 1			Cohort 2			Cohort 3			Cohort 4		
	**Unvaccinated**	**Vaccinated**	**ASMD**	**Unvaccinated**	**Vaccinated**	**ASMD**	**Unvaccinated**	**Vaccinated**	**ASMD**	**Unvaccinated**	**Vaccinated**	**ASMD**
**N (individuals)**	58,342	58,535		235,543	235,780		344,520	342,678		413,428	412,893	
**Age, median [Q25-Q75]**	86 [83-89]	86 [82-89]	0.002	68 [63-73]	68 [63-74]	0.003	52 [46-58]	52 [46-59]	0.000	36 [28-44]	36 [28-44]	0.002
**Sex: Female, N(%)**	36,115 (62%)	36,214 (62%)	0.001	128,806 (55%)	129,053 (55%)	0.001	164,316 (48%)	163,188 (48%)	0.001	188,426 (46%)	188,471 (46%)	0.001
**Years of prior history*, median [Q25-Q75]**	15 [15-15]	15 [15-15]	0.001	15 [15-15]	15 [15-15]	0.000	15 [15-15]	15 [15-15]	0.003	15 [13-16]	15 [13-16]	0.004
**Number of GP visits, median [Q25-Q75]**	8 [3-15]	8 [4-15]	0.007	4 [0-9]	4 [1-9]	0.014	0 [0-6]	2 [0-6]	0.001	0 [0-4]	0 [0-5]	0.014
**Number of PCR tests, median [Q25-Q75]**	0[0-0]	0[0-0]	0.006	0[0-0]	0[0-0]	0.002	0[0-0]	0[0-0]	0.004	0[0-0]	0[0-0]	0.009
**Comorbidities**, N(%)**												
Anxiety	11,384 (20%)	11,467 (20%)	0.002	52,314 (22%)	52,262 (22%)	0.001	81,514 (24%)	80,775 (24%)	0.002	84,856 (21%)	83,145 (20%)	0.010
Asthma	3,708 (6%)	3,627 (6%)	0.007	10,973 (5%)	10,703 (5%)	0.006	14,915 (4%)	14,492 (4%)	0.005	19,969 (5%)	19,628 (5%)	0.004
Chronic kidney disease	18,755 (32%)	18,803 (32%)	0.001	21,147 (9%)	21,249 (9%)	0.001	7,411 (2%)	7,335 (2%)	0.001	3,293 (1%)	2,846 (1%)	0.012
COPD	7,008 (12%)	6,983 (12%)	0.002	17,160 (7%)	16,784 (7%)	0.006	8,852 (3%)	8,622 (3%)	0.003	3,210 (1%)	3,215 (1%)	0.000
Dementia	5,686 (10%)	5,812 (10%)	0.006	4,628 (2%)	4,425 (2%)	0.006	1,247 (0%)	1,229 (0%)	0.001	673 (0%)	689 (0%)	0.001
Depressive disorder	10,960 (19%)	11,020 (19%)	0.001	34,232 (15%)	34,571 (15%)	0.004	34,243 (10%)	32,427 (9%)	0.016	23,679 (6%)	23,024 (6%)	0.007
Diabetes	14,529 (25%)	14,269 (24%)	0.012	39,335 (17%)	39,133 (17%)	0.003	25,964 (8%)	26,135 (8%)	0.003	14,136 (3%)	14,091 (3%)	0.000
GERD	6,898 (12%)	6,871 (12%)	0.003	21,088 (9%)	21,212 (9%)	0.002	16,959 (5%)	17,024 (5%)	0.002	10,569 (3%)	10,903 (3%)	0.005
Heart failure	9,320 (16%)	9,141 (16%)	0.010	10,251 (4%)	10,196 (4%)	0.001	4,001 (1%)	3,743 (1%)	0.007	1,606 (0%)	1,565 (0%)	0.002
Hypertension	38,140 (65%)	37,917 (65%)	0.013	96,384 (41%)	96,118 (41%)	0.003	58,596 (17%)	57,241 (17%)	0.008	22,160 (5%)	22,739 (6%)	0.006
Hypothyroidism	7,350 (13%)	7,236 (12%)	0.007	24,054 (10%)	24,260 (10%)	0.003	21,956 (6%)	21,573 (6%)	0.003	17,452 (4%)	17,758 (4%)	0.004
Malignant neoplastic disease	15,402 (26%)	15,230 (26%)	0.009	37,052 (16%)	38,490 (16%)	0.016	19,765 (6%)	19,599 (6%)	0.001	9,413 (2%)	8,865 (2%)	0.009

	Cohort 1			Cohort 2			Cohort 3			Cohort 4		
	**Unvaccinated**	**Vaccinated**	**ASMD**	**Unvaccinated**	**Vaccinated**	**ASMD**	**Unvaccinated**	**Vaccinated**	**ASMD**	**Unvaccinated**	**Vaccinated**	**ASMD**
Myocardial infarction	2,258 (4%)	2,235 (4%)	0.003	5,972 (3%)	5,812 (2%)	0.005	3,615 (1%)	3,448 (1%)	0.004	1,362 (0%)	1,364 (0%)	0.000
Osteoporosis	9,270 (16%)	9,151 (16%)	0.007	18,870 (8%)	19,036 (8%)	0.002	7,126 (2%)	7,039 (2%)	0.001	2,254 (1%)	2,040 (0%)	0.007
Pneumonia	6,643 (11%)	6,563 (11%)	0.005	15,630 (7%)	15,557 (7%)	0.002	14,821 (4%)	14,580 (4%)	0.002	14,171 (3%)	14,083 (3%)	0.001
Rheumatoid arthritis	765 (1%)	727 (1%)	0.006	2,274 (1%)	2,286 (1%)	0.000	1,544 (0%)	1,592 (0%)	0.002	754 (0%)	646 (0%)	0.006
Stroke	5,119 (9%)	5,043 (9%)	0.006	8,750 (4%)	7,706 (3%)	0.024	4,343 (1%)	4,147 (1%)	0.005	1,828 (0%)	1,840 (0%)	0.001
Venous thromboembolism	2,469 (4%)	2,418 (4%)	0.005	4,745 (2%)	4,419 (2%)	0.010	3,365 (1%)	2,982 (1%)	0.011	1,931 (0%)	1,777 (0%)	0.005

The 4 cohorts represent vaccine rollout periods. *Calculated as the days of previous observation in the database before index date. **Assessed anytime before index date. ASMD = Absolute standardized mean difference, GP = General practice, PCR = Polymerase chain reaction, COPD = Chronic obstructive pulmonary disease, GERD = Gastro-Esophageal reflux disease

Mercadé-Besora N, *et al. Heart* 2024; 110:635–643. doi: 10.1136/heartjnl-2023-323483

Table S13: Characteristics of unweighted populations in SIDIAP, database, stratified by staggered cohort and exposure status. Exposure is any COVID-19 vaccine.

	Cohort 1			Cohort 2			Cohort 3			Cohort 4		
	**Unvaccinated**	**Vaccinated**	**ASMD**	**Unvaccinated**	**Vaccinated**	**ASMD**	**Unvaccinated**	**Vaccinated**	**ASMD**	**Unvaccinated**	**Vaccinated**	**ASMD**
**N (individuals)**	223,962	89,941		433,151	819,590		869,497	954,232		1,061,634	880,950	
**Age, median [Q25-Q75]**	85 [82-88]	87 [83-90]	0.253	67 [62-72]	72 [65-78]	0.391	48 [43-57]	54 [49-58]	0.192	38 [28-51]	37 [29-43]	0.246
**Sex: Female, N(%)**	136,888 (61%)	56,235 (63%)	0.029	237,494 (55%)	443,019 (54%)	0.016	409,207 (47%)	462,599 (48%)	0.028	488,499 (46%)	408,555 (46%)	0.007
**Years of prior history*, median [Q25-Q75]**	15 [15-15]	15 [15-15]	0.051	15 [15-15]	15 [15-15]	0.185	15 [15-15]	15 [15-15]	0.246	15 [10-16]	15 [15-16]	0.195
**Number of GP visits, median [Q25-Q75]**	7 [2-13]	10 [5-18]	0.251	3 [0-8]	6 [2-11]	0.168	1 [0-5]	2 [0-7]	0.105	0 [0-4]	2 [0-6]	0.060
**Number of PCR tests, median [Q25-Q75]**	0[0-0]	0[0-1]	0.070	0[0-0]	0[0-1]	0.042	0[0-0]	0[0-1]	0.073	0[0-0]	0[0-1]	0.123
**Comorbidities**, N(%)**												
Anxiety	41,908 (19%)	18,117 (20%)	0.036	97,857 (23%)	181,286 (22%)	0.011	197,922 (23%)	233,123 (24%)	0.039	203,019 (19%)	186,562 (21%)	0.051
Asthma	13,371 (6%)	5,841 (6%)	0.022	19,809 (5%)	41,966 (5%)	0.025	36,811 (4%)	42,221 (4%)	0.009	45,374 (4%)	46,776 (5%)	0.049
Chronic kidney disease	63,041 (28%)	31,132 (35%)	0.140	32,450 (7%)	101,792 (12%)	0.165	22,454 (3%)	20,303 (2%)	0.030	20,368 (2%)	5,314 (1%)	0.118
COPD	24,459 (11%)	11,292 (13%)	0.051	28,780 (7%)	67,795 (8%)	0.062	19,020 (2%)	27,451 (3%)	0.044	15,415 (1%)	5,836 (1%)	0.077
Dementia	16,743 (7%)	10,455 (12%)	0.142	7,404 (2%)	19,153 (2%)	0.045	6,009 (1%)	2,787 (0%)	0.057	5,866 (1%)	1,310 (0%)	0.068
Depressive disorder	38,148 (17%)	18,040 (20%)	0.078	61,308 (14%)	124,929 (15%)	0.031	77,039 (9%)	98,723 (10%)	0.050	66,864 (6%)	49,608 (6%)	0.028
Diabetes	51,964 (23%)	22,968 (26%)	0.054	65,572 (15%)	155,362 (19%)	0.102	60,254 (7%)	78,356 (8%)	0.048	49,331 (5%)	29,768 (3%)	0.065
GERD	25,376 (11%)	10,728 (12%)	0.019	34,715 (8%)	87,626 (11%)	0.092	37,828 (4%)	53,565 (6%)	0.058	31,600 (3%)	23,657 (3%)	0.018
Heart failure	28,538 (13%)	16,202 (18%)	0.147	16,697 (4%)	42,924 (5%)	0.066	11,909 (1%)	10,469 (1%)	0.025	10,738 (1%)	2,776 (0%)	0.086
Hypertension	138,631 (62%)	60,128 (67%)	0.104	157,290 (36%)	390,610 (48%)	0.231	125,239 (14%)	188,390 (20%)	0.142	97,835 (9%)	43,351 (5%)	0.168

	Cohort 1			Cohort 2			Cohort 3			Cohort 4		
	Unvaccinated	Vaccinated	ASMD	Unvaccinated	Vaccinated	ASMD	Unvaccinated	Vaccinated	ASMD	Unvaccinated	Vaccinated	ASMD
Hypothyroidism	26,875 (12%)	11,473 (13%)	0.023	42,245 (10%)	89,496 (11%)	0.038	50,396 (6%)	64,873 (7%)	0.041	49,279 (5%)	38,477 (4%)	0.013
Malignant neoplastic disease	54,714 (24%)	24,173 (27%)	0.056	81,394 (19%)	151,745 (19%)	0.007	48,971 (6%)	59,566 (6%)	0.026	37,755 (4%)	18,021 (2%)	0.092
Myocardial infarction	7,886 (4%)	3,642 (4%)	0.028	9,894 (2%)	22,773 (3%)	0.031	7,570 (1%)	11,483 (1%)	0.033	6,171 (1%)	2,459 (0%)	0.046
Osteoporosis	32,239 (14%)	14,846 (17%)	0.058	29,512 (7%)	81,707 (10%)	0.114	16,393 (2%)	21,679 (2%)	0.027	14,373 (1%)	3,343 (0%)	0.105
Pneumonia	22,254 (10%)	10,959 (12%)	0.072	27,837 (6%)	61,325 (7%)	0.042	34,513 (4%)	44,236 (5%)	0.033	36,413 (3%)	32,657 (4%)	0.015
Rheumatoid arthritis	2,666 (1%)	1,204 (1%)	0.013	3,771 (1%)	8,890 (1%)	0.022	3,239 (0%)	4,770 (0%)	0.019	2,706 (0%)	1,281 (0%)	0.024
Stroke	16,690 (7%)	8,542 (9%)	0.073	14,256 (3%)	32,612 (4%)	0.037	10,877 (1%)	12,068 (1%)	0.001	9,400 (1%)	3,384 (0%)	0.063
Venous thromboembolism	8,014 (4%)	4,126 (5%)	0.051	8,172 (2%)	17,846 (2%)	0.021	7,747 (1%)	8,920 (1%)	0.005	6,632 (1%)	3,768 (0%)	0.027

The 4 cohorts represent vaccine rollout periods. *Calculated as the days of previous observation in the database before index date. **Assessed anytime before index date. ASMD = Absolute standardized mean difference, GP = General practice, PCR = Polymerase chain reaction, COPD = Chronic obstructive pulmonary disease, GERD = Gastro-Esophageal reflux disease

Table S14: Characteristics of weighted populations in SIDIAP, database, stratified by staggered cohort and exposure status. Exposure is BNT162b2 vaccine.

	**Cohort 1**			**Cohort 2**			**Cohort 3**			**Cohort 4**		
	**Unvaccinated**	**Vaccinated**	**ASMD**	**Unvaccinated**	**Vaccinated**	**ASMD**	**Unvaccinated**	**Vaccinated**	**ASMD**	**Unvaccinated**	**Vaccinated**	**ASMD**
**N (individuals)**	57,981	57,843		99,838	99,488		276,241	273,593		312,492	313,346	
**Age, median [Q25-Q75]**	86 [83-89]	86 [82-89]	0.000	74 [71-81]	74 [72-81]	0.006	50 [45-56]	50 [45-56]	0.003	36 [29-43]	36 [29-43]	0.004
**Sex: Female, N(%)**	35,828 (62%)	35,895 (62%)	0.005	58,343 (58%)	57,844 (58%)	0.006	130,375 (47%)	129,701 (47%)	0.004	145,270 (46%)	144,384 (46%)	0.008
**Years of prior history*, median [Q25-Q75]**	15 [15-15]	15 [15-15]	0.002	15 [15-15]	15 [15-15]	0.005	15 [15-15]	15 [15-15]	0.001	15 [13-16]	15 [14-16]	0.004
**Number of GP visits, median [Q25-Q75]**	8 [3-15]	8 [4-15]	0.008	4 [0-10]	6 [2-10]	0.013	0 [0-5]	2 [0-6]	0.000	0 [0-4]	0 [0-5]	0.012
**Number of PCR tests, median [Q25-Q75]**	0[0-0]	0[0-0]	0.006	0[0-0]	0[0-0]	0.009	0[0-0]	0[0-0]	0.005	0[0-0]	0[0-0]	0.010
**Comorbidities**, N(%)**												
Anxiety	11,327 (20%)	11,359 (20%)	0.003	20,281 (20%)	20,181 (20%)	0.001	67,000 (24%)	63,263 (23%)	0.027	65,060 (21%)	64,616 (21%)	0.005
Asthma	3,734 (6%)	3,583 (6%)	0.010	5,213 (5%)	5,179 (5%)	0.001	12,220 (4%)	11,760 (4%)	0.006	15,491 (5%)	15,211 (5%)	0.005
Chronic kidney disease	18,610 (32%)	18,576 (32%)	0.000	15,548 (16%)	15,607 (16%)	0.003	5,583 (2%)	5,399 (2%)	0.003	1,999 (1%)	2,008 (1%)	0.000
COPD	6,980 (12%)	6,907 (12%)	0.003	9,163 (9%)	9,045 (9%)	0.003	6,020 (2%)	6,047 (2%)	0.002	1,998 (1%)	2,000 (1%)	0.000
Dementia	5,678 (10%)	5,771 (10%)	0.006	4,152 (4%)	3,716 (4%)	0.022	1,037 (0%)	980 (0%)	0.003	502 (0%)	506 (0%)	0.000
Depressive disorder	10,920 (19%)	10,923 (19%)	0.001	15,653 (16%)	15,532 (16%)	0.002	26,419 (10%)	24,357 (9%)	0.023	17,262 (6%)	17,507 (6%)	0.003
Diabetes	14,416 (25%)	14,096 (24%)	0.011	20,461 (20%)	20,645 (21%)	0.006	19,302 (7%)	18,543 (7%)	0.008	10,119 (3%)	10,440 (3%)	0.005
GERD	6,784 (12%)	6,826 (12%)	0.003	10,187 (10%)	10,088 (10%)	0.002	12,811 (5%)	12,805 (5%)	0.002	7,750 (2%)	8,224 (3%)	0.009
Heart failure	9,390 (16%)	8,983 (16%)	0.018	7,563 (8%)	7,296 (7%)	0.009	3,044 (1%)	2,699 (1%)	0.011	1,069 (0%)	1,049 (0%)	0.001
Hypertension	37,879 (65%)	37,465 (65%)	0.012	51,262 (51%)	50,608 (51%)	0.010	42,266 (15%)	41,291 (15%)	0.006	15,063 (5%)	15,323 (5%)	0.003
Hypothyroidism	7,310 (13%)	7,166 (12%)	0.007	11,611 (12%)	11,249 (11%)	0.010	16,597 (6%)	16,509 (6%)	0.001	13,155 (4%)	13,617 (4%)	0.007
Malignant neoplastic disease	15,302 (26%)	14,971 (26%)	0.012	19,393 (19%)	18,719 (19%)	0.015	14,311 (5%)	13,981 (5%)	0.003	6,555 (2%)	6,398 (2%)	0.004
Myocardial infarction	2,286 (4%)	2,182 (4%)	0.009	3,130 (3%)	2,924 (3%)	0.011	2,569 (1%)	2,451 (1%)	0.004	875 (0%)	880 (0%)	0.000

Mercadé-Besora N, *et al. Heart* 2024; 110:635–643. doi: 10.1136/heartjnl-2023-323483

	Cohort 1			Cohort 2			Cohort 3			Cohort 4		
	Unvaccinated	Vaccinated	ASMD	Unvaccinated	Vaccinated	ASMD	Unvaccinated	Vaccinated	ASMD	Unvaccinated	Vaccinated	ASMD
Osteoporosis	9,170 (16%)	9,085 (16%)	0.003	11,182 (11%)	11,201 (11%)	0.002	4,764 (2%)	4,639 (2%)	0.002	1,344 (0%)	1,306 (0%)	0.002
Pneumonia	6,588 (11%)	6,476 (11%)	0.005	7,945 (8%)	7,918 (8%)	0.000	11,759 (4%)	11,273 (4%)	0.007	10,730 (3%)	10,798 (3%)	0.001
Rheumatoid arthritis	755 (1%)	718 (1%)	0.006	1,064 (1%)	1,111 (1%)	0.005	1,113 (0%)	1,026 (0%)	0.004	536 (0%)	461 (0%)	0.006
Stroke	5,070 (9%)	5,006 (9%)	0.003	5,363 (5%)	5,086 (5%)	0.012	3,121 (1%)	3,043 (1%)	0.002	1,199 (0%)	1,253 (0%)	0.003
Venous thromboembolism	2,452 (4%)	2,378 (4%)	0.006	2,660 (3%)	2,526 (3%)	0.008	2,527 (1%)	2,211 (1%)	0.012	1,394 (0%)	1,327 (0%)	0.003

The 4 cohorts represent vaccine rollout periods. *Calculated as the days of previous observation in the database before index date. **Assessed anytime before index date. ASMD = Absolute standardized mean difference, GP = General practice, PCR = Polymerase chain reaction, COPD = Chronic obstructive pulmonary disease, GERD = Gastro-Esophageal reflux disease

Table S15: Characteristics of unweighted populations in SIDIAP, database, stratified by staggered cohort and exposure status. Exposure is BNT162b2 vaccine.

	Cohort 1			Cohort 2			Cohort 3			Cohort 4		
	**Unvaccinated**	**Vaccinated**	**ASMD**	**Unvaccinated**	**Vaccinated**	**ASMD**	**Unvaccinated**	**Vaccinated**	**ASMD**	**Unvaccinated**	**Vaccinated**	**ASMD**
**N (individuals)**	223,960	88,896		433,111	445,581		869,109	706,435		1,068,043	580,329	
**Age, median [Q25-Q75]**	85 [82-88]	87 [84-90]	0.258	67 [62-72]	78 [74-83]	0.943	48 [43-57]	53 [48-57]	0.127	38 [28-51]	38 [31-43]	0.231
**Sex: Female, N(%)**	136,891 (61%)	55,704 (63%)	0.032	237,440 (55%)	254,378 (57%)	0.046	409,076 (47%)	341,289 (48%)	0.025	491,528 (46%)	274,150 (47%)	0.024
**Years of prior history*, median [Q25-Q75]**	15 [15-15]	15 [15-15]	0.051	15 [15-15]	15 [15-15]	0.206	15 [15-15]	15 [15-15]	0.244	15 [10-16]	15 [15-16]	0.211
**Number of GP visits, median [Q25-Q75]**	7 [2-13]	10 [5-18]	0.253	3 [0-8]	7 [3-12]	0.247	1 [0-5]	2 [0-7]	0.093	0 [0-4]	2 [0-6]	0.059
**Number of PCR tests, median [Q25-Q75]**	0[0-0]	0[0-1]	0.067	0[0-0]	0[0-1]	0.019	0[0-0]	0[0-1]	0.074	0[0-0]	0[0-1]	0.121
**Comorbidities**, N(%)**												
Anxiety	41,914 (19%)	17,962 (20%)	0.038	97,901 (23%)	91,620 (21%)	0.050	197,933 (23%)	172,434 (24%)	0.039	204,108 (19%)	125,466 (22%)	0.062
Asthma	13,370 (6%)	5,796 (7%)	0.023	19,824 (5%)	25,291 (6%)	0.050	36,788 (4%)	31,586 (4%)	0.012	45,747 (4%)	30,865 (5%)	0.048
Chronic kidney disease	63,046 (28%)	30,745 (35%)	0.139	32,447 (7%)	83,968 (19%)	0.341	22,494 (3%)	13,920 (2%)	0.041	20,416 (2%)	3,171 (1%)	0.124
COPD	24,471 (11%)	11,169 (13%)	0.051	28,773 (7%)	44,207 (10%)	0.119	19,028 (2%)	17,815 (3%)	0.022	15,424 (1%)	3,218 (1%)	0.090
Dementia	16,748 (7%)	10,393 (12%)	0.143	7,389 (2%)	17,606 (4%)	0.136	6,003 (1%)	2,033 (0%)	0.058	5,878 (1%)	856 (0%)	0.068
Depressive disorder	38,161 (17%)	17,894 (20%)	0.079	61,309 (14%)	72,447 (16%)	0.059	77,050 (9%)	69,517 (10%)	0.033	67,070 (6%)	32,476 (6%)	0.029
Diabetes	51,948 (23%)	22,682 (26%)	0.054	65,583 (15%)	99,692 (22%)	0.186	60,242 (7%)	53,330 (8%)	0.024	49,433 (5%)	19,502 (3%)	0.065
GERD	25,370 (11%)	10,611 (12%)	0.019	34,704 (8%)	53,110 (12%)	0.131	37,842 (4%)	37,707 (5%)	0.046	31,662 (3%)	15,544 (3%)	0.017
Heart failure	28,525 (13%)	16,041 (18%)	0.147	16,686 (4%)	34,932 (8%)	0.171	11,920 (1%)	7,057 (1%)	0.034	10,734 (1%)	1,642 (0%)	0.090
Hypertension	138,655 (62%)	59,414 (67%)	0.103	157,291 (36%)	252,365 (57%)	0.416	125,235 (14%)	128,859 (18%)	0.104	97,944 (9%)	26,383 (5%)	0.184

Mercadé-Besora N, *et al. Heart* 2024; 110:635–643. doi: 10.1136/heartjnl-2023-323483

	Cohort 1			Cohort 2			Cohort 3			Cohort 4		
	Unvaccinated	Vaccinated	ASMD	Unvaccinated	Vaccinated	ASMD	Unvaccinated	Vaccinated	ASMD	Unvaccinated	Vaccinated	ASMD
Hypothyroidism	26,868 (12%)	11,363 (13%)	0.024	42,216 (10%)	53,488 (12%)	0.073	50,384 (6%)	46,214 (7%)	0.031	49,424 (5%)	25,961 (4%)	0.007
Malignant neoplastic disease	54,729 (24%)	23,784 (27%)	0.053	81,399 (19%)	96,204 (22%)	0.070	48,967 (6%)	40,510 (6%)	0.004	37,874 (4%)	11,535 (2%)	0.095
Myocardial infarction	7,890 (4%)	3,590 (4%)	0.027	9,892 (2%)	14,140 (3%)	0.055	7,573 (1%)	7,753 (1%)	0.023	6,182 (1%)	1,405 (0%)	0.053
Osteoporosis	32,250 (14%)	14,701 (17%)	0.059	29,528 (7%)	56,885 (13%)	0.201	16,385 (2%)	14,126 (2%)	0.008	14,380 (1%)	1,876 (0%)	0.113
Pneumonia	22,249 (10%)	10,811 (12%)	0.071	27,831 (6%)	38,127 (9%)	0.081	34,511 (4%)	31,972 (5%)	0.028	36,645 (3%)	21,355 (4%)	0.013
Rheumatoid arthritis	2,666 (1%)	1,189 (1%)	0.013	3,769 (1%)	5,143 (1%)	0.028	3,242 (0%)	2,843 (0%)	0.005	2,700 (0%)	812 (0%)	0.026
Stroke	16,693 (7%)	8,475 (10%)	0.075	14,244 (3%)	24,307 (5%)	0.106	10,868 (1%)	8,114 (1%)	0.009	9,394 (1%)	2,033 (0%)	0.068
Venous thromboembolism	8,023 (4%)	4,072 (5%)	0.050	8,162 (2%)	12,564 (3%)	0.062	7,738 (1%)	6,066 (1%)	0.003	6,658 (1%)	2,434 (0%)	0.028

The 4 cohorts represent vaccine rollout periods. *Calculated as the days of previous observation in the database before index date. **Assessed anytime before index date. ASMD = Absolute standardized mean difference, GP = General practice, PCR = Polymerase chain reaction, COPD = Chronic obstructive pulmonary disease, GERD = Gastro-Esophageal reflux disease

Table S16: Characteristics of weighted populations in SIDIAP, database, stratified by staggered cohort and exposure status. Exposure is ChAdOx1 vaccine.

	Cohort 2			Cohort 3		
	**Unvaccinated**	**Vaccinated**	**ASMD**	**Unvaccinated**	**Vaccinated**	**ASMD**
**N (individuals)**	120,307	120,802		44,033	43,901	
**Age, median [Q25-Q75]**	64 [61-66]	64 [61-66]	0.000	64 [61-66]	64 [61-67]	0.002
**Sex: Female, N(%)**	62,434 (52%)	62,425 (52%)	0.004	23,009 (52%)	22,980 (52%)	0.002
**Years of prior history*, median [Q25-Q75]**	15 [15-15]	15 [15-15]	0.003	15 [15-15]	15 [15-15]	0.005
**Number of GP visits, median [Q25-Q75]**	2 [0-7]	4 [1-8]	0.017	2 [0-7]	2 [0-7]	0.003
**Number of PCR tests, median [Q25-Q75]**	0[0-0]	0[0-0]	0.013	0[0-0]	0[0-0]	0.005
**Comorbidities**, N(%)**						
Anxiety	28,213 (23%)	27,989 (23%)	0.007	10,333 (23%)	9,717 (22%)	0.032
Asthma	4,909 (4%)	4,785 (4%)	0.006	1,664 (4%)	1,639 (4%)	0.002
Chronic kidney disease	4,390 (4%)	4,526 (4%)	0.005	1,556 (4%)	1,597 (4%)	0.006
COPD	6,702 (6%)	6,846 (6%)	0.004	2,405 (5%)	2,350 (5%)	0.005
Dementia	470 (0%)	397 (0%)	0.010	170 (0%)	171 (0%)	0.000
Depressive disorder	16,483 (14%)	16,258 (13%)	0.007	5,940 (13%)	5,813 (13%)	0.007
Diabetes	16,127 (13%)	16,418 (14%)	0.005	5,698 (13%)	5,705 (13%)	0.002
GERD	9,580 (8%)	9,569 (8%)	0.002	3,206 (7%)	3,216 (7%)	0.002
Heart failure	2,268 (2%)	2,236 (2%)	0.003	844 (2%)	847 (2%)	0.001
Hypertension	39,422 (33%)	39,551 (33%)	0.001	13,589 (31%)	13,737 (31%)	0.009
Hypothyroidism	10,965 (9%)	11,173 (9%)	0.005	4,002 (9%)	3,830 (9%)	0.013
Malignant neoplastic disease	12,621 (10%)	12,418 (10%)	0.007	4,304 (10%)	3,910 (9%)	0.030
Myocardial infarction	2,553 (2%)	2,500 (2%)	0.004	888 (2%)	868 (2%)	0.003
Osteoporosis	6,687 (6%)	6,679 (6%)	0.001	2,155 (5%)	2,200 (5%)	0.005
Pneumonia	6,452 (5%)	6,392 (5%)	0.003	2,243 (5%)	2,167 (5%)	0.007
Rheumatoid arthritis	1,009 (1%)	931 (1%)	0.008	337 (1%)	259 (1%)	0.021
Stroke	2,693 (2%)	2,582 (2%)	0.007	965 (2%)	1,000 (2%)	0.006
Venous thromboembolism	1,686 (1%)	1,469 (1%)	0.016	659 (1%)	541 (1%)	0.023

The 4 cohorts represent vaccine rollout periods. *Calculated as the days of previous observation in the database before index date. **Assessed anytime before index date. ASMD = Absolute standardized mean difference, GP = General practice, PCR = Polymerase chain reaction, COPD = Chronic obstructive pulmonary disease, GERD = Gastro-Esophageal reflux disease

Table S17: Characteristics of unweighted populations in SIDIAP, database, stratified by staggered cohort and exposure status. Exposure is ChAdOx1 vaccine.

	Cohort 2			Cohort 3		
	**Unvaccinated**	**Vaccinated**	**ASMD**	**Unvaccinated**	**Vaccinated**	**ASMD**
**N (individuals)**	433,636	323,204		873,400	84,204	
**Age, median [Q25-Q75]**	67 [62-72]	64 [62-67]	0.241	48 [43-57]	64 [61-67]	0.986
**Sex: Female, N(%)**	237,722 (55%)	162,269 (50%)	0.093	411,107 (47%)	44,374 (53%)	0.113
**Years of prior history*, median [Q25-Q75]**	15 [15-15]	15 [15-15]	0.158	15 [15-15]	15 [15-15]	0.281
**Number of GP visits, median [Q25-Q75]**	3 [0-8]	4 [1-9]	0.037	1 [0-5]	4 [1-8]	0.182
**Number of PCR tests, median [Q25-Q75]**	0[0-0]	0[0-1]	0.047	0[0-0]	0[0-0]	0.029
**Comorbidities**, N(%)**						
Anxiety	97,950 (23%)	77,793 (24%)	0.035	198,282 (23%)	19,626 (23%)	0.014
Asthma	19,861 (5%)	13,894 (4%)	0.014	36,915 (4%)	3,283 (4%)	0.017
Chronic kidney disease	32,497 (7%)	12,206 (4%)	0.162	22,570 (3%)	3,199 (4%)	0.069
COPD	28,826 (7%)	18,658 (6%)	0.036	19,103 (2%)	4,996 (6%)	0.191
Dementia	7,407 (2%)	1,009 (0%)	0.140	6,041 (1%)	345 (0%)	0.038
Depressive disorder	61,377 (14%)	44,463 (14%)	0.011	77,249 (9%)	11,874 (14%)	0.166
Diabetes	65,666 (15%)	45,453 (14%)	0.031	60,383 (7%)	12,142 (14%)	0.245
GERD	34,755 (8%)	28,610 (9%)	0.030	37,840 (4%)	6,754 (8%)	0.154
Heart failure	16,741 (4%)	5,636 (2%)	0.129	11,981 (1%)	1,703 (2%)	0.050
Hypertension	157,484 (36%)	114,179 (35%)	0.021	125,527 (14%)	28,314 (34%)	0.463
Hypothyroidism	42,257 (10%)	30,350 (9%)	0.012	50,476 (6%)	7,763 (9%)	0.131
Malignant neoplastic disease	81,510 (19%)	36,352 (11%)	0.212	49,066 (6%)	7,842 (9%)	0.141
Myocardial infarction	9,910 (2%)	7,242 (2%)	0.003	7,561 (1%)	1,784 (2%)	0.103
Osteoporosis	29,546 (7%)	19,864 (6%)	0.027	16,434 (2%)	4,382 (5%)	0.180
Pneumonia	27,865 (6%)	18,329 (6%)	0.032	34,617 (4%)	4,445 (5%)	0.063
Rheumatoid arthritis	3,762 (1%)	2,557 (1%)	0.008	3,246 (0%)	535 (1%)	0.037
Stroke	14,284 (3%)	6,369 (2%)	0.083	10,884 (1%)	1,915 (2%)	0.078
Venous thromboembolism	8,163 (2%)	3,613 (1%)	0.063	7,746 (1%)	1,110 (1%)	0.041

The 4 cohorts represent vaccine rollout periods. *Calculated as the days of previous observation in the database before index date. **Assessed anytime before index date. ASMD = Absolute standardized mean difference, GP = General practice, PCR = Polymerase chain reaction, COPD = Chronic obstructive pulmonary disease, GERD = Gastro-Esophageal reflux disease

Table S18: Characteristics of weighted populations in CORIVA, database, stratified by staggered cohort and exposure status. Exposure is any COVID-19 vaccine.

	Cohort 1			Cohort 2			Cohort 3			Cohort 4		
	**Unvaccinated**	**Vaccinated**	**ASMD**	**Unvaccinated**	**Vaccinated**	**ASMD**	**Unvaccinated**	**Vaccinated**	**ASMD**	**Unvaccinated**	**Vaccinated**	**ASMD**
**N (individuals)**	10,630	10,630		3,863	3,863		17,929	17,929		18,740	18,740	
**Age, median [Q25-Q75]**	78 [72-83]	78 [72-83]	0.000	70 [65-75]	70 [65-75]	0.000	52 [45-59]	50 [45-59]	0.000	40 [30-51]	40 [30-51]	0.000
**Sex: Female, N(%)**	7,165 (67%)	7,165 (67%)	0.000	2,439 (63%)	2,439 (63%)	0.000	9,054 (50%)	9,054 (50%)	0.000	8,493 (45%)	8,493 (45%)	0.000
**Years of prior history*, median [Q25-Q75]**	4 [4-4]	4 [4-4]	0.000	4 [4-4]	4 [4-4]	0.000	4 [4-4]	4 [4-4]	0.000	5 [4-5]	5 [4-5]	0.000
**Number of GP visits, median [Q25-Q75]**	14 [4-24]	14 [6-23]	0.004	12 [3-23]	12 [5-22]	0.000	2 [0-11]	4 [1-10]	0.002	2 [0-8]	2 [0-8]	0.005
**Number of PCR tests, median [Q25-Q75]**	0[0-0]	0[0-0]	0.003	0[0-0]	0[0-0]	0.002	0[0-0]	0[0-0]	0.000	0[0-0]	0[0-0]	0.002
**Comorbidities**, N(%)**												
Anxiety	1,060 (10%)	1,076 (10%)	0.005	405 (10%)	415 (11%)	0.008	1,545 (9%)	1,500 (8%)	0.009	1,552 (8%)	1,495 (8%)	0.011
Asthma	996 (9%)	917 (9%)	0.026	372 (10%)	365 (9%)	0.006	936 (5%)	894 (5%)	0.011	845 (5%)	782 (4%)	0.016
Chronic kidney disease	1,015 (10%)	999 (9%)	0.005	336 (9%)	309 (8%)	0.025	347 (2%)	339 (2%)	0.003	212 (1%)	194 (1%)	0.009
COPD	757 (7%)	723 (7%)	0.012	264 (7%)	258 (7%)	0.006	442 (2%)	417 (2%)	0.009	318 (2%)	249 (1%)	0.030
Dementia	438 (4%)	412 (4%)	0.013	106 (3%)	90 (2%)	0.027	120 (1%)	131 (1%)	0.008	71 (0%)	70 (0%)	0.001
Depressive disorder	1,076 (10%)	1,083 (10%)	0.002	462 (12%)	433 (11%)	0.023	1,787 (10%)	1,764 (10%)	0.004	1,679 (9%)	1,739 (9%)	0.011
Diabetes	1,861 (18%)	1,833 (17%)	0.007	640 (17%)	588 (15%)	0.037	1,012 (6%)	963 (5%)	0.012	814 (4%)	764 (4%)	0.014
GERD	1,416 (13%)	1,458 (14%)	0.011	523 (14%)	531 (14%)	0.006	1,562 (9%)	1,600 (9%)	0.007	1,166 (6%)	1,172 (6%)	0.001
Heart failure	3,898 (37%)	3,839 (36%)	0.011	1,003 (26%)	970 (25%)	0.020	1,348 (8%)	1,324 (7%)	0.005	781 (4%)	737 (4%)	0.012
Hypertension	8,016 (75%)	8,221 (77%)	0.045	2,569 (67%)	2,641 (68%)	0.040	5,521 (31%)	5,643 (31%)	0.015	3,359 (18%)	3,538 (19%)	0.025
Hypothyroidism	1,218 (11%)	1,160 (11%)	0.017	419 (11%)	428 (11%)	0.007	981 (5%)	948 (5%)	0.008	654 (3%)	675 (4%)	0.006
Malignant neoplastic disease	1,655 (16%)	1,732 (16%)	0.020	640 (17%)	659 (17%)	0.013	771 (4%)	782 (4%)	0.003	455 (2%)	453 (2%)	0.001
Myocardial infarction	269 (3%)	264 (2%)	0.003	86 (2%)	71 (2%)	0.026	127 (1%)	116 (1%)	0.008	81 (0%)	75 (0%)	0.005
Osteoporosis	675 (6%)	685 (6%)	0.004	187 (5%)	176 (5%)	0.013	233 (1%)	230 (1%)	0.001	131 (1%)	146 (1%)	0.009
Pneumonia	771 (7%)	768 (7%)	0.001	249 (6%)	244 (6%)	0.005	657 (4%)	670 (4%)	0.004	567 (3%)	562 (3%)	0.002
Rheumatoid arthritis	268 (3%)	273 (3%)	0.003	139 (4%)	122 (3%)	0.024	209 (1%)	187 (1%)	0.012	130 (1%)	116 (1%)	0.009

Mercadé-Besora N, *et al. Heart* 2024; 110:635–643. doi: 10.1136/heartjnl-2023-323483

	Cohort 1			Cohort 2			Cohort 3			Cohort 4		
	Unvaccinated	Vaccinated	ASMD	Unvaccinated	Vaccinated	ASMD	Unvaccinated	Vaccinated	ASMD	Unvaccinated	Vaccinated	ASMD
Stroke	460 (4%)	461 (4%)	0.001	147 (4%)	144 (4%)	0.004	179 (1%)	189 (1%)	0.006	112 (1%)	116 (1%)	0.003
Venous thromboembolism	469 (4%)	472 (4%)	0.001	171 (4%)	123 (3%)	0.064	306 (2%)	301 (2%)	0.002	219 (1%)	201 (1%)	0.009

The 4 cohorts represent vaccine rollout periods. *Calculated as the days of previous observation in the database before index date. **Assessed anytime before index date. ASMD = Absolute standardized mean difference, GP = General practice, PCR = Polymerase chain reaction, COPD = Chronic obstructive pulmonary disease, GERD = Gastro-Esophageal reflux disease

Mercadé-Besora N, *et al. Heart* 2024; 110:635–643. doi: 10.1136/heartjnl-2023-323483

Table S19: Characteristics of unweighted populations in CORIVA, database, stratified by staggered cohort and exposure status. Exposure is any COVID-19 vaccine.

	Cohort 1			Cohort 2			Cohort 3			Cohort 4		
	**Unvaccinated**	**Vaccinated**	**ASMD**	**Unvaccinated**	**Vaccinated**	**ASMD**	**Unvaccinated**	**Vaccinated**	**ASMD**	**Unvaccinated**	**Vaccinated**	**ASMD**
**N (individuals)**	23,982	26,736		34,317	4,572		96,423	24,050		147,545	22,245	
**Age, median [Q25-Q75]**	79 [73-85]	77 [73-82]	0.127	72 [67-81]	70 [66-75]	0.177	54 [46-66]	51 [45-59]	0.227	42 [32-58]	40 [30-51]	0.186
**Sex: Female, N(%)**	16,532 (69%)	17,342 (65%)	0.087	21,862 (64%)	2,891 (63%)	0.010	48,984 (51%)	12,233 (51%)	0.001	72,057 (49%)	10,026 (45%)	0.076
**Years of prior history*, median [Q25-Q75]**	4 [4-4]	4 [4-4]	0.019	4 [4-4]	4 [4-4]	0.002	4 [4-4]	4 [4-4]	0.002	5 [4-5]	5 [4-5]	0.003
**Number of GP visits, median [Q25-Q75]**	11 [1-22]	16 [8-26]	0.216	9 [1-20]	13 [6-23]	0.147	2 [0-10]	5 [1-11]	0.088	1 [0-6]	3 [1-8]	0.106
**Number of PCR tests, median [Q25-Q75]**	0[0-0]	0[0-0]	0.029	0[0-0]	0[0-0]	0.027	0[0-0]	0[0-0]	0.008	0[0-0]	0[0-0]	0.035
**Comorbidities**, N(%)**												
Anxiety	1,966 (8%)	3,238 (12%)	0.130	2,817 (8%)	513 (11%)	0.102	6,406 (7%)	2,162 (9%)	0.087	9,287 (6%)	1,847 (8%)	0.077
Asthma	1,986 (8%)	2,668 (10%)	0.059	2,653 (8%)	447 (10%)	0.072	4,385 (5%)	1,250 (5%)	0.030	5,405 (4%)	968 (4%)	0.035
Chronic kidney disease	2,270 (9%)	2,507 (9%)	0.003	2,939 (9%)	367 (8%)	0.019	2,523 (3%)	415 (2%)	0.061	2,335 (2%)	221 (1%)	0.052
COPD	1,569 (7%)	1,976 (7%)	0.033	2,015 (6%)	312 (7%)	0.039	2,743 (3%)	529 (2%)	0.041	2,558 (2%)	294 (1%)	0.034
Dementia	1,152 (5%)	819 (3%)	0.090	1,297 (4%)	100 (2%)	0.094	1,147 (1%)	151 (1%)	0.059	1,067 (1%)	76 (0%)	0.052
Depressive disorder	2,066 (9%)	3,233 (12%)	0.114	3,189 (9%)	535 (12%)	0.079	7,286 (8%)	2,561 (11%)	0.108	10,034 (7%)	2,152 (10%)	0.105
Diabetes	3,971 (17%)	4,734 (18%)	0.030	4,818 (14%)	712 (16%)	0.043	6,065 (6%)	1,228 (5%)	0.051	6,594 (4%)	901 (4%)	0.021
GERD	2,533 (11%)	4,585 (17%)	0.192	3,364 (10%)	667 (15%)	0.147	6,287 (7%)	2,322 (10%)	0.115	7,455 (5%)	1,427 (6%)	0.059
Heart failure	8,724 (36%)	9,642 (36%)	0.007	9,570 (28%)	1,139 (25%)	0.068	9,982 (10%)	1,607 (7%)	0.132	9,182 (6%)	828 (4%)	0.115
Hypertension	16,651 (69%)	21,677 (81%)	0.272	20,654 (60%)	3,167 (69%)	0.191	29,974 (31%)	7,555 (31%)	0.007	27,992 (19%)	4,172 (19%)	0.006
Hypothyroidism	2,496 (10%)	3,094 (12%)	0.037	3,198 (9%)	515 (11%)	0.064	4,725 (5%)	1,309 (5%)	0.025	5,180 (4%)	799 (4%)	0.004
Malignant neoplastic disease	3,194 (13%)	5,044 (19%)	0.151	5,061 (15%)	782 (17%)	0.064	4,587 (5%)	1,011 (4%)	0.027	4,134 (3%)	523 (2%)	0.028
Myocardial infarction	548 (2%)	743 (3%)	0.031	696 (2%)	86 (2%)	0.011	840 (1%)	146 (1%)	0.031	777 (1%)	87 (0%)	0.020
Osteoporosis	1,273 (5%)	2,015 (8%)	0.091	1,446 (4%)	212 (5%)	0.021	1,545 (2%)	289 (1%)	0.034	1,408 (1%)	164 (1%)	0.024
Pneumonia	1,611 (7%)	2,046 (8%)	0.036	2,081 (6%)	291 (6%)	0.012	3,342 (3%)	898 (4%)	0.014	3,919 (3%)	683 (3%)	0.025

	Cohort 1			Cohort 2			Cohort 3			Cohort 4		
	**Unvaccinated**	**Vaccinated**	**ASMD**	**Unvaccinated**	**Vaccinated**	**ASMD**	**Unvaccinated**	**Vaccinated**	**ASMD**	**Unvaccinated**	**Vaccinated**	**ASMD**
Rheumatoid arthritis	493 (2%)	817 (3%)	0.063	891 (3%)	153 (3%)	0.044	1,022 (1%)	250 (1%)	0.002	987 (1%)	137 (1%)	0.007
Stroke	1,137 (5%)	1,011 (4%)	0.048	1,450 (4%)	169 (4%)	0.027	1,359 (1%)	232 (1%)	0.041	1,256 (1%)	133 (1%)	0.030
Venous thromboembolism	1,058 (4%)	1,207 (5%)	0.005	1,349 (4%)	149 (3%)	0.036	1,808 (2%)	387 (2%)	0.020	1,788 (1%)	235 (1%)	0.015

The 4 cohorts represent vaccine rollout periods. *Calculated as the days of previous observation in the database before index date. **Assessed anytime before index date. ASMD = Absolute standardized mean difference, GP = General practice, PCR = Polymerase chain reaction, COPD = Chronic obstructive pulmonary disease, GERD = Gastro-Esophageal reflux disease

Mercadé-Besora N, *et al. Heart* 2024; 110:635–643. doi: 10.1136/heartjnl-2023-323483

Table S20: Characteristics of weighted populations in CORIVA, database, stratified by staggered cohort and exposure status. Exposure is BNT162b2 vaccine.

	Cohort 1			Cohort 2			Cohort 3			Cohort 4		
	Unvaccinated	Vaccinated	ASMD	Unvaccinated	Vaccinated	ASMD	Unvaccinated	Vaccinated	ASMD	Unvaccinated	Vaccinated	ASMD
**N (individuals)**	9,118	9,118		3,465	3,465		14,414	14,414		13,759	13,759	
**Age, median [Q25-Q75]**	78 [73-83]	78 [73-83]	0.000	70 [65-75]	70 [65-75]	0.000	50 [45-59]	50 [45-59]	0.000	38 [30-50]	38 [30-50]	0.000
**Sex: Female, N(%)**	6,206 (68%)	6,206 (68%)	0.000	2,197 (63%)	2,197 (63%)	0.000	7,324 (51%)	7,324 (51%)	0.000	6,552 (48%)	6,552 (48%)	0.000
**Years of prior history*, median [Q25-Q75]**	4 [4-4]	4 [4-4]	0.000	4 [4-4]	4 [4-4]	0.000	4 [4-4]	4 [4-4]	0.000	5 [4-5]	5 [4-5]	0.000
**Number of GP visits, median [Q25-Q75]**	14 [4-25]	14 [7-24]	0.004	12 [3-23]	12 [5-22]	0.000	2 [0-10]	4 [1-10]	0.002	2 [0-8]	2 [0-8]	0.004
**Number of PCR tests, median [Q25-Q75]**	0[0-0]	0[0-0]	0.002	0[0-0]	0[0-0]	0.003	0[0-0]	0[0-0]	0.000	0[0-0]	0[0-0]	0.001
**Comorbidities**, N(%)**												
Anxiety	935 (10%)	956 (10%)	0.007	367 (11%)	373 (11%)	0.006	1,284 (9%)	1,278 (9%)	0.001	1,206 (9%)	1,170 (9%)	0.009
Asthma	891 (10%)	822 (9%)	0.026	340 (10%)	333 (10%)	0.007	747 (5%)	718 (5%)	0.009	644 (5%)	615 (4%)	0.010
Chronic kidney disease	939 (10%)	905 (10%)	0.012	302 (9%)	278 (8%)	0.025	225 (2%)	220 (2%)	0.003	144 (1%)	132 (1%)	0.008
COPD	666 (7%)	639 (7%)	0.011	238 (7%)	227 (7%)	0.013	333 (2%)	316 (2%)	0.008	223 (2%)	171 (1%)	0.032
Dementia	403 (4%)	377 (4%)	0.014	91 (3%)	81 (2%)	0.018	75 (1%)	72 (0%)	0.002	46 (0%)	38 (0%)	0.012
Depressive disorder	960 (11%)	966 (11%)	0.002	420 (12%)	411 (12%)	0.008	1,472 (10%)	1,485 (10%)	0.003	1,311 (10%)	1,367 (10%)	0.014
Diabetes	1,647 (18%)	1,624 (18%)	0.007	576 (17%)	531 (15%)	0.036	747 (5%)	731 (5%)	0.005	596 (4%)	565 (4%)	0.011
GERD	1,240 (14%)	1,268 (14%)	0.009	476 (14%)	473 (14%)	0.003	1,283 (9%)	1,322 (9%)	0.009	892 (6%)	900 (7%)	0.002
Heart failure	3,527 (39%)	3,470 (38%)	0.013	911 (26%)	877 (25%)	0.022	920 (6%)	899 (6%)	0.006	519 (4%)	484 (4%)	0.014
Hypertension	7,040 (77%)	7,234 (79%)	0.051	2,327 (67%)	2,384 (69%)	0.036	4,296 (30%)	4,340 (30%)	0.007	2,391 (17%)	2,479 (18%)	0.017
Hypothyroidism	1,080 (12%)	1,035 (11%)	0.015	376 (11%)	388 (11%)	0.011	770 (5%)	743 (5%)	0.009	500 (4%)	503 (4%)	0.001
Malignant neoplastic disease	1,485 (16%)	1,565 (17%)	0.023	579 (17%)	594 (17%)	0.012	582 (4%)	606 (4%)	0.008	324 (2%)	317 (2%)	0.003
Myocardial infarction	248 (3%)	234 (3%)	0.010	78 (2%)	68 (2%)	0.020	97 (1%)	88 (1%)	0.008	52 (0%)	52 (0%)	0.000
Osteoporosis	615 (7%)	632 (7%)	0.007	170 (5%)	167 (5%)	0.003	167 (1%)	161 (1%)	0.004	97 (1%)	107 (1%)	0.008
Pneumonia	698 (8%)	696 (8%)	0.001	222 (6%)	222 (6%)	0.001	543 (4%)	505 (4%)	0.014	418 (3%)	429 (3%)	0.005
Rheumatoid arthritis	240 (3%)	239 (3%)	0.001	128 (4%)	110 (3%)	0.028	167 (1%)	148 (1%)	0.013	97 (1%)	88 (1%)	0.008
Stroke	419 (5%)	417 (5%)	0.001	134 (4%)	131 (4%)	0.004	121 (1%)	130 (1%)	0.007	74 (1%)	82 (1%)	0.008

Mercadé-Besora N, *et al. Heart* 2024; 110:635–643. doi: 10.1136/heartjnl-2023-323483

	Cohort 1			Cohort 2			Cohort 3			Cohort 4		
	**Unvaccinated**	**Vaccinated**	**ASMD**	**Unvaccinated**	**Vaccinated**	**ASMD**	**Unvaccinated**	**Vaccinated**	**ASMD**	**Unvaccinated**	**Vaccinated**	**ASMD**
Venous thromboembolism	438 (5%)	416 (5%)	0.011	153 (4%)	117 (3%)	0.053	220 (2%)	214 (1%)	0.003	159 (1%)	138 (1%)	0.015

The 4 cohorts represent vaccine rollout periods. *Calculated as the days of previous observation in the database before index date. **Assessed anytime before index date. ASMD = Absolute standardized mean difference, GP = General practice, PCR = Polymerase chain reaction, COPD = Chronic obstructive pulmonary disease, GERD = Gastro-Esophageal reflux disease

Mercadé-Besora N, *et al. Heart* 2024; 110:635–643. doi: 10.1136/heartjnl-2023-323483

Table S21: Characteristics of unweighted populations in CORIVA, database, stratified by staggered cohort and exposure status. Exposure is BNT162b2 vaccine.

	Cohort 1			Cohort 2			Cohort 3			Cohort 4		
	**Unvaccinated**	**Vaccinated**	**ASMD**	**Unvaccinated**	**Vaccinated**	**ASMD**	**Unvaccinated**	**Vaccinated**	**ASMD**	**Unvaccinated**	**Vaccinated**	**ASMD**
**N (individuals)**	24,073	19,686		34,320	4,067		96,471	18,645		147,553	15,683	
**Age, median [Q25-Q75]**	79 [73-85]	79 [74-83]	0.040	72 [67-81]	70 [66-75]	0.174	54 [46-66]	51 [45-59]	0.257	42 [32-58]	38 [30-50]	0.216
**Sex: Female, N(%)**	16,582 (69%)	13,022 (66%)	0.058	21,867 (64%)	2,582 (63%)	0.005	49,023 (51%)	9,570 (51%)	0.010	72,056 (49%)	7,500 (48%)	0.020
**Years of prior history*, median [Q25-Q75]**	4 [4-4]	4 [4-4]	0.026	4 [4-4]	4 [4-4]	0.000	4 [4-4]	4 [4-4]	0.000	5 [4-5]	5 [4-5]	0.003
**Number of GP visits, median [Q25-Q75]**	11 [1-22]	17 [9-27]	0.252	9 [1-20]	13 [6-23]	0.150	2 [0-10]	5 [1-11]	0.077	1 [0-6]	4 [1-9]	0.116
**Number of PCR tests, median [Q25-Q75]**	0[0-0]	0[0-0]	0.021	0[0-0]	0[0-0]	0.038	0[0-0]	0[0-0]	0.020	0[0-0]	0[0-0]	0.037
**Comorbidities**, N(%)**												
Anxiety	1,966 (8%)	2,429 (12%)	0.138	2,816 (8%)	458 (11%)	0.103	6,416 (7%)	1,780 (10%)	0.106	9,291 (6%)	1,384 (9%)	0.096
Asthma	2,004 (8%)	2,029 (10%)	0.068	2,653 (8%)	404 (10%)	0.078	4,393 (5%)	969 (5%)	0.030	5,403 (4%)	730 (5%)	0.050
Chronic kidney disease	2,284 (9%)	2,041 (10%)	0.029	2,943 (9%)	326 (8%)	0.020	2,537 (3%)	263 (1%)	0.087	2,339 (2%)	146 (1%)	0.059
COPD	1,587 (7%)	1,501 (8%)	0.040	2,013 (6%)	274 (7%)	0.036	2,746 (3%)	388 (2%)	0.049	2,560 (2%)	193 (1%)	0.042
Dementia	1,162 (5%)	688 (3%)	0.067	1,301 (4%)	90 (2%)	0.093	1,136 (1%)	81 (0%)	0.083	1,062 (1%)	40 (0%)	0.067
Depressive disorder	2,070 (9%)	2,449 (12%)	0.125	3,188 (9%)	502 (12%)	0.098	7,303 (8%)	2,075 (11%)	0.122	10,051 (7%)	1,623 (10%)	0.127
Diabetes	3,964 (16%)	3,599 (18%)	0.048	4,814 (14%)	638 (16%)	0.047	6,071 (6%)	900 (5%)	0.064	6,594 (4%)	644 (4%)	0.018
GERD	2,532 (11%)	3,307 (17%)	0.184	3,364 (10%)	587 (14%)	0.142	6,296 (7%)	1,858 (10%)	0.125	7,446 (5%)	1,050 (7%)	0.070
Heart failure	8,750 (36%)	7,743 (39%)	0.062	9,570 (28%)	1,026 (25%)	0.060	10,000 (10%)	1,057 (6%)	0.174	9,185 (6%)	525 (3%)	0.135
Hypertension	16,718 (69%)	16,325 (83%)	0.321	20,659 (60%)	2,838 (70%)	0.202	29,995 (31%)	5,624 (30%)	0.020	27,979 (19%)	2,798 (18%)	0.029
Hypothyroidism	2,526 (10%)	2,352 (12%)	0.046	3,197 (9%)	464 (11%)	0.069	4,732 (5%)	988 (5%)	0.018	5,173 (4%)	576 (4%)	0.009
Malignant neoplastic disease	3,216 (13%)	3,873 (20%)	0.171	5,060 (15%)	700 (17%)	0.067	4,587 (5%)	764 (4%)	0.032	4,139 (3%)	352 (2%)	0.036
Myocardial infarction	539 (2%)	591 (3%)	0.048	696 (2%)	82 (2%)	0.001	850 (1%)	108 (1%)	0.035	779 (1%)	58 (0%)	0.024
Osteoporosis	1,283 (5%)	1,628 (8%)	0.117	1,447 (4%)	200 (5%)	0.034	1,550 (2%)	197 (1%)	0.048	1,403 (1%)	117 (1%)	0.022
Pneumonia	1,621 (7%)	1,624 (8%)	0.058	2,080 (6%)	264 (6%)	0.018	3,353 (3%)	672 (4%)	0.007	3,920 (3%)	499 (3%)	0.031

Mercadé-Besora N, *et al. Heart* 2024; 110:635–643. doi: 10.1136/heartjnl-2023-323483

	Cohort 1			Cohort 2			Cohort 3			Cohort 4		
	Unvaccinated	Vaccinated	ASMD	Unvaccinated	Vaccinated	ASMD	Unvaccinated	Vaccinated	ASMD	Unvaccinated	Vaccinated	ASMD
Rheumatoid arthritis	491 (2%)	603 (3%)	0.065	892 (3%)	137 (3%)	0.045	1,025 (1%)	190 (1%)	0.004	990 (1%)	99 (1%)	0.005
Stroke	1,129 (5%)	834 (4%)	0.022	1,447 (4%)	152 (4%)	0.025	1,370 (1%)	155 (1%)	0.056	1,254 (1%)	90 (1%)	0.033
Venous thromboembolism	1,067 (4%)	972 (5%)	0.024	1,353 (4%)	140 (3%)	0.027	1,808 (2%)	266 (1%)	0.035	1,785 (1%)	156 (1%)	0.021

The 4 cohorts represent vaccine rollout periods. *Calculated as the days of previous observation in the database before index date. **Assessed anytime before index date. ASMD = Absolute standardized mean difference, GP = General practice, PCR = Polymerase chain reaction, COPD = Chronic obstructive pulmonary disease, GERD = Gastro-Esophageal reflux disease

Mercadé-Besora N, *et al. Heart* 2024; 110:635–643. doi: 10.1136/heartjnl-2023-323483

Table S22: Characteristics of weighted populations in CPRD AURUM database, stratified by staggered cohort and vaccine.

	Cohort 1			Cohort 2			Cohort 3			Cohort 4		
	**ChAdOx1**	**BNT162b2**	**ASMD**	**ChAdOx1**	**BNT162b2**	**ASMD**	**ChAdOx1**	**BNT162b2**	**ASMD**	**ChAdOx1**	**BNT162b2**	**ASMD**
**N (individuals)**	48,138	48,432		306,114	307,972		35,517	35,840		117,220	116,722	
**Age, median [Q25-Q75]**	80 [77-85]	80 [77-85]	0.003	66 [57-72]	66 [57-72]	0.003	54 [46-60]	54 [46-60]	0.000	42 [39-46]	42 [39-46]	0.004
**Sex: Female, N(%)**	27,920 (58%)	27,962 (58%)	0.005	167,623 (55%)	168,473 (55%)	0.001	21,299 (60%)	21,484 (60%)	0.000	54,350 (46%)	54,304 (47%)	0.003
**Years of prior history*, median [Q25-Q75]**	24 [9-36]	25 [11-36]	0.011	21 [9-32]	21 [9-32]	0.000	17 [8-27]	17 [7-27]	0.007	9 [4-17]	9 [4-17]	0.001
**Number of GP visits, median [Q25-Q75]**	12 [6-19]	12 [6-19]	0.003	10 [5-16]	10 [6-16]	0.001	8 [4-14]	8 [4-14]	0.012	4 [1-8]	4 [1-8]	0.008
**Number of PCR tests, median [Q25-Q75]**	0[0-0]	0[0-0]	0.006	0[0-0]	0[0-0]	0.003	0[0-0]	0[0-0]	0.013	0[0-0]	0[0-0]	0.003
**Comorbidities**, N(%)**												
Anxiety	7,101 (15%)	7,442 (15%)	0.017	62,230 (20%)	61,892 (20%)	0.006	7,419 (21%)	7,362 (21%)	0.009	19,086 (16%)	19,543 (17%)	0.012
Asthma	5,241 (11%)	5,396 (11%)	0.008	57,043 (19%)	56,551 (18%)	0.007	6,365 (18%)	6,097 (17%)	0.024	9,186 (8%)	9,059 (8%)	0.003
Chronic kidney disease	11,403 (24%)	11,809 (24%)	0.016	28,219 (9%)	29,790 (10%)	0.016	1,565 (4%)	1,700 (5%)	0.016	793 (1%)	897 (1%)	0.011
COPD	4,080 (8%)	4,014 (8%)	0.007	20,765 (7%)	20,456 (7%)	0.006	661 (2%)	669 (2%)	0.000	424 (0%)	471 (0%)	0.007
Dementia	3,667 (8%)	3,510 (7%)	0.014	2,733 (1%)	2,137 (1%)	0.022	419 (1%)	288 (1%)	0.038	109 (0%)	60 (0%)	0.016
Depressive disorder	5,666 (12%)	5,885 (12%)	0.012	56,642 (19%)	57,377 (19%)	0.003	6,508 (18%)	6,556 (18%)	0.001	16,027 (14%)	16,191 (14%)	0.006
Diabetes	8,849 (18%)	9,020 (19%)	0.006	50,019 (16%)	50,218 (16%)	0.001	2,560 (7%)	2,850 (8%)	0.028	3,231 (3%)	3,106 (3%)	0.006
GERD	2,637 (5%)	2,694 (6%)	0.004	16,666 (5%)	16,658 (5%)	0.002	1,479 (4%)	1,443 (4%)	0.007	3,087 (3%)	3,046 (3%)	0.001
Heart failure	2,843 (6%)	2,904 (6%)	0.004	6,822 (2%)	6,936 (2%)	0.002	443 (1%)	361 (1%)	0.023	204 (0%)	202 (0%)	0.000
Hypertension	25,422 (53%)	26,106 (54%)	0.022	103,545 (34%)	105,521 (34%)	0.009	6,295 (18%)	6,514 (18%)	0.012	6,692 (6%)	6,522 (6%)	0.005
Hypothyroidism	4,806 (10%)	4,978 (10%)	0.010	22,667 (7%)	23,290 (8%)	0.006	1,845 (5%)	1,886 (5%)	0.003	2,985 (3%)	3,025 (3%)	0.003
Malignant neoplastic disease	10,958 (23%)	11,284 (23%)	0.013	38,507 (13%)	40,499 (13%)	0.017	2,019 (6%)	2,152 (6%)	0.014	1,504 (1%)	1,576 (1%)	0.006
Myocardial infarction	2,481 (5%)	2,516 (5%)	0.002	11,083 (4%)	11,197 (4%)	0.001	425 (1%)	521 (1%)	0.022	256 (0%)	349 (0%)	0.016
Osteoporosis	5,117 (11%)	5,335 (11%)	0.012	11,759 (4%)	11,858 (4%)	0.000	719 (2%)	667 (2%)	0.012	344 (0%)	372 (0%)	0.004

Mercadé-Besora N, *et al. Heart* 2024; 110:635–643. doi: 10.1136/heartjnl-2023-323483

	Cohort 1			Cohort 2			Cohort 3			Cohort 4		
	ChAdOx1	BNT162b2	ASMD	ChAdOx1	BNT162b2	ASMD	ChAdOx1	BNT162b2	ASMD	ChAdOx1	BNT162b2	ASMD
Pneumonia	2,576 (5%)	2,527 (5%)	0.006	9,543 (3%)	9,423 (3%)	0.003	808 (2%)	692 (2%)	0.024	979 (1%)	904 (1%)	0.007
Rheumatoid arthritis	935 (2%)	1,005 (2%)	0.010	6,476 (2%)	6,640 (2%)	0.003	284 (1%)	344 (1%)	0.017	216 (0%)	258 (0%)	0.008
Stroke	2,314 (5%)	2,418 (5%)	0.009	7,284 (2%)	7,949 (3%)	0.013	446 (1%)	414 (1%)	0.009	294 (0%)	336 (0%)	0.007
Venous thromboembolism	3,001 (6%)	3,101 (6%)	0.007	11,833 (4%)	12,315 (4%)	0.007	812 (2%)	853 (2%)	0.006	601 (1%)	765 (1%)	0.019

The 4 cohorts represent vaccine rollout periods. *Calculated as the days of previous observation in the database before index date. **Assessed anytime before index date. ASMD = Absolute standardized mean difference, GP = General practice, PCR = Polymerase chain reaction, COPD = Chronic obstructive pulmonary disease, GERD = Gastro-Esophageal reflux disease

Table S23: Characteristics of unweighted populations in CPRD AURUM database, stratified by staggered cohort and vaccine.

	Cohort 1			Cohort 2			Cohort 3			Cohort 4		
	ChAdOx1	BNT162b2	ASMD	ChAdOx1	BNT162b2	ASMD	ChAdOx1	BNT162b2	ASMD	ChAdOx1	BNT162b2	ASMD
**N (individuals)**	219,804	332,790		969,262	594,262		1,473,602	54,102		542,670	1,335,671	
**Age, median [Q25-Q75]**	80 [77-85]	82 [79-86]	0.129	69 [63-73]	67 [56-73]	0.148	55 [51-60]	58 [50-82]	0.381	45 [42-48]	31 [25-37]	1.319
**Sex: Female, N(%)**	127,656 (58%)	186,481 (56%)	0.041	528,692 (55%)	324,259 (55%)	0.000	739,444 (50%)	32,310 (60%)	0.193	242,758 (45%)	625,195 (47%)	0.042
**Years of prior history*, median [Q25-Q75]**	24 [9-36]	26 [13-38]	0.059	21 [9-33]	21 [9-32]	0.028	17 [8-27]	19 [8-30]	0.116	10 [5-18]	7 [3-19]	0.146
**Number of GP visits, median [Q25-Q75]**	12 [6-19]	11 [6-18]	0.038	10 [6-17]	11 [6-17]	0.015	7 [3-12]	10 [5-17]	0.294	3 [1-8]	3 [1-7]	0.045
**Number of PCR tests, median [Q25-Q75]**	0[0-0]	0[0-0]	0.183	0[0-0]	0[0-0]	0.014	0[0-0]	0[0-0]	0.171	0[0-0]	0[0-0]	0.008
**Comorbidities**, N(%)**												
Anxiety	33,355 (15%)	49,410 (15%)	0.009	187,469 (19%)	122,087 (21%)	0.030	281,846 (19%)	10,441 (19%)	0.004	86,805 (16%)	218,144 (16%)	0.009
Asthma	24,009 (11%)	37,889 (11%)	0.015	159,162 (16%)	116,596 (20%)	0.083	200,995 (14%)	8,668 (16%)	0.067	41,393 (8%)	114,378 (9%)	0.034
Chronic kidney disease	51,053 (23%)	86,899 (26%)	0.067	97,339 (10%)	56,923 (10%)	0.016	24,198 (2%)	6,692 (12%)	0.430	2,879 (1%)	2,664 (0%)	0.055
COPD	19,105 (9%)	28,317 (9%)	0.007	67,700 (7%)	39,595 (7%)	0.013	13,621 (1%)	2,063 (4%)	0.191	1,510 (0%)	942 (0%)	0.050
Dementia	16,795 (8%)	16,138 (5%)	0.116	12,106 (1%)	3,720 (1%)	0.065	1,896 (0%)	1,323 (2%)	0.207	447 (0%)	119 (0%)	0.034
Depressive disorder	27,009 (12%)	37,831 (11%)	0.028	170,626 (18%)	112,753 (19%)	0.035	256,789 (17%)	9,046 (17%)	0.019	74,210 (14%)	155,037 (12%)	0.062
Diabetes	39,751 (18%)	61,929 (19%)	0.014	154,348 (16%)	98,676 (17%)	0.018	78,394 (5%)	5,817 (11%)	0.201	9,729 (2%)	12,040 (1%)	0.077
GERD	12,156 (6%)	20,204 (6%)	0.023	52,694 (5%)	32,288 (5%)	0.000	56,444 (4%)	2,506 (5%)	0.040	13,807 (3%)	21,599 (2%)	0.065
Heart failure	13,147 (6%)	20,430 (6%)	0.007	24,276 (3%)	13,120 (2%)	0.020	5,673 (0%)	1,529 (3%)	0.195	681 (0%)	448 (0%)	0.033
Hypertension	114,922 (52%)	186,367 (56%)	0.075	345,014 (36%)	200,762 (34%)	0.038	224,543 (15%)	15,536 (29%)	0.330	31,330 (6%)	19,649 (1%)	0.232
Hypothyroidism	21,855 (10%)	34,512 (10%)	0.014	73,992 (8%)	44,456 (7%)	0.006	64,026 (4%)	3,642 (7%)	0.104	13,920 (3%)	18,516 (1%)	0.085
Malignant neoplastic disease	49,023 (22%)	82,610 (25%)	0.059	131,135 (14%)	76,103 (13%)	0.021	55,601 (4%)	6,311 (12%)	0.299	7,505 (1%)	5,104 (0%)	0.107
Myocardial infarction	11,236 (5%)	18,289 (5%)	0.017	33,142 (3%)	23,019 (4%)	0.024	11,237 (1%)	1,464 (3%)	0.149	861 (0%)	715 (0%)	0.032

Mercadé-Besora N, *et al. Heart* 2024; 110:635–643. doi: 10.1136/heartjnl-2023-323483

	Cohort 1			Cohort 2			Cohort 3			Cohort 4		
	**ChAdOx1**	**BNT162b2**	**ASMD**	**ChAdOx1**	**BNT162b2**	**ASMD**	**ChAdOx1**	**BNT162b2**	**ASMD**	**ChAdOx1**	**BNT162b2**	**ASMD**
Osteoporosis	22,986 (10%)	36,535 (11%)	0.017	42,319 (4%)	21,789 (4%)	0.036	12,455 (1%)	2,632 (5%)	0.243	1,380 (0%)	1,028 (0%)	0.044
Pneumonia	12,101 (6%)	16,297 (5%)	0.027	33,170 (3%)	17,567 (3%)	0.027	20,399 (1%)	1,658 (3%)	0.114	4,297 (1%)	7,793 (1%)	0.025
Rheumatoid arthritis	4,544 (2%)	6,693 (2%)	0.004	20,294 (2%)	12,878 (2%)	0.005	8,463 (1%)	667 (1%)	0.070	821 (0%)	891 (0%)	0.026
Stroke	10,876 (5%)	15,601 (5%)	0.012	24,851 (3%)	15,056 (3%)	0.002	9,547 (1%)	1,281 (2%)	0.141	1,153 (0%)	1,022 (0%)	0.036
Venous thromboembolism	13,782 (6%)	21,549 (6%)	0.008	39,415 (4%)	23,632 (4%)	0.005	24,312 (2%)	1,980 (4%)	0.125	1,936 (0%)	2,541 (0%)	0.032

The 4 cohorts represent vaccine rollout periods. *Calculated as the days of previous observation in the database before index date. **Assessed anytime before index date. ASMD = Absolute standardized mean difference, GP = General practice, PCR = Polymerase chain reaction, COPD = Chronic obstructive pulmonary disease, GERD = Gastro-Esophageal reflux disease

Table S24: Characteristics of weighted populations in CPRD GOLD database, stratified by staggered cohort and vaccine.

	Cohort 1			Cohort 2			Cohort 3			Cohort 4		
	**ChAdOx1**	**BNT162b2**	**ASMD**	**ChAdOx1**	**BNT162b2**	**ASMD**	**ChAdOx1**	**BNT162b2**	**ASMD**	**ChAdOx1**	**BNT162b2**	**ASMD**
**N (individuals)**	7,490	7,490		77,012	76,978		24,590	24,597		49,538	49,577	
**Age, median [Q25-Q75]**	82 [78-86]	80 [78-86]	0.000	70 [65-74]	70 [65-74]	0.004	54 [49-59]	54 [49-59]	0.009	42 [37-46]	42 [37-47]	0.003
**Sex: Female, N(%)**	4,266 (57%)	4,266 (57%)	0.000	41,394 (54%)	41,607 (54%)	0.006	13,052 (53%)	13,078 (53%)	0.002	22,308 (45%)	22,520 (45%)	0.008
**Years of prior history*, median [Q25-Q75]**	18 [12-21]	18 [11-21]	0.000	17 [12-20]	17 [12-20]	0.005	17 [12-20]	17 [12-20]	0.004	14 [6-18]	14 [6-18]	0.005
**Number of GP visits, median [Q25-Q75]**	14 [9-21]	14 [9-21]	0.007	10 [6-16]	10 [6-17]	0.012	6 [2-11]	6 [2-11]	0.009	2 [0-6]	2 [0-7]	0.008
**Number of PCR tests, median [Q25-Q75]**	0[0-0]	0[0-0]	0.004	0[0-0]	0[0-0]	0.002	0[0-0]	0[0-0]	0.011	0[0-0]	0[0-0]	0.002
**Comorbidities**, N(%)**												
Anxiety	1,008 (13%)	1,015 (14%)	0.002	12,765 (17%)	12,897 (17%)	0.005	5,218 (21%)	5,293 (22%)	0.007	8,501 (17%)	8,578 (17%)	0.004
Asthma	783 (10%)	774 (10%)	0.004	9,867 (13%)	10,028 (13%)	0.006	2,993 (12%)	2,826 (11%)	0.021	3,700 (7%)	3,568 (7%)	0.010
Chronic kidney disease	1,711 (23%)	1,694 (23%)	0.005	7,209 (9%)	7,045 (9%)	0.007	530 (2%)	411 (2%)	0.035	193 (0%)	202 (0%)	0.003
COPD	568 (8%)	583 (8%)	0.008	5,739 (7%)	5,725 (7%)	0.001	321 (1%)	274 (1%)	0.017	170 (0%)	165 (0%)	0.002
Dementia	543 (7%)	495 (7%)	0.025	726 (1%)	737 (1%)	0.001	55 (0%)	74 (0%)	0.015	28 (0%)	21 (0%)	0.006
Depressive disorder	733 (10%)	729 (10%)	0.002	11,461 (15%)	11,746 (15%)	0.011	4,478 (18%)	4,427 (18%)	0.005	7,038 (14%)	7,119 (14%)	0.004
Diabetes	1,114 (15%)	1,107 (15%)	0.003	10,260 (13%)	10,130 (13%)	0.005	1,051 (4%)	963 (4%)	0.018	768 (2%)	654 (1%)	0.019
GERD	451 (6%)	447 (6%)	0.002	3,691 (5%)	3,753 (5%)	0.004	763 (3%)	770 (3%)	0.001	975 (2%)	978 (2%)	0.000
Heart failure	395 (5%)	411 (5%)	0.009	1,763 (2%)	1,819 (2%)	0.005	125 (1%)	81 (0%)	0.028	50 (0%)	54 (0%)	0.002
Hypertension	2,761 (37%)	2,754 (37%)	0.002	21,627 (28%)	21,686 (28%)	0.002	3,184 (13%)	3,261 (13%)	0.009	1,842 (4%)	1,975 (4%)	0.014
Hypothyroidism	579 (8%)	566 (8%)	0.007	4,932 (6%)	4,869 (6%)	0.003	1,064 (4%)	1,065 (4%)	0.000	979 (2%)	1,028 (2%)	0.007
Malignant neoplastic disease	1,632 (22%)	1,744 (23%)	0.036	10,682 (14%)	9,986 (13%)	0.026	1,007 (4%)	1,089 (4%)	0.017	598 (1%)	660 (1%)	0.011
Myocardial infarction	330 (4%)	325 (4%)	0.004	2,919 (4%)	2,943 (4%)	0.002	239 (1%)	217 (1%)	0.010	90 (0%)	85 (0%)	0.002

Mercadé-Besora N, *et al. Heart* 2024; 110:635–643. doi: 10.1136/heartjnl-2023-323483

	Cohort 1			Cohort 2			Cohort 3			Cohort 4		
	**ChAdOx1**	**BNT162b2**	**ASMD**	**ChAdOx1**	**BNT162b2**	**ASMD**	**ChAdOx1**	**BNT162b2**	**ASMD**	**ChAdOx1**	**BNT162b2**	**ASMD**
Osteoporosis	665 (9%)	638 (9%)	0.013	3,388 (4%)	3,496 (5%)	0.007	323 (1%)	314 (1%)	0.003	103 (0%)	92 (0%)	0.005
Pneumonia	286 (4%)	258 (3%)	0.020	1,807 (2%)	1,724 (2%)	0.007	263 (1%)	252 (1%)	0.005	271 (1%)	265 (1%)	0.002
Rheumatoid arthritis	111 (1%)	111 (1%)	0.001	1,437 (2%)	1,460 (2%)	0.002	123 (1%)	115 (0%)	0.005	58 (0%)	40 (0%)	0.011
Stroke	325 (4%)	321 (4%)	0.003	2,134 (3%)	2,072 (3%)	0.005	207 (1%)	148 (1%)	0.028	96 (0%)	91 (0%)	0.002
Venous thromboembolism	306 (4%)	341 (5%)	0.023	2,125 (3%)	2,153 (3%)	0.002	288 (1%)	285 (1%)	0.001	146 (0%)	198 (0%)	0.018

The 4 cohorts represent vaccine rollout periods. *Calculated as the days of previous observation in the database before index date. **Assessed anytime before index date. ASMD = Absolute standardized mean difference, GP = General practice, PCR = Polymerase chain reaction, COPD = Chronic obstructive pulmonary disease, GERD = Gastro-Esophageal reflux disease

Mercadé-Besora N, *et al. Heart* 2024; 110:635–643. doi: 10.1136/heartjnl-2023-323483

Table S25: Characteristics of unweighted populations in CPRD GOLD database, stratified by staggered cohort and vaccine.

	Cohort 1			Cohort 2			Cohort 3			Cohort 4		
	**ChAdOx1**	**BNT162b2**	**ASMD**	**ChAdOx1**	**BNT162b2**	**ASMD**	**ChAdOx1**	**BNT162b2**	**ASMD**	**ChAdOx1**	**BNT162b2**	**ASMD**
**N (individuals)**	82,406	32,755		302,999	180,670		423,876	36,748		147,744	365,096	
**Age, median [Q25-Q75]**	84 [82-88]	81 [79-86]	0.351	71 [66-76]	70 [66-74]	0.132	56 [52-61]	54 [50-60]	0.171	44 [41-48]	31 [24-37]	1.090
**Sex: Female, N(%)**	47,915 (58%)	18,415 (56%)	0.039	162,753 (54%)	97,747 (54%)	0.008	210,283 (50%)	19,649 (53%)	0.077	64,999 (44%)	167,510 (46%)	0.038
**Years of prior history*, median [Q25-Q75]**	17 [14-20]	18 [13-21]	0.001	17 [13-19]	17 [12-19]	0.014	17 [10-19]	18 [12-20]	0.107	14 [7-18]	15 [6-18]	0.017
**Number of GP visits, median [Q25-Q75]**	14 [9-21]	14 [9-21]	0.005	11 [7-18]	10 [6-16]	0.122	6 [2-12]	6 [2-11]	0.069	3 [0-7]	2 [0-6]	0.098
**Number of PCR tests, median [Q25-Q75]**	0[0-0]	0[0-0]	0.006	0[0-0]	0[0-0]	0.009	0[0-0]	0[0-0]	0.021	0[0-0]	0[0-0]	0.011
**Comorbidities**, N(%)**												
Anxiety	9,923 (12%)	4,336 (13%)	0.036	49,010 (16%)	28,697 (16%)	0.008	75,258 (18%)	8,004 (22%)	0.101	24,601 (17%)	59,094 (16%)	0.013
Asthma	8,491 (10%)	3,250 (10%)	0.013	39,988 (13%)	21,396 (12%)	0.041	48,034 (11%)	4,053 (11%)	0.010	10,517 (7%)	33,240 (9%)	0.073
Chronic kidney disease	21,564 (26%)	7,430 (23%)	0.081	34,168 (11%)	15,033 (8%)	0.100	7,553 (2%)	566 (2%)	0.019	739 (1%)	578 (0%)	0.060
COPD	6,837 (8%)	2,417 (7%)	0.034	27,472 (9%)	11,545 (6%)	0.100	6,334 (1%)	348 (1%)	0.050	640 (0%)	285 (0%)	0.070
Dementia	4,773 (6%)	1,895 (6%)	0.000	5,124 (2%)	1,375 (1%)	0.085	548 (0%)	87 (0%)	0.025	126 (0%)	29 (0%)	0.036
Depressive disorder	6,998 (8%)	3,310 (10%)	0.056	45,154 (15%)	26,294 (15%)	0.010	70,703 (17%)	6,569 (18%)	0.032	21,711 (15%)	39,181 (11%)	0.119
Diabetes	12,387 (15%)	4,882 (15%)	0.004	43,647 (14%)	22,552 (12%)	0.056	24,219 (6%)	1,191 (3%)	0.120	2,254 (2%)	2,507 (1%)	0.080
GERD	4,054 (5%)	2,012 (6%)	0.054	14,294 (5%)	8,525 (5%)	0.000	14,447 (3%)	1,093 (3%)	0.025	2,883 (2%)	5,213 (1%)	0.041
Heart failure	5,058 (6%)	1,782 (5%)	0.030	9,180 (3%)	3,669 (2%)	0.064	2,308 (1%)	100 (0%)	0.043	191 (0%)	98 (0%)	0.037
Hypertension	31,217 (38%)	11,995 (37%)	0.026	88,833 (29%)	49,763 (28%)	0.039	59,554 (14%)	4,679 (13%)	0.039	6,761 (5%)	4,776 (1%)	0.194
Hypothyroidism	7,042 (9%)	2,373 (7%)	0.048	20,521 (7%)	11,106 (6%)	0.025	17,382 (4%)	1,553 (4%)	0.006	3,245 (2%)	4,180 (1%)	0.082
Malignant neoplastic disease	18,950 (23%)	6,972 (21%)	0.041	42,687 (14%)	22,617 (13%)	0.046	17,903 (4%)	1,522 (4%)	0.004	2,192 (1%)	1,796 (0%)	0.100
Myocardial infarction	4,500 (5%)	1,603 (5%)	0.026	13,456 (4%)	6,621 (4%)	0.039	5,280 (1%)	271 (1%)	0.051	330 (0%)	154 (0%)	0.050

Mercadé-Besora N, *et al. Heart* 2024; 110:635–643. doi: 10.1136/heartjnl-2023-323483

	Cohort 1			Cohort 2			Cohort 3			Cohort 4		
	ChAdOx1	BNT162b2	ASMD	ChAdOx1	BNT162b2	ASMD	ChAdOx1	BNT162b2	ASMD	ChAdOx1	BNT162b2	ASMD
Osteoporosis	8,564 (10%)	2,770 (8%)	0.066	16,117 (5%)	7,531 (4%)	0.054	4,330 (1%)	458 (1%)	0.021	408 (0%)	205 (0%)	0.054
Pneumonia	3,231 (4%)	1,032 (3%)	0.042	8,604 (3%)	3,418 (2%)	0.062	4,527 (1%)	345 (1%)	0.013	921 (1%)	1,696 (0%)	0.022
Rheumatoid arthritis	1,257 (2%)	512 (2%)	0.003	5,999 (2%)	2,929 (2%)	0.027	2,405 (1%)	154 (0%)	0.021	191 (0%)	111 (0%)	0.035
Stroke	4,193 (5%)	1,490 (5%)	0.025	10,213 (3%)	4,632 (3%)	0.048	3,789 (1%)	199 (1%)	0.042	369 (0%)	225 (0%)	0.048
Venous thromboembolism	3,550 (4%)	1,389 (4%)	0.003	9,466 (3%)	4,490 (2%)	0.039	6,339 (1%)	370 (1%)	0.044	460 (0%)	496 (0%)	0.037

The 4 cohorts represent vaccine rollout periods. *Calculated as the days of previous observation in the database before index date. **Assessed anytime before index date. ASMD = Absolute standardized mean difference, GP = General practice, PCR = Polymerase chain reaction, COPD = Chronic obstructive pulmonary disease, GERD = Gastro-Esophageal reflux disease

Table S26: Number of records (and risk per 10,000 individuals) for post COVID-19 cardiac and thromboembolic complications, across cohorts and databases, stratified by exposure status (any COVID-19 vaccine). Follow-up ends at first vaccine dose after index date.

Cohort	Time window	Outcome	AURUM		CORIVA		GOLD		SIDIAP	
			Unvaccinated	Vaccinated	Unvaccinated	Vaccinated	Unvaccinated	Vaccinated	Unvaccinated	Vaccinated
**Cohort 1**			**N = 346,674**	**N = 552,602**	**N = 23,982**	**N = 26,736**	**N = 169,100**	**N = 118,507**	**N = 223,962**	**N = 89,941**
	**0 to 30 days**	**VTE**	93 (2.68)	45 (0.81)	77 (32.11)	6 (2.24)	8 (0.47)	< 5	74 (3.30)	< 5
		**DVT**	22 (0.63)	10 (0.18)	12 (5.00)	< 5	< 5	< 5	19 (0.85)	< 5
		**PE**	75 (2.16)	37 (0.67)	67 (27.94)	5 (1.87)	7 (0.41)	< 5	59 (2.63)	< 5
		**ATE**	22 (0.63)	28 (0.51)	110 (45.87)	6 (2.24)	6 (0.35)	< 5	77 (3.44)	6 (0.67)
		**IS**	8 (0.23)	< 5	64 (26.69)	< 5	< 5	< 5	45 (2.01)	< 5
		**TIA**	< 5	6 (0.11)	20 (8.34)	< 5	< 5	< 5	7 (0.31)	< 5
		**MI**	11 (0.32)	20 (0.36)	35 (14.59)	5 (1.87)	< 5	< 5	27 (1.21)	< 5
		**HF**	59 (1.70)	73 (1.32)	395 (164.71)	27 (10.10)	10 (0.59)	< 5	302 (13.48)	23 (2.56)
		**HS**	< 5	< 5	< 5	< 5	< 5	< 5	7 (0.31)	< 5
		**MP**	< 5	< 5	< 5	< 5	< 5	< 5	< 5	< 5
	**31 to 90 days**	**VTE**	19 (0.55)	20 (0.36)	37 (15.43)	< 5	< 5	< 5	16 (0.71)	< 5
		**DVT**	10 (0.29)	7 (0.13)	20 (8.34)	< 5	< 5	< 5	10 (0.45)	< 5
		**PE**	9 (0.26)	13 (0.24)	21 (8.76)	< 5	< 5	< 5	6 (0.27)	< 5
		**ATE**	5 (0.14)	16 (0.29)	33 (13.76)	< 5	< 5	< 5	41 (1.83)	5 (0.56)
		**IS**	< 5	< 5	19 (7.92)	< 5	< 5	< 5	20 (0.89)	< 5
		**TIA**	< 5	6 (0.11)	8 (3.34)	< 5	< 5	< 5	13 (0.58)	< 5
		**MI**	< 5	7 (0.13)	12 (5.00)	< 5	< 5	< 5	11 (0.49)	< 5
		**HF**	30 (0.87)	56 (1.01)	151 (62.96)	14 (5.24)	< 5	< 5	89 (3.97)	8 (0.89)
		**HS**	< 5	< 5	< 5	< 5	< 5	< 5	< 5	< 5
		**MP**	< 5	< 5	< 5	< 5	< 5	< 5	< 5	< 5
	**91 to 180 days**	**VTE**	10 (0.29)	9 (0.16)	21 (8.76)	5 (1.87)	< 5	< 5	20 (0.89)	< 5
		**DVT**	5 (0.14)	< 5	14 (5.84)	< 5	< 5	< 5	9 (0.40)	< 5
		**PE**	5 (0.14)	5 (0.09)	9 (3.75)	< 5	< 5	< 5	11 (0.49)	< 5
		**ATE**	11 (0.32)	14 (0.25)	31 (12.93)	< 5	< 5	< 5	30 (1.34)	< 5
		**IS**	< 5	< 5	16 (6.67)	< 5	< 5	< 5	16 (0.71)	< 5
		**TIA**	5 (0.14)	6 (0.11)	8 (3.34)	< 5	< 5	< 5	8 (0.36)	< 5
		**MI**	5 (0.14)	< 5	10 (4.17)	< 5	< 5	< 5	7 (0.31)	< 5
		**HF**	37 (1.07)	51 (0.92)	162 (67.55)	21 (7.85)	< 5	< 5	87 (3.88)	7 (0.78)
		**HS**	< 5	< 5	< 5	< 5	< 5	< 5	< 5	< 5
		**MP**	< 5	< 5	< 5	< 5	< 5	< 5	< 5	< 5

Cohort	Time window	Outcome	AURUM		CORIVA		GOLD		SIDIAP	
			Unvaccinated	Vaccinated	Unvaccinated	Vaccinated	Unvaccinated	Vaccinated	Unvaccinated	Vaccinated
	**181 to 365 days**	**VTE**	10 (0.29)	8 (0.14)	45 (18.76)	7 (2.62)	< 5	< 5	10 (0.45)	< 5
		**DVT**	5 (0.14)	< 5	16 (6.67)	< 5	< 5	< 5	6 (0.27)	< 5
		**PE**	5 (0.14)	< 5	34 (14.18)	< 5	< 5	< 5	< 5	< 5
		**ATE**	10 (0.29)	19 (0.34)	55 (22.93)	11 (4.11)	< 5	< 5	42 (1.88)	8 (0.89)
		**IS**	< 5	6 (0.11)	32 (13.34)	10 (3.74)	< 5	< 5	21 (0.94)	5 (0.56)
		**TIA**	6 (0.17)	< 5	16 (6.67)	< 5	< 5	< 5	10 (0.45)	< 5
		**MI**	< 5	10 (0.18)	14 (5.84)	< 5	< 5	< 5	11 (0.49)	< 5
		**HF**	40 (1.15)	53 (0.96)	268 (111.75)	48 (17.95)	< 5	6 (0.51)	86 (3.84)	20 (2.22)
		**HS**	< 5	< 5	< 5	< 5	< 5	< 5	< 5	< 5
		**MP**	< 5	< 5	< 5	< 5	< 5	< 5	< 5	< 5
**Cohort 2**			**N = 1,975,726**	**N = 1,563,569**	**N = 34,317**	**N = 4,572**	**N = 583,399**	**N = 486,619**	**N = 433,151**	**N = 819,590**
	**0 to 30 days**	**VTE**	241 (1.22)	54 (0.35)	79 (23.02)	< 5	31 (0.53)	7 (0.14)	258 (5.96)	27 (0.33)
		**DVT**	41 (0.21)	19 (0.12)	12 (3.50)	< 5	8 (0.14)	< 5	63 (1.45)	9 (0.11)
		**PE**	204 (1.03)	38 (0.24)	69 (20.11)	< 5	24 (0.41)	6 (0.12)	213 (4.92)	20 (0.24)
		**ATE**	41 (0.21)	25 (0.16)	110 (32.05)	< 5	< 5	< 5	173 (3.99)	29 (0.35)
		**IS**	7 (0.04)	5 (0.03)	68 (19.82)	< 5	< 5	< 5	96 (2.22)	12 (0.15)
		**TIA**	8 (0.04)	< 5	18 (5.25)	< 5	< 5	< 5	17 (0.39)	6 (0.07)
		**MI**	26 (0.13)	17 (0.11)	35 (10.20)	< 5	< 5	< 5	64 (1.48)	11 (0.13)
		**HF**	45 (0.23)	47 (0.30)	364 (106.07)	< 5	5 (0.09)	< 5	378 (8.73)	76 (0.93)
		**HS**	6 (0.03)	< 5	< 5	< 5	< 5	< 5	18 (0.42)	< 5
		**MP**	12 (0.06)	< 5	< 5	< 5	< 5	< 5	14 (0.32)	< 5
	**31 to 90 days**	**VTE**	43 (0.22)	13 (0.08)	31 (9.03)	< 5	< 5	< 5	59 (1.36)	9 (0.11)
		**DVT**	24 (0.12)	6 (0.04)	16 (4.66)	< 5	< 5	< 5	32 (0.74)	5 (0.06)
		**PE**	23 (0.12)	8 (0.05)	18 (5.25)	< 5	< 5	< 5	33 (0.76)	< 5
		**ATE**	18 (0.09)	18 (0.12)	32 (9.32)	< 5	< 5	< 5	85 (1.96)	14 (0.17)
		**IS**	< 5	< 5	20 (5.83)	< 5	< 5	< 5	43 (0.99)	6 (0.07)
		**TIA**	< 5	7 (0.04)	< 5	< 5	< 5	< 5	23 (0.53)	5 (0.06)
		**MI**	13 (0.07)	10 (0.06)	13 (3.79)	< 5	< 5	< 5	26 (0.60)	< 5
		**HF**	27 (0.14)	30 (0.19)	149 (43.42)	< 5	< 5	< 5	138 (3.19)	29 (0.35)
		**HS**	< 5	< 5	< 5	< 5	< 5	< 5	7 (0.16)	< 5
		**MP**	6 (0.03)	< 5	< 5	< 5	< 5	< 5	< 5	< 5
	**91 to 180 days**	**VTE**	28 (0.14)	15 (0.10)	26 (7.58)	< 5	6 (0.10)	< 5	58 (1.34)	6 (0.07)
		**DVT**	13 (0.07)	9 (0.06)	15 (4.37)	< 5	< 5	< 5	31 (0.72)	6 (0.07)
		**PE**	15 (0.08)	6 (0.04)	12 (3.50)	< 5	< 5	< 5	30 (0.69)	< 5

BMJ Publishing Group Limited (BMJ) disclaims all liability and responsibility arising from any reliance placed on this supplemental material which has been supplied by the author(s)

Cohort	Time window	Outcome	AURUM		CORIVA		GOLD		SIDIAP	
			Unvaccinated	Vaccinated	Unvaccinated	Vaccinated	Unvaccinated	Vaccinated	Unvaccinated	Vaccinated
		ATE	17 (0.09)	15 (0.10)	32 (9.32)	< 5	< 5	< 5	91 (2.10)	19 (0.23)
		IS	< 5	< 5	15 (4.37)	< 5	< 5	< 5	49 (1.13)	9 (0.11)
		TIA	9 (0.05)	5 (0.03)	11 (3.21)	< 5	< 5	< 5	20 (0.46)	5 (0.06)
		MI	9 (0.05)	10 (0.06)	8 (2.33)	< 5	< 5	< 5	25 (0.58)	6 (0.07)
		HF	22 (0.11)	27 (0.17)	166 (48.37)	< 5	< 5	< 5	110 (2.54)	41 (0.50)
		HS	< 5	< 5	< 5	< 5	< 5	< 5	7 (0.16)	< 5
		MP	< 5	< 5	< 5	< 5	< 5	< 5	7 (0.16)	< 5
	181 to 365 days	VTE	9 (0.05)	12 (0.08)	44 (12.82)	< 5	< 5	< 5	16 (0.37)	23 (0.28)
		DVT	< 5	5 (0.03)	20 (5.83)	< 5	< 5	< 5	9 (0.21)	14 (0.17)
		PE	5 (0.03)	7 (0.04)	27 (7.87)	< 5	< 5	< 5	8 (0.18)	10 (0.12)
		ATE	12 (0.06)	16 (0.10)	53 (15.44)	< 5	< 5	< 5	63 (1.45)	33 (0.40)
		IS	< 5	< 5	31 (9.03)	< 5	< 5	< 5	35 (0.81)	11 (0.13)
		TIA	< 5	< 5	12 (3.50)	< 5	< 5	< 5	16 (0.37)	13 (0.16)
		MI	8 (0.04)	10 (0.06)	15 (4.37)	< 5	< 5	< 5	14 (0.32)	10 (0.12)
		HF	20 (0.10)	31 (0.20)	259 (75.47)	5 (10.94)	< 5	< 5	81 (1.87)	41 (0.50)
		HS	< 5	< 5	< 5	< 5	< 5	< 5	< 5	5 (0.06)
		MP	< 5	< 5	< 5	< 5	< 5	< 5	< 5	< 5
Cohort 3			N = 1,510,401	N = 1,528,031	N = 96,423	N = 24,050	N = 417,996	N = 462,832	N = 869,497	N = 954,232
	0 to 30 days	VTE	245 (1.62)	25 (0.16)	115 (11.93)	< 5	27 (0.65)	< 5	325 (3.74)	27 (0.28)
		DVT	45 (0.30)	< 5	22 (2.28)	< 5	6 (0.14)	< 5	90 (1.04)	7 (0.07)
		PE	209 (1.38)	22 (0.14)	96 (9.96)	< 5	22 (0.53)	< 5	262 (3.01)	22 (0.23)
		ATE	29 (0.19)	10 (0.07)	119 (12.34)	< 5	< 5	< 5	213 (2.45)	14 (0.15)
		IS	< 5	< 5	70 (7.26)	< 5	< 5	< 5	102 (1.17)	5 (0.05)
		TIA	5 (0.03)	< 5	19 (1.97)	< 5	< 5	< 5	20 (0.23)	< 5
		MI	20 (0.13)	8 (0.05)	40 (4.15)	< 5	< 5	< 5	97 (1.12)	7 (0.07)
		HF	31 (0.21)	18 (0.12)	380 (39.41)	< 5	< 5	< 5	364 (4.19)	23 (0.24)
		HS	< 5	< 5	< 5	< 5	< 5	< 5	20 (0.23)	< 5
		MP	9 (0.06)	< 5	6 (0.62)	< 5	< 5	< 5	19 (0.22)	< 5
	31 to 90 days	VTE	44 (0.29)	< 5	50 (5.19)	< 5	< 5	< 5	85 (0.98)	9 (0.09)
		DVT	20 (0.13)	< 5	28 (2.90)	< 5	< 5	< 5	57 (0.66)	5 (0.05)
		PE	27 (0.18)	< 5	26 (2.70)	< 5	< 5	< 5	38 (0.44)	< 5
		ATE	11 (0.07)	7 (0.05)	48 (4.98)	< 5	< 5	< 5	109 (1.25)	10 (0.10)
		IS	< 5	< 5	24 (2.49)	< 5	< 5	< 5	54 (0.62)	6 (0.06)
		TIA	< 5	< 5	7 (0.73)	< 5	< 5	< 5	27 (0.31)	< 5

Cohort	Time window	Outcome	AURUM		CORIVA		GOLD		SIDIAP	
			Unvaccinated	Vaccinated	Unvaccinated	Vaccinated	Unvaccinated	Vaccinated	Unvaccinated	Vaccinated
		MI	9 (0.06)	< 5	20 (2.07)	< 5	< 5	< 5	36 (0.41)	< 5
		HF	15 (0.10)	8 (0.05)	180 (18.67)	< 5	< 5	< 5	137 (1.58)	13 (0.14)
		HS	< 5	< 5	< 5	< 5	< 5	< 5	7 (0.08)	< 5
		MP	8 (0.05)	< 5	5 (0.52)	< 5	< 5	< 5	8 (0.09)	< 5
	91 to 180 days	VTE	24 (0.16)	8 (0.05)	43 (4.46)	< 5	< 5	< 5	64 (0.74)	8 (0.08)
		DVT	11 (0.07)	6 (0.04)	26 (2.70)	< 5	< 5	< 5	36 (0.41)	7 (0.07)
		PE	14 (0.09)	< 5	18 (1.87)	< 5	< 5	< 5	32 (0.37)	< 5
		ATE	< 5	7 (0.05)	44 (4.56)	< 5	< 5	< 5	113 (1.30)	20 (0.21)
		IS	< 5	< 5	21 (2.18)	< 5	< 5	< 5	57 (0.66)	12 (0.13)
		TIA	< 5	< 5	13 (1.35)	< 5	< 5	< 5	22 (0.25)	< 5
		MI	< 5	< 5	15 (1.56)	< 5	< 5	< 5	37 (0.43)	6 (0.06)
		HF	11 (0.07)	6 (0.04)	216 (22.40)	< 5	< 5	< 5	120 (1.38)	15 (0.16)
		HS	< 5	< 5	< 5	< 5	< 5	< 5	9 (0.10)	< 5
		MP	< 5	< 5	< 5	< 5	< 5	< 5	8 (0.09)	< 5
	181 to 365 days	VTE	< 5	9 (0.06)	72 (7.47)	< 5	< 5	< 5	34 (0.39)	5 (0.05)
		DVT	< 5	< 5	39 (4.04)	< 5	< 5	< 5	20 (0.23)	< 5
		PE	< 5	6 (0.04)	37 (3.84)	< 5	< 5	< 5	16 (0.18)	< 5
		ATE	< 5	< 5	80 (8.30)	< 5	< 5	< 5	51 (0.59)	18 (0.19)
		IS	< 5	< 5	36 (3.73)	< 5	< 5	< 5	24 (0.28)	9 (0.09)
		TIA	< 5	< 5	19 (1.97)	< 5	< 5	< 5	11 (0.13)	< 5
		MI	< 5	< 5	32 (3.32)	< 5	< 5	< 5	17 (0.20)	7 (0.07)
		HF	5 (0.03)	< 5	324 (33.60)	< 5	< 5	< 5	62 (0.71)	13 (0.14)
		HS	< 5	< 5	7 (0.73)	< 5	< 5	< 5	< 5	< 5
		MP	< 5	< 5	< 5	< 5	< 5	< 5	< 5	< 5
**Cohort 4**			**N = 2,027,763**	**N = 2,085,598**	**N = 147,545**	**N = 22,245**	**N = 469,876**	**N = 550,437**	**N = 1,061,634**	**N = 880,950**
	0 to 30 days	VTE	334 (1.65)	19 (0.09)	116 (7.86)	< 5	36 (0.77)	7 (0.13)	350 (3.30)	38 (0.43)
		DVT	55 (0.27)	6 (0.03)	22 (1.49)	< 5	6 (0.13)	< 5	106 (1.00)	14 (0.16)
		PE	291 (1.44)	13 (0.06)	97 (6.57)	< 5	31 (0.66)	7 (0.13)	272 (2.56)	25 (0.28)
		ATE	26 (0.13)	5 (0.02)	116 (7.86)	< 5	< 5	< 5	231 (2.18)	35 (0.40)
		IS	< 5	< 5	69 (4.68)	< 5	< 5	< 5	115 (1.08)	17 (0.19)
		TIA	6 (0.03)	< 5	18 (1.22)	< 5	< 5	< 5	26 (0.24)	< 5
		MI	16 (0.08)	< 5	38 (2.58)	< 5	< 5	< 5	96 (0.90)	15 (0.17)
		HF	28 (0.14)	< 5	364 (24.67)	< 5	< 5	< 5	362 (3.41)	28 (0.32)
		HS	< 5	< 5	5 (0.34)	< 5	< 5	< 5	23 (0.22)	< 5

Cohort	Time window	Outcome	AURUM		CORIVA		GOLD		SIDIAP	
			Unvaccinated	Vaccinated	Unvaccinated	Vaccinated	Unvaccinated	Vaccinated	Unvaccinated	Vaccinated
		MP	12 (0.06)	< 5	7 (0.47)	< 5	< 5	< 5	26 (0.24)	9 (0.10)
	31 to 90 days	VTE	58 (0.29)	11 (0.05)	54 (3.66)	< 5	5 (0.11)	< 5	91 (0.86)	12 (0.14)
		DVT	21 (0.10)	< 5	32 (2.17)	< 5	< 5	< 5	61 (0.57)	7 (0.08)
		PE	40 (0.20)	7 (0.03)	26 (1.76)	< 5	< 5	< 5	40 (0.38)	6 (0.07)
		ATE	12 (0.06)	< 5	46 (3.12)	< 5	< 5	< 5	118 (1.11)	18 (0.20)
		IS	< 5	< 5	24 (1.63)	< 5	< 5	< 5	52 (0.49)	7 (0.08)
		TIA	< 5	< 5	6 (0.41)	< 5	< 5	< 5	33 (0.31)	7 (0.08)
		MI	9 (0.04)	< 5	19 (1.29)	< 5	< 5	< 5	41 (0.39)	< 5
		HF	14 (0.07)	8 (0.04)	176 (11.93)	< 5	< 5	< 5	142 (1.34)	17 (0.19)
		HS	< 5	< 5	< 5	< 5	< 5	< 5	8 (0.08)	< 5
		MP	7 (0.03)	< 5	5 (0.34)	< 5	< 5	< 5	10 (0.09)	< 5
	91 to 180 days	VTE	26 (0.13)	9 (0.04)	49 (3.32)	< 5	< 5	< 5	71 (0.67)	11 (0.12)
		DVT	10 (0.05)	7 (0.03)	31 (2.10)	< 5	< 5	< 5	42 (0.40)	6 (0.07)
		PE	17 (0.08)	< 5	19 (1.29)	< 5	< 5	< 5	32 (0.30)	6 (0.07)
		ATE	< 5	5 (0.02)	41 (2.78)	< 5	< 5	< 5	128 (1.21)	21 (0.24)
		IS	< 5	< 5	19 (1.29)	< 5	< 5	< 5	65 (0.61)	11 (0.12)
		TIA	< 5	< 5	12 (0.81)	< 5	< 5	< 5	29 (0.27)	< 5
		MI	< 5	< 5	14 (0.95)	< 5	< 5	< 5	37 (0.35)	7 (0.08)
		HF	10 (0.05)	< 5	208 (14.10)	< 5	< 5	< 5	139 (1.31)	19 (0.22)
		HS	< 5	< 5	< 5	< 5	< 5	< 5	14 (0.13)	< 5
		MP	< 5	< 5	5 (0.34)	< 5	< 5	< 5	11 (0.10)	< 5
	181 to 365 days	VTE	< 5	< 5	77 (5.22)	< 5	< 5	< 5	46 (0.43)	9 (0.10)
		DVT	< 5	< 5	46 (3.12)	< 5	< 5	< 5	33 (0.31)	5 (0.06)
		PE	< 5	< 5	35 (2.37)	< 5	< 5	< 5	14 (0.13)	< 5
		ATE	< 5	< 5	73 (4.95)	< 5	< 5	< 5	54 (0.51)	22 (0.25)
		IS	< 5	< 5	33 (2.24)	< 5	< 5	< 5	27 (0.25)	15 (0.17)
		TIA	< 5	< 5	19 (1.29)	< 5	< 5	< 5	12 (0.11)	< 5
		MI	< 5	< 5	27 (1.83)	< 5	< 5	< 5	16 (0.15)	7 (0.08)
		HF	< 5	< 5	301 (20.40)	< 5	< 5	< 5	57 (0.54)	11 (0.12)
		HS	< 5	< 5	8 (0.54)	< 5	< 5	< 5	< 5	< 5
		MP	< 5	< 5	5 (0.34)	< 5	< 5	< 5	7 (0.07)	< 5

VTE = Venous thromboembolism (DVT + PE), DVT = Deep vein thrombosis, PE = Pulmonary embolism, ATE = Arterial thrombosis/thromboembolism (IS + TIA + MI), IS = Ischemic stroke, TIA = Transient ischemic attack, MI = Myocardial infarction, HF = Heart failure, HS = Hemorrhagic stroke, MP = Myocarditis or Pericarditis

Mercadé-Besora N, *et al. Heart* 2024; 110:635–643. doi: 10.1136/heartjnl-2023-323483

Table S27: Number of records (and risk per 10,000 individuals) for post COVID-19 cardiac and thromboembolic complications, across cohorts and databases, stratified by exposure status (any COVID-19 vaccine). Only first outcome after COVID-19 captured.

Cohort	Time window	Outcome	AURUM		CORIVA		GOLD		SIDIAP	
			Unvaccinated	Vaccinated	Unvaccinated	Vaccinated	Unvaccinated	Vaccinated	Unvaccinated	Vaccinated
**Cohort 1**			**N = 346,674**	**N = 552,602**	**N = 23,982**	**N = 26,736**	**N = 169,100**	**N = 118,507**	**N = 223,962**	**N = 89,941**
	**0 to 30 days**	**VTE**	93 (2.68)	117 (2.12)	77 (32.11)	45 (16.83)	8 (0.47)	8 (0.68)	74 (3.30)	96 (10.67)
		**DVT**	22 (0.63)	27 (0.49)	12 (5.00)	14 (5.24)	< 5	< 5	19 (0.85)	29 (3.22)
		**PE**	75 (2.16)	95 (1.72)	67 (27.94)	33 (12.34)	7 (0.41)	7 (0.59)	59 (2.63)	77 (8.56)
		**ATE**	22 (0.63)	70 (1.27)	110 (45.87)	81 (30.30)	6 (0.35)	7 (0.59)	77 (3.44)	208 (23.13)
		**IS**	8 (0.23)	5 (0.09)	64 (26.69)	37 (13.84)	< 5	< 5	45 (2.01)	116 (12.90)
		**TIA**	< 5	18 (0.33)	20 (8.34)	18 (6.73)	< 5	< 5	7 (0.31)	41 (4.56)
		**MI**	11 (0.32)	46 (0.83)	35 (14.59)	35 (13.09)	< 5	5 (0.42)	27 (1.21)	63 (7.00)
		**HF**	59 (1.70)	198 (3.58)	395 (164.71)	299 (111.83)	10 (0.59)	9 (0.76)	302 (13.48)	640 (71.16)
		**HS**	< 5	7 (0.13)	< 5	< 5	< 5	< 5	7 (0.31)	14 (1.56)
		**MP**	< 5	< 5	< 5	< 5	< 5	< 5	< 5	< 5
	**31 to 90 days**	**VTE**	19 (0.55)	39 (0.71)	35 (14.59)	29 (10.85)	< 5	< 5	15 (0.67)	45 (5.00)
		**DVT**	10 (0.29)	15 (0.27)	19 (7.92)	15 (5.61)	< 5	< 5	9 (0.40)	29 (3.22)
		**PE**	9 (0.26)	25 (0.45)	20 (8.34)	14 (5.24)	< 5	< 5	6 (0.27)	20 (2.22)
		**ATE**	5 (0.14)	43 (0.78)	29 (12.09)	44 (16.46)	< 5	< 5	39 (1.74)	127 (14.12)
		**IS**	< 5	< 5	16 (6.67)	19 (7.11)	< 5	< 5	20 (0.89)	70 (7.78)
		**TIA**	< 5	19 (0.34)	8 (3.34)	17 (6.36)	< 5	< 5	13 (0.58)	32 (3.56)
		**MI**	< 5	20 (0.36)	10 (4.17)	11 (4.11)	< 5	< 5	9 (0.40)	29 (3.22)
		**HF**	30 (0.87)	109 (1.97)	134 (55.88)	162 (60.59)	< 5	8 (0.68)	85 (3.80)	290 (32.24)
		**HS**	< 5	< 5	< 5	< 5	< 5	< 5	< 5	12 (1.33)
		**MP**	< 5	< 5	< 5	< 5	< 5	< 5	< 5	< 5
	**91 to 180 days**	**VTE**	10 (0.29)	18 (0.33)	16 (6.67)	29 (10.85)	< 5	< 5	18 (0.80)	35 (3.89)
		**DVT**	5 (0.14)	8 (0.14)	9 (3.75)	16 (5.98)	< 5	< 5	7 (0.31)	19 (2.11)
		**PE**	5 (0.14)	10 (0.18)	9 (3.75)	15 (5.61)	< 5	< 5	11 (0.49)	18 (2.00)
		**ATE**	11 (0.32)	28 (0.51)	28 (11.68)	45 (16.83)	< 5	6 (0.51)	25 (1.12)	104 (11.56)
		**IS**	< 5	8 (0.14)	13 (5.42)	19 (7.11)	< 5	< 5	13 (0.58)	57 (6.34)
		**TIA**	5 (0.14)	12 (0.22)	8 (3.34)	15 (5.61)	< 5	< 5	7 (0.31)	34 (3.78)
		**MI**	5 (0.14)	8 (0.14)	9 (3.75)	16 (5.98)	< 5	5 (0.42)	6 (0.27)	23 (2.56)
		**HF**	35 (1.01)	89 (1.61)	143 (59.63)	203 (75.93)	< 5	5 (0.42)	81 (3.62)	228 (25.35)
		**HS**	< 5	< 5	< 5	< 5	< 5	< 5	< 5	< 5
		**MP**	< 5	< 5	< 5	< 5	< 5	< 5	< 5	< 5

Mercadé-Besora N, *et al. Heart* 2024; 110:635–643. doi: 10.1136/heartjnl-2023-323483

Cohort	Time window	Outcome	AURUM		CORIVA		GOLD		SIDIAP	
			Unvaccinated	Vaccinated	Unvaccinated	Vaccinated	Unvaccinated	Vaccinated	Unvaccinated	Vaccinated
	**181 to 365 days**	**VTE**	9 (0.26)	11 (0.20)	29 (12.09)	27 (10.10)	< 5	< 5	5 (0.22)	11 (1.22)
		**DVT**	< 5	6 (0.11)	10 (4.17)	17 (6.36)	< 5	< 5	< 5	7 (0.78)
		**PE**	5 (0.14)	5 (0.09)	24 (10.01)	11 (4.11)	< 5	< 5	< 5	< 5
		**ATE**	10 (0.29)	23 (0.42)	45 (18.76)	74 (27.68)	< 5	< 5	35 (1.56)	39 (4.34)
		**IS**	< 5	6 (0.11)	26 (10.84)	34 (12.72)	< 5	< 5	18 (0.80)	24 (2.67)
		**TIA**	6 (0.17)	7 (0.13)	15 (6.25)	29 (10.85)	< 5	< 5	9 (0.40)	9 (1.00)
		**MI**	< 5	11 (0.20)	10 (4.17)	17 (6.36)	< 5	< 5	8 (0.36)	7 (0.78)
		**HF**	36 (1.04)	53 (0.96)	213 (88.82)	292 (109.22)	< 5	6 (0.51)	75 (3.35)	117 (13.01)
		**HS**	< 5	< 5	< 5	< 5	< 5	< 5	< 5	7 (0.78)
		**MP**	< 5	< 5	< 5	< 5	< 5	< 5	< 5	< 5
**Cohort 2**			**N = 1,975,726**	**N = 1,563,569**	**N = 34,317**	**N = 4,572**	**N = 583,399**	**N = 486,619**	**N = 433,151**	**N = 819,590**
	**0 to 30 days**	**VTE**	241 (1.22)	220 (1.41)	79 (23.02)	7 (15.31)	31 (0.53)	24 (0.49)	258 (5.96)	400 (4.88)
		**DVT**	41 (0.21)	65 (0.42)	12 (3.50)	< 5	8 (0.14)	5 (0.10)	63 (1.45)	165 (2.01)
		**PE**	204 (1.03)	165 (1.06)	69 (20.11)	5 (10.94)	24 (0.41)	19 (0.39)	213 (4.92)	269 (3.28)
		**ATE**	41 (0.21)	104 (0.67)	110 (32.05)	5 (10.94)	< 5	6 (0.12)	173 (3.99)	669 (8.16)
		**IS**	7 (0.04)	14 (0.09)	68 (19.82)	< 5	< 5	< 5	96 (2.22)	346 (4.22)
		**TIA**	8 (0.04)	24 (0.15)	18 (5.25)	< 5	< 5	< 5	17 (0.39)	122 (1.49)
		**MI**	26 (0.13)	68 (0.43)	35 (10.20)	< 5	< 5	6 (0.12)	64 (1.48)	239 (2.92)
		**HF**	45 (0.23)	146 (0.93)	364 (106.07)	23 (50.31)	5 (0.09)	13 (0.27)	378 (8.73)	1,331 (16.24)
		**HS**	6 (0.03)	5 (0.03)	< 5	< 5	< 5	< 5	18 (0.42)	77 (0.94)
		**MP**	12 (0.06)	7 (0.04)	< 5	< 5	< 5	< 5	14 (0.32)	37 (0.45)
	**31 to 90 days**	**VTE**	41 (0.21)	75 (0.48)	28 (8.16)	5 (10.94)	< 5	9 (0.18)	56 (1.29)	187 (2.28)
		**DVT**	23 (0.12)	32 (0.20)	15 (4.37)	5 (10.94)	< 5	5 (0.10)	30 (0.69)	119 (1.45)
		**PE**	22 (0.11)	45 (0.29)	16 (4.66)	< 5	< 5	< 5	31 (0.72)	87 (1.06)
		**ATE**	18 (0.09)	92 (0.59)	29 (8.45)	< 5	< 5	9 (0.18)	84 (1.94)	437 (5.33)
		**IS**	< 5	11 (0.07)	18 (5.25)	< 5	< 5	< 5	43 (0.99)	231 (2.82)
		**TIA**	< 5	33 (0.21)	< 5	< 5	< 5	6 (0.12)	23 (0.53)	115 (1.40)
		**MI**	13 (0.07)	50 (0.32)	11 (3.21)	< 5	< 5	< 5	25 (0.58)	113 (1.38)
		**HF**	27 (0.14)	99 (0.63)	137 (39.92)	19 (41.56)	< 5	7 (0.14)	132 (3.05)	622 (7.59)
		**HS**	< 5	< 5	< 5	< 5	< 5	< 5	7 (0.16)	27 (0.33)
		**MP**	6 (0.03)	< 5	< 5	< 5	< 5	< 5	< 5	8 (0.10)
	**91 to 180 days**	**VTE**	25 (0.13)	37 (0.24)	21 (6.12)	6 (13.12)	6 (0.10)	< 5	53 (1.22)	109 (1.33)
		**DVT**	12 (0.06)	21 (0.13)	10 (2.91)	< 5	< 5	< 5	29 (0.67)	71 (0.87)
		**PE**	13 (0.07)	19 (0.12)	12 (3.50)	< 5	< 5	< 5	27 (0.62)	46 (0.56)

Mercadé-Besora N, *et al. Heart* 2024; 110:635–643. doi: 10.1136/heartjnl-2023-323483

Cohort	Time window	Outcome	AURUM		CORIVA		GOLD		SIDIAP	
			Unvaccinated	Vaccinated	Unvaccinated	Vaccinated	Unvaccinated	Vaccinated	Unvaccinated	Vaccinated
		ATE	15 (0.08)	43 (0.28)	31 (9.03)	< 5	< 5	< 5	81 (1.87)	394 (4.81)
		IS	< 5	< 5	14 (4.08)	< 5	< 5	< 5	43 (0.99)	202 (2.46)
		TIA	8 (0.04)	18 (0.12)	11 (3.21)	< 5	< 5	< 5	19 (0.44)	109 (1.33)
		MI	8 (0.04)	25 (0.16)	8 (2.33)	< 5	< 5	< 5	22 (0.51)	104 (1.27)
		HF	22 (0.11)	65 (0.42)	149 (43.42)	20 (43.74)	< 5	< 5	100 (2.31)	534 (6.52)
		HS	< 5	< 5	< 5	< 5	< 5	< 5	7 (0.16)	35 (0.43)
		MP	< 5	< 5	< 5	< 5	< 5	< 5	7 (0.16)	9 (0.11)
	181 to 365 days	VTE	8 (0.04)	12 (0.08)	30 (8.74)	5 (10.94)	< 5	< 5	14 (0.32)	52 (0.63)
		DVT	< 5	5 (0.03)	15 (4.37)	< 5	< 5	< 5	8 (0.18)	36 (0.44)
		PE	< 5	8 (0.05)	18 (5.25)	< 5	< 5	< 5	7 (0.16)	19 (0.23)
		ATE	10 (0.05)	17 (0.11)	44 (12.82)	< 5	< 5	< 5	53 (1.22)	151 (1.84)
		IS	< 5	< 5	25 (7.29)	< 5	< 5	< 5	30 (0.69)	71 (0.87)
		TIA	< 5	5 (0.03)	12 (3.50)	< 5	< 5	< 5	14 (0.32)	46 (0.56)
		MI	6 (0.03)	10 (0.06)	11 (3.21)	< 5	< 5	< 5	11 (0.25)	45 (0.55)
		HF	19 (0.10)	34 (0.22)	210 (61.19)	29 (63.43)	< 5	< 5	72 (1.66)	209 (2.55)
		HS	< 5	< 5	< 5	< 5	< 5	< 5	< 5	10 (0.12)
		MP	< 5	< 5	< 5	< 5	< 5	< 5	< 5	< 5
**Cohort 3**			**N = 1,510,401**	**N = 1,528,031**	**N = 96,423**	**N = 24,050**	**N = 417,996**	**N = 462,832**	**N = 869,497**	**N = 954,232**
	0 to 30 days	VTE	245 (1.62)	142 (0.93)	115 (11.93)	9 (3.74)	27 (0.65)	17 (0.37)	325 (3.74)	180 (1.89)
		DVT	45 (0.30)	41 (0.27)	22 (2.28)	7 (2.91)	6 (0.14)	< 5	90 (1.04)	78 (0.82)
		PE	209 (1.38)	105 (0.69)	96 (9.96)	< 5	22 (0.53)	13 (0.28)	262 (3.01)	118 (1.24)
		ATE	29 (0.19)	49 (0.32)	119 (12.34)	12 (4.99)	< 5	12 (0.26)	213 (2.45)	275 (2.88)
		IS	< 5	5 (0.03)	70 (7.26)	9 (3.74)	< 5	< 5	102 (1.17)	130 (1.36)
		TIA	5 (0.03)	17 (0.11)	19 (1.97)	< 5	< 5	< 5	20 (0.23)	53 (0.56)
		MI	20 (0.13)	27 (0.18)	40 (4.15)	< 5	< 5	9 (0.19)	97 (1.12)	113 (1.18)
		HF	31 (0.21)	38 (0.25)	380 (39.41)	23 (9.56)	< 5	< 5	364 (4.19)	256 (2.68)
		HS	< 5	< 5	< 5	< 5	< 5	< 5	20 (0.23)	35 (0.37)
		MP	9 (0.06)	6 (0.04)	6 (0.62)	< 5	< 5	< 5	19 (0.22)	20 (0.21)
	31 to 90 days	VTE	41 (0.27)	46 (0.30)	45 (4.67)	10 (4.16)	< 5	6 (0.13)	81 (0.93)	90 (0.94)
		DVT	19 (0.13)	27 (0.18)	25 (2.59)	7 (2.91)	< 5	< 5	54 (0.62)	60 (0.63)
		PE	25 (0.17)	19 (0.12)	23 (2.39)	< 5	< 5	< 5	36 (0.41)	37 (0.39)
		ATE	11 (0.07)	33 (0.22)	45 (4.67)	8 (3.33)	< 5	8 (0.17)	108 (1.24)	207 (2.17)
		IS	< 5	< 5	22 (2.28)	< 5	< 5	< 5	54 (0.62)	87 (0.91)
		TIA	< 5	14 (0.09)	7 (0.73)	< 5	< 5	< 5	27 (0.31)	35 (0.37)

Mercadé-Besora N, *et al. Heart* 2024; 110:635–643. doi: 10.1136/heartjnl-2023-323483

Cohort	Time window	Outcome	AURUM		CORIVA		GOLD		SIDIAP	
			Unvaccinated	Vaccinated	Unvaccinated	Vaccinated	Unvaccinated	Vaccinated	Unvaccinated	Vaccinated
		**MI**	9 (0.06)	16 (0.10)	18 (1.87)	< 5	< 5	5 (0.11)	35 (0.40)	93 (0.97)
		**HF**	15 (0.10)	26 (0.17)	166 (17.22)	25 (10.40)	< 5	< 5	130 (1.50)	154 (1.61)
		**HS**	< 5	< 5	< 5	< 5	< 5	< 5	7 (0.08)	19 (0.20)
		**MP**	8 (0.05)	8 (0.05)	< 5	< 5	< 5	< 5	8 (0.09)	11 (0.12)
	**91 to 180 days**	**VTE**	23 (0.15)	25 (0.16)	34 (3.53)	11 (4.57)	< 5	< 5	57 (0.66)	92 (0.96)
		**DVT**	10 (0.07)	15 (0.10)	20 (2.07)	7 (2.91)	< 5	< 5	33 (0.38)	61 (0.64)
		**PE**	14 (0.09)	13 (0.09)	15 (1.56)	< 5	< 5	< 5	28 (0.32)	34 (0.36)
		**ATE**	< 5	28 (0.18)	40 (4.15)	8 (3.33)	< 5	< 5	102 (1.17)	199 (2.09)
		**IS**	< 5	< 5	20 (2.07)	< 5	< 5	< 5	49 (0.56)	92 (0.96)
		**TIA**	< 5	9 (0.06)	13 (1.35)	< 5	< 5	< 5	20 (0.23)	47 (0.49)
		**MI**	< 5	18 (0.12)	13 (1.35)	< 5	< 5	< 5	35 (0.40)	69 (0.72)
		**HF**	11 (0.07)	12 (0.08)	194 (20.12)	27 (11.23)	< 5	< 5	110 (1.27)	133 (1.39)
		**HS**	< 5	< 5	< 5	< 5	< 5	< 5	9 (0.10)	13 (0.14)
		**MP**	< 5	< 5	< 5	< 5	< 5	< 5	8 (0.09)	14 (0.15)
	**181 to 365 days**	**VTE**	< 5	9 (0.06)	50 (5.19)	16 (6.65)	< 5	< 5	31 (0.36)	19 (0.20)
		**DVT**	< 5	< 5	29 (3.01)	13 (5.41)	< 5	< 5	18 (0.21)	14 (0.15)
		**PE**	< 5	6 (0.04)	24 (2.49)	< 5	< 5	< 5	15 (0.17)	6 (0.06)
		**ATE**	< 5	< 5	66 (6.84)	7 (2.91)	< 5	< 5	42 (0.48)	58 (0.61)
		**IS**	< 5	< 5	30 (3.11)	< 5	< 5	< 5	19 (0.22)	22 (0.23)
		**TIA**	< 5	< 5	17 (1.76)	< 5	< 5	< 5	10 (0.12)	7 (0.07)
		**MI**	< 5	< 5	24 (2.49)	< 5	< 5	< 5	14 (0.16)	32 (0.34)
		**HF**	5 (0.03)	< 5	262 (27.17)	31 (12.89)	< 5	< 5	54 (0.62)	36 (0.38)
		**HS**	< 5	< 5	7 (0.73)	< 5	< 5	< 5	< 5	< 5
		**MP**	< 5	< 5	< 5	< 5	< 5	< 5	< 5	< 5
**Cohort 4**			**N = 2,027,763**	**N = 2,085,598**	**N = 147,545**	**N = 22,245**	**N = 469,876**	**N = 550,437**	**N = 1,061,634**	**N = 880,950**
	**0 to 30 days**	**VTE**	334 (1.65)	50 (0.24)	116 (7.86)	< 5	36 (0.77)	11 (0.20)	350 (3.30)	98 (1.11)
		**DVT**	55 (0.27)	17 (0.08)	22 (1.49)	< 5	6 (0.13)	< 5	106 (1.00)	48 (0.54)
		**PE**	291 (1.44)	34 (0.16)	97 (6.57)	< 5	31 (0.66)	8 (0.15)	272 (2.56)	55 (0.62)
		**ATE**	26 (0.13)	8 (0.04)	116 (7.86)	10 (4.50)	< 5	< 5	231 (2.18)	95 (1.08)
		**IS**	< 5	< 5	69 (4.68)	< 5	< 5	< 5	115 (1.08)	47 (0.53)
		**TIA**	6 (0.03)	< 5	18 (1.22)	< 5	< 5	< 5	26 (0.24)	12 (0.14)
		**MI**	16 (0.08)	< 5	38 (2.58)	< 5	< 5	< 5	96 (0.90)	39 (0.44)
		**HF**	28 (0.14)	< 5	364 (24.67)	17 (7.64)	< 5	< 5	362 (3.41)	75 (0.85)
		**HS**	< 5	< 5	5 (0.34)	< 5	< 5	< 5	23 (0.22)	13 (0.15)

Cohort	Time window	Outcome	AURUM		CORIVA		GOLD		SIDIAP	
			Unvaccinated	Vaccinated	Unvaccinated	Vaccinated	Unvaccinated	Vaccinated	Unvaccinated	Vaccinated
		**MP**	12 (0.06)	8 (0.04)	7 (0.47)	< 5	< 5	< 5	26 (0.24)	17 (0.19)
	**31 to 90 days**	**VTE**	55 (0.27)	22 (0.11)	49 (3.32)	< 5	5 (0.11)	< 5	86 (0.81)	48 (0.54)
		**DVT**	20 (0.10)	12 (0.06)	29 (1.97)	< 5	< 5	< 5	57 (0.54)	32 (0.36)
		**PE**	38 (0.19)	10 (0.05)	23 (1.56)	< 5	< 5	< 5	38 (0.36)	17 (0.19)
		**ATE**	12 (0.06)	9 (0.04)	43 (2.91)	5 (2.25)	< 5	< 5	117 (1.10)	75 (0.85)
		**IS**	< 5	< 5	22 (1.49)	< 5	< 5	< 5	52 (0.49)	30 (0.34)
		**TIA**	< 5	< 5	6 (0.41)	< 5	< 5	< 5	33 (0.31)	23 (0.26)
		**MI**	9 (0.04)	7 (0.03)	17 (1.15)	< 5	< 5	< 5	40 (0.38)	25 (0.28)
		**HF**	14 (0.07)	9 (0.04)	162 (10.98)	13 (5.84)	< 5	< 5	135 (1.27)	47 (0.53)
		**HS**	< 5	< 5	< 5	< 5	< 5	< 5	8 (0.08)	5 (0.06)
		**MP**	7 (0.03)	< 5	< 5	< 5	< 5	< 5	10 (0.09)	11 (0.12)
	**91 to 180 days**	**VTE**	25 (0.12)	9 (0.04)	40 (2.71)	< 5	< 5	< 5	65 (0.61)	52 (0.59)
		**DVT**	9 (0.04)	7 (0.03)	25 (1.69)	< 5	< 5	< 5	39 (0.37)	33 (0.37)
		**PE**	17 (0.08)	< 5	16 (1.08)	< 5	< 5	< 5	29 (0.27)	22 (0.25)
		**ATE**	< 5	6 (0.03)	38 (2.58)	6 (2.70)	< 5	< 5	116 (1.09)	86 (0.98)
		**IS**	< 5	< 5	18 (1.22)	< 5	< 5	< 5	57 (0.54)	42 (0.48)
		**TIA**	< 5	< 5	12 (0.81)	< 5	< 5	< 5	26 (0.24)	17 (0.19)
		**MI**	< 5	< 5	12 (0.81)	< 5	< 5	< 5	35 (0.33)	27 (0.31)
		**HF**	10 (0.05)	< 5	187 (12.67)	18 (8.09)	< 5	< 5	127 (1.20)	54 (0.61)
		**HS**	< 5	< 5	< 5	< 5	< 5	< 5	13 (0.12)	6 (0.07)
		**MP**	< 5	< 5	< 5	< 5	< 5	< 5	11 (0.10)	6 (0.07)
	**181 to 365 days**	**VTE**	< 5	< 5	53 (3.59)	< 5	< 5	< 5	42 (0.40)	8 (0.09)
		**DVT**	< 5	< 5	33 (2.24)	< 5	< 5	< 5	30 (0.28)	6 (0.07)
		**PE**	< 5	< 5	23 (1.56)	< 5	< 5	< 5	13 (0.12)	< 5
		**ATE**	< 5	< 5	64 (4.34)	9 (4.05)	< 5	< 5	42 (0.40)	16 (0.18)
		**IS**	< 5	< 5	29 (1.97)	5 (2.25)	< 5	< 5	20 (0.19)	11 (0.12)
		**TIA**	< 5	< 5	19 (1.29)	< 5	< 5	< 5	10 (0.09)	< 5
		**MI**	< 5	< 5	21 (1.42)	< 5	< 5	< 5	13 (0.12)	5 (0.06)
		**HF**	< 5	< 5	243 (16.47)	12 (5.39)	< 5	< 5	47 (0.44)	11 (0.12)
		**HS**	< 5	< 5	8 (0.54)	< 5	< 5	< 5	< 5	< 5
		**MP**	< 5	< 5	< 5	< 5	< 5	< 5	7 (0.07)	< 5

VTE = Venous thromboembolism (DVT + PE), DVT = Deep vein thrombosis, PE = Pulmonary embolism, ATE = Arterial thrombosis/thromboembolism (IS + TIA + MI), IS = Ischemic stroke, TIA = Transient ischemic attack, MI = Myocardial infarction, HF = Heart failure, HS = Hemorrhagic stroke, MP = Myocarditis or Pericarditis

Mercadé-Besora N, *et al. Heart* 2024; 110:635–643. doi: 10.1136/heartjnl-2023-323483

Table S28: Number of records (and risk per 10,000 individuals) for post COVID-19 cardiac and thromboembolic complications, across cohorts and databases, stratified by exposure status (ChAdOx1 vaccine).

Cohort	Time window	Outcome	AURUM		GOLD		SIDIAP	
			Unvaccinated	Vaccinated	Unvaccinated	Vaccinated	Unvaccinated	Vaccinated
Cohort 1			N = 344,687	N = 219,804	N = 168,972	N = 82,406		
	0 to 30 days	VTE	77 (2.23)	41 (1.87)	7 (0.41)	< 5		
		DVT	16 (0.46)	12 (0.55)	< 5	< 5		
		PE	64 (1.86)	31 (1.41)	6 (0.36)	< 5		
		ATE	18 (0.52)	25 (1.14)	7 (0.41)	6 (0.73)		
		IS	6 (0.17)	< 5	< 5	< 5		
		TIA	< 5	7 (0.32)	< 5	< 5		
		MI	9 (0.26)	17 (0.77)	5 (0.30)	< 5		
		HF	42 (1.22)	72 (3.28)	9 (0.53)	5 (0.61)		
		HS	< 5	< 5	< 5	< 5		
		MP	< 5	< 5	< 5	< 5		
	31 to 90 days	VTE	12 (0.35)	14 (0.64)	< 5	< 5		
		DVT	5 (0.15)	8 (0.36)	< 5	< 5		
		PE	7 (0.20)	7 (0.32)	< 5	< 5		
		ATE	6 (0.17)	20 (0.91)	< 5	< 5		
		IS	< 5	< 5	< 5	< 5		
		TIA	< 5	9 (0.41)	< 5	< 5		
		MI	5 (0.15)	9 (0.41)	< 5	< 5		
		HF	26 (0.75)	42 (1.91)	< 5	5 (0.61)		
		HS	< 5	< 5	< 5	< 5		
		MP	< 5	< 5	< 5	< 5		
	91 to 180 days	VTE	6 (0.17)	15 (0.68)	< 5	< 5		
		DVT	< 5	5 (0.23)	< 5	< 5		
		PE	< 5	10 (0.45)	< 5	< 5		
		ATE	10 (0.29)	10 (0.45)	< 5	6 (0.73)		
		IS	< 5	< 5	< 5	< 5		
		TIA	5 (0.15)	6 (0.27)	< 5	< 5		
		MI	< 5	< 5	< 5	5 (0.61)		
		HF	31 (0.90)	38 (1.73)	< 5	< 5		
		HS	< 5	< 5	< 5	< 5		
		MP	< 5	< 5	< 5	< 5		

Cohort	Time window	Outcome	AURUM		GOLD		SIDIAP	
			Unvaccinated	Vaccinated	Unvaccinated	Vaccinated	Unvaccinated	Vaccinated
	181 to 365 days	VTE	5 (0.15)	< 5	< 5	< 5		
		DVT	< 5	< 5	< 5	< 5		
		PE	< 5	< 5	< 5	< 5		
		ATE	9 (0.26)	9 (0.41)	< 5	< 5		
		IS	< 5	< 5	< 5	< 5		
		TIA	< 5	< 5	< 5	< 5		
		MI	< 5	< 5	< 5	< 5		
		HF	35 (1.02)	20 (0.91)	< 5	5 (0.61)		
		HS	< 5	< 5	< 5	< 5		
		MP	< 5	< 5	< 5	< 5		
Cohort 2			N = 1,975,770	N = 969,262	N = 582,791	N = 302,999	N = 433,636	N = 323,204
	0 to 30 days	VTE	243 (1.23)	158 (1.63)	33 (0.57)	19 (0.63)	262 (6.04)	90 (2.78)
		DVT	42 (0.21)	47 (0.48)	8 (0.14)	< 5	65 (1.50)	45 (1.39)
		PE	206 (1.04)	120 (1.24)	26 (0.45)	15 (0.50)	218 (5.03)	55 (1.70)
		ATE	38 (0.19)	75 (0.77)	< 5	< 5	177 (4.08)	131 (4.05)
		IS	6 (0.03)	12 (0.12)	< 5	< 5	97 (2.24)	52 (1.61)
		TIA	7 (0.04)	13 (0.13)	< 5	< 5	17 (0.39)	23 (0.71)
		MI	25 (0.13)	52 (0.54)	< 5	< 5	67 (1.55)	62 (1.92)
		HF	46 (0.23)	103 (1.06)	5 (0.09)	11 (0.36)	379 (8.74)	123 (3.81)
		HS	5 (0.03)	< 5	< 5	< 5	21 (0.48)	7 (0.22)
		MP	11 (0.06)	6 (0.06)	< 5	< 5	15 (0.35)	10 (0.31)
	31 to 90 days	VTE	49 (0.25)	55 (0.57)	< 5	9 (0.30)	60 (1.38)	36 (1.11)
		DVT	25 (0.13)	25 (0.26)	< 5	5 (0.17)	32 (0.74)	25 (0.77)
		PE	27 (0.14)	32 (0.33)	< 5	< 5	34 (0.78)	18 (0.56)
		ATE	16 (0.08)	57 (0.59)	< 5	5 (0.17)	86 (1.98)	107 (3.31)
		IS	< 5	6 (0.06)	< 5	< 5	42 (0.97)	58 (1.79)
		TIA	< 5	25 (0.26)	< 5	< 5	23 (0.53)	24 (0.74)
		MI	11 (0.06)	28 (0.29)	< 5	< 5	28 (0.65)	27 (0.84)
		HF	24 (0.12)	68 (0.70)	< 5	7 (0.23)	140 (3.23)	62 (1.92)
		HS	< 5	< 5	< 5	< 5	7 (0.16)	7 (0.22)
		MP	6 (0.03)	< 5	< 5	< 5	< 5	< 5
	91 to 180 days	VTE	25 (0.13)	30 (0.31)	< 5	< 5	58 (1.34)	23 (0.71)
		DVT	11 (0.06)	16 (0.17)	< 5	< 5	31 (0.71)	16 (0.50)
		PE	14 (0.07)	16 (0.17)	< 5	< 5	29 (0.67)	8 (0.25)

Cohort	Time window	Outcome	AURUM		GOLD		SIDIAP	
			Unvaccinated	Vaccinated	Unvaccinated	Vaccinated	Unvaccinated	Vaccinated
		**ATE**	12 (0.06)	28 (0.29)	< 5	< 5	94 (2.17)	106 (3.28)
		**IS**	< 5	< 5	< 5	< 5	51 (1.18)	40 (1.24)
		**TIA**	7 (0.04)	13 (0.13)	< 5	< 5	21 (0.48)	32 (0.99)
		**MI**	6 (0.03)	15 (0.15)	< 5	< 5	25 (0.58)	37 (1.14)
		**HF**	16 (0.08)	44 (0.45)	< 5	< 5	115 (2.65)	60 (1.86)
		**HS**	< 5	< 5	< 5	< 5	8 (0.18)	< 5
		**MP**	< 5	< 5	< 5	< 5	8 (0.18)	< 5
	**181 to 365 days**	**VTE**	14 (0.07)	5 (0.05)	< 5	< 5	18 (0.42)	16 (0.50)
		**DVT**	< 5	< 5	< 5	< 5	10 (0.23)	13 (0.40)
		**PE**	10 (0.05)	< 5	< 5	< 5	9 (0.21)	< 5
		**ATE**	11 (0.06)	11 (0.11)	< 5	< 5	71 (1.64)	46 (1.42)
		**IS**	< 5	< 5	< 5	< 5	38 (0.88)	21 (0.65)
		**TIA**	< 5	< 5	< 5	< 5	20 (0.46)	13 (0.40)
		**MI**	8 (0.04)	7 (0.07)	< 5	< 5	15 (0.35)	16 (0.50)
		**HF**	18 (0.09)	23 (0.24)	< 5	< 5	81 (1.87)	26 (0.80)
		**HS**	< 5	< 5	< 5	< 5	5 (0.12)	< 5
		**MP**	< 5	< 5	< 5	< 5	< 5	< 5
**Cohort 3**			**N = 1,510,493**	**N = 1,473,602**	**N = 418,184**	**N = 423,876**	**N = 873,400**	**N = 84,204**
	**0 to 30 days**	**VTE**	244 (1.62)	139 (0.94)	28 (0.67)	16 (0.38)	332 (3.80)	27 (3.21)
		**DVT**	44 (0.29)	41 (0.28)	6 (0.14)	< 5	95 (1.09)	9 (1.07)
		**PE**	209 (1.38)	101 (0.69)	23 (0.55)	12 (0.28)	266 (3.05)	22 (2.61)
		**ATE**	28 (0.19)	47 (0.32)	< 5	12 (0.28)	216 (2.47)	32 (3.80)
		**IS**	< 5	5 (0.03)	< 5	< 5	103 (1.18)	13 (1.54)
		**TIA**	5 (0.03)	16 (0.11)	< 5	< 5	22 (0.25)	5 (0.59)
		**MI**	19 (0.13)	26 (0.18)	< 5	9 (0.21)	97 (1.11)	16 (1.90)
		**HF**	30 (0.20)	28 (0.19)	< 5	< 5	372 (4.26)	51 (6.06)
		**HS**	< 5	< 5	< 5	< 5	20 (0.23)	< 5
		**MP**	9 (0.06)	6 (0.04)	< 5	< 5	20 (0.23)	6 (0.71)
	**31 to 90 days**	**VTE**	46 (0.30)	44 (0.30)	< 5	7 (0.17)	85 (0.97)	9 (1.07)
		**DVT**	21 (0.14)	26 (0.18)	< 5	< 5	57 (0.65)	< 5
		**PE**	28 (0.19)	18 (0.12)	< 5	< 5	38 (0.44)	6 (0.71)
		**ATE**	11 (0.07)	31 (0.21)	< 5	7 (0.17)	109 (1.25)	25 (2.97)
		**IS**	< 5	< 5	< 5	< 5	52 (0.60)	11 (1.31)
		**TIA**	< 5	13 (0.09)	< 5	< 5	27 (0.31)	< 5

Cohort	Time window	Outcome	AURUM		GOLD		SIDIAP	
			Unvaccinated	Vaccinated	Unvaccinated	Vaccinated	Unvaccinated	Vaccinated
		MI	9 (0.06)	16 (0.11)	< 5	< 5	38 (0.44)	10 (1.19)
		HF	14 (0.09)	23 (0.16)	< 5	< 5	142 (1.63)	25 (2.97)
		HS	< 5	< 5	< 5	< 5	7 (0.08)	< 5
		MP	8 (0.05)	7 (0.05)	< 5	< 5	9 (0.10)	< 5
	91 to 180 days	VTE	24 (0.16)	26 (0.18)	< 5	< 5	65 (0.74)	14 (1.66)
		DVT	11 (0.07)	15 (0.10)	< 5	< 5	36 (0.41)	7 (0.83)
		PE	14 (0.09)	14 (0.10)	< 5	< 5	33 (0.38)	8 (0.95)
		ATE	< 5	26 (0.18)	< 5	< 5	115 (1.32)	30 (3.56)
		IS	< 5	< 5	< 5	< 5	58 (0.66)	14 (1.66)
		TIA	< 5	8 (0.05)	< 5	< 5	22 (0.25)	10 (1.19)
		MI	< 5	17 (0.12)	< 5	< 5	38 (0.44)	8 (0.95)
		HF	10 (0.07)	12 (0.08)	< 5	< 5	122 (1.40)	12 (1.43)
		HS	< 5	< 5	< 5	< 5	9 (0.10)	< 5
		MP	< 5	< 5	< 5	< 5	8 (0.09)	< 5
	181 to 365 days	VTE	< 5	11 (0.07)	< 5	< 5	35 (0.40)	7 (0.83)
		DVT	< 5	5 (0.03)	< 5	< 5	21 (0.24)	< 5
		PE	< 5	7 (0.05)	< 5	< 5	16 (0.18)	5 (0.59)
		ATE	< 5	< 5	< 5	< 5	57 (0.65)	15 (1.78)
		IS	< 5	< 5	< 5	< 5	26 (0.30)	6 (0.71)
		TIA	< 5	< 5	< 5	< 5	12 (0.14)	< 5
		MI	< 5	< 5	< 5	< 5	20 (0.23)	8 (0.95)
		HF	< 5	< 5	< 5	< 5	64 (0.73)	6 (0.71)
		HS	< 5	< 5	< 5	< 5	< 5	< 5
		MP	< 5	< 5	< 5	< 5	< 5	< 5
Cohort 4			N = 2,066,318	N = 542,670	N = 485,154	N = 147,744		
	0 to 30 days	VTE	346 (1.67)	27 (0.50)	38 (0.78)	8 (0.54)		
		DVT	56 (0.27)	7 (0.13)	6 (0.12)	< 5		
		PE	302 (1.46)	21 (0.39)	33 (0.68)	6 (0.41)		
		ATE	26 (0.13)	5 (0.09)	< 5	< 5		
		IS	< 5	< 5	< 5	< 5		
		TIA	5 (0.02)	< 5	< 5	< 5		
		MI	17 (0.08)	< 5	< 5	< 5		
		HF	28 (0.14)	< 5	< 5	< 5		
		HS	< 5	< 5	< 5	< 5		

Mercadé-Besora N, *et al. Heart* 2024; 110:635–643. doi: 10.1136/heartjnl-2023-323483

Cohort	Time window	Outcome	AURUM		GOLD		SIDIAP	
			Unvaccinated	Vaccinated	Unvaccinated	Vaccinated	Unvaccinated	Vaccinated
		MP	14 (0.07)	< 5	< 5	< 5		
	31 to 90 days	VTE	60 (0.29)	14 (0.26)	< 5	< 5		
		DVT	23 (0.11)	8 (0.15)	< 5	< 5		
		PE	40 (0.19)	6 (0.11)	< 5	< 5		
		ATE	13 (0.06)	< 5	< 5	< 5		
		IS	< 5	< 5	< 5	< 5		
		TIA	< 5	< 5	< 5	< 5		
		MI	9 (0.04)	< 5	< 5	< 5		
		HF	14 (0.07)	< 5	< 5	< 5		
		HS	< 5	< 5	< 5	< 5		
		MP	7 (0.03)	< 5	< 5	< 5		
	91 to 180 days	VTE	29 (0.14)	< 5	< 5	< 5		
		DVT	10 (0.05)	< 5	< 5	< 5		
		PE	20 (0.10)	< 5	< 5	< 5		
		ATE	< 5	< 5	< 5	< 5		
		IS	< 5	< 5	< 5	< 5		
		TIA	< 5	< 5	< 5	< 5		
		MI	< 5	< 5	< 5	< 5		
		HF	10 (0.05)	< 5	< 5	< 5		
		HS	< 5	< 5	< 5	< 5		
		MP	< 5	< 5	< 5	< 5		
	181 to 365 days	VTE	< 5	< 5	< 5	< 5		
		DVT	< 5	< 5	< 5	< 5		
		PE	< 5	< 5	< 5	< 5		
		ATE	< 5	< 5	< 5	< 5		
		IS	< 5	< 5	< 5	< 5		
		TIA	< 5	< 5	< 5	< 5		
		MI	< 5	< 5	< 5	< 5		
		HF	< 5	< 5	< 5	< 5		
		HS	< 5	< 5	< 5	< 5		
		MP	< 5	< 5	< 5	< 5		

VTE = Venous thromboembolism (DVT + PE), DVT = Deep vein thrombosis, PE = Pulmonary embolism, ATE = Arterial thrombosis/thromboembolism (IS + TIA + MI), IS = Ischemic stroke, TIA = Transient ischemic attack, MI = Myocardial infarction, HF = Heart failure, HS = Hemorrhagic stroke, MP = Myocarditis or Pericarditis

Mercadé-Besora N, *et al. Heart* 2024; 110:635–643. doi: 10.1136/heartjnl-2023-323483

Table S29: Number of records (and risk per 10,000 individuals) for post COVID-19 cardiac and thromboembolic complications, across cohorts and databases, stratified by exposure status (ChAdOx1 vaccine). Follow-up ends at first vaccine dose after index date.

Cohort	Time window	Outcome	AURUM		GOLD		SIDIAP	
			Unvaccinated	Vaccinated	Unvaccinated	Vaccinated	Unvaccinated	Vaccinated
Cohort 1			N = 344,687	N = 219,804	N = 168,972	N = 82,406		
	0 to 30 days	VTE	77 (2.23)	14 (0.64)	7 (0.41)	< 5		
		DVT	16 (0.46)	< 5	< 5	< 5		
		PE	64 (1.86)	13 (0.59)	6 (0.36)	< 5		
		ATE	18 (0.52)	10 (0.45)	7 (0.41)	< 5		
		IS	6 (0.17)	< 5	< 5	< 5		
		TIA	< 5	< 5	< 5	< 5		
		MI	9 (0.26)	6 (0.27)	5 (0.30)	< 5		
		HF	42 (1.22)	28 (1.27)	9 (0.53)	< 5		
		HS	< 5	< 5	< 5	< 5		
		MP	< 5	< 5	< 5	< 5		
	31 to 90 days	VTE	12 (0.35)	5 (0.23)	< 5	< 5		
		DVT	5 (0.15)	< 5	< 5	< 5		
		PE	7 (0.20)	< 5	< 5	< 5		
		ATE	6 (0.17)	8 (0.36)	< 5	< 5		
		IS	< 5	< 5	< 5	< 5		
		TIA	< 5	< 5	< 5	< 5		
		MI	5 (0.15)	< 5	< 5	< 5		
		HF	26 (0.75)	20 (0.91)	< 5	< 5		
		HS	< 5	< 5	< 5	< 5		
		MP	< 5	< 5	< 5	< 5		
	91 to 180 days	VTE	6 (0.17)	5 (0.23)	< 5	< 5		
		DVT	< 5	< 5	< 5	< 5		
		PE	< 5	< 5	< 5	< 5		
		ATE	10 (0.29)	< 5	< 5	< 5		
		IS	< 5	< 5	< 5	< 5		
		TIA	5 (0.15)	< 5	< 5	< 5		
		MI	< 5	< 5	< 5	< 5		
		HF	31 (0.90)	19 (0.86)	< 5	< 5		
		HS	< 5	< 5	< 5	< 5		
		MP	< 5	< 5	< 5	< 5		

Mercadé-Besora N, *et al. Heart* 2024; 110:635–643. doi: 10.1136/heartjnl-2023-323483

Cohort	Time window	Outcome	AURUM		GOLD		SIDIAP	
			Unvaccinated	Vaccinated	Unvaccinated	Vaccinated	Unvaccinated	Vaccinated
	181 to 365 days	VTE	5 (0.15)	< 5	< 5	< 5		
		DVT	< 5	< 5	< 5	< 5		
		PE	< 5	< 5	< 5	< 5		
		ATE	9 (0.26)	8 (0.36)	< 5	< 5		
		IS	< 5	< 5	< 5	< 5		
		TIA	< 5	< 5	< 5	< 5		
		MI	< 5	< 5	< 5	< 5		
		HF	35 (1.02)	18 (0.82)	< 5	5 (0.61)		
		HS	< 5	< 5	< 5	< 5		
		MP	< 5	< 5	< 5	< 5		
Cohort 2			N = 1,975,770	N = 969,262	N = 582,791	N = 302,999	N = 433,636	N = 323,204
	0 to 30 days	VTE	243 (1.23)	40 (0.41)	33 (0.57)	< 5	262 (6.04)	9 (0.28)
		DVT	42 (0.21)	13 (0.13)	8 (0.14)	< 5	65 (1.50)	5 (0.15)
		PE	206 (1.04)	30 (0.31)	26 (0.45)	< 5	218 (5.03)	6 (0.19)
		ATE	38 (0.19)	11 (0.11)	< 5	< 5	177 (4.08)	6 (0.19)
		IS	6 (0.03)	< 5	< 5	< 5	97 (2.24)	< 5
		TIA	7 (0.04)	< 5	< 5	< 5	17 (0.39)	< 5
		MI	25 (0.13)	9 (0.09)	< 5	< 5	67 (1.55)	< 5
		HF	46 (0.23)	32 (0.33)	5 (0.09)	< 5	379 (8.74)	12 (0.37)
		HS	5 (0.03)	< 5	< 5	< 5	21 (0.48)	< 5
		MP	11 (0.06)	< 5	< 5	< 5	15 (0.35)	< 5
	31 to 90 days	VTE	49 (0.25)	8 (0.08)	< 5	< 5	60 (1.38)	< 5
		DVT	25 (0.13)	5 (0.05)	< 5	< 5	32 (0.74)	< 5
		PE	27 (0.14)	< 5	< 5	< 5	34 (0.78)	< 5
		ATE	16 (0.08)	9 (0.09)	< 5	< 5	86 (1.98)	6 (0.19)
		IS	< 5	< 5	< 5	< 5	42 (0.97)	< 5
		TIA	< 5	< 5	< 5	< 5	23 (0.53)	< 5
		MI	11 (0.06)	6 (0.06)	< 5	< 5	28 (0.65)	< 5
		HF	24 (0.12)	13 (0.13)	< 5	< 5	140 (3.23)	7 (0.22)
		HS	< 5	< 5	< 5	< 5	7 (0.16)	< 5
		MP	6 (0.03)	< 5	< 5	< 5	< 5	< 5
	91 to 180 days	VTE	25 (0.13)	12 (0.12)	< 5	< 5	58 (1.34)	< 5
		DVT	11 (0.06)	7 (0.07)	< 5	< 5	31 (0.71)	< 5
		PE	14 (0.07)	5 (0.05)	< 5	< 5	29 (0.67)	< 5

Mercadé-Besora N, *et al. Heart* 2024; 110:635–643. doi: 10.1136/heartjnl-2023-323483

Cohort	Time window	Outcome	AURUM		GOLD		SIDIAP	
			Unvaccinated	Vaccinated	Unvaccinated	Vaccinated	Unvaccinated	Vaccinated
		ATE	12 (0.06)	10 (0.10)	< 5	< 5	94 (2.17)	6 (0.19)
		IS	< 5	< 5	< 5	< 5	51 (1.18)	< 5
		TIA	7 (0.04)	< 5	< 5	< 5	21 (0.48)	< 5
		MI	6 (0.03)	6 (0.06)	< 5	< 5	25 (0.58)	< 5
		HF	16 (0.08)	18 (0.19)	< 5	< 5	115 (2.65)	< 5
		HS	< 5	< 5	< 5	< 5	8 (0.18)	< 5
		MP	< 5	< 5	< 5	< 5	8 (0.18)	< 5
	181 to 365 days	VTE	14 (0.07)	5 (0.05)	< 5	< 5	18 (0.42)	9 (0.28)
		DVT	< 5	< 5	< 5	< 5	10 (0.23)	7 (0.22)
		PE	10 (0.05)	< 5	< 5	< 5	9 (0.21)	< 5
		ATE	11 (0.06)	10 (0.10)	< 5	< 5	71 (1.64)	10 (0.31)
		IS	< 5	< 5	< 5	< 5	38 (0.88)	< 5
		TIA	< 5	< 5	< 5	< 5	20 (0.46)	< 5
		MI	8 (0.04)	6 (0.06)	< 5	< 5	15 (0.35)	5 (0.15)
		HF	18 (0.09)	20 (0.21)	< 5	< 5	81 (1.87)	9 (0.28)
		HS	< 5	< 5	< 5	< 5	5 (0.12)	< 5
		MP	< 5	< 5	< 5	< 5	< 5	< 5
Cohort 3			N = 1,510,493	N = 1,473,602	N = 418,184	N = 423,876	N = 873,400	N = 84,204
	0 to 30 days	VTE	244 (1.62)	23 (0.16)	28 (0.67)	< 5	332 (3.80)	5 (0.59)
		DVT	44 (0.29)	< 5	6 (0.14)	< 5	95 (1.09)	< 5
		PE	209 (1.38)	19 (0.13)	23 (0.55)	< 5	266 (3.05)	< 5
		ATE	28 (0.19)	8 (0.05)	< 5	< 5	216 (2.47)	< 5
		IS	< 5	< 5	< 5	< 5	103 (1.18)	< 5
		TIA	5 (0.03)	< 5	< 5	< 5	22 (0.25)	< 5
		MI	19 (0.13)	7 (0.05)	< 5	< 5	97 (1.11)	< 5
		HF	30 (0.20)	11 (0.07)	< 5	< 5	372 (4.26)	5 (0.59)
		HS	< 5	< 5	< 5	< 5	20 (0.23)	< 5
		MP	9 (0.06)	< 5	< 5	< 5	20 (0.23)	< 5
	31 to 90 days	VTE	46 (0.30)	< 5	< 5	< 5	85 (0.97)	< 5
		DVT	21 (0.14)	< 5	< 5	< 5	57 (0.65)	< 5
		PE	28 (0.19)	< 5	< 5	< 5	38 (0.44)	< 5
		ATE	11 (0.07)	6 (0.04)	< 5	< 5	109 (1.25)	< 5
		IS	< 5	< 5	< 5	< 5	52 (0.60)	< 5
		TIA	< 5	< 5	< 5	< 5	27 (0.31)	< 5

Cohort	Time window	Outcome	AURUM		GOLD		SIDIAP	
			Unvaccinated	Vaccinated	Unvaccinated	Vaccinated	Unvaccinated	Vaccinated
		**MI**	9 (0.06)	< 5	< 5	< 5	38 (0.44)	< 5
		**HF**	14 (0.09)	5 (0.03)	< 5	< 5	142 (1.63)	< 5
		**HS**	< 5	< 5	< 5	< 5	7 (0.08)	< 5
		**MP**	8 (0.05)	< 5	< 5	< 5	9 (0.10)	< 5
	**91 to 180 days**	**VTE**	24 (0.16)	8 (0.05)	< 5	< 5	65 (0.74)	< 5
		**DVT**	11 (0.07)	6 (0.04)	< 5	< 5	36 (0.41)	< 5
		**PE**	14 (0.09)	< 5	< 5	< 5	33 (0.38)	< 5
		**ATE**	< 5	7 (0.05)	< 5	< 5	115 (1.32)	6 (0.71)
		**IS**	< 5	< 5	< 5	< 5	58 (0.66)	5 (0.59)
		**TIA**	< 5	< 5	< 5	< 5	22 (0.25)	< 5
		**MI**	< 5	< 5	< 5	< 5	38 (0.44)	< 5
		**HF**	10 (0.07)	< 5	< 5	< 5	122 (1.40)	< 5
		**HS**	< 5	< 5	< 5	< 5	9 (0.10)	< 5
		**MP**	< 5	< 5	< 5	< 5	8 (0.09)	< 5
	**181 to 365 days**	**VTE**	< 5	9 (0.06)	< 5	< 5	35 (0.40)	< 5
		**DVT**	< 5	< 5	< 5	< 5	21 (0.24)	< 5
		**PE**	< 5	6 (0.04)	< 5	< 5	16 (0.18)	< 5
		**ATE**	< 5	< 5	< 5	< 5	57 (0.65)	8 (0.95)
		**IS**	< 5	< 5	< 5	< 5	26 (0.30)	< 5
		**TIA**	< 5	< 5	< 5	< 5	12 (0.14)	< 5
		**MI**	< 5	< 5	< 5	< 5	20 (0.23)	< 5
		**HF**	< 5	< 5	< 5	< 5	64 (0.73)	5 (0.59)
		**HS**	< 5	< 5	< 5	< 5	< 5	< 5
		**MP**	< 5	< 5	< 5	< 5	< 5	< 5
**Cohort 4**			**N = 2,066,318**	**N = 542,670**	**N = 485,154**	**N = 147,744**		
	**0 to 30 days**	**VTE**	346 (1.67)	8 (0.15)	38 (0.78)	6 (0.41)		
		**DVT**	56 (0.27)	< 5	6 (0.12)	< 5		
		**PE**	302 (1.46)	7 (0.13)	33 (0.68)	6 (0.41)		
		**ATE**	26 (0.13)	< 5	< 5	< 5		
		**IS**	< 5	< 5	< 5	< 5		
		**TIA**	5 (0.02)	< 5	< 5	< 5		
		**MI**	17 (0.08)	< 5	< 5	< 5		
		**HF**	28 (0.14)	< 5	< 5	< 5		
		**HS**	< 5	< 5	< 5	< 5		

Mercadé-Besora N, *et al. Heart* 2024; 110:635–643. doi: 10.1136/heartjnl-2023-323483

Cohort	Time window	Outcome	AURUM		GOLD		SIDIAP	
			Unvaccinated	Vaccinated	Unvaccinated	Vaccinated	Unvaccinated	Vaccinated
		**MP**	14 (0.07)	< 5	< 5	< 5		
	**31 to 90 days**	**VTE**	60 (0.29)	8 (0.15)	< 5	< 5		
		**DVT**	23 (0.11)	< 5	< 5	< 5		
		**PE**	40 (0.19)	5 (0.09)	< 5	< 5		
		**ATE**	13 (0.06)	< 5	< 5	< 5		
		**IS**	< 5	< 5	< 5	< 5		
		**TIA**	< 5	< 5	< 5	< 5		
		**MI**	9 (0.04)	< 5	< 5	< 5		
		**HF**	14 (0.07)	< 5	< 5	< 5		
		**HS**	< 5	< 5	< 5	< 5		
		**MP**	7 (0.03)	< 5	< 5	< 5		
	**91 to 180 days**	**VTE**	29 (0.14)	< 5	< 5	< 5		
		**DVT**	10 (0.05)	< 5	< 5	< 5		
		**PE**	20 (0.10)	< 5	< 5	< 5		
		**ATE**	< 5	< 5	< 5	< 5		
		**IS**	< 5	< 5	< 5	< 5		
		**TIA**	< 5	< 5	< 5	< 5		
		**MI**	< 5	< 5	< 5	< 5		
		**HF**	10 (0.05)	< 5	< 5	< 5		
		**HS**	< 5	< 5	< 5	< 5		
		**MP**	< 5	< 5	< 5	< 5		
	**181 to 365 days**	**VTE**	< 5	< 5	< 5	< 5		
		**DVT**	< 5	< 5	< 5	< 5		
		**PE**	< 5	< 5	< 5	< 5		
		**ATE**	< 5	< 5	< 5	< 5		
		**IS**	< 5	< 5	< 5	< 5		
		**TIA**	< 5	< 5	< 5	< 5		
		**MI**	< 5	< 5	< 5	< 5		
		**HF**	< 5	< 5	< 5	< 5		
		**HS**	< 5	< 5	< 5	< 5		
		**MP**	< 5	< 5	< 5	< 5		

VTE = Venous thromboembolism (DVT + PE), DVT = Deep vein thrombosis, PE = Pulmonary embolism, ATE = Arterial thrombosis/thromboembolism (IS + TIA + MI), IS = Ischemic stroke, TIA = Transient ischemic attack, MI = Myocardial infarction, HF = Heart failure, HS = Hemorrhagic stroke, MP = Myocarditis or Pericarditis

Mercadé-Besora N, *et al. Heart* 2024; 110:635–643. doi: 10.1136/heartjnl-2023-323483

Table S30: Number of records (and risk per 10,000 individuals) for post COVID-19 cardiac and thromboembolic complications, across cohorts and databases, stratified by exposure status (ChAdOx1 vaccine). Only first outcome after COVID-19 captured.

Cohort	Time window	Outcome	AURUM		GOLD		SIDIAP	
			Unvaccinated	Vaccinated	Unvaccinated	Vaccinated	Unvaccinated	Vaccinated
Cohort 1			N = 344,687	N = 219,804	N = 168,972	N = 82,406		
	0 to 30 days	VTE	77 (2.23)	41 (1.87)	7 (0.41)	< 5		
		DVT	16 (0.46)	12 (0.55)	< 5	< 5		
		PE	64 (1.86)	31 (1.41)	6 (0.36)	< 5		
		ATE	18 (0.52)	25 (1.14)	7 (0.41)	6 (0.73)		
		IS	6 (0.17)	< 5	< 5	< 5		
		TIA	< 5	7 (0.32)	< 5	< 5		
		MI	9 (0.26)	17 (0.77)	5 (0.30)	< 5		
		HF	42 (1.22)	72 (3.28)	9 (0.53)	5 (0.61)		
		HS	< 5	< 5	< 5	< 5		
		MP	< 5	< 5	< 5	< 5		
	31 to 90 days	VTE	12 (0.35)	14 (0.64)	< 5	< 5		
		DVT	5 (0.15)	8 (0.36)	< 5	< 5		
		PE	7 (0.20)	7 (0.32)	< 5	< 5		
		ATE	6 (0.17)	20 (0.91)	< 5	< 5		
		IS	< 5	< 5	< 5	< 5		
		TIA	< 5	9 (0.41)	< 5	< 5		
		MI	5 (0.15)	9 (0.41)	< 5	< 5		
		HF	26 (0.75)	39 (1.77)	< 5	5 (0.61)		
		HS	< 5	< 5	< 5	< 5		
		MP	< 5	< 5	< 5	< 5		
	91 to 180 days	VTE	6 (0.17)	15 (0.68)	< 5	< 5		
		DVT	< 5	5 (0.23)	< 5	< 5		
		PE	< 5	10 (0.45)	< 5	< 5		
		ATE	10 (0.29)	10 (0.45)	< 5	6 (0.73)		
		IS	< 5	< 5	< 5	< 5		
		TIA	5 (0.15)	6 (0.27)	< 5	< 5		
		MI	< 5	< 5	< 5	5 (0.61)		
		HF	28 (0.81)	35 (1.59)	< 5	< 5		
		HS	< 5	< 5	< 5	< 5		
		MP	< 5	< 5	< 5	< 5		

Mercadé-Besora N, *et al. Heart* 2024; 110:635–643. doi: 10.1136/heartjnl-2023-323483

Cohort	Time window	Outcome	AURUM		GOLD		SIDIAP	
			Unvaccinated	Vaccinated	Unvaccinated	Vaccinated	Unvaccinated	Vaccinated
	181 to 365 days	VTE	< 5	< 5	< 5	< 5		
		DVT	< 5	< 5	< 5	< 5		
		PE	< 5	< 5	< 5	< 5		
		ATE	9 (0.26)	9 (0.41)	< 5	< 5		
		IS	< 5	< 5	< 5	< 5		
		TIA	< 5	< 5	< 5	< 5		
		MI	< 5	< 5	< 5	< 5		
		HF	32 (0.93)	18 (0.82)	< 5	5 (0.61)		
		HS	< 5	< 5	< 5	< 5		
		MP	< 5	< 5	< 5	< 5		
Cohort 2			N = 1,975,770	N = 969,262	N = 582,791	N = 302,999	N = 433,636	N = 323,204
	0 to 30 days	VTE	243 (1.23)	158 (1.63)	33 (0.57)	19 (0.63)	262 (6.04)	90 (2.78)
		DVT	42 (0.21)	47 (0.48)	8 (0.14)	< 5	65 (1.50)	45 (1.39)
		PE	206 (1.04)	120 (1.24)	26 (0.45)	15 (0.50)	218 (5.03)	55 (1.70)
		ATE	38 (0.19)	75 (0.77)	< 5	< 5	177 (4.08)	131 (4.05)
		IS	6 (0.03)	12 (0.12)	< 5	< 5	97 (2.24)	52 (1.61)
		TIA	7 (0.04)	13 (0.13)	< 5	< 5	17 (0.39)	23 (0.71)
		MI	25 (0.13)	52 (0.54)	< 5	< 5	67 (1.55)	62 (1.92)
		HF	46 (0.23)	103 (1.06)	5 (0.09)	11 (0.36)	379 (8.74)	123 (3.81)
		HS	5 (0.03)	< 5	< 5	< 5	21 (0.48)	7 (0.22)
		MP	11 (0.06)	6 (0.06)	< 5	< 5	15 (0.35)	10 (0.31)
	31 to 90 days	VTE	46 (0.23)	54 (0.56)	< 5	9 (0.30)	57 (1.31)	34 (1.05)
		DVT	24 (0.12)	25 (0.26)	< 5	5 (0.17)	30 (0.69)	23 (0.71)
		PE	25 (0.13)	31 (0.32)	< 5	< 5	32 (0.74)	16 (0.50)
		ATE	16 (0.08)	56 (0.58)	< 5	5 (0.17)	84 (1.94)	106 (3.28)
		IS	< 5	6 (0.06)	< 5	< 5	42 (0.97)	57 (1.76)
		TIA	< 5	24 (0.25)	< 5	< 5	23 (0.53)	24 (0.74)
		MI	11 (0.06)	28 (0.29)	< 5	< 5	26 (0.60)	27 (0.84)
		HF	24 (0.12)	65 (0.67)	< 5	7 (0.23)	134 (3.09)	61 (1.89)
		HS	< 5	< 5	< 5	< 5	7 (0.16)	7 (0.22)
		MP	6 (0.03)	< 5	< 5	< 5	< 5	< 5
	91 to 180 days	VTE	22 (0.11)	28 (0.29)	< 5	< 5	52 (1.20)	21 (0.65)
		DVT	10 (0.05)	15 (0.15)	< 5	< 5	28 (0.65)	16 (0.50)
		PE	12 (0.06)	15 (0.15)	< 5	< 5	26 (0.60)	6 (0.19)

Mercadé-Besora N, *et al. Heart* 2024; 110:635–643. doi: 10.1136/heartjnl-2023-323483

Cohort	Time window	Outcome	AURUM		GOLD		SIDIAP	
			Unvaccinated	Vaccinated	Unvaccinated	Vaccinated	Unvaccinated	Vaccinated
		**ATE**	11 (0.06)	28 (0.29)	< 5	< 5	83 (1.91)	106 (3.28)
		**IS**	< 5	< 5	< 5	< 5	45 (1.04)	40 (1.24)
		**TIA**	7 (0.04)	13 (0.13)	< 5	< 5	20 (0.46)	32 (0.99)
		**MI**	5 (0.03)	15 (0.15)	< 5	< 5	21 (0.48)	37 (1.14)
		**HF**	16 (0.08)	41 (0.42)	< 5	< 5	105 (2.42)	54 (1.67)
		**HS**	< 5	< 5	< 5	< 5	7 (0.16)	< 5
		**MP**	< 5	< 5	< 5	< 5	7 (0.16)	< 5
	**181 to 365 days**	**VTE**	14 (0.07)	< 5	< 5	< 5	17 (0.39)	14 (0.43)
		**DVT**	< 5	< 5	< 5	< 5	9 (0.21)	12 (0.37)
		**PE**	10 (0.05)	< 5	< 5	< 5	9 (0.21)	< 5
		**ATE**	10 (0.05)	10 (0.10)	< 5	< 5	61 (1.41)	40 (1.24)
		**IS**	< 5	< 5	< 5	< 5	33 (0.76)	19 (0.59)
		**TIA**	< 5	< 5	< 5	< 5	18 (0.42)	12 (0.37)
		**MI**	7 (0.04)	6 (0.06)	< 5	< 5	12 (0.28)	13 (0.40)
		**HF**	17 (0.09)	22 (0.23)	< 5	< 5	70 (1.61)	25 (0.77)
		**HS**	< 5	< 5	< 5	< 5	< 5	< 5
		**MP**	< 5	< 5	< 5	< 5	< 5	< 5
**Cohort 3**			**N = 1,510,493**	**N = 1,473,602**	**N = 418,184**	**N = 423,876**	**N = 873,400**	**N = 84,204**
	**0 to 30 days**	**VTE**	244 (1.62)	139 (0.94)	28 (0.67)	16 (0.38)	332 (3.80)	27 (3.21)
		**DVT**	44 (0.29)	41 (0.28)	6 (0.14)	< 5	95 (1.09)	9 (1.07)
		**PE**	209 (1.38)	101 (0.69)	23 (0.55)	12 (0.28)	266 (3.05)	22 (2.61)
		**ATE**	28 (0.19)	47 (0.32)	< 5	12 (0.28)	216 (2.47)	32 (3.80)
		**IS**	< 5	5 (0.03)	< 5	< 5	103 (1.18)	13 (1.54)
		**TIA**	5 (0.03)	16 (0.11)	< 5	< 5	22 (0.25)	5 (0.59)
		**MI**	19 (0.13)	26 (0.18)	< 5	9 (0.21)	97 (1.11)	16 (1.90)
		**HF**	30 (0.20)	28 (0.19)	< 5	< 5	372 (4.26)	51 (6.06)
		**HS**	< 5	< 5	< 5	< 5	20 (0.23)	< 5
		**MP**	9 (0.06)	6 (0.04)	< 5	< 5	20 (0.23)	6 (0.71)
	**31 to 90 days**	**VTE**	43 (0.28)	44 (0.30)	< 5	6 (0.14)	81 (0.93)	9 (1.07)
		**DVT**	20 (0.13)	26 (0.18)	< 5	< 5	54 (0.62)	< 5
		**PE**	26 (0.17)	18 (0.12)	< 5	< 5	36 (0.41)	6 (0.71)
		**ATE**	11 (0.07)	31 (0.21)	< 5	7 (0.17)	108 (1.24)	25 (2.97)
		**IS**	< 5	< 5	< 5	< 5	52 (0.60)	11 (1.31)
		**TIA**	< 5	13 (0.09)	< 5	< 5	27 (0.31)	< 5

Cohort	Time window	Outcome	AURUM		GOLD		SIDIAP	
			Unvaccinated	Vaccinated	Unvaccinated	Vaccinated	Unvaccinated	Vaccinated
		**MI**	9 (0.06)	16 (0.11)	< 5	< 5	37 (0.42)	10 (1.19)
		**HF**	14 (0.09)	23 (0.16)	< 5	< 5	135 (1.55)	25 (2.97)
		**HS**	< 5	< 5	< 5	< 5	7 (0.08)	< 5
		**MP**	8 (0.05)	7 (0.05)	< 5	< 5	9 (0.10)	< 5
	**91 to 180 days**	**VTE**	23 (0.15)	25 (0.17)	< 5	< 5	58 (0.66)	13 (1.54)
		**DVT**	10 (0.07)	15 (0.10)	< 5	< 5	33 (0.38)	7 (0.83)
		**PE**	14 (0.09)	13 (0.09)	< 5	< 5	29 (0.33)	7 (0.83)
		**ATE**	< 5	26 (0.18)	< 5	< 5	104 (1.19)	28 (3.33)
		**IS**	< 5	< 5	< 5	< 5	50 (0.57)	12 (1.43)
		**TIA**	< 5	8 (0.05)	< 5	< 5	20 (0.23)	10 (1.19)
		**MI**	< 5	17 (0.12)	< 5	< 5	36 (0.41)	8 (0.95)
		**HF**	10 (0.07)	10 (0.07)	< 5	< 5	111 (1.27)	12 (1.43)
		**HS**	< 5	< 5	< 5	< 5	9 (0.10)	< 5
		**MP**	< 5	< 5	< 5	< 5	8 (0.09)	< 5
	**181 to 365 days**	**VTE**	< 5	9 (0.06)	< 5	< 5	31 (0.35)	< 5
		**DVT**	< 5	< 5	< 5	< 5	18 (0.21)	< 5
		**PE**	< 5	6 (0.04)	< 5	< 5	15 (0.17)	< 5
		**ATE**	< 5	< 5	< 5	< 5	48 (0.55)	15 (1.78)
		**IS**	< 5	< 5	< 5	< 5	21 (0.24)	6 (0.71)
		**TIA**	< 5	< 5	< 5	< 5	11 (0.13)	< 5
		**MI**	< 5	< 5	< 5	< 5	17 (0.19)	8 (0.95)
		**HF**	< 5	< 5	< 5	< 5	55 (0.63)	6 (0.71)
		**HS**	< 5	< 5	< 5	< 5	< 5	< 5
		**MP**	< 5	< 5	< 5	< 5	< 5	< 5
**Cohort 4**			**N = 2,066,318**	**N = 542,670**	**N = 485,154**	**N = 147,744**		
	**0 to 30 days**	**VTE**	346 (1.67)	27 (0.50)	38 (0.78)	8 (0.54)		
		**DVT**	56 (0.27)	7 (0.13)	6 (0.12)	< 5		
		**PE**	302 (1.46)	21 (0.39)	33 (0.68)	6 (0.41)		
		**ATE**	26 (0.13)	5 (0.09)	< 5	< 5		
		**IS**	< 5	< 5	< 5	< 5		
		**TIA**	5 (0.02)	< 5	< 5	< 5		
		**MI**	17 (0.08)	< 5	< 5	< 5		
		**HF**	28 (0.14)	< 5	< 5	< 5		
		**HS**	< 5	< 5	< 5	< 5		

Cohort	Time window	Outcome	AURUM		GOLD		SIDIAP	
			Unvaccinated	Vaccinated	Unvaccinated	Vaccinated	Unvaccinated	Vaccinated
		**MP**	14 (0.07)	< 5	< 5	< 5		
	**31 to 90 days**	**VTE**	57 (0.28)	14 (0.26)	< 5	< 5		
		**DVT**	22 (0.11)	8 (0.15)	< 5	< 5		
		**PE**	38 (0.18)	6 (0.11)	< 5	< 5		
		**ATE**	13 (0.06)	< 5	< 5	< 5		
		**IS**	< 5	< 5	< 5	< 5		
		**TIA**	< 5	< 5	< 5	< 5		
		**MI**	9 (0.04)	< 5	< 5	< 5		
		**HF**	14 (0.07)	< 5	< 5	< 5		
		**HS**	< 5	< 5	< 5	< 5		
		**MP**	7 (0.03)	< 5	< 5	< 5		
	**91 to 180 days**	**VTE**	28 (0.14)	< 5	< 5	< 5		
		**DVT**	9 (0.04)	< 5	< 5	< 5		
		**PE**	20 (0.10)	< 5	< 5	< 5		
		**ATE**	< 5	< 5	< 5	< 5		
		**IS**	< 5	< 5	< 5	< 5		
		**TIA**	< 5	< 5	< 5	< 5		
		**MI**	< 5	< 5	< 5	< 5		
		**HF**	10 (0.05)	< 5	< 5	< 5		
		**HS**	< 5	< 5	< 5	< 5		
		**MP**	< 5	< 5	< 5	< 5		
	**181 to 365 days**	**VTE**	< 5	< 5	< 5	< 5		
		**DVT**	< 5	< 5	< 5	< 5		
		**PE**	< 5	< 5	< 5	< 5		
		**ATE**	< 5	< 5	< 5	< 5		
		**IS**	< 5	< 5	< 5	< 5		
		**TIA**	< 5	< 5	< 5	< 5		
		**MI**	< 5	< 5	< 5	< 5		
		**HF**	< 5	< 5	< 5	< 5		
		**HS**	< 5	< 5	< 5	< 5		
		**MP**	< 5	< 5	< 5	< 5		

VTE = Venous thromboembolism (DVT + PE), DVT = Deep vein thrombosis, PE = Pulmonary embolism, ATE = Arterial thrombosis/thromboembolism (IS + TIA + MI), IS = Ischemic stroke, TIA = Transient ischemic attack, MI = Myocardial infarction, HF = Heart failure, HS = Hemorrhagic stroke, MP = Myocarditis or Pericarditis

Mercadé-Besora N, *et al. Heart* 2024; 110:635–643. doi: 10.1136/heartjnl-2023-323483

Table S31: Number of records (and risk per 10,000 individuals) for post COVID-19 cardiac and thromboembolic complications, across cohorts and databases, stratified by exposure status (BNT162b2 vaccine).

Cohort	Time window	Outcome	AURUM		CORIVA		GOLD		SIDIAP	
			Unvaccinated	Vaccinated	Unvaccinated	Vaccinated	Unvaccinated	Vaccinated	Unvaccinated	Vaccinated
**Cohort 1**			**N = 348,052**	**N = 332,790**	**N = 24,073**	**N = 19,686**	**N = 169,459**	**N = 32,755**	**N = 223,960**	**N = 88,896**
	**0 to 30 days**	**VTE**	106 (3.05)	76 (2.28)	76 (31.57)	40 (20.32)	8 (0.47)	< 5	75 (3.35)	94 (10.57)
		**DVT**	21 (0.60)	15 (0.45)	14 (5.82)	13 (6.60)	< 5	< 5	20 (0.89)	29 (3.26)
		**PE**	90 (2.59)	64 (1.92)	64 (26.59)	28 (14.22)	6 (0.35)	< 5	60 (2.68)	75 (8.44)
		**ATE**	27 (0.78)	45 (1.35)	114 (47.36)	61 (30.99)	6 (0.35)	< 5	79 (3.53)	206 (23.17)
		**IS**	9 (0.26)	5 (0.15)	68 (28.25)	26 (13.21)	< 5	< 5	46 (2.05)	116 (13.05)
		**TIA**	5 (0.14)	11 (0.33)	22 (9.14)	15 (7.62)	< 5	< 5	7 (0.31)	41 (4.61)
		**MI**	14 (0.40)	29 (0.87)	34 (14.12)	27 (13.72)	< 5	< 5	28 (1.25)	61 (6.86)
		**HF**	59 (1.70)	126 (3.79)	393 (163.25)	231 (117.34)	9 (0.53)	< 5	299 (13.35)	634 (71.32)
		**HS**	< 5	< 5	< 5	< 5	< 5	< 5	7 (0.31)	14 (1.57)
		**MP**	< 5	< 5	< 5	< 5	< 5	< 5	< 5	< 5
	**31 to 90 days**	**VTE**	18 (0.52)	26 (0.78)	40 (16.62)	24 (12.19)	< 5	< 5	16 (0.71)	44 (4.95)
		**DVT**	9 (0.26)	7 (0.21)	19 (7.89)	14 (7.11)	< 5	< 5	10 (0.45)	29 (3.26)
		**PE**	9 (0.26)	19 (0.57)	24 (9.97)	10 (5.08)	< 5	< 5	6 (0.27)	19 (2.14)
		**ATE**	9 (0.26)	23 (0.69)	31 (12.88)	35 (17.78)	< 5	< 5	41 (1.83)	128 (14.40)
		**IS**	< 5	< 5	18 (7.48)	14 (7.11)	< 5	< 5	20 (0.89)	71 (7.99)
		**TIA**	5 (0.14)	10 (0.30)	7 (2.91)	14 (7.11)	< 5	< 5	13 (0.58)	32 (3.60)
		**MI**	5 (0.14)	11 (0.33)	11 (4.57)	10 (5.08)	< 5	< 5	11 (0.49)	29 (3.26)
		**HF**	32 (0.92)	71 (2.13)	151 (62.73)	141 (71.62)	< 5	< 5	87 (3.88)	296 (33.30)
		**HS**	< 5	< 5	< 5	< 5	< 5	< 5	< 5	13 (1.46)
		**MP**	< 5	< 5	< 5	< 5	< 5	< 5	< 5	< 5
	**91 to 180 days**	**VTE**	10 (0.29)	6 (0.18)	19 (7.89)	27 (13.72)	< 5	< 5	21 (0.94)	40 (4.50)
		**DVT**	< 5	< 5	11 (4.57)	14 (7.11)	< 5	< 5	9 (0.40)	23 (2.59)
		**PE**	6 (0.17)	< 5	9 (3.74)	14 (7.11)	< 5	< 5	12 (0.54)	20 (2.25)
		**ATE**	15 (0.43)	18 (0.54)	31 (12.88)	40 (20.32)	< 5	< 5	31 (1.38)	110 (12.37)
		**IS**	< 5	6 (0.18)	16 (6.65)	17 (8.64)	< 5	< 5	17 (0.76)	59 (6.64)
		**TIA**	8 (0.23)	6 (0.18)	8 (3.32)	11 (5.59)	< 5	< 5	8 (0.36)	34 (3.82)
		**MI**	5 (0.14)	6 (0.18)	8 (3.32)	16 (8.13)	< 5	< 5	7 (0.31)	26 (2.92)
		**HF**	43 (1.24)	57 (1.71)	166 (68.96)	169 (85.85)	< 5	< 5	87 (3.88)	249 (28.01)
		**HS**	< 5	< 5	< 5	< 5	< 5	< 5	< 5	< 5
		**MP**	< 5	< 5	< 5	< 5	< 5	< 5	< 5	< 5

Cohort	Time window	Outcome	AURUM		CORIVA		GOLD		SIDIAP	
			Unvaccinated	Vaccinated	Unvaccinated	Vaccinated	Unvaccinated	Vaccinated	Unvaccinated	Vaccinated
	181 to 365 days	VTE	14 (0.40)	10 (0.30)	43 (17.86)	31 (15.75)	< 5	< 5	10 (0.45)	12 (1.35)
		DVT	< 5	6 (0.18)	16 (6.65)	18 (9.14)	< 5	< 5	5 (0.22)	8 (0.90)
		PE	11 (0.32)	< 5	31 (12.88)	14 (7.11)	< 5	< 5	5 (0.22)	< 5
		ATE	15 (0.43)	14 (0.42)	57 (23.68)	62 (31.49)	< 5	< 5	41 (1.83)	52 (5.85)
		IS	< 5	< 5	32 (13.29)	30 (15.24)	< 5	< 5	20 (0.89)	33 (3.71)
		TIA	10 (0.29)	< 5	16 (6.65)	24 (12.19)	< 5	< 5	11 (0.49)	13 (1.46)
		MI	< 5	7 (0.21)	14 (5.82)	16 (8.13)	< 5	< 5	10 (0.45)	8 (0.90)
		HF	52 (1.49)	38 (1.14)	278 (115.48)	244 (123.95)	< 5	< 5	82 (3.66)	149 (16.76)
		HS	< 5	< 5	< 5	< 5	< 5	< 5	< 5	9 (1.01)
		MP	< 5	< 5	< 5	< 5	< 5	< 5	< 5	< 5
Cohort 2			N = 1,976,163	N = 594,262			N = 584,309	N = 180,670	N = 433,111	N = 445,581
	0 to 30 days	VTE	240 (1.21)	62 (1.04)			29 (0.50)	5 (0.28)	259 (5.98)	265 (5.95)
		DVT	39 (0.20)	18 (0.30)			7 (0.12)	< 5	63 (1.45)	96 (2.15)
		PE	205 (1.04)	45 (0.76)			23 (0.39)	< 5	215 (4.96)	189 (4.24)
		ATE	39 (0.20)	29 (0.49)			< 5	< 5	172 (3.97)	489 (10.97)
		IS	7 (0.04)	< 5			< 5	< 5	94 (2.17)	267 (5.99)
		TIA	6 (0.03)	11 (0.19)			< 5	< 5	17 (0.39)	90 (2.02)
		MI	26 (0.13)	16 (0.27)			< 5	< 5	65 (1.50)	163 (3.66)
		HF	50 (0.25)	43 (0.72)			6 (0.10)	< 5	380 (8.77)	1,137 (25.52)
		HS	6 (0.03)	< 5			< 5	< 5	18 (0.42)	61 (1.37)
		MP	11 (0.06)	< 5			< 5	< 5	14 (0.32)	22 (0.49)
	31 to 90 days	VTE	49 (0.25)	21 (0.35)			< 5	< 5	60 (1.39)	128 (2.87)
		DVT	26 (0.13)	7 (0.12)			< 5	< 5	33 (0.76)	81 (1.82)
		PE	26 (0.13)	14 (0.24)			< 5	< 5	34 (0.79)	56 (1.26)
		ATE	17 (0.09)	36 (0.61)			< 5	< 5	86 (1.99)	313 (7.02)
		IS	< 5	5 (0.08)			< 5	< 5	43 (0.99)	167 (3.75)
		TIA	< 5	9 (0.15)			< 5	< 5	24 (0.55)	86 (1.93)
		MI	11 (0.06)	22 (0.37)			< 5	< 5	26 (0.60)	80 (1.80)
		HF	27 (0.14)	35 (0.59)			< 5	< 5	137 (3.16)	554 (12.43)
		HS	< 5	< 5			< 5	< 5	7 (0.16)	19 (0.43)
		MP	6 (0.03)	< 5			< 5	< 5	< 5	< 5
	91 to 180 days	VTE	28 (0.14)	10 (0.17)			5 (0.09)	< 5	59 (1.36)	96 (2.15)
		DVT	12 (0.06)	6 (0.10)			< 5	< 5	32 (0.74)	59 (1.32)
		PE	16 (0.08)	5 (0.08)			< 5	< 5	30 (0.69)	45 (1.01)

Cohort	Time window	Outcome	AURUM		CORIVA		GOLD		SIDIAP	
			Unvaccinated	Vaccinated	Unvaccinated	Vaccinated	Unvaccinated	Vaccinated	Unvaccinated	Vaccinated
		ATE	14 (0.07)	15 (0.25)			< 5	< 5	90 (2.08)	287 (6.44)
		IS	< 5	< 5			< 5	< 5	49 (1.13)	168 (3.77)
		TIA	7 (0.04)	5 (0.08)			< 5	< 5	21 (0.48)	76 (1.71)
		MI	8 (0.04)	10 (0.17)			< 5	< 5	23 (0.53)	63 (1.41)
		HF	19 (0.10)	25 (0.42)			< 5	< 5	110 (2.54)	472 (10.59)
		HS	< 5	< 5			< 5	< 5	7 (0.16)	34 (0.76)
		MP	< 5	< 5			< 5	< 5	7 (0.16)	< 5
	181 to 365 days	VTE	10 (0.05)	8 (0.13)			< 5	< 5	16 (0.37)	40 (0.90)
		DVT	< 5	< 5			< 5	< 5	10 (0.23)	26 (0.58)
		PE	8 (0.04)	7 (0.12)			< 5	< 5	8 (0.18)	16 (0.36)
		ATE	12 (0.06)	7 (0.12)			< 5	< 5	59 (1.36)	123 (2.76)
		IS	< 5	< 5			< 5	< 5	29 (0.67)	58 (1.30)
		TIA	< 5	< 5			< 5	< 5	18 (0.42)	39 (0.88)
		MI	9 (0.05)	< 5			< 5	< 5	14 (0.32)	32 (0.72)
		HF	23 (0.12)	12 (0.20)			< 5	< 5	73 (1.69)	212 (4.76)
		HS	< 5	< 5			< 5	< 5	6 (0.14)	12 (0.27)
		MP	< 5	< 5			< 5	< 5	< 5	< 5
Cohort 3			N = 1,510,323	N = 54,102			N = 416,549	N = 36,748	N = 869,109	N = 706,435
	0 to 30 days	VTE	244 (1.62)	< 5			28 (0.67)	< 5	321 (3.69)	114 (1.61)
		DVT	44 (0.29)	< 5			6 (0.14)	< 5	89 (1.02)	51 (0.72)
		PE	209 (1.38)	< 5			23 (0.55)	< 5	259 (2.98)	71 (1.01)
		ATE	29 (0.19)	< 5			< 5	< 5	211 (2.43)	197 (2.79)
		IS	< 5	< 5			< 5	< 5	102 (1.17)	95 (1.34)
		TIA	5 (0.03)	< 5			< 5	< 5	20 (0.23)	42 (0.59)
		MI	20 (0.13)	< 5			< 5	< 5	95 (1.09)	78 (1.10)
		HF	30 (0.20)	10 (1.85)			< 5	< 5	366 (4.21)	163 (2.31)
		HS	< 5	< 5			< 5	< 5	20 (0.23)	26 (0.37)
		MP	9 (0.06)	< 5			< 5	< 5	19 (0.22)	13 (0.18)
	31 to 90 days	VTE	44 (0.29)	< 5			< 5	< 5	84 (0.97)	65 (0.92)
		DVT	20 (0.13)	< 5			< 5	< 5	56 (0.64)	47 (0.67)
		PE	27 (0.18)	< 5			< 5	< 5	38 (0.44)	23 (0.33)
		ATE	11 (0.07)	< 5			< 5	< 5	107 (1.23)	144 (2.04)
		IS	< 5	< 5			< 5	< 5	53 (0.61)	55 (0.78)
		TIA	< 5	< 5			< 5	< 5	27 (0.31)	28 (0.40)

Cohort	Time window	Outcome	AURUM		CORIVA		GOLD		SIDIAP	
			Unvaccinated	Vaccinated	Unvaccinated	Vaccinated	Unvaccinated	Vaccinated	Unvaccinated	Vaccinated
		MI	9 (0.06)	< 5			< 5	< 5	35 (0.40)	69 (0.98)
		HF	15 (0.10)	< 5			< 5	< 5	139 (1.60)	111 (1.57)
		HS	< 5	< 5			< 5	< 5	7 (0.08)	14 (0.20)
		MP	8 (0.05)	< 5			< 5	< 5	8 (0.09)	9 (0.13)
	91 to 180 days	VTE	24 (0.16)	< 5			< 5	< 5	63 (0.72)	71 (1.01)
		DVT	11 (0.07)	< 5			< 5	< 5	35 (0.40)	45 (0.64)
		PE	14 (0.09)	< 5			< 5	< 5	32 (0.37)	27 (0.38)
		ATE	< 5	< 5			< 5	< 5	112 (1.29)	141 (2.00)
		IS	< 5	< 5			< 5	< 5	57 (0.66)	65 (0.92)
		TIA	< 5	< 5			< 5	< 5	22 (0.25)	29 (0.41)
		MI	< 5	< 5			< 5	< 5	36 (0.41)	53 (0.75)
		HF	10 (0.07)	< 5			< 5	< 5	121 (1.39)	100 (1.42)
		HS	< 5	< 5			< 5	< 5	9 (0.10)	10 (0.14)
		MP	< 5	< 5			< 5	< 5	7 (0.08)	9 (0.13)
	181 to 365 days	VTE	< 5	< 5			< 5	< 5	36 (0.41)	17 (0.24)
		DVT	< 5	< 5			< 5	< 5	22 (0.25)	14 (0.20)
		PE	< 5	< 5			< 5	< 5	16 (0.18)	< 5
		ATE	< 5	< 5			< 5	< 5	48 (0.55)	45 (0.64)
		IS	< 5	< 5			< 5	< 5	23 (0.26)	17 (0.24)
		TIA	< 5	< 5			< 5	< 5	9 (0.10)	7 (0.10)
		MI	< 5	< 5			< 5	< 5	17 (0.20)	24 (0.34)
		HF	5 (0.03)	< 5			< 5	< 5	61 (0.70)	31 (0.44)
		HS	< 5	< 5			< 5	< 5	< 5	< 5
		MP	< 5	< 5			< 5	< 5	< 5	< 5
**Cohort 4**			**N = 2,014,161**	**N = 1,335,671**	**N = 147,553**	**N = 15,683**	**N = 465,326**	**N = 365,096**	**N = 1,068,043**	**N = 580,329**
	0 to 30 days	VTE	328 (1.63)	20 (0.15)	116 (7.86)	< 5	36 (0.77)	< 5	350 (3.28)	66 (1.14)
		DVT	52 (0.26)	9 (0.07)	22 (1.49)	< 5	6 (0.13)	< 5	107 (1.00)	35 (0.60)
		PE	287 (1.42)	11 (0.08)	97 (6.57)	< 5	31 (0.67)	< 5	271 (2.54)	35 (0.60)
		ATE	25 (0.12)	< 5	116 (7.86)	< 5	< 5	< 5	233 (2.18)	55 (0.95)
		IS	< 5	< 5	69 (4.68)	< 5	< 5	< 5	114 (1.07)	29 (0.50)
		TIA	6 (0.03)	< 5	18 (1.22)	< 5	< 5	< 5	28 (0.26)	7 (0.12)
		MI	15 (0.07)	< 5	38 (2.58)	< 5	< 5	< 5	97 (0.91)	20 (0.34)
		HF	26 (0.13)	< 5	362 (24.53)	8 (5.10)	< 5	< 5	369 (3.45)	44 (0.76)
		HS	< 5	< 5	5 (0.34)	< 5	< 5	< 5	24 (0.22)	7 (0.12)

Cohort	Time window	Outcome	AURUM		CORIVA		GOLD		SIDIAP	
			Unvaccinated	Vaccinated	Unvaccinated	Vaccinated	Unvaccinated	Vaccinated	Unvaccinated	Vaccinated
		MP	13 (0.06)	< 5	7 (0.47)	< 5	< 5	< 5	26 (0.24)	11 (0.19)
	31 to 90 days	VTE	56 (0.28)	7 (0.05)	54 (3.66)	< 5	< 5	< 5	93 (0.87)	33 (0.57)
		DVT	20 (0.10)	< 5	32 (2.17)	< 5	< 5	< 5	62 (0.58)	23 (0.40)
		PE	39 (0.19)	< 5	26 (1.76)	< 5	< 5	< 5	41 (0.38)	11 (0.19)
		ATE	13 (0.06)	< 5	46 (3.12)	< 5	< 5	< 5	115 (1.08)	51 (0.88)
		IS	< 5	< 5	24 (1.63)	< 5	< 5	< 5	51 (0.48)	21 (0.36)
		TIA	< 5	< 5	6 (0.41)	< 5	< 5	< 5	31 (0.29)	12 (0.21)
		MI	9 (0.04)	< 5	19 (1.29)	< 5	< 5	< 5	41 (0.38)	21 (0.36)
		HF	14 (0.07)	6 (0.04)	174 (11.79)	5 (3.19)	< 5	< 5	143 (1.34)	28 (0.48)
		HS	< 5	< 5	< 5	< 5	< 5	< 5	10 (0.09)	5 (0.09)
		MP	7 (0.03)	< 5	5 (0.34)	< 5	< 5	< 5	12 (0.11)	8 (0.14)
	91 to 180 days	VTE	24 (0.12)	9 (0.07)	49 (3.32)	< 5	< 5	< 5	77 (0.72)	45 (0.78)
		DVT	11 (0.05)	7 (0.05)	31 (2.10)	< 5	< 5	< 5	42 (0.39)	28 (0.48)
		PE	14 (0.07)	< 5	19 (1.29)	< 5	< 5	< 5	38 (0.36)	20 (0.34)
		ATE	< 5	< 5	41 (2.78)	< 5	< 5	< 5	134 (1.25)	71 (1.22)
		IS	< 5	< 5	19 (1.29)	< 5	< 5	< 5	66 (0.62)	37 (0.64)
		TIA	< 5	< 5	12 (0.81)	< 5	< 5	< 5	31 (0.29)	14 (0.24)
		MI	< 5	< 5	14 (0.95)	< 5	< 5	< 5	40 (0.37)	20 (0.34)
		HF	9 (0.04)	< 5	208 (14.10)	< 5	< 5	< 5	145 (1.36)	35 (0.60)
		HS	< 5	< 5	< 5	< 5	< 5	< 5	15 (0.14)	< 5
		MP	< 5	< 5	5 (0.34)	< 5	< 5	< 5	13 (0.12)	< 5
	181 to 365 days	VTE	< 5	< 5	77 (5.22)	5 (3.19)	< 5	< 5	52 (0.49)	10 (0.17)
		DVT	< 5	< 5	46 (3.12)	< 5	< 5	< 5	36 (0.34)	8 (0.14)
		PE	< 5	< 5	35 (2.37)	< 5	< 5	< 5	18 (0.17)	< 5
		ATE	< 5	< 5	72 (4.88)	< 5	< 5	< 5	52 (0.49)	21 (0.36)
		IS	< 5	< 5	33 (2.24)	< 5	< 5	< 5	28 (0.26)	11 (0.19)
		TIA	< 5	< 5	18 (1.22)	< 5	< 5	< 5	10 (0.09)	< 5
		MI	< 5	< 5	27 (1.83)	< 5	< 5	< 5	15 (0.14)	9 (0.16)
		HF	< 5	< 5	298 (20.20)	5 (3.19)	< 5	< 5	58 (0.54)	8 (0.14)
		HS	< 5	< 5	8 (0.54)	< 5	< 5	< 5	5 (0.05)	< 5
		MP	< 5	< 5	5 (0.34)	< 5	< 5	< 5	6 (0.06)	< 5

VTE = Venous thromboembolism (DVT + PE), DVT = Deep vein thrombosis, PE = Pulmonary embolism, ATE = Arterial thrombosis/thromboembolism (IS + TIA + MI), IS = Ischemic stroke, TIA = Transient ischemic attack, MI = Myocardial infarction, HF = Heart failure, HS = Hemorrhagic stroke, MP = Myocarditis or Pericarditis

Table S32: Number of records (and risk per 10,000 individuals) for post COVID-19 cardiac and thromboembolic complications, across cohorts and databases, stratified by exposure status (BNT162b2 vaccine). Follow-up ends at first vaccine dose after index date.

Cohort	Time window	Outcome	AURUM		CORIVA		GOLD		SIDIAP	
			Unvaccinated	Vaccinated	Unvaccinated	Vaccinated	Unvaccinated	Vaccinated	Unvaccinated	Vaccinated
**Cohort 1**			**N = 348,052**	**N = 332,790**	**N = 24,073**	**N = 19,686**	**N = 169,459**	**N = 32,755**	**N = 223,960**	**N = 88,896**
	**0 to 30 days**	**VTE**	106 (3.05)	31 (0.93)	76 (31.57)	< 5	8 (0.47)	< 5	75 (3.35)	< 5
		**DVT**	21 (0.60)	8 (0.24)	14 (5.82)	< 5	< 5	< 5	20 (0.89)	< 5
		**PE**	90 (2.59)	24 (0.72)	64 (26.59)	< 5	6 (0.35)	< 5	60 (2.68)	< 5
		**ATE**	27 (0.78)	18 (0.54)	114 (47.36)	< 5	6 (0.35)	< 5	79 (3.53)	5 (0.56)
		**IS**	9 (0.26)	< 5	68 (28.25)	< 5	< 5	< 5	46 (2.05)	< 5
		**TIA**	5 (0.14)	< 5	22 (9.14)	< 5	< 5	< 5	7 (0.31)	< 5
		**MI**	14 (0.40)	14 (0.42)	34 (14.12)	< 5	< 5	< 5	28 (1.25)	< 5
		**HF**	59 (1.70)	45 (1.35)	393 (163.25)	20 (10.16)	9 (0.53)	< 5	299 (13.35)	23 (2.59)
		**HS**	< 5	< 5	< 5	< 5	< 5	< 5	7 (0.31)	< 5
		**MP**	< 5	< 5	< 5	< 5	< 5	< 5	< 5	< 5
	**31 to 90 days**	**VTE**	18 (0.52)	15 (0.45)	40 (16.62)	< 5	< 5	< 5	16 (0.71)	< 5
		**DVT**	9 (0.26)	< 5	19 (7.89)	< 5	< 5	< 5	10 (0.45)	< 5
		**PE**	9 (0.26)	11 (0.33)	24 (9.97)	< 5	< 5	< 5	6 (0.27)	< 5
		**ATE**	9 (0.26)	8 (0.24)	31 (12.88)	< 5	< 5	< 5	41 (1.83)	5 (0.56)
		**IS**	< 5	< 5	18 (7.48)	< 5	< 5	< 5	20 (0.89)	< 5
		**TIA**	5 (0.14)	< 5	7 (2.91)	< 5	< 5	< 5	13 (0.58)	< 5
		**MI**	5 (0.14)	< 5	11 (4.57)	< 5	< 5	< 5	11 (0.49)	< 5
		**HF**	32 (0.92)	36 (1.08)	151 (62.73)	11 (5.59)	< 5	< 5	87 (3.88)	8 (0.90)
		**HS**	< 5	< 5	< 5	< 5	< 5	< 5	< 5	< 5
		**MP**	< 5	< 5	< 5	< 5	< 5	< 5	< 5	< 5
	**91 to 180 days**	**VTE**	10 (0.29)	< 5	19 (7.89)	< 5	< 5	< 5	21 (0.94)	< 5
		**DVT**	< 5	< 5	11 (4.57)	< 5	< 5	< 5	9 (0.40)	< 5
		**PE**	6 (0.17)	< 5	9 (3.74)	< 5	< 5	< 5	12 (0.54)	< 5
		**ATE**	15 (0.43)	10 (0.30)	31 (12.88)	< 5	< 5	< 5	31 (1.38)	< 5
		**IS**	< 5	< 5	16 (6.65)	< 5	< 5	< 5	17 (0.76)	< 5
		**TIA**	8 (0.23)	< 5	8 (3.32)	< 5	< 5	< 5	8 (0.36)	< 5
		**MI**	5 (0.14)	< 5	8 (3.32)	< 5	< 5	< 5	7 (0.31)	< 5
		**HF**	43 (1.24)	32 (0.96)	166 (68.96)	14 (7.11)	< 5	< 5	87 (3.88)	6 (0.67)
		**HS**	< 5	< 5	< 5	< 5	< 5	< 5	< 5	< 5
		**MP**	< 5	< 5	< 5	< 5	< 5	< 5	< 5	< 5

Mercadé-Besora N, *et al. Heart* 2024; 110:635–643. doi: 10.1136/heartjnl-2023-323483

Cohort	Time window	Outcome	AURUM		CORIVA		GOLD		SIDIAP	
			Unvaccinated	Vaccinated	Unvaccinated	Vaccinated	Unvaccinated	Vaccinated	Unvaccinated	Vaccinated
	**181 to 365 days**	**VTE**	14 (0.40)	7 (0.21)	43 (17.86)	6 (3.05)	< 5	< 5	10 (0.45)	< 5
		**DVT**	< 5	< 5	16 (6.65)	< 5	< 5	< 5	5 (0.22)	< 5
		**PE**	11 (0.32)	< 5	31 (12.88)	< 5	< 5	< 5	5 (0.22)	< 5
		**ATE**	15 (0.43)	11 (0.33)	57 (23.68)	10 (5.08)	< 5	< 5	41 (1.83)	8 (0.90)
		**IS**	< 5	< 5	32 (13.29)	9 (4.57)	< 5	< 5	20 (0.89)	5 (0.56)
		**TIA**	10 (0.29)	< 5	16 (6.65)	< 5	< 5	< 5	11 (0.49)	< 5
		**MI**	< 5	6 (0.18)	14 (5.82)	< 5	< 5	< 5	10 (0.45)	< 5
		**HF**	52 (1.49)	35 (1.05)	278 (115.48)	37 (18.80)	< 5	< 5	82 (3.66)	20 (2.25)
		**HS**	< 5	< 5	< 5	< 5	< 5	< 5	< 5	< 5
		**MP**	< 5	< 5	< 5	< 5	< 5	< 5	< 5	< 5
**Cohort 2**			**N = 1,976,163**	**N = 594,262**			**N = 584,309**	**N = 180,670**	**N = 433,111**	**N = 445,581**
	**0 to 30 days**	**VTE**	240 (1.21)	14 (0.24)			29 (0.50)	< 5	259 (5.98)	14 (0.31)
		**DVT**	39 (0.20)	6 (0.10)			7 (0.12)	< 5	63 (1.45)	< 5
		**PE**	205 (1.04)	8 (0.13)			23 (0.39)	< 5	215 (4.96)	12 (0.27)
		**ATE**	39 (0.20)	14 (0.24)			< 5	< 5	172 (3.97)	16 (0.36)
		**IS**	7 (0.04)	< 5			< 5	< 5	94 (2.17)	8 (0.18)
		**TIA**	6 (0.03)	< 5			< 5	< 5	17 (0.39)	< 5
		**MI**	26 (0.13)	8 (0.13)			< 5	< 5	65 (1.50)	5 (0.11)
		**HF**	50 (0.25)	15 (0.25)			6 (0.10)	< 5	380 (8.77)	56 (1.26)
		**HS**	6 (0.03)	< 5			< 5	< 5	18 (0.42)	< 5
		**MP**	11 (0.06)	< 5			< 5	< 5	14 (0.32)	< 5
	**31 to 90 days**	**VTE**	49 (0.25)	5 (0.08)			< 5	< 5	60 (1.39)	6 (0.13)
		**DVT**	26 (0.13)	< 5			< 5	< 5	33 (0.76)	< 5
		**PE**	26 (0.13)	< 5			< 5	< 5	34 (0.79)	< 5
		**ATE**	17 (0.09)	9 (0.15)			< 5	< 5	86 (1.99)	7 (0.16)
		**IS**	< 5	< 5			< 5	< 5	43 (0.99)	< 5
		**TIA**	< 5	< 5			< 5	< 5	24 (0.55)	< 5
		**MI**	11 (0.06)	< 5			< 5	< 5	26 (0.60)	< 5
		**HF**	27 (0.14)	17 (0.29)			< 5	< 5	137 (3.16)	19 (0.43)
		**HS**	< 5	< 5			< 5	< 5	7 (0.16)	< 5
		**MP**	6 (0.03)	< 5			< 5	< 5	< 5	< 5
	**91 to 180 days**	**VTE**	28 (0.14)	< 5			5 (0.09)	< 5	59 (1.36)	< 5
		**DVT**	12 (0.06)	< 5			< 5	< 5	32 (0.74)	< 5
		**PE**	16 (0.08)	< 5			< 5	< 5	30 (0.69)	< 5

Cohort	Time window	Outcome	AURUM		CORIVA		GOLD		SIDIAP	
			Unvaccinated	Vaccinated	Unvaccinated	Vaccinated	Unvaccinated	Vaccinated	Unvaccinated	Vaccinated
		**ATE**	14 (0.07)	5 (0.08)			< 5	< 5	90 (2.08)	10 (0.22)
		**IS**	< 5	< 5			< 5	< 5	49 (1.13)	5 (0.11)
		**TIA**	7 (0.04)	< 5			< 5	< 5	21 (0.48)	< 5
		**MI**	8 (0.04)	< 5			< 5	< 5	23 (0.53)	< 5
		**HF**	19 (0.10)	9 (0.15)			< 5	< 5	110 (2.54)	25 (0.56)
		**HS**	< 5	< 5			< 5	< 5	7 (0.16)	< 5
		**MP**	< 5	< 5			< 5	< 5	7 (0.16)	< 5
	**181 to 365 days**	**VTE**	10 (0.05)	7 (0.12)			< 5	< 5	16 (0.37)	10 (0.22)
		**DVT**	< 5	< 5			< 5	< 5	10 (0.23)	6 (0.13)
		**PE**	8 (0.04)	5 (0.08)			< 5	< 5	8 (0.18)	< 5
		**ATE**	12 (0.06)	6 (0.10)			< 5	< 5	59 (1.36)	19 (0.43)
		**IS**	< 5	< 5			< 5	< 5	29 (0.67)	9 (0.20)
		**TIA**	< 5	< 5			< 5	< 5	18 (0.42)	5 (0.11)
		**MI**	9 (0.05)	< 5			< 5	< 5	14 (0.32)	5 (0.11)
		**HF**	23 (0.12)	11 (0.19)			< 5	< 5	73 (1.69)	27 (0.61)
		**HS**	< 5	< 5			< 5	< 5	6 (0.14)	< 5
		**MP**	< 5	< 5			< 5	< 5	< 5	< 5
**Cohort 3**			**N = 1,510,323**	**N = 54,102**			**N = 416,549**	**N = 36,748**	**N = 869,109**	**N = 706,435**
	**0 to 30 days**	**VTE**	244 (1.62)	< 5			28 (0.67)	< 5	321 (3.69)	< 5
		**DVT**	44 (0.29)	< 5			6 (0.14)	< 5	89 (1.02)	< 5
		**PE**	209 (1.38)	< 5			23 (0.55)	< 5	259 (2.98)	< 5
		**ATE**	29 (0.19)	< 5			< 5	< 5	211 (2.43)	< 5
		**IS**	< 5	< 5			< 5	< 5	102 (1.17)	< 5
		**TIA**	5 (0.03)	< 5			< 5	< 5	20 (0.23)	< 5
		**MI**	20 (0.13)	< 5			< 5	< 5	95 (1.09)	< 5
		**HF**	30 (0.20)	7 (1.29)			< 5	< 5	366 (4.21)	5 (0.07)
		**HS**	< 5	< 5			< 5	< 5	20 (0.23)	< 5
		**MP**	9 (0.06)	< 5			< 5	< 5	19 (0.22)	< 5
	**31 to 90 days**	**VTE**	44 (0.29)	< 5			< 5	< 5	84 (0.97)	< 5
		**DVT**	20 (0.13)	< 5			< 5	< 5	56 (0.64)	< 5
		**PE**	27 (0.18)	< 5			< 5	< 5	38 (0.44)	< 5
		**ATE**	11 (0.07)	< 5			< 5	< 5	107 (1.23)	< 5
		**IS**	< 5	< 5			< 5	< 5	53 (0.61)	< 5
		**TIA**	< 5	< 5			< 5	< 5	27 (0.31)	< 5

Cohort	Time window	Outcome	AURUM		CORIVA		GOLD		SIDIAP	
			Unvaccinated	Vaccinated	Unvaccinated	Vaccinated	Unvaccinated	Vaccinated	Unvaccinated	Vaccinated
		MI	9 (0.06)	< 5			< 5	< 5	35 (0.40)	< 5
		HF	15 (0.10)	< 5			< 5	< 5	139 (1.60)	< 5
		HS	< 5	< 5			< 5	< 5	7 (0.08)	< 5
		MP	8 (0.05)	< 5			< 5	< 5	8 (0.09)	< 5
	91 to 180 days	VTE	24 (0.16)	< 5			< 5	< 5	63 (0.72)	< 5
		DVT	11 (0.07)	< 5			< 5	< 5	35 (0.40)	< 5
		PE	14 (0.09)	< 5			< 5	< 5	32 (0.37)	< 5
		ATE	< 5	< 5			< 5	< 5	112 (1.29)	6 (0.08)
		IS	< 5	< 5			< 5	< 5	57 (0.66)	< 5
		TIA	< 5	< 5			< 5	< 5	22 (0.25)	< 5
		MI	< 5	< 5			< 5	< 5	36 (0.41)	< 5
		HF	10 (0.07)	< 5			< 5	< 5	121 (1.39)	< 5
		HS	< 5	< 5			< 5	< 5	9 (0.10)	< 5
		MP	< 5	< 5			< 5	< 5	7 (0.08)	< 5
	181 to 365 days	VTE	< 5	< 5			< 5	< 5	36 (0.41)	< 5
		DVT	< 5	< 5			< 5	< 5	22 (0.25)	< 5
		PE	< 5	< 5			< 5	< 5	16 (0.18)	< 5
		ATE	< 5	< 5			< 5	< 5	48 (0.55)	6 (0.08)
		IS	< 5	< 5			< 5	< 5	23 (0.26)	< 5
		TIA	< 5	< 5			< 5	< 5	9 (0.10)	< 5
		MI	< 5	< 5			< 5	< 5	17 (0.20)	< 5
		HF	5 (0.03)	< 5			< 5	< 5	61 (0.70)	< 5
		HS	< 5	< 5			< 5	< 5	< 5	< 5
		MP	< 5	< 5			< 5	< 5	< 5	< 5
**Cohort 4**			**N = 2,014,161**	**N = 1,335,671**	**N = 147,553**	**N = 15,683**	**N = 465,326**	**N = 365,096**	**N = 1,068,043**	**N = 580,329**
	0 to 30 days	VTE	328 (1.63)	9 (0.07)	116 (7.86)	< 5	36 (0.77)	< 5	350 (3.28)	18 (0.31)
		DVT	52 (0.26)	5 (0.04)	22 (1.49)	< 5	6 (0.13)	< 5	107 (1.00)	8 (0.14)
		PE	287 (1.42)	< 5	97 (6.57)	< 5	31 (0.67)	< 5	271 (2.54)	10 (0.17)
		ATE	25 (0.12)	< 5	116 (7.86)	< 5	< 5	< 5	233 (2.18)	9 (0.16)
		IS	< 5	< 5	69 (4.68)	< 5	< 5	< 5	114 (1.07)	6 (0.10)
		TIA	6 (0.03)	< 5	18 (1.22)	< 5	< 5	< 5	28 (0.26)	< 5
		MI	15 (0.07)	< 5	38 (2.58)	< 5	< 5	< 5	97 (0.91)	< 5
		HF	26 (0.13)	< 5	362 (24.53)	6 (3.83)	< 5	< 5	369 (3.45)	7 (0.12)
		HS	< 5	< 5	5 (0.34)	< 5	< 5	< 5	24 (0.22)	< 5

Mercadé-Besora N, *et al. Heart* 2024; 110:635–643. doi: 10.1136/heartjnl-2023-323483

Cohort	Time window	Outcome	AURUM		CORIVA		GOLD		SIDIAP	
			Unvaccinated	Vaccinated	Unvaccinated	Vaccinated	Unvaccinated	Vaccinated	Unvaccinated	Vaccinated
		MP	13 (0.06)	< 5	7 (0.47)	< 5	< 5	< 5	26 (0.24)	< 5
	31 to 90 days	VTE	56 (0.28)	< 5	54 (3.66)	< 5	< 5	< 5	93 (0.87)	6 (0.10)
		DVT	20 (0.10)	< 5	32 (2.17)	< 5	< 5	< 5	62 (0.58)	< 5
		PE	39 (0.19)	< 5	26 (1.76)	< 5	< 5	< 5	41 (0.38)	< 5
		ATE	13 (0.06)	< 5	46 (3.12)	< 5	< 5	< 5	115 (1.08)	< 5
		IS	< 5	< 5	24 (1.63)	< 5	< 5	< 5	51 (0.48)	< 5
		TIA	< 5	< 5	6 (0.41)	< 5	< 5	< 5	31 (0.29)	< 5
		MI	9 (0.04)	< 5	19 (1.29)	< 5	< 5	< 5	41 (0.38)	< 5
		HF	14 (0.07)	6 (0.04)	174 (11.79)	< 5	< 5	< 5	143 (1.34)	< 5
		HS	< 5	< 5	< 5	< 5	< 5	< 5	10 (0.09)	< 5
		MP	7 (0.03)	< 5	5 (0.34)	< 5	< 5	< 5	12 (0.11)	< 5
	91 to 180 days	VTE	24 (0.12)	8 (0.06)	49 (3.32)	< 5	< 5	< 5	77 (0.72)	< 5
		DVT	11 (0.05)	7 (0.05)	31 (2.10)	< 5	< 5	< 5	42 (0.39)	< 5
		PE	14 (0.07)	< 5	19 (1.29)	< 5	< 5	< 5	38 (0.36)	< 5
		ATE	< 5	< 5	41 (2.78)	< 5	< 5	< 5	134 (1.25)	9 (0.16)
		IS	< 5	< 5	19 (1.29)	< 5	< 5	< 5	66 (0.62)	7 (0.12)
		TIA	< 5	< 5	12 (0.81)	< 5	< 5	< 5	31 (0.29)	< 5
		MI	< 5	< 5	14 (0.95)	< 5	< 5	< 5	40 (0.37)	< 5
		HF	9 (0.04)	< 5	208 (14.10)	< 5	< 5	< 5	145 (1.36)	9 (0.16)
		HS	< 5	< 5	< 5	< 5	< 5	< 5	15 (0.14)	< 5
		MP	< 5	< 5	5 (0.34)	< 5	< 5	< 5	13 (0.12)	< 5
	181 to 365 days	VTE	< 5	< 5	77 (5.22)	5 (3.19)	< 5	< 5	52 (0.49)	7 (0.12)
		DVT	< 5	< 5	46 (3.12)	< 5	< 5	< 5	36 (0.34)	5 (0.09)
		PE	< 5	< 5	35 (2.37)	< 5	< 5	< 5	18 (0.17)	< 5
		ATE	< 5	< 5	72 (4.88)	< 5	< 5	< 5	52 (0.49)	15 (0.26)
		IS	< 5	< 5	33 (2.24)	< 5	< 5	< 5	28 (0.26)	10 (0.17)
		TIA	< 5	< 5	18 (1.22)	< 5	< 5	< 5	10 (0.09)	< 5
		MI	< 5	< 5	27 (1.83)	< 5	< 5	< 5	15 (0.14)	5 (0.09)
		HF	< 5	< 5	298 (20.20)	< 5	< 5	< 5	58 (0.54)	< 5
		HS	< 5	< 5	8 (0.54)	< 5	< 5	< 5	5 (0.05)	< 5
		MP	< 5	< 5	5 (0.34)	< 5	< 5	< 5	6 (0.06)	< 5

VTE = Venous thromboembolism (DVT + PE), DVT = Deep vein thrombosis, PE = Pulmonary embolism, ATE = Arterial thrombosis/thromboembolism (IS + TIA + MI), IS = Ischemic stroke, TIA = Transient ischemic attack, MI = Myocardial infarction, HF = Heart failure, HS = Hemorrhagic stroke, MP = Myocarditis or Pericarditis

Mercadé-Besora N, *et al. Heart* 2024; 110:635–643. doi: 10.1136/heartjnl-2023-323483

Table S33: Number of records (and risk per 10,000 individuals) for post COVID-19 cardiac and thromboembolic complications, across cohorts and databases, stratified by exposure status (BNT162b2 vaccine). Only first outcome after COVID-19 captured.

Cohort	Time window	Outcome	AURUM		CORIVA		GOLD		SIDIAP	
			Unvaccinated	Vaccinated	Unvaccinated	Vaccinated	Unvaccinated	Vaccinated	Unvaccinated	Vaccinated
**Cohort 1**			**N = 348,052**	**N = 332,790**	**N = 24,073**	**N = 19,686**	**N = 169,459**	**N = 32,755**	**N = 223,960**	**N = 88,896**
	**0 to 30 days**	**VTE**	106 (3.05)	76 (2.28)	76 (31.57)	40 (20.32)	8 (0.47)	< 5	75 (3.35)	94 (10.57)
		**DVT**	21 (0.60)	15 (0.45)	14 (5.82)	13 (6.60)	< 5	< 5	20 (0.89)	29 (3.26)
		**PE**	90 (2.59)	64 (1.92)	64 (26.59)	28 (14.22)	6 (0.35)	< 5	60 (2.68)	75 (8.44)
		**ATE**	27 (0.78)	45 (1.35)	114 (47.36)	61 (30.99)	6 (0.35)	< 5	79 (3.53)	206 (23.17)
		**IS**	9 (0.26)	5 (0.15)	68 (28.25)	26 (13.21)	< 5	< 5	46 (2.05)	116 (13.05)
		**TIA**	5 (0.14)	11 (0.33)	22 (9.14)	15 (7.62)	< 5	< 5	7 (0.31)	41 (4.61)
		**MI**	14 (0.40)	29 (0.87)	34 (14.12)	27 (13.72)	< 5	< 5	28 (1.25)	61 (6.86)
		**HF**	59 (1.70)	126 (3.79)	393 (163.25)	231 (117.34)	9 (0.53)	< 5	299 (13.35)	634 (71.32)
		**HS**	< 5	< 5	< 5	< 5	< 5	< 5	7 (0.31)	14 (1.57)
		**MP**	< 5	< 5	< 5	< 5	< 5	< 5	< 5	< 5
	**31 to 90 days**	**VTE**	18 (0.52)	25 (0.75)	37 (15.37)	23 (11.68)	< 5	< 5	15 (0.67)	43 (4.84)
		**DVT**	9 (0.26)	7 (0.21)	18 (7.48)	13 (6.60)	< 5	< 5	9 (0.40)	28 (3.15)
		**PE**	9 (0.26)	18 (0.54)	22 (9.14)	10 (5.08)	< 5	< 5	6 (0.27)	19 (2.14)
		**ATE**	9 (0.26)	23 (0.69)	27 (11.22)	33 (16.76)	< 5	< 5	39 (1.74)	125 (14.06)
		**IS**	< 5	< 5	15 (6.23)	13 (6.60)	< 5	< 5	20 (0.89)	69 (7.76)
		**TIA**	5 (0.14)	10 (0.30)	7 (2.91)	14 (7.11)	< 5	< 5	13 (0.58)	32 (3.60)
		**MI**	5 (0.14)	11 (0.33)	9 (3.74)	8 (4.06)	< 5	< 5	9 (0.40)	28 (3.15)
		**HF**	32 (0.92)	70 (2.10)	136 (56.49)	135 (68.58)	< 5	< 5	83 (3.71)	288 (32.40)
		**HS**	< 5	< 5	< 5	< 5	< 5	< 5	< 5	12 (1.35)
		**MP**	< 5	< 5	< 5	< 5	< 5	< 5	< 5	< 5
	**91 to 180 days**	**VTE**	10 (0.29)	< 5	14 (5.82)	22 (11.18)	< 5	< 5	19 (0.85)	35 (3.94)
		**DVT**	< 5	< 5	6 (2.49)	11 (5.59)	< 5	< 5	7 (0.31)	19 (2.14)
		**PE**	6 (0.17)	< 5	9 (3.74)	12 (6.10)	< 5	< 5	12 (0.54)	18 (2.02)
		**ATE**	15 (0.43)	18 (0.54)	29 (12.05)	35 (17.78)	< 5	< 5	26 (1.16)	102 (11.47)
		**IS**	< 5	6 (0.18)	14 (5.82)	14 (7.11)	< 5	< 5	14 (0.63)	56 (6.30)
		**TIA**	8 (0.23)	6 (0.18)	8 (3.32)	10 (5.08)	< 5	< 5	7 (0.31)	33 (3.71)
		**MI**	5 (0.14)	6 (0.18)	8 (3.32)	15 (7.62)	< 5	< 5	6 (0.27)	22 (2.47)
		**HF**	40 (1.15)	54 (1.62)	147 (61.06)	159 (80.77)	< 5	< 5	81 (3.62)	226 (25.42)
		**HS**	< 5	< 5	< 5	< 5	< 5	< 5	< 5	< 5
		**MP**	< 5	< 5	< 5	< 5	< 5	< 5	< 5	< 5

Cohort	Time window	Outcome	AURUM		CORIVA		GOLD		SIDIAP	
			Unvaccinated	Vaccinated	Unvaccinated	Vaccinated	Unvaccinated	Vaccinated	Unvaccinated	Vaccinated
	181 to 365 days	VTE	11 (0.32)	10 (0.30)	27 (11.22)	24 (12.19)	< 5	< 5	5 (0.22)	10 (1.12)
		DVT	< 5	6 (0.18)	10 (4.15)	14 (7.11)	< 5	< 5	< 5	7 (0.79)
		PE	9 (0.26)	< 5	21 (8.72)	11 (5.59)	< 5	< 5	< 5	< 5
		ATE	14 (0.40)	14 (0.42)	47 (19.52)	58 (29.46)	< 5	< 5	34 (1.52)	38 (4.27)
		IS	< 5	< 5	26 (10.80)	27 (13.72)	< 5	< 5	17 (0.76)	23 (2.59)
		TIA	9 (0.26)	< 5	15 (6.23)	24 (12.19)	< 5	< 5	10 (0.45)	9 (1.01)
		MI	< 5	7 (0.21)	10 (4.15)	12 (6.10)	< 5	< 5	7 (0.31)	7 (0.79)
		HF	47 (1.35)	35 (1.05)	226 (93.88)	223 (113.28)	< 5	< 5	70 (3.13)	117 (13.16)
		HS	< 5	< 5	< 5	< 5	< 5	< 5	< 5	7 (0.79)
		MP	< 5	< 5	< 5	< 5	< 5	< 5	< 5	< 5
Cohort 2			N = 1,976,163	N = 594,262			N = 584,309	N = 180,670	N = 433,111	N = 445,581
	0 to 30 days	VTE	240 (1.21)	62 (1.04)			29 (0.50)	5 (0.28)	259 (5.98)	265 (5.95)
		DVT	39 (0.20)	18 (0.30)			7 (0.12)	< 5	63 (1.45)	96 (2.15)
		PE	205 (1.04)	45 (0.76)			23 (0.39)	< 5	215 (4.96)	189 (4.24)
		ATE	39 (0.20)	29 (0.49)			< 5	< 5	172 (3.97)	489 (10.97)
		IS	7 (0.04)	< 5			< 5	< 5	94 (2.17)	267 (5.99)
		TIA	6 (0.03)	11 (0.19)			< 5	< 5	17 (0.39)	90 (2.02)
		MI	26 (0.13)	16 (0.27)			< 5	< 5	65 (1.50)	163 (3.66)
		HF	50 (0.25)	43 (0.72)			6 (0.10)	< 5	380 (8.77)	1,137 (25.52)
		HS	6 (0.03)	< 5			< 5	< 5	18 (0.42)	61 (1.37)
		MP	11 (0.06)	< 5			< 5	< 5	14 (0.32)	22 (0.49)
	31 to 90 days	VTE	46 (0.23)	21 (0.35)			< 5	< 5	57 (1.32)	123 (2.76)
		DVT	25 (0.13)	7 (0.12)			< 5	< 5	31 (0.72)	77 (1.73)
		PE	24 (0.12)	14 (0.24)			< 5	< 5	32 (0.74)	55 (1.23)
		ATE	17 (0.09)	36 (0.61)			< 5	< 5	84 (1.94)	307 (6.89)
		IS	< 5	5 (0.08)			< 5	< 5	43 (0.99)	162 (3.64)
		TIA	< 5	9 (0.15)			< 5	< 5	24 (0.55)	85 (1.91)
		MI	11 (0.06)	22 (0.37)			< 5	< 5	24 (0.55)	79 (1.77)
		HF	27 (0.14)	34 (0.57)			< 5	< 5	131 (3.02)	534 (11.98)
		HS	< 5	< 5			< 5	< 5	7 (0.16)	19 (0.43)
		MP	6 (0.03)	< 5			< 5	< 5	< 5	< 5
	91 to 180 days	VTE	25 (0.13)	9 (0.15)			5 (0.09)	< 5	53 (1.22)	84 (1.89)
		DVT	11 (0.06)	6 (0.10)			< 5	< 5	29 (0.67)	52 (1.17)
		PE	14 (0.07)	< 5			< 5	< 5	27 (0.62)	39 (0.88)

Cohort	Time window	Outcome	AURUM		CORIVA		GOLD		SIDIAP	
			Unvaccinated	Vaccinated	Unvaccinated	Vaccinated	Unvaccinated	Vaccinated	Unvaccinated	Vaccinated
		ATE	13 (0.07)	15 (0.25)			< 5	< 5	80 (1.85)	265 (5.95)
		IS	< 5	< 5			< 5	< 5	43 (0.99)	148 (3.32)
		TIA	7 (0.04)	5 (0.08)			< 5	< 5	19 (0.44)	72 (1.62)
		MI	7 (0.04)	10 (0.17)			< 5	< 5	21 (0.48)	62 (1.39)
		HF	19 (0.10)	24 (0.40)			< 5	< 5	101 (2.33)	438 (9.83)
		HS	< 5	< 5			< 5	< 5	7 (0.16)	33 (0.74)
		MP	< 5	< 5			< 5	< 5	7 (0.16)	< 5
	181 to 365 days	VTE	9 (0.05)	8 (0.13)			< 5	< 5	15 (0.35)	30 (0.67)
		DVT	< 5	< 5			< 5	< 5	9 (0.21)	20 (0.45)
		PE	7 (0.04)	7 (0.12)			< 5	< 5	8 (0.18)	12 (0.27)
		ATE	9 (0.05)	7 (0.12)			< 5	< 5	50 (1.15)	103 (2.31)
		IS	< 5	< 5			< 5	< 5	25 (0.58)	51 (1.14)
		TIA	< 5	< 5			< 5	< 5	16 (0.37)	29 (0.65)
		MI	7 (0.04)	< 5			< 5	< 5	11 (0.25)	29 (0.65)
		HF	22 (0.11)	12 (0.20)			< 5	< 5	63 (1.45)	178 (3.99)
		HS	< 5	< 5			< 5	< 5	5 (0.12)	9 (0.20)
		MP	< 5	< 5			< 5	< 5	< 5	< 5
Cohort 3			N = 1,510,323	N = 54,102			N = 416,549	N = 36,748	N = 869,109	N = 706,435
	0 to 30 days	VTE	244 (1.62)	< 5			28 (0.67)	< 5	321 (3.69)	114 (1.61)
		DVT	44 (0.29)	< 5			6 (0.14)	< 5	89 (1.02)	51 (0.72)
		PE	209 (1.38)	< 5			23 (0.55)	< 5	259 (2.98)	71 (1.01)
		ATE	29 (0.19)	< 5			< 5	< 5	211 (2.43)	197 (2.79)
		IS	< 5	< 5			< 5	< 5	102 (1.17)	95 (1.34)
		TIA	5 (0.03)	< 5			< 5	< 5	20 (0.23)	42 (0.59)
		MI	20 (0.13)	< 5			< 5	< 5	95 (1.09)	78 (1.10)
		HF	30 (0.20)	10 (1.85)			< 5	< 5	366 (4.21)	163 (2.31)
		HS	< 5	< 5			< 5	< 5	20 (0.23)	26 (0.37)
		MP	9 (0.06)	< 5			< 5	< 5	19 (0.22)	13 (0.18)
	31 to 90 days	VTE	41 (0.27)	< 5			< 5	< 5	80 (0.92)	63 (0.89)
		DVT	19 (0.13)	< 5			< 5	< 5	53 (0.61)	47 (0.67)
		PE	25 (0.17)	< 5			< 5	< 5	36 (0.41)	21 (0.30)
		ATE	11 (0.07)	< 5			< 5	< 5	106 (1.22)	142 (2.01)
		IS	< 5	< 5			< 5	< 5	53 (0.61)	54 (0.76)
		TIA	< 5	< 5			< 5	< 5	27 (0.31)	27 (0.38)

Cohort	Time window	Outcome	AURUM		CORIVA		GOLD		SIDIAP	
			Unvaccinated	Vaccinated	Unvaccinated	Vaccinated	Unvaccinated	Vaccinated	Unvaccinated	Vaccinated
		MI	9 (0.06)	< 5			< 5	< 5	34 (0.39)	69 (0.98)
		HF	15 (0.10)	< 5			< 5	< 5	132 (1.52)	108 (1.53)
		HS	< 5	< 5			< 5	< 5	7 (0.08)	14 (0.20)
		MP	8 (0.05)	< 5			< 5	< 5	8 (0.09)	9 (0.13)
	91 to 180 days	VTE	23 (0.15)	< 5			< 5	< 5	56 (0.64)	67 (0.95)
		DVT	10 (0.07)	< 5			< 5	< 5	32 (0.37)	43 (0.61)
		PE	14 (0.09)	< 5			< 5	< 5	28 (0.32)	25 (0.35)
		ATE	< 5	< 5			< 5	< 5	101 (1.16)	137 (1.94)
		IS	< 5	< 5			< 5	< 5	49 (0.56)	62 (0.88)
		TIA	< 5	< 5			< 5	< 5	20 (0.23)	28 (0.40)
		MI	< 5	< 5			< 5	< 5	34 (0.39)	53 (0.75)
		HF	10 (0.07)	< 5			< 5	< 5	110 (1.27)	97 (1.37)
		HS	< 5	< 5			< 5	< 5	9 (0.10)	10 (0.14)
		MP	< 5	< 5			< 5	< 5	7 (0.08)	9 (0.13)
	181 to 365 days	VTE	< 5	< 5			< 5	< 5	33 (0.38)	13 (0.18)
		DVT	< 5	< 5			< 5	< 5	20 (0.23)	11 (0.16)
		PE	< 5	< 5			< 5	< 5	15 (0.17)	< 5
		ATE	< 5	< 5			< 5	< 5	39 (0.45)	36 (0.51)
		IS	< 5	< 5			< 5	< 5	18 (0.21)	13 (0.18)
		TIA	< 5	< 5			< 5	< 5	8 (0.09)	5 (0.07)
		MI	< 5	< 5			< 5	< 5	14 (0.16)	21 (0.30)
		HF	5 (0.03)	< 5			< 5	< 5	53 (0.61)	25 (0.35)
		HS	< 5	< 5			< 5	< 5	< 5	< 5
		MP	< 5	< 5			< 5	< 5	< 5	< 5
**Cohort 4**			**N = 2,014,161**	**N = 1,335,671**	**N = 147,553**	**N = 15,683**	**N = 465,326**	**N = 365,096**	**N = 1,068,043**	**N = 580,329**
	0 to 30 days	VTE	328 (1.63)	20 (0.15)	116 (7.86)	< 5	36 (0.77)	< 5	350 (3.28)	66 (1.14)
		DVT	52 (0.26)	9 (0.07)	22 (1.49)	< 5	6 (0.13)	< 5	107 (1.00)	35 (0.60)
		PE	287 (1.42)	11 (0.08)	97 (6.57)	< 5	31 (0.67)	< 5	271 (2.54)	35 (0.60)
		ATE	25 (0.12)	< 5	116 (7.86)	< 5	< 5	< 5	233 (2.18)	55 (0.95)
		IS	< 5	< 5	69 (4.68)	< 5	< 5	< 5	114 (1.07)	29 (0.50)
		TIA	6 (0.03)	< 5	18 (1.22)	< 5	< 5	< 5	28 (0.26)	7 (0.12)
		MI	15 (0.07)	< 5	38 (2.58)	< 5	< 5	< 5	97 (0.91)	20 (0.34)
		HF	26 (0.13)	< 5	362 (24.53)	8 (5.10)	< 5	< 5	369 (3.45)	44 (0.76)
		HS	< 5	< 5	5 (0.34)	< 5	< 5	< 5	24 (0.22)	7 (0.12)

Cohort	Time window	Outcome	AURUM		CORIVA		GOLD		SIDIAP	
			Unvaccinated	Vaccinated	Unvaccinated	Vaccinated	Unvaccinated	Vaccinated	Unvaccinated	Vaccinated
		**MP**	13 (0.06)	< 5	7 (0.47)	< 5	< 5	< 5	26 (0.24)	11 (0.19)
	**31 to 90 days**	**VTE**	53 (0.26)	7 (0.05)	49 (3.32)	< 5	< 5	< 5	88 (0.82)	32 (0.55)
		**DVT**	19 (0.09)	< 5	29 (1.97)	< 5	< 5	< 5	58 (0.54)	23 (0.40)
		**PE**	37 (0.18)	< 5	23 (1.56)	< 5	< 5	< 5	39 (0.37)	10 (0.17)
		**ATE**	13 (0.06)	< 5	43 (2.91)	< 5	< 5	< 5	114 (1.07)	51 (0.88)
		**IS**	< 5	< 5	22 (1.49)	< 5	< 5	< 5	51 (0.48)	21 (0.36)
		**TIA**	< 5	< 5	6 (0.41)	< 5	< 5	< 5	31 (0.29)	12 (0.21)
		**MI**	9 (0.04)	< 5	17 (1.15)	< 5	< 5	< 5	40 (0.37)	21 (0.36)
		**HF**	14 (0.07)	6 (0.04)	160 (10.84)	5 (3.19)	< 5	< 5	136 (1.27)	28 (0.48)
		**HS**	< 5	< 5	< 5	< 5	< 5	< 5	10 (0.09)	5 (0.09)
		**MP**	7 (0.03)	< 5	< 5	< 5	< 5	< 5	12 (0.11)	8 (0.14)
	**91 to 180 days**	**VTE**	23 (0.11)	9 (0.07)	40 (2.71)	< 5	< 5	< 5	71 (0.66)	40 (0.69)
		**DVT**	10 (0.05)	7 (0.05)	25 (1.69)	< 5	< 5	< 5	40 (0.37)	25 (0.43)
		**PE**	14 (0.07)	< 5	16 (1.08)	< 5	< 5	< 5	34 (0.32)	18 (0.31)
		**ATE**	< 5	< 5	38 (2.58)	< 5	< 5	< 5	122 (1.14)	68 (1.17)
		**IS**	< 5	< 5	18 (1.22)	< 5	< 5	< 5	58 (0.54)	34 (0.59)
		**TIA**	< 5	< 5	12 (0.81)	< 5	< 5	< 5	28 (0.26)	14 (0.24)
		**MI**	< 5	< 5	12 (0.81)	< 5	< 5	< 5	38 (0.36)	20 (0.34)
		**HF**	9 (0.04)	< 5	187 (12.67)	< 5	< 5	< 5	132 (1.24)	35 (0.60)
		**HS**	< 5	< 5	< 5	< 5	< 5	< 5	14 (0.13)	< 5
		**MP**	< 5	< 5	< 5	< 5	< 5	< 5	13 (0.12)	< 5
	**181 to 365 days**	**VTE**	< 5	< 5	53 (3.59)	< 5	< 5	< 5	44 (0.41)	7 (0.12)
		**DVT**	< 5	< 5	33 (2.24)	< 5	< 5	< 5	31 (0.29)	6 (0.10)
		**PE**	< 5	< 5	23 (1.56)	< 5	< 5	< 5	15 (0.14)	< 5
		**ATE**	< 5	< 5	63 (4.27)	< 5	< 5	< 5	41 (0.38)	9 (0.16)
		**IS**	< 5	< 5	29 (1.97)	< 5	< 5	< 5	20 (0.19)	6 (0.10)
		**TIA**	< 5	< 5	18 (1.22)	< 5	< 5	< 5	9 (0.08)	< 5
		**MI**	< 5	< 5	21 (1.42)	< 5	< 5	< 5	13 (0.12)	< 5
		**HF**	< 5	< 5	240 (16.27)	< 5	< 5	< 5	49 (0.46)	5 (0.09)
		**HS**	< 5	< 5	8 (0.54)	< 5	< 5	< 5	5 (0.05)	< 5
		**MP**	< 5	< 5	< 5	< 5	< 5	< 5	6 (0.06)	< 5

VTE = Venous thromboembolism (DVT + PE), DVT = Deep vein thrombosis, PE = Pulmonary embolism, ATE = Arterial thrombosis/thromboembolism (IS + TIA + MI), IS = Ischemic stroke, TIA = Transient ischemic attack, MI = Myocardial infarction, HF = Heart failure, HS = Hemorrhagic stroke, MP = Myocarditis or Pericarditis

Table S34: Number of records (and risk per 10,000 individuals) for post COVID-19 cardiac and thromboembolic complications, across cohorts and databases, stratified by vaccine (BNT162b2 - ChAdOx1).

Cohort	Time window	Outcome	AURUM		GOLD	
			BNT162b2	ChAdOx1	BNT162b2	ChAdOx1
**Cohort 1**			**N = 332,790**	**N = 219,804**	**N = 32,755**	**N = 82,406**
	**0 to 30 days**	**VTE**	76 (2.28)	41 (1.87)	< 5	< 5
		**DVT**	15 (0.45)	12 (0.55)	< 5	< 5
		**PE**	64 (1.92)	31 (1.41)	< 5	< 5
		**ATE**	45 (1.35)	25 (1.14)	< 5	6 (0.73)
		**IS**	5 (0.15)	< 5	< 5	< 5
		**TIA**	11 (0.33)	7 (0.32)	< 5	< 5
		**MI**	29 (0.87)	17 (0.77)	< 5	< 5
		**HF**	126 (3.79)	72 (3.28)	< 5	5 (0.61)
		**HS**	< 5	< 5	< 5	< 5
		**MP**	< 5	< 5	< 5	< 5
	**31 to 90 days**	**VTE**	26 (0.78)	14 (0.64)	< 5	< 5
		**DVT**	7 (0.21)	8 (0.36)	< 5	< 5
		**PE**	19 (0.57)	7 (0.32)	< 5	< 5
		**ATE**	23 (0.69)	20 (0.91)	< 5	< 5
		**IS**	< 5	< 5	< 5	< 5
		**TIA**	10 (0.30)	9 (0.41)	< 5	< 5
		**MI**	11 (0.33)	9 (0.41)	< 5	< 5
		**HF**	71 (2.13)	42 (1.91)	< 5	5 (0.61)
		**HS**	< 5	< 5	< 5	< 5
		**MP**	< 5	< 5	< 5	< 5
	**91 to 180 days**	**VTE**	6 (0.18)	15 (0.68)	< 5	< 5
		**DVT**	< 5	5 (0.23)	< 5	< 5
		**PE**	< 5	10 (0.45)	< 5	< 5
		**ATE**	18 (0.54)	10 (0.45)	< 5	6 (0.73)
		**IS**	6 (0.18)	< 5	< 5	< 5
		**TIA**	6 (0.18)	6 (0.27)	< 5	< 5
		**MI**	6 (0.18)	< 5	< 5	5 (0.61)
		**HF**	57 (1.71)	38 (1.73)	< 5	< 5
		**HS**	< 5	< 5	< 5	< 5
		**MP**	< 5	< 5	< 5	< 5

Cohort	Time window	Outcome	AURUM		GOLD	
			BNT162b2	ChAdOx1	BNT162b2	ChAdOx1
	**181 to 365 days**	**VTE**	10 (0.30)	< 5	< 5	< 5
		**DVT**	6 (0.18)	< 5	< 5	< 5
		**PE**	< 5	< 5	< 5	< 5
		**ATE**	14 (0.42)	9 (0.41)	< 5	< 5
		**IS**	< 5	< 5	< 5	< 5
		**TIA**	< 5	< 5	< 5	< 5
		**MI**	7 (0.21)	< 5	< 5	< 5
		**HF**	38 (1.14)	20 (0.91)	< 5	5 (0.61)
		**HS**	< 5	< 5	< 5	< 5
		**MP**	< 5	< 5	< 5	< 5
**Cohort 2**			**N = 594,262**	**N = 969,262**	**N = 180,670**	**N = 302,999**
	**0 to 30 days**	**VTE**	62 (1.04)	158 (1.63)	5 (0.28)	19 (0.63)
		**DVT**	18 (0.30)	47 (0.48)	< 5	< 5
		**PE**	45 (0.76)	120 (1.24)	< 5	15 (0.50)
		**ATE**	29 (0.49)	75 (0.77)	< 5	< 5
		**IS**	< 5	12 (0.12)	< 5	< 5
		**TIA**	11 (0.19)	13 (0.13)	< 5	< 5
		**MI**	16 (0.27)	52 (0.54)	< 5	< 5
		**HF**	43 (0.72)	103 (1.06)	< 5	11 (0.36)
		**HS**	< 5	< 5	< 5	< 5
		**MP**	< 5	6 (0.06)	< 5	< 5
	**31 to 90 days**	**VTE**	21 (0.35)	55 (0.57)	< 5	9 (0.30)
		**DVT**	7 (0.12)	25 (0.26)	< 5	5 (0.17)
		**PE**	14 (0.24)	32 (0.33)	< 5	< 5
		**ATE**	36 (0.61)	57 (0.59)	< 5	5 (0.17)
		**IS**	5 (0.08)	6 (0.06)	< 5	< 5
		**TIA**	9 (0.15)	25 (0.26)	< 5	< 5
		**MI**	22 (0.37)	28 (0.29)	< 5	< 5
		**HF**	35 (0.59)	68 (0.70)	< 5	7 (0.23)
		**HS**	< 5	< 5	< 5	< 5
		**MP**	< 5	< 5	< 5	< 5
	**91 to 180 days**	**VTE**	10 (0.17)	30 (0.31)	< 5	< 5
		**DVT**	6 (0.10)	16 (0.17)	< 5	< 5
		**PE**	5 (0.08)	16 (0.17)	< 5	< 5

Mercadé-Besora N, *et al. Heart* 2024; 110:635–643. doi: 10.1136/heartjnl-2023-323483

Cohort	Time window	Outcome	AURUM		GOLD	
			BNT162b2	ChAdOx1	BNT162b2	ChAdOx1
		ATE	15 (0.25)	28 (0.29)	< 5	< 5
		IS	< 5	< 5	< 5	< 5
		TIA	5 (0.08)	13 (0.13)	< 5	< 5
		MI	10 (0.17)	15 (0.15)	< 5	< 5
		HF	25 (0.42)	44 (0.45)	< 5	< 5
		HS	< 5	< 5	< 5	< 5
		MP	< 5	< 5	< 5	< 5
	181 to 365 days	VTE	8 (0.13)	5 (0.05)	< 5	< 5
		DVT	< 5	< 5	< 5	< 5
		PE	7 (0.12)	< 5	< 5	< 5
		ATE	7 (0.12)	11 (0.11)	< 5	< 5
		IS	< 5	< 5	< 5	< 5
		TIA	< 5	< 5	< 5	< 5
		MI	< 5	7 (0.07)	< 5	< 5
		HF	12 (0.20)	23 (0.24)	< 5	< 5
		HS	< 5	< 5	< 5	< 5
		MP	< 5	< 5	< 5	< 5
Cohort 3			N = 54,102	N = 1,473,602	N = 36,748	N = 423,876
	0 to 30 days	VTE	< 5	139 (0.94)	< 5	16 (0.38)
		DVT	< 5	41 (0.28)	< 5	< 5
		PE	< 5	101 (0.69)	< 5	12 (0.28)
		ATE	< 5	47 (0.32)	< 5	12 (0.28)
		IS	< 5	5 (0.03)	< 5	< 5
		TIA	< 5	16 (0.11)	< 5	< 5
		MI	< 5	26 (0.18)	< 5	9 (0.21)
		HF	10 (1.85)	28 (0.19)	< 5	< 5
		HS	< 5	< 5	< 5	< 5
		MP	< 5	6 (0.04)	< 5	< 5
	31 to 90 days	VTE	< 5	44 (0.30)	< 5	7 (0.17)
		DVT	< 5	26 (0.18)	< 5	< 5
		PE	< 5	18 (0.12)	< 5	< 5
		ATE	< 5	31 (0.21)	< 5	7 (0.17)
		IS	< 5	< 5	< 5	< 5
		TIA	< 5	13 (0.09)	< 5	< 5

Cohort	Time window	Outcome	AURUM		GOLD	
			BNT162b2	ChAdOx1	BNT162b2	ChAdOx1
		MI	< 5	16 (0.11)	< 5	< 5
		HF	< 5	23 (0.16)	< 5	< 5
		HS	< 5	< 5	< 5	< 5
		MP	< 5	7 (0.05)	< 5	< 5
	91 to 180 days	VTE	< 5	26 (0.18)	< 5	< 5
		DVT	< 5	15 (0.10)	< 5	< 5
		PE	< 5	14 (0.10)	< 5	< 5
		ATE	< 5	26 (0.18)	< 5	< 5
		IS	< 5	< 5	< 5	< 5
		TIA	< 5	8 (0.05)	< 5	< 5
		MI	< 5	17 (0.12)	< 5	< 5
		HF	< 5	12 (0.08)	< 5	< 5
		HS	< 5	< 5	< 5	< 5
		MP	< 5	< 5	< 5	< 5
	181 to 365 days	VTE	< 5	11 (0.07)	< 5	< 5
		DVT	< 5	5 (0.03)	< 5	< 5
		PE	< 5	7 (0.05)	< 5	< 5
		ATE	< 5	< 5	< 5	< 5
		IS	< 5	< 5	< 5	< 5
		TIA	< 5	< 5	< 5	< 5
		MI	< 5	< 5	< 5	< 5
		HF	< 5	< 5	< 5	< 5
		HS	< 5	< 5	< 5	< 5
		MP	< 5	< 5	< 5	< 5
Cohort 4			N = 1,335,671	N = 542,670	N = 365,096	N = 147,744
	0 to 30 days	VTE	20 (0.15)	27 (0.50)	< 5	8 (0.54)
		DVT	9 (0.07)	7 (0.13)	< 5	< 5
		PE	11 (0.08)	21 (0.39)	< 5	6 (0.41)
		ATE	< 5	5 (0.09)	< 5	< 5
		IS	< 5	< 5	< 5	< 5
		TIA	< 5	< 5	< 5	< 5
		MI	< 5	< 5	< 5	< 5
		HF	< 5	< 5	< 5	< 5
		HS	< 5	< 5	< 5	< 5

Mercadé-Besora N, *et al. Heart* 2024; 110:635–643. doi: 10.1136/heartjnl-2023-323483

Cohort	Time window	Outcome	AURUM		GOLD	
			BNT162b2	ChAdOx1	BNT162b2	ChAdOx1
		**MP**	< 5	< 5	< 5	< 5
	**31 to 90 days**	**VTE**	7 (0.05)	14 (0.26)	< 5	< 5
		**DVT**	< 5	8 (0.15)	< 5	< 5
		**PE**	< 5	6 (0.11)	< 5	< 5
		**ATE**	< 5	< 5	< 5	< 5
		**IS**	< 5	< 5	< 5	< 5
		**TIA**	< 5	< 5	< 5	< 5
		**MI**	< 5	< 5	< 5	< 5
		**HF**	6 (0.04)	< 5	< 5	< 5
		**HS**	< 5	< 5	< 5	< 5
		**MP**	< 5	< 5	< 5	< 5
	**91 to 180 days**	**VTE**	9 (0.07)	< 5	< 5	< 5
		**DVT**	7 (0.05)	< 5	< 5	< 5
		**PE**	< 5	< 5	< 5	< 5
		**ATE**	< 5	< 5	< 5	< 5
		**IS**	< 5	< 5	< 5	< 5
		**TIA**	< 5	< 5	< 5	< 5
		**MI**	< 5	< 5	< 5	< 5
		**HF**	< 5	< 5	< 5	< 5
		**HS**	< 5	< 5	< 5	< 5
		**MP**	< 5	< 5	< 5	< 5
	**181 to 365 days**	**VTE**	< 5	< 5	< 5	< 5
		**DVT**	< 5	< 5	< 5	< 5
		**PE**	< 5	< 5	< 5	< 5
		**ATE**	< 5	< 5	< 5	< 5
		**IS**	< 5	< 5	< 5	< 5
		**TIA**	< 5	< 5	< 5	< 5
		**MI**	< 5	< 5	< 5	< 5
		**HF**	< 5	< 5	< 5	< 5
		**HS**	< 5	< 5	< 5	< 5
		**MP**	< 5	< 5	< 5	< 5

VTE = Venous thromboembolism (DVT + PE), DVT = Deep vein thrombosis, PE = Pulmonary embolism, ATE = Arterial thrombosis/thromboembolism (IS + TIA + MI), IS = Ischemic stroke, TIA = Transient ischemic attack, MI = Myocardial infarction, HF = Heart failure, HS = Hemorrhagic stroke, MP = Myocarditis or Pericarditis

Table S35: Number of records (and risk per 10,000 individuals) for post COVID-19 cardiac and thromboembolic complications, across cohorts and databases, stratified by vaccine (BNT162b2 - ChAdOx1). Follow-up ends at first vaccine dose after index date.

Cohort	Time window	Outcome	AURUM		GOLD	
			BNT162b2	ChAdOx1	BNT162b2	ChAdOx1
**Cohort 1**			**N = 332,790**	**N = 219,804**	**N = 32,755**	**N = 82,406**
	**0 to 30 days**	**VTE**	31 (0.93)	14 (0.64)	< 5	< 5
		**DVT**	8 (0.24)	< 5	< 5	< 5
		**PE**	24 (0.72)	13 (0.59)	< 5	< 5
		**ATE**	18 (0.54)	10 (0.45)	< 5	< 5
		**IS**	< 5	< 5	< 5	< 5
		**TIA**	< 5	< 5	< 5	< 5
		**MI**	14 (0.42)	6 (0.27)	< 5	< 5
		**HF**	45 (1.35)	28 (1.27)	< 5	< 5
		**HS**	< 5	< 5	< 5	< 5
		**MP**	< 5	< 5	< 5	< 5
	**31 to 90 days**	**VTE**	15 (0.45)	5 (0.23)	< 5	< 5
		**DVT**	< 5	< 5	< 5	< 5
		**PE**	11 (0.33)	< 5	< 5	< 5
		**ATE**	8 (0.24)	8 (0.36)	< 5	< 5
		**IS**	< 5	< 5	< 5	< 5
		**TIA**	< 5	< 5	< 5	< 5
		**MI**	< 5	< 5	< 5	< 5
		**HF**	36 (1.08)	20 (0.91)	< 5	< 5
		**HS**	< 5	< 5	< 5	< 5
		**MP**	< 5	< 5	< 5	< 5
	**91 to 180 days**	**VTE**	< 5	5 (0.23)	< 5	< 5
		**DVT**	< 5	< 5	< 5	< 5
		**PE**	< 5	< 5	< 5	< 5
		**ATE**	10 (0.30)	< 5	< 5	< 5
		**IS**	< 5	< 5	< 5	< 5
		**TIA**	< 5	< 5	< 5	< 5
		**MI**	< 5	< 5	< 5	< 5
		**HF**	32 (0.96)	19 (0.86)	< 5	< 5
		**HS**	< 5	< 5	< 5	< 5
		**MP**	< 5	< 5	< 5	< 5

Cohort	Time window	Outcome	AURUM		GOLD	
			BNT162b2	ChAdOx1	BNT162b2	ChAdOx1
	181 to 365 days	VTE	7 (0.21)	< 5	< 5	< 5
		DVT	< 5	< 5	< 5	< 5
		PE	< 5	< 5	< 5	< 5
		ATE	11 (0.33)	8 (0.36)	< 5	< 5
		IS	< 5	< 5	< 5	< 5
		TIA	< 5	< 5	< 5	< 5
		MI	6 (0.18)	< 5	< 5	< 5
		HF	35 (1.05)	18 (0.82)	< 5	5 (0.61)
		HS	< 5	< 5	< 5	< 5
		MP	< 5	< 5	< 5	< 5
Cohort 2			N = 594,262	N = 969,262	N = 180,670	N = 302,999
	0 to 30 days	VTE	14 (0.24)	40 (0.41)	< 5	< 5
		DVT	6 (0.10)	13 (0.13)	< 5	< 5
		PE	8 (0.13)	30 (0.31)	< 5	< 5
		ATE	14 (0.24)	11 (0.11)	< 5	< 5
		IS	< 5	< 5	< 5	< 5
		TIA	< 5	< 5	< 5	< 5
		MI	8 (0.13)	9 (0.09)	< 5	< 5
		HF	15 (0.25)	32 (0.33)	< 5	< 5
		HS	< 5	< 5	< 5	< 5
		MP	< 5	< 5	< 5	< 5
	31 to 90 days	VTE	5 (0.08)	8 (0.08)	< 5	< 5
		DVT	< 5	5 (0.05)	< 5	< 5
		PE	< 5	< 5	< 5	< 5
		ATE	9 (0.15)	9 (0.09)	< 5	< 5
		IS	< 5	< 5	< 5	< 5
		TIA	< 5	< 5	< 5	< 5
		MI	< 5	6 (0.06)	< 5	< 5
		HF	17 (0.29)	13 (0.13)	< 5	< 5
		HS	< 5	< 5	< 5	< 5
		MP	< 5	< 5	< 5	< 5
	91 to 180 days	VTE	< 5	12 (0.12)	< 5	< 5
		DVT	< 5	7 (0.07)	< 5	< 5
		PE	< 5	5 (0.05)	< 5	< 5

Mercadé-Besora N, *et al. Heart* 2024; 110:635–643. doi: 10.1136/heartjnl-2023-323483

Cohort	Time window	Outcome	AURUM		GOLD	
			BNT162b2	ChAdOx1	BNT162b2	ChAdOx1
		ATE	5 (0.08)	10 (0.10)	< 5	< 5
		IS	< 5	< 5	< 5	< 5
		TIA	< 5	< 5	< 5	< 5
		MI	< 5	6 (0.06)	< 5	< 5
		HF	9 (0.15)	18 (0.19)	< 5	< 5
		HS	< 5	< 5	< 5	< 5
		MP	< 5	< 5	< 5	< 5
	181 to 365 days	VTE	7 (0.12)	5 (0.05)	< 5	< 5
		DVT	< 5	< 5	< 5	< 5
		PE	5 (0.08)	< 5	< 5	< 5
		ATE	6 (0.10)	10 (0.10)	< 5	< 5
		IS	< 5	< 5	< 5	< 5
		TIA	< 5	< 5	< 5	< 5
		MI	< 5	6 (0.06)	< 5	< 5
		HF	11 (0.19)	20 (0.21)	< 5	< 5
		HS	< 5	< 5	< 5	< 5
		MP	< 5	< 5	< 5	< 5
Cohort 3			N = 54,102	N = 1,473,602	N = 36,748	N = 423,876
	0 to 30 days	VTE	< 5	23 (0.16)	< 5	< 5
		DVT	< 5	< 5	< 5	< 5
		PE	< 5	19 (0.13)	< 5	< 5
		ATE	< 5	8 (0.05)	< 5	< 5
		IS	< 5	< 5	< 5	< 5
		TIA	< 5	< 5	< 5	< 5
		MI	< 5	7 (0.05)	< 5	< 5
		HF	7 (1.29)	11 (0.07)	< 5	< 5
		HS	< 5	< 5	< 5	< 5
		MP	< 5	< 5	< 5	< 5
	31 to 90 days	VTE	< 5	< 5	< 5	< 5
		DVT	< 5	< 5	< 5	< 5
		PE	< 5	< 5	< 5	< 5
		ATE	< 5	6 (0.04)	< 5	< 5
		IS	< 5	< 5	< 5	< 5
		TIA	< 5	< 5	< 5	< 5

Cohort	Time window	Outcome	AURUM		GOLD	
			BNT162b2	ChAdOx1	BNT162b2	ChAdOx1
		MI	< 5	< 5	< 5	< 5
		HF	< 5	5 (0.03)	< 5	< 5
		HS	< 5	< 5	< 5	< 5
		MP	< 5	< 5	< 5	< 5
	91 to 180 days	VTE	< 5	8 (0.05)	< 5	< 5
		DVT	< 5	6 (0.04)	< 5	< 5
		PE	< 5	< 5	< 5	< 5
		ATE	< 5	7 (0.05)	< 5	< 5
		IS	< 5	< 5	< 5	< 5
		TIA	< 5	< 5	< 5	< 5
		MI	< 5	< 5	< 5	< 5
		HF	< 5	< 5	< 5	< 5
		HS	< 5	< 5	< 5	< 5
		MP	< 5	< 5	< 5	< 5
	181 to 365 days	VTE	< 5	9 (0.06)	< 5	< 5
		DVT	< 5	< 5	< 5	< 5
		PE	< 5	6 (0.04)	< 5	< 5
		ATE	< 5	< 5	< 5	< 5
		IS	< 5	< 5	< 5	< 5
		TIA	< 5	< 5	< 5	< 5
		MI	< 5	< 5	< 5	< 5
		HF	< 5	< 5	< 5	< 5
		HS	< 5	< 5	< 5	< 5
		MP	< 5	< 5	< 5	< 5
Cohort 4			N = 1,335,671	N = 542,670	N = 365,096	N = 147,744
	0 to 30 days	VTE	9 (0.07)	8 (0.15)	< 5	6 (0.41)
		DVT	5 (0.04)	< 5	< 5	< 5
		PE	< 5	7 (0.13)	< 5	6 (0.41)
		ATE	< 5	< 5	< 5	< 5
		IS	< 5	< 5	< 5	< 5
		TIA	< 5	< 5	< 5	< 5
		MI	< 5	< 5	< 5	< 5
		HF	< 5	< 5	< 5	< 5
		HS	< 5	< 5	< 5	< 5

Mercadé-Besora N, *et al. Heart* 2024; 110:635–643. doi: 10.1136/heartjnl-2023-323483

Cohort	Time window	Outcome	AURUM		GOLD	
			BNT162b2	ChAdOx1	BNT162b2	ChAdOx1
		MP	< 5	< 5	< 5	< 5
	31 to 90 days	VTE	< 5	8 (0.15)	< 5	< 5
		DVT	< 5	< 5	< 5	< 5
		PE	< 5	5 (0.09)	< 5	< 5
		ATE	< 5	< 5	< 5	< 5
		IS	< 5	< 5	< 5	< 5
		TIA	< 5	< 5	< 5	< 5
		MI	< 5	< 5	< 5	< 5
		HF	6 (0.04)	< 5	< 5	< 5
		HS	< 5	< 5	< 5	< 5
		MP	< 5	< 5	< 5	< 5
	91 to 180 days	VTE	8 (0.06)	< 5	< 5	< 5
		DVT	7 (0.05)	< 5	< 5	< 5
		PE	< 5	< 5	< 5	< 5
		ATE	< 5	< 5	< 5	< 5
		IS	< 5	< 5	< 5	< 5
		TIA	< 5	< 5	< 5	< 5
		MI	< 5	< 5	< 5	< 5
		HF	< 5	< 5	< 5	< 5
		HS	< 5	< 5	< 5	< 5
		MP	< 5	< 5	< 5	< 5
	181 to 365 days	VTE	< 5	< 5	< 5	< 5
		DVT	< 5	< 5	< 5	< 5
		PE	< 5	< 5	< 5	< 5
		ATE	< 5	< 5	< 5	< 5
		IS	< 5	< 5	< 5	< 5
		TIA	< 5	< 5	< 5	< 5
		MI	< 5	< 5	< 5	< 5
		HF	< 5	< 5	< 5	< 5
		HS	< 5	< 5	< 5	< 5
		MP	< 5	< 5	< 5	< 5

VTE = Venous thromboembolism (DVT + PE), DVT = Deep vein thrombosis, PE = Pulmonary embolism, ATE = Arterial thrombosis/thromboembolism (IS + TIA + MI), IS = Ischemic stroke, TIA = Transient ischemic attack, MI = Myocardial infarction, HF = Heart failure, HS = Hemorrhagic stroke, MP = Myocarditis or Pericarditis

Mercadé-Besora N, *et al. Heart* 2024; 110:635–643. doi: 10.1136/heartjnl-2023-323483

Table S36: Number of records (and risk per 10,000 individuals) for post COVID-19 cardiac and thromboembolic complications, across cohorts and databases, stratified by vaccine (BNT162b2 - ChAdOx1). Only first outcome after COVID-19 captured.

Cohort	Time window	Outcome	AURUM		GOLD	
			BNT162b2	ChAdOx1	BNT162b2	ChAdOx1
**Cohort 1**			**N = 332,790**	**N = 219,804**	**N = 32,755**	**N = 82,406**
	**0 to 30 days**	**VTE**	76 (2.28)	41 (1.87)	< 5	< 5
		**DVT**	15 (0.45)	12 (0.55)	< 5	< 5
		**PE**	64 (1.92)	31 (1.41)	< 5	< 5
		**ATE**	45 (1.35)	25 (1.14)	< 5	6 (0.73)
		**IS**	5 (0.15)	< 5	< 5	< 5
		**TIA**	11 (0.33)	7 (0.32)	< 5	< 5
		**MI**	29 (0.87)	17 (0.77)	< 5	< 5
		**HF**	126 (3.79)	72 (3.28)	< 5	5 (0.61)
		**HS**	< 5	< 5	< 5	< 5
		**MP**	< 5	< 5	< 5	< 5
	**31 to 90 days**	**VTE**	25 (0.75)	14 (0.64)	< 5	< 5
		**DVT**	7 (0.21)	8 (0.36)	< 5	< 5
		**PE**	18 (0.54)	7 (0.32)	< 5	< 5
		**ATE**	23 (0.69)	20 (0.91)	< 5	< 5
		**IS**	< 5	< 5	< 5	< 5
		**TIA**	10 (0.30)	9 (0.41)	< 5	< 5
		**MI**	11 (0.33)	9 (0.41)	< 5	< 5
		**HF**	70 (2.10)	39 (1.77)	< 5	5 (0.61)
		**HS**	< 5	< 5	< 5	< 5
		**MP**	< 5	< 5	< 5	< 5
	**91 to 180 days**	**VTE**	< 5	15 (0.68)	< 5	< 5
		**DVT**	< 5	5 (0.23)	< 5	< 5
		**PE**	< 5	10 (0.45)	< 5	< 5
		**ATE**	18 (0.54)	10 (0.45)	< 5	6 (0.73)
		**IS**	6 (0.18)	< 5	< 5	< 5
		**TIA**	6 (0.18)	6 (0.27)	< 5	< 5
		**MI**	6 (0.18)	< 5	< 5	5 (0.61)
		**HF**	54 (1.62)	35 (1.59)	< 5	< 5
		**HS**	< 5	< 5	< 5	< 5
		**MP**	< 5	< 5	< 5	< 5

Cohort	Time window	Outcome	AURUM		GOLD	
			BNT162b2	ChAdOx1	BNT162b2	ChAdOx1
	**181 to 365 days**	**VTE**	10 (0.30)	< 5	< 5	< 5
		**DVT**	6 (0.18)	< 5	< 5	< 5
		**PE**	< 5	< 5	< 5	< 5
		**ATE**	14 (0.42)	9 (0.41)	< 5	< 5
		**IS**	< 5	< 5	< 5	< 5
		**TIA**	< 5	< 5	< 5	< 5
		**MI**	7 (0.21)	< 5	< 5	< 5
		**HF**	35 (1.05)	18 (0.82)	< 5	5 (0.61)
		**HS**	< 5	< 5	< 5	< 5
		**MP**	< 5	< 5	< 5	< 5
**Cohort 2**			**N = 594,262**	**N = 969,262**	**N = 180,670**	**N = 302,999**
	**0 to 30 days**	**VTE**	62 (1.04)	158 (1.63)	5 (0.28)	19 (0.63)
		**DVT**	18 (0.30)	47 (0.48)	< 5	< 5
		**PE**	45 (0.76)	120 (1.24)	< 5	15 (0.50)
		**ATE**	29 (0.49)	75 (0.77)	< 5	< 5
		**IS**	< 5	12 (0.12)	< 5	< 5
		**TIA**	11 (0.19)	13 (0.13)	< 5	< 5
		**MI**	16 (0.27)	52 (0.54)	< 5	< 5
		**HF**	43 (0.72)	103 (1.06)	< 5	11 (0.36)
		**HS**	< 5	< 5	< 5	< 5
		**MP**	< 5	6 (0.06)	< 5	< 5
	**31 to 90 days**	**VTE**	21 (0.35)	54 (0.56)	< 5	9 (0.30)
		**DVT**	7 (0.12)	25 (0.26)	< 5	5 (0.17)
		**PE**	14 (0.24)	31 (0.32)	< 5	< 5
		**ATE**	36 (0.61)	56 (0.58)	< 5	5 (0.17)
		**IS**	5 (0.08)	6 (0.06)	< 5	< 5
		**TIA**	9 (0.15)	24 (0.25)	< 5	< 5
		**MI**	22 (0.37)	28 (0.29)	< 5	< 5
		**HF**	34 (0.57)	65 (0.67)	< 5	7 (0.23)
		**HS**	< 5	< 5	< 5	< 5
		**MP**	< 5	< 5	< 5	< 5
	**91 to 180 days**	**VTE**	9 (0.15)	28 (0.29)	< 5	< 5
		**DVT**	6 (0.10)	15 (0.15)	< 5	< 5
		**PE**	< 5	15 (0.15)	< 5	< 5

Cohort	Time window	Outcome	AURUM		GOLD	
			BNT162b2	ChAdOx1	BNT162b2	ChAdOx1
		ATE	15 (0.25)	28 (0.29)	< 5	< 5
		IS	< 5	< 5	< 5	< 5
		TIA	5 (0.08)	13 (0.13)	< 5	< 5
		MI	10 (0.17)	15 (0.15)	< 5	< 5
		HF	24 (0.40)	41 (0.42)	< 5	< 5
		HS	< 5	< 5	< 5	< 5
		MP	< 5	< 5	< 5	< 5
	181 to 365 days	VTE	8 (0.13)	< 5	< 5	< 5
		DVT	< 5	< 5	< 5	< 5
		PE	7 (0.12)	< 5	< 5	< 5
		ATE	7 (0.12)	10 (0.10)	< 5	< 5
		IS	< 5	< 5	< 5	< 5
		TIA	< 5	< 5	< 5	< 5
		MI	< 5	6 (0.06)	< 5	< 5
		HF	12 (0.20)	22 (0.23)	< 5	< 5
		HS	< 5	< 5	< 5	< 5
		MP	< 5	< 5	< 5	< 5
Cohort 3			N = 54,102	N = 1,473,602	N = 36,748	N = 423,876
	0 to 30 days	VTE	< 5	139 (0.94)	< 5	16 (0.38)
		DVT	< 5	41 (0.28)	< 5	< 5
		PE	< 5	101 (0.69)	< 5	12 (0.28)
		ATE	< 5	47 (0.32)	< 5	12 (0.28)
		IS	< 5	5 (0.03)	< 5	< 5
		TIA	< 5	16 (0.11)	< 5	< 5
		MI	< 5	26 (0.18)	< 5	9 (0.21)
		HF	10 (1.85)	28 (0.19)	< 5	< 5
		HS	< 5	< 5	< 5	< 5
		MP	< 5	6 (0.04)	< 5	< 5
	31 to 90 days	VTE	< 5	44 (0.30)	< 5	6 (0.14)
		DVT	< 5	26 (0.18)	< 5	< 5
		PE	< 5	18 (0.12)	< 5	< 5
		ATE	< 5	31 (0.21)	< 5	7 (0.17)
		IS	< 5	< 5	< 5	< 5
		TIA	< 5	13 (0.09)	< 5	< 5

Cohort	Time window	Outcome	AURUM		GOLD	
			BNT162b2	ChAdOx1	BNT162b2	ChAdOx1
		**MI**	< 5	16 (0.11)	< 5	< 5
		**HF**	< 5	23 (0.16)	< 5	< 5
		**HS**	< 5	< 5	< 5	< 5
		**MP**	< 5	7 (0.05)	< 5	< 5
	**91 to 180 days**	**VTE**	< 5	25 (0.17)	< 5	< 5
		**DVT**	< 5	15 (0.10)	< 5	< 5
		**PE**	< 5	13 (0.09)	< 5	< 5
		**ATE**	< 5	26 (0.18)	< 5	< 5
		**IS**	< 5	< 5	< 5	< 5
		**TIA**	< 5	8 (0.05)	< 5	< 5
		**MI**	< 5	17 (0.12)	< 5	< 5
		**HF**	< 5	10 (0.07)	< 5	< 5
		**HS**	< 5	< 5	< 5	< 5
		**MP**	< 5	< 5	< 5	< 5
	**181 to 365 days**	**VTE**	< 5	9 (0.06)	< 5	< 5
		**DVT**	< 5	< 5	< 5	< 5
		**PE**	< 5	6 (0.04)	< 5	< 5
		**ATE**	< 5	< 5	< 5	< 5
		**IS**	< 5	< 5	< 5	< 5
		**TIA**	< 5	< 5	< 5	< 5
		**MI**	< 5	< 5	< 5	< 5
		**HF**	< 5	< 5	< 5	< 5
		**HS**	< 5	< 5	< 5	< 5
		**MP**	< 5	< 5	< 5	< 5
**Cohort 4**			**N = 1,335,671**	**N = 542,670**	**N = 365,096**	**N = 147,744**
	**0 to 30 days**	**VTE**	20 (0.15)	27 (0.50)	< 5	8 (0.54)
		**DVT**	9 (0.07)	7 (0.13)	< 5	< 5
		**PE**	11 (0.08)	21 (0.39)	< 5	6 (0.41)
		**ATE**	< 5	5 (0.09)	< 5	< 5
		**IS**	< 5	< 5	< 5	< 5
		**TIA**	< 5	< 5	< 5	< 5
		**MI**	< 5	< 5	< 5	< 5
		**HF**	< 5	< 5	< 5	< 5
		**HS**	< 5	< 5	< 5	< 5

Mercadé-Besora N, *et al. Heart* 2024; 110:635–643. doi: 10.1136/heartjnl-2023-323483

Cohort	Time window	Outcome	AURUM		GOLD	
			BNT162b2	ChAdOx1	BNT162b2	ChAdOx1
		MP	< 5	< 5	< 5	< 5
	31 to 90 days	VTE	7 (0.05)	14 (0.26)	< 5	< 5
		DVT	< 5	8 (0.15)	< 5	< 5
		PE	< 5	6 (0.11)	< 5	< 5
		ATE	< 5	< 5	< 5	< 5
		IS	< 5	< 5	< 5	< 5
		TIA	< 5	< 5	< 5	< 5
		MI	< 5	< 5	< 5	< 5
		HF	6 (0.04)	< 5	< 5	< 5
		HS	< 5	< 5	< 5	< 5
		MP	< 5	< 5	< 5	< 5
	91 to 180 days	VTE	9 (0.07)	< 5	< 5	< 5
		DVT	7 (0.05)	< 5	< 5	< 5
		PE	< 5	< 5	< 5	< 5
		ATE	< 5	< 5	< 5	< 5
		IS	< 5	< 5	< 5	< 5
		TIA	< 5	< 5	< 5	< 5
		MI	< 5	< 5	< 5	< 5
		HF	< 5	< 5	< 5	< 5
		HS	< 5	< 5	< 5	< 5
		MP	< 5	< 5	< 5	< 5
	181 to 365 days	VTE	< 5	< 5	< 5	< 5
		DVT	< 5	< 5	< 5	< 5
		PE	< 5	< 5	< 5	< 5
		ATE	< 5	< 5	< 5	< 5
		IS	< 5	< 5	< 5	< 5
		TIA	< 5	< 5	< 5	< 5
		MI	< 5	< 5	< 5	< 5
		HF	< 5	< 5	< 5	< 5
		HS	< 5	< 5	< 5	< 5
		MP	< 5	< 5	< 5	< 5

VTE = Venous thromboembolism (DVT + PE), DVT = Deep vein thrombosis, PE = Pulmonary embolism, ATE = Arterial thrombosis/thromboembolism (IS + TIA + MI), IS = Ischemic stroke, TIA = Transient ischemic attack, MI = Myocardial infarction, HF = Heart failure, HS = Hemorrhagic stroke, MP = Myocarditis or Pericarditis

Mercadé-Besora N, *et al. Heart* 2024; 110:635–643. doi: 10.1136/heartjnl-2023-323483

Figure S6: Forest plots for vaccine effect (any COVID-19 vaccine), meta-analysis across cohorts and databases. Follow-up ends at first vaccine dose after index date. Dashed line represents a level of hetereogeneity I2 > 0.4.

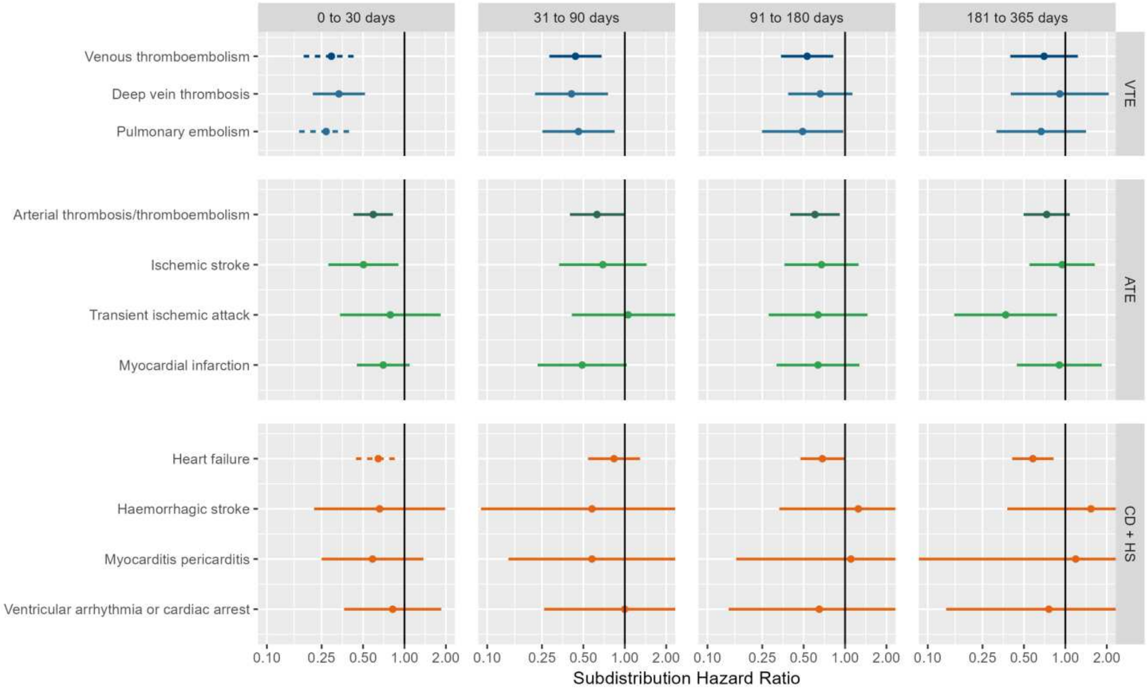

VTE = Venous thromboembolism, ATE = Arterial thrombosis/thromboembolism, CD + HS = Cardiac diseases and Hemorrhagic stroke

BMJ Publishing Group Limited (BMJ) disclaims all liability and responsibility arising from any reliance placed on this supplemental material which has been supplied by the author(s)

Figure S7: Forest plots for vaccine effect (any COVID-19 vaccine), meta-analysis across cohorts and databases. Only first outcome after COVID-19 captured. Dashed line represents a level of hetereogeneity I2 > 0.4.

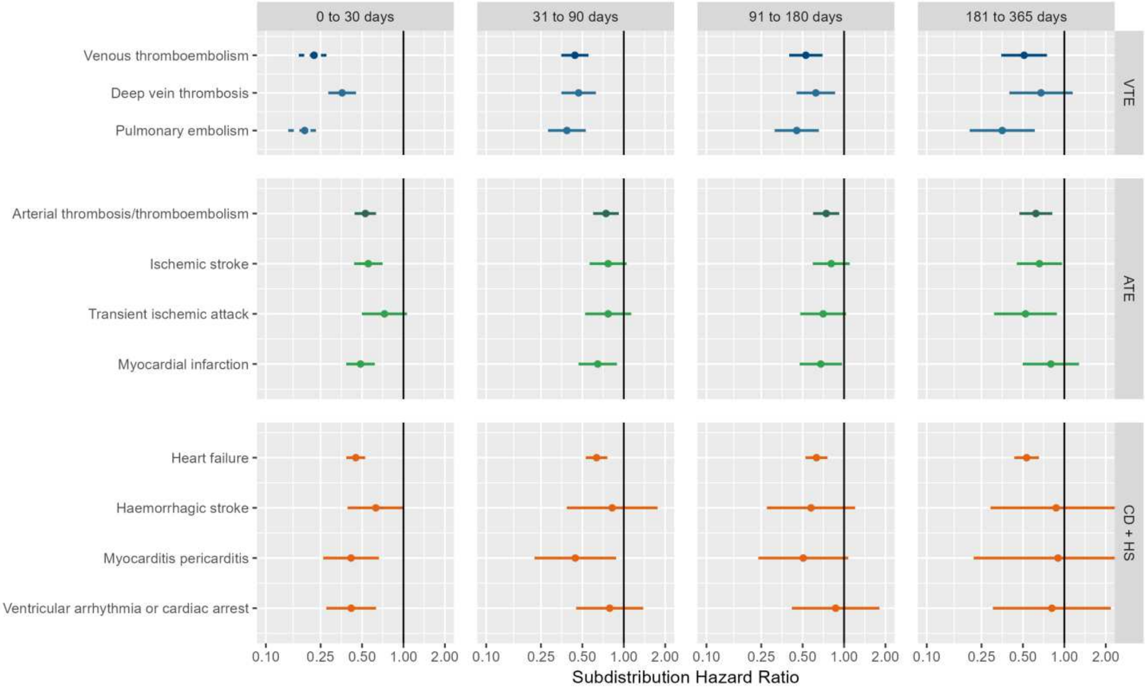

VTE = Venous thromboembolism, ATE = Arterial thrombosis/thromboembolism, CD + HS = Cardiac diseases and Hemorrhagic stroke

Mercadé-Besora N, *et al. Heart* 2024; 110:635–643. doi: 10.1136/heartjnl-2023-323483

Figure S8: Forest plots for vaccine effect (ChAdOx1 vaccine), meta-analysis across cohorts and databases. Dashed line represents a level of hetereogeneity I2 > 0.4.

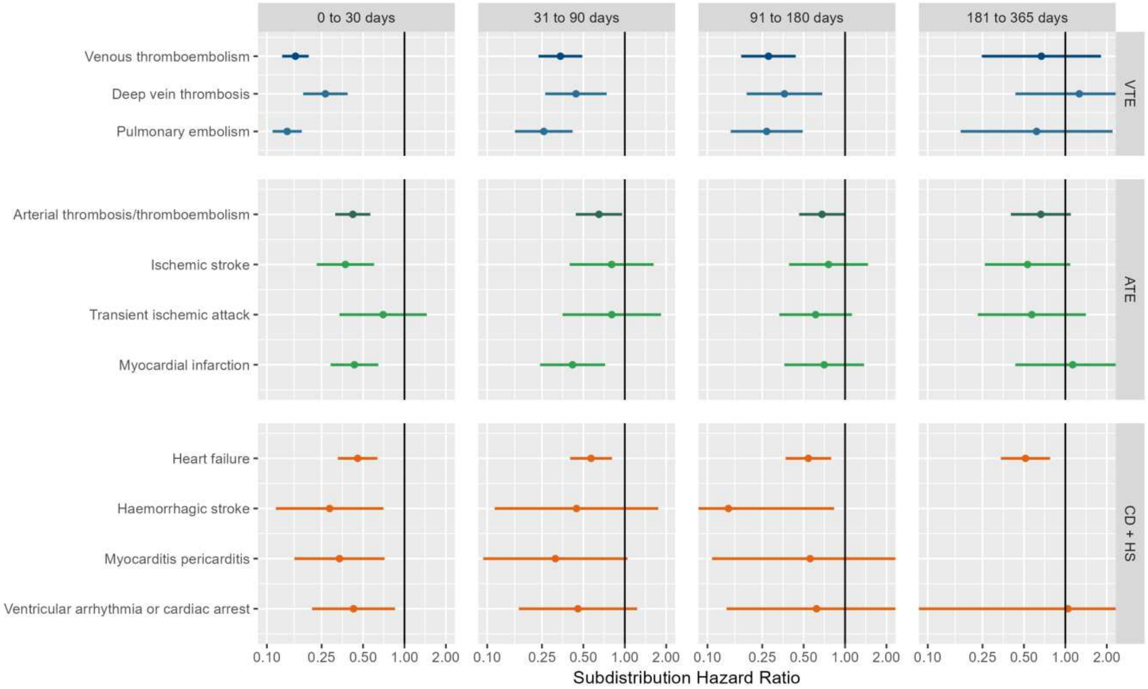

VTE = Venous thromboembolism, ATE = Arterial thrombosis/thromboembolism, CD + HS = Cardiac diseases and Hemorrhagic stroke

Figure S9: Forest plots for vaccine effect (ChAdOx1 vaccine), meta-analysis across cohorts and databases. Follow-up ends at first vaccine dose after index date. Dashed line represents a level of hetereogeneity I2 > 0.4.

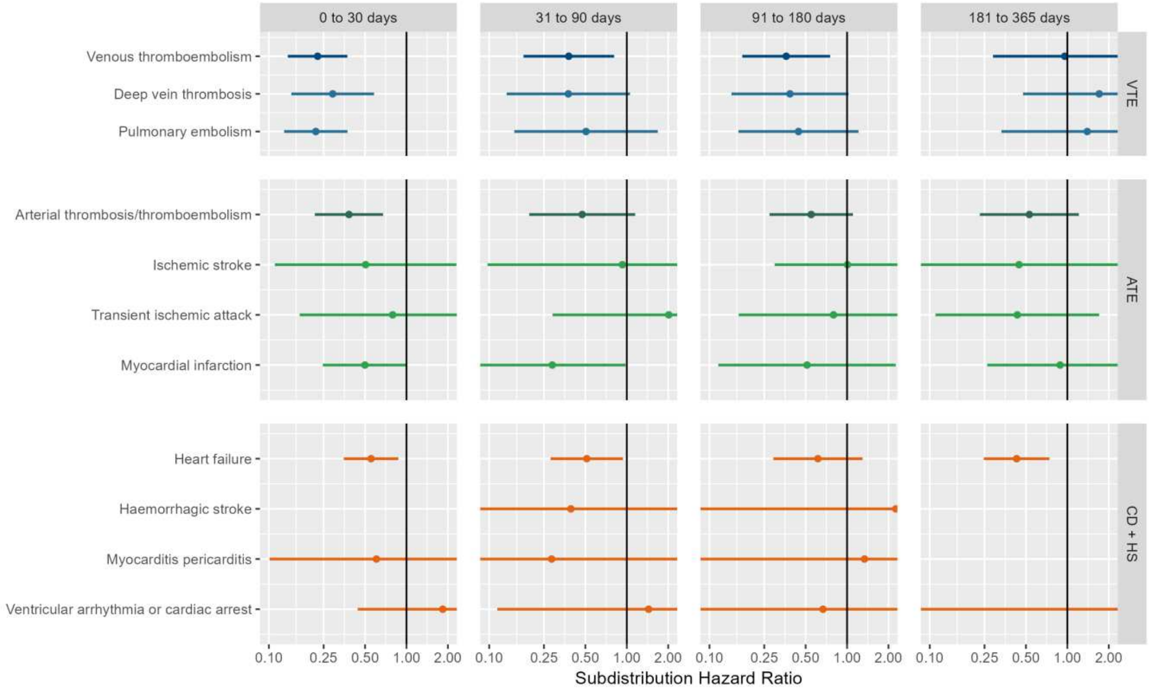

VTE = Venous thromboembolism, ATE = Arterial thrombosis/thromboembolism, CD + HS = Cardiac diseases and Hemorrhagic stroke

Figure S10: Forest plots for vaccine effect (ChAdOx1 vaccine), meta-analysis across cohorts and databases. Only first outcome after COVID-19 captured. Dashed line represents a level of hetereogeneity I2 > 0.4.

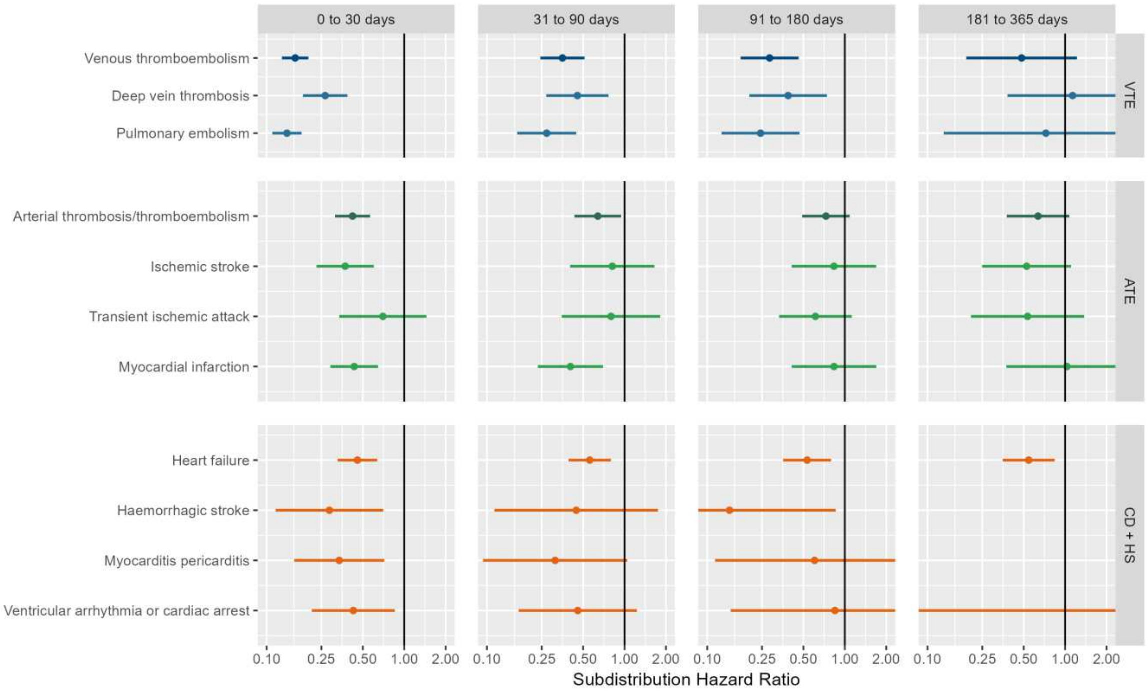

VTE = Venous thromboembolism, ATE = Arterial thrombosis/thromboembolism, CD + HS = Cardiac diseases and Hemorrhagic stroke

Figure S11: Forest plots for vaccine effect (BNT162b2 vaccine), meta-analysis across cohorts and databases. Dashed line represents a level of hetereogeneity I2 > 0.4.

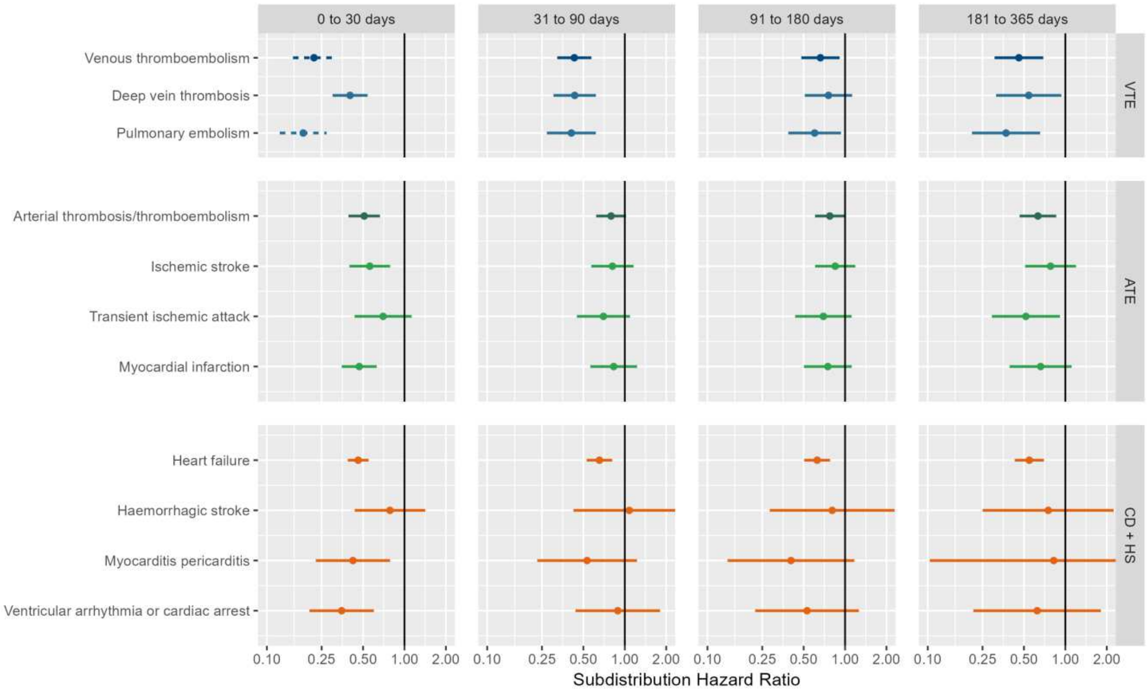

VTE = Venous thromboembolism, ATE = Arterial thrombosis/thromboembolism, CD + HS = Cardiac diseases and Hemorrhagic stroke

Mercadé-Besora N, *et al. Heart* 2024; 110:635–643. doi: 10.1136/heartjnl-2023-323483

Figure S12: Forest plots for vaccine effect (BNT162b2 vaccine), meta-analysis across cohorts and databases. Follow-up ends at first vaccine dose after index date. Dashed line represents a level of hetereogeneity I2 > 0.4.

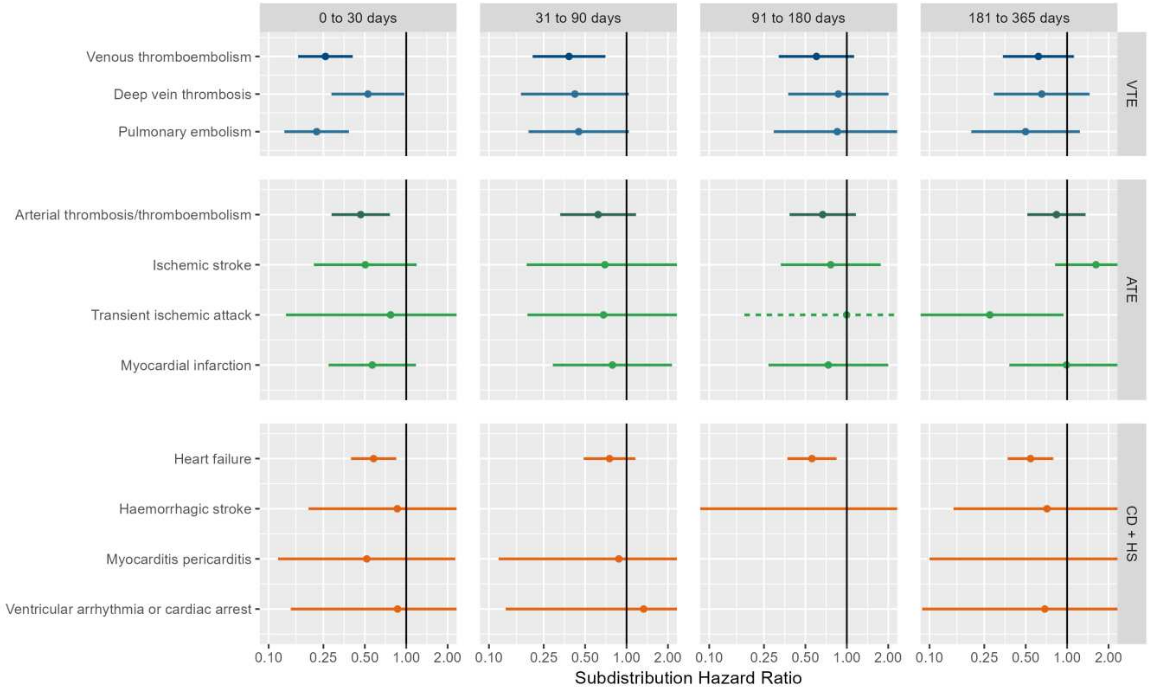

VTE = Venous thromboembolism, ATE = Arterial thrombosis/thromboembolism, CD + HS = Cardiac diseases and Hemorrhagic stroke

Figure S13: Forest plots for vaccine effect (BNT162b2 vaccine), meta-analysis across cohorts and databases. Only first outcome after COVID-19 captured. Dashed line represents a level of hetereogeneity I2 > 0.4.

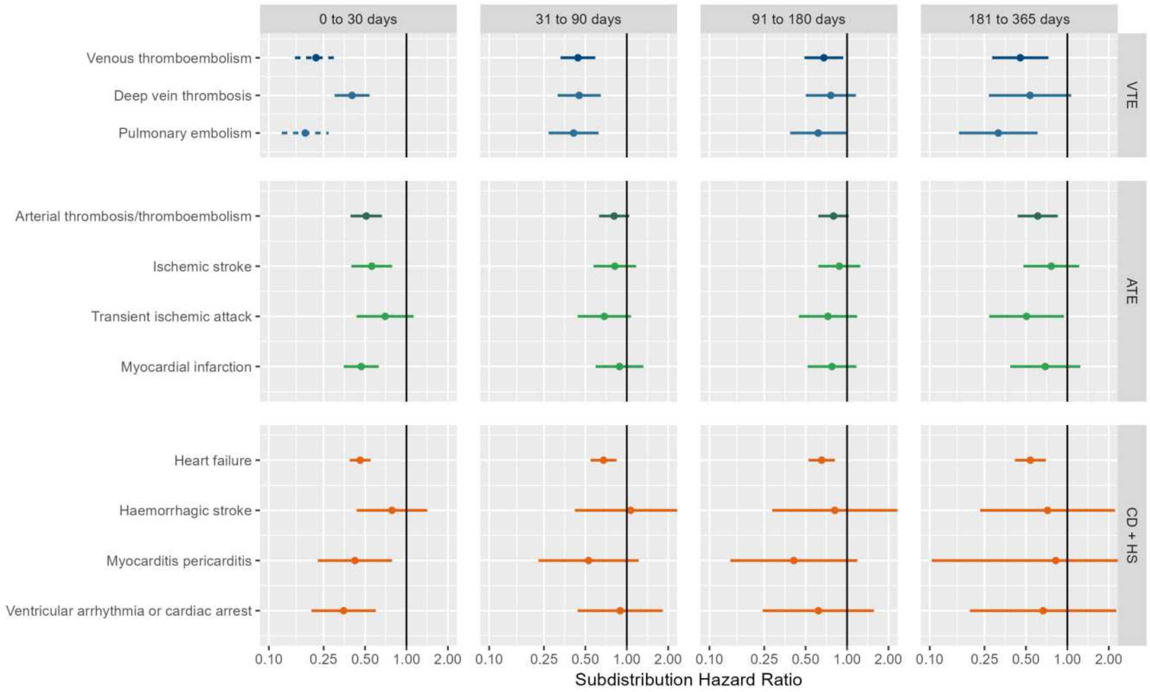

VTE = Venous thromboembolism, ATE = Arterial thrombosis/thromboembolism, CD + HS = Cardiac diseases and Hemorrhagic stroke

Figure S14: Forest plots for vaccine effect (any COVID-19 vaccine) on preventing venous thromboembolism complications, for each cohort and database (meta-analysis estimates across cohorts in the last panel). Dashed line represents a level of hetereogeneity I2 > 0.4.

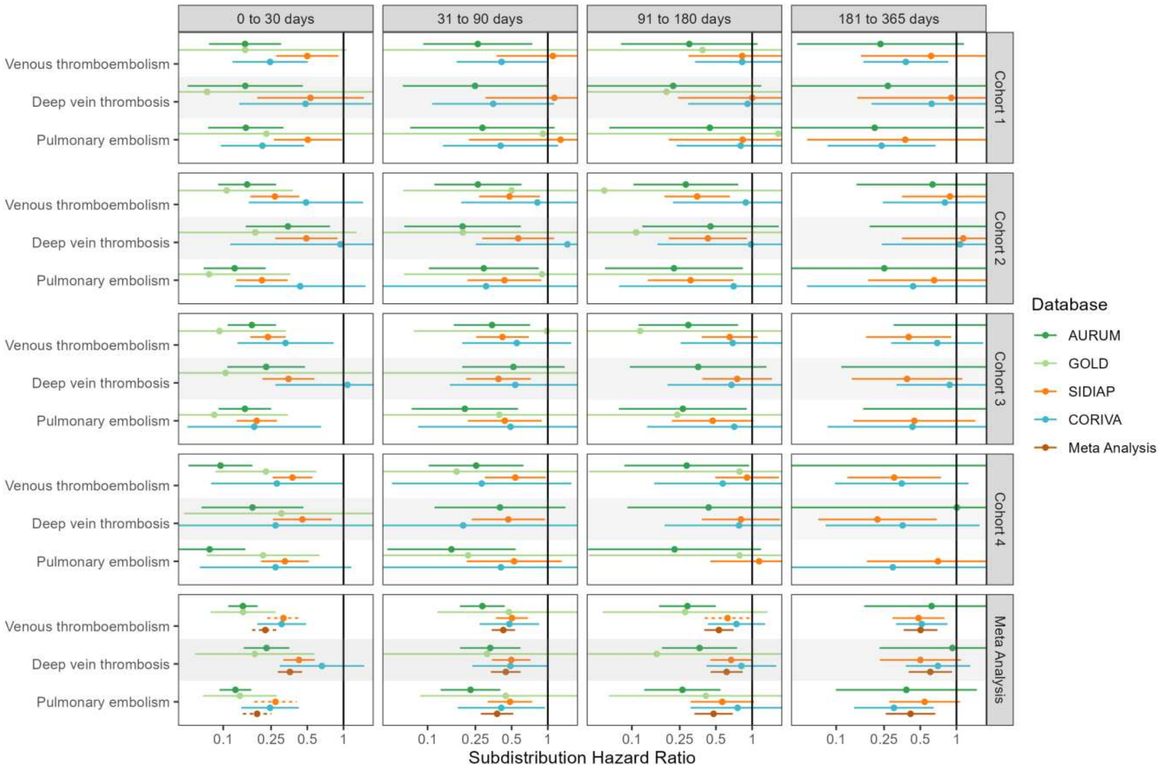

Mercadé-Besora N, *et al. Heart* 2024; 110:635–643. doi: 10.1136/heartjnl-2023-323483

Figure S15: Forest plots for vaccine effect (any COVID-19 vaccine) on preventing venous thromboembolism complications, for each cohort and database (meta-analysis estimates across cohorts in the last panel).Follow-up ends at first vaccine dose after index date. Dashed line represents a level of hetereogeneity I2 > 0.4.

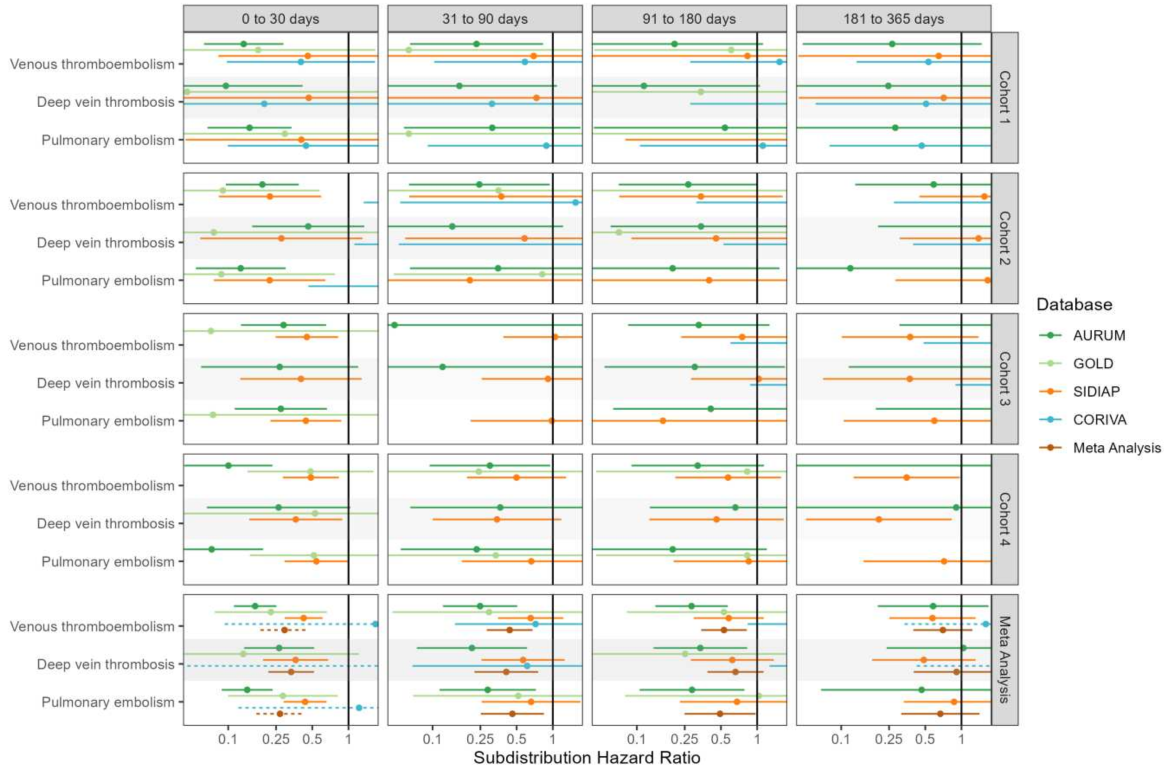

Figure S16: Forest plots for vaccine effect (any COVID-19 vaccine) on preventing venous thromboembolism complications, for each cohort and database (meta-analysis estimates across cohorts in the last panel).Only first outcome after COVID-19 captured. Dashed line represents a level of hetereogeneity I2 > 0.4.

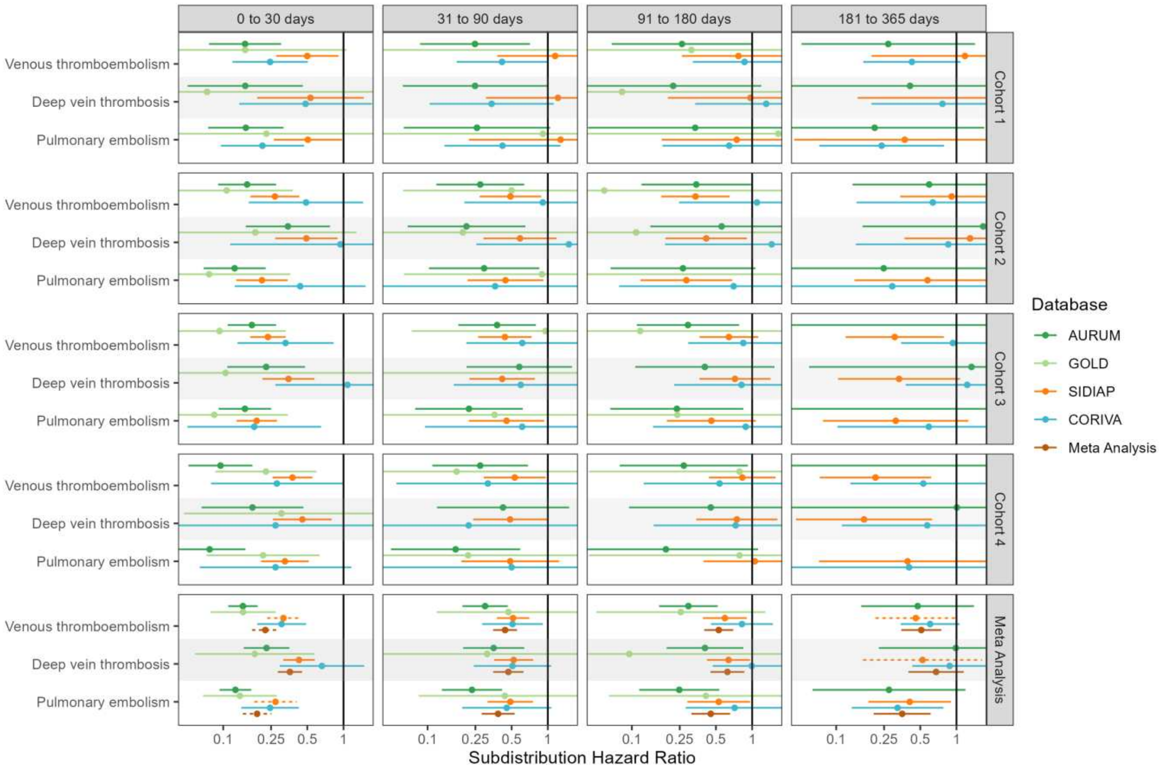

Mercadé-Besora N, *et al. Heart* 2024; 110:635–643. doi: 10.1136/heartjnl-2023-323483

Figure S17: Forest plots for vaccine effect (ChAdOx1 vaccine) on preventing venous thromboembolism complications, for each cohort and database (meta-analysis estimates across cohorts in the last panel). Dashed line represents a level of hetereogeneity I2 > 0.4.

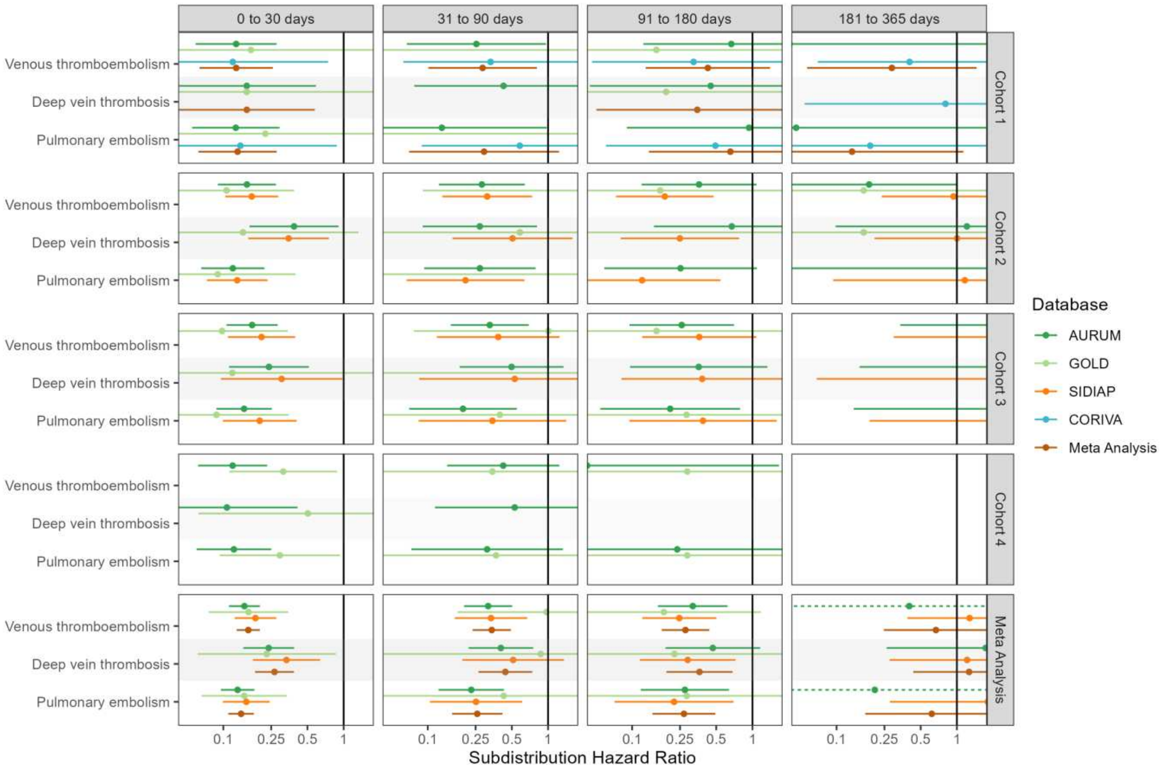

Figure S18: Forest plots for vaccine effect (ChAdOx1 vaccine) on preventing venous thromboembolism complications, for each cohort and database (meta-analysis estimates across cohorts in the last panel).Follow-up ends at first vaccine dose after index date. Dashed line represents a level of hetereogeneity I2 > 0.4.

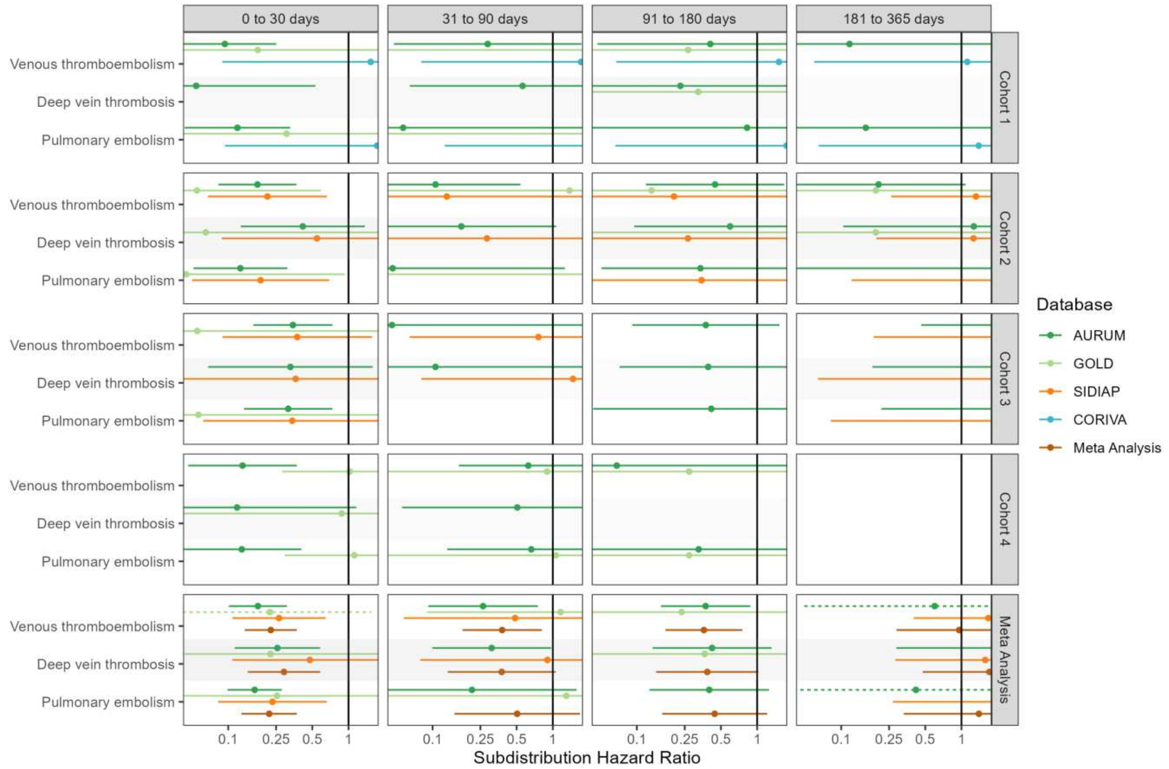

Figure S19: Forest plots for vaccine effect (ChAdOx1 vaccine) on preventing venous thromboembolism complications, for each cohort and database (meta-analysis estimates across cohorts in the last panel).Only first outcome after COVID-19 captured. Dashed line represents a level of hetereogeneity I2 > 0.4.

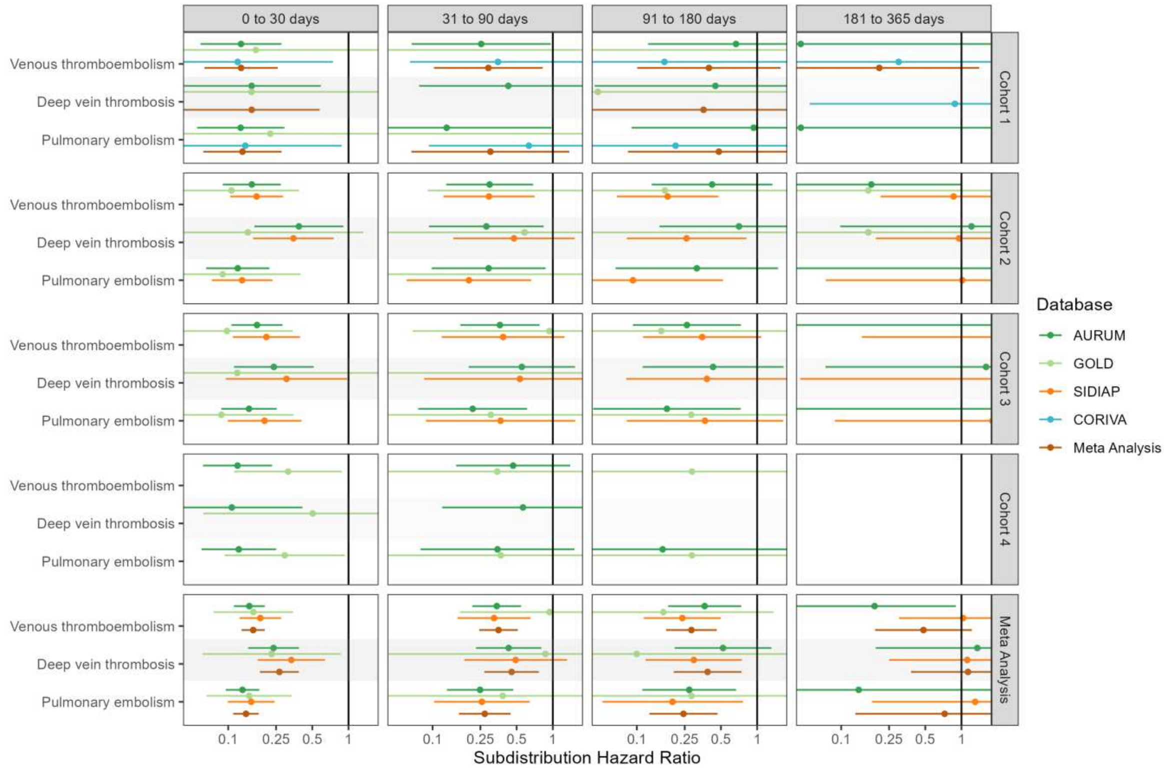

Mercadé-Besora N, *et al. Heart* 2024; 110:635–643. doi: 10.1136/heartjnl-2023-323483

Figure S20: Forest plots for vaccine effect (BNT162b2 vaccine) on preventing venous thromboembolism complications, for each cohort and database (meta-analysis estimates across cohorts in the last panel). Dashed line represents a level of hetereogeneity I2 > 0.4.

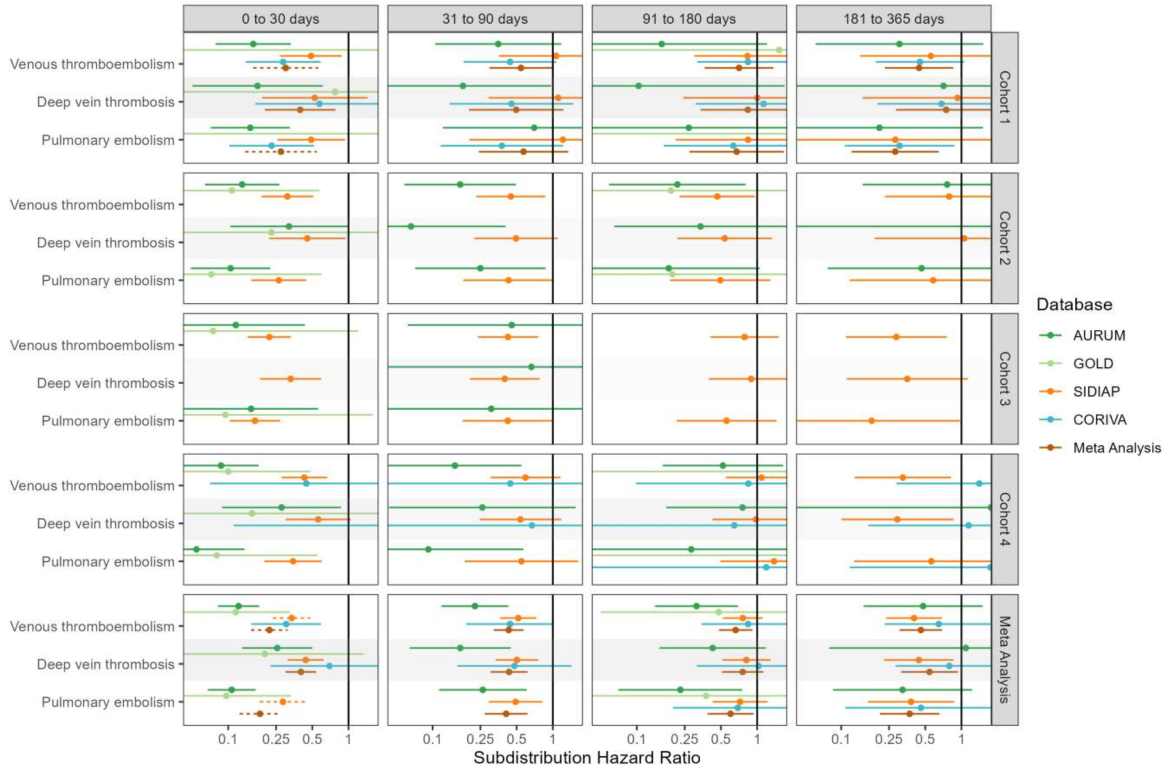

Figure S21: Forest plots for vaccine effect (BNT162b2 vaccine) on preventing venous thromboembolism complications, for each cohort and database (meta-analysis estimates across cohorts in the last panel).Follow-up ends at first vaccine dose after index date. Dashed line represents a level of hetereogeneity I2 > 0.4.

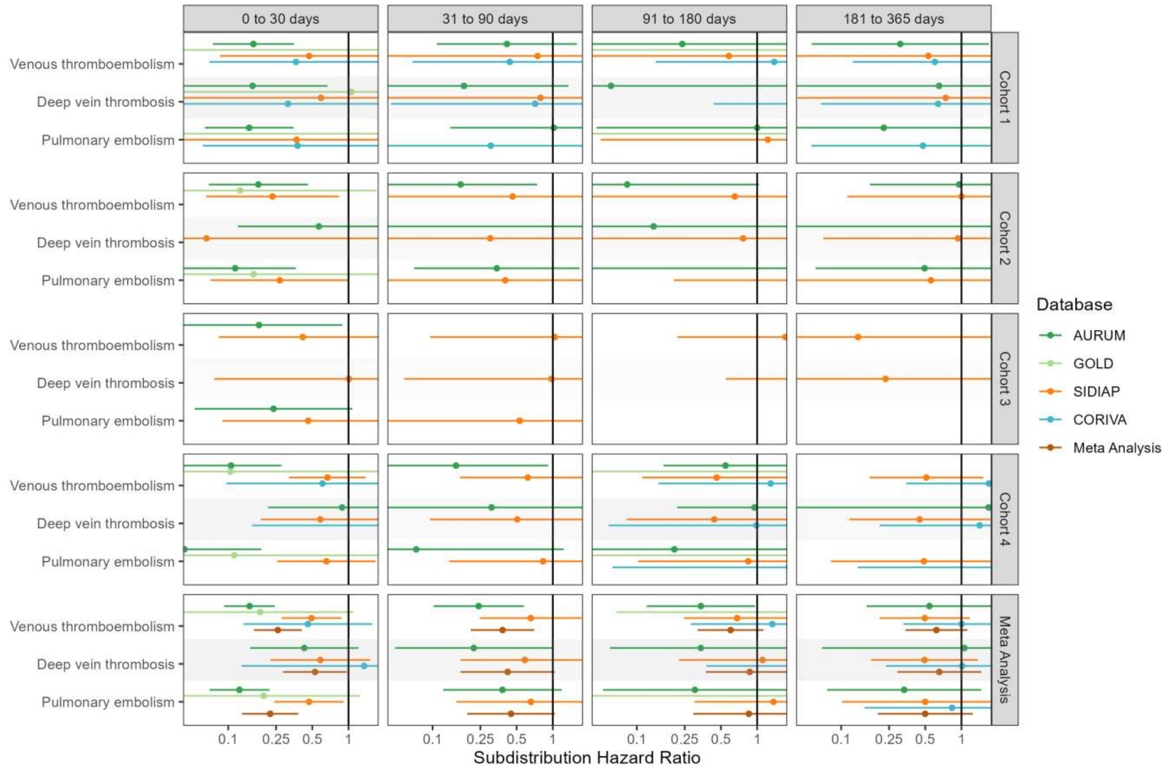

Figure S22: Forest plots for vaccine effect (BNT162b2 vaccine) on preventing venous thromboembolism complications, for each cohort and database (meta-analysis estimates across cohorts in the last panel).Only first outcome after COVID-19 captured. Dashed line represents a level of hetereogeneity I2 > 0.4.

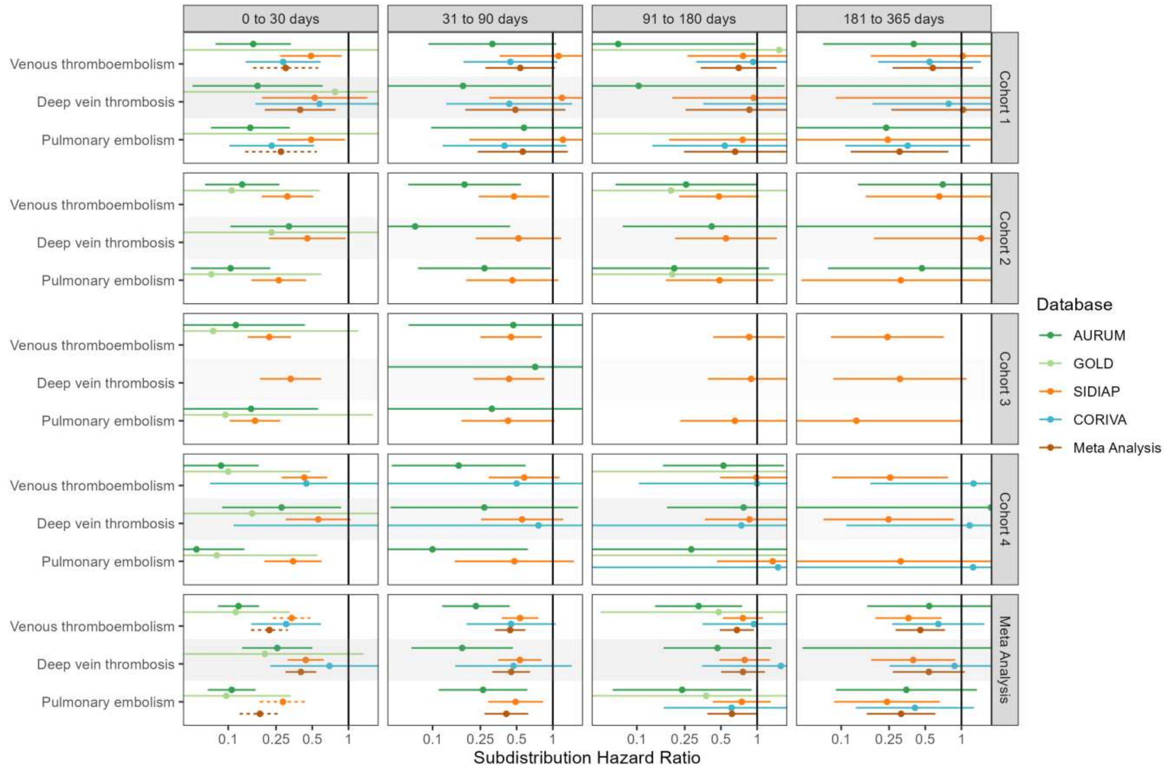

Mercadé-Besora N, *et al. Heart* 2024; 110:635–643. doi: 10.1136/heartjnl-2023-323483

Figure S23: Forest plots for vaccine effect (any COVID-19 vaccine) on preventing arterial thrombosis/thromboembolism complications, for each cohort and database (meta-analysis estimates across cohorts in the last panel). Dashed line represents a level of hetereogeneity I2 > 0.4.

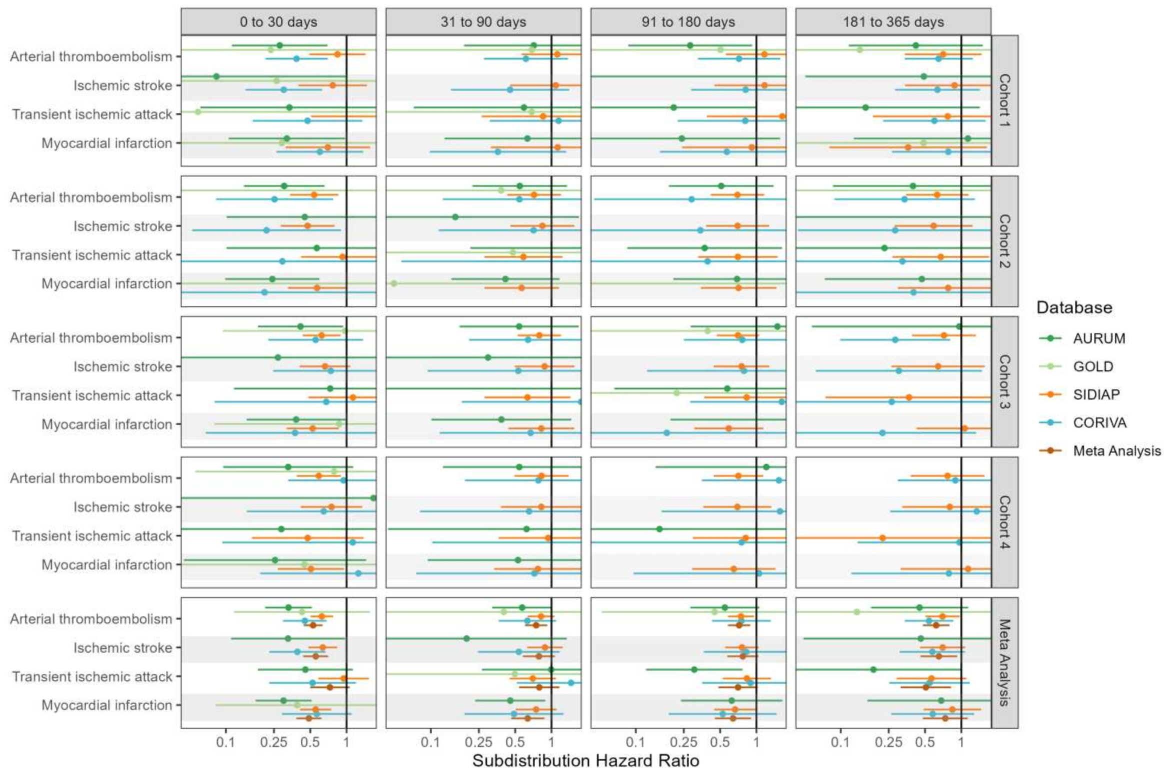

133

Figure S24: Forest plots for vaccine effect (any COVID-19 vaccine) on preventing arterial thrombosis/thromboembolism complications, for each cohort and database (meta-analysis estimates across cohorts in the last panel).Follow-up ends at first vaccine dose after index date. Dashed line represents a level of hetereogeneity I2 > 0.4.

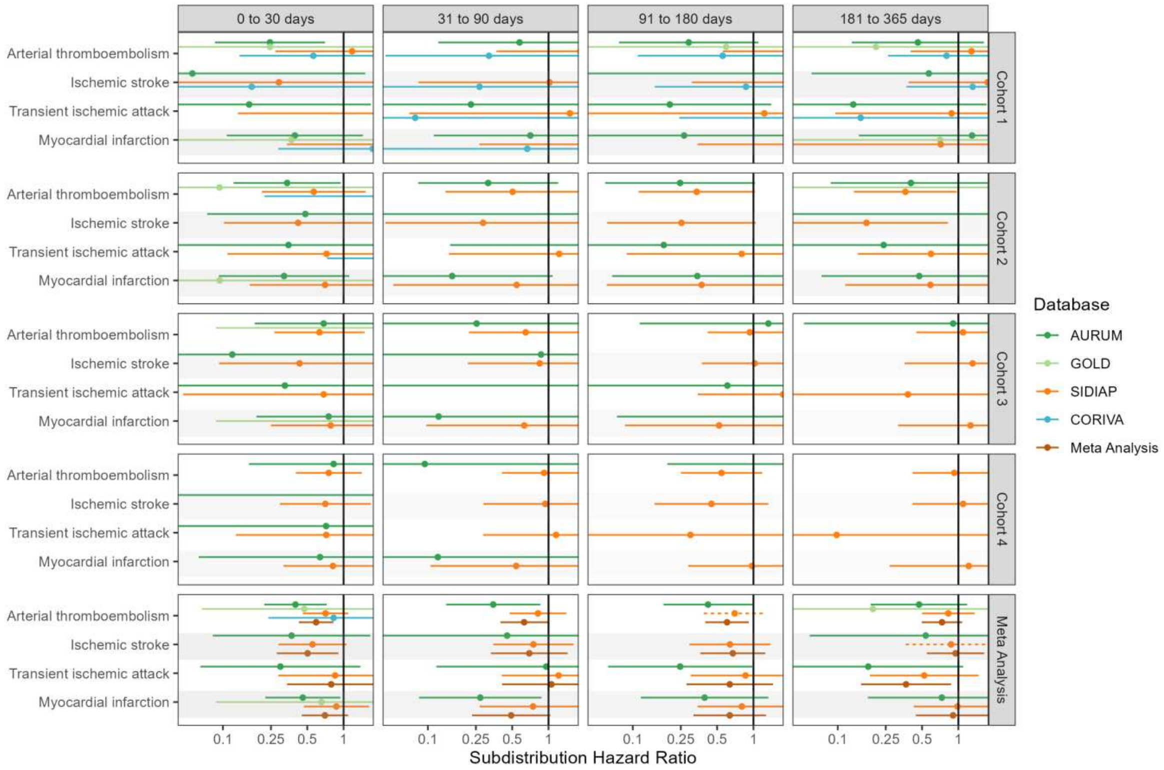

Mercadé-Besora N, *et al. Heart* 2024; 110:635–643. doi: 10.1136/heartjnl-2023-323483

Figure S25: Forest plots for vaccine effect (any COVID-19 vaccine) on preventing arterial thrombosis/thromboembolism complications, for each cohort and database (meta-analysis estimates across cohorts in the last panel).Only first outcome after COVID-19 captured. Dashed line represents a level of hetereogeneity I2 > 0.4.

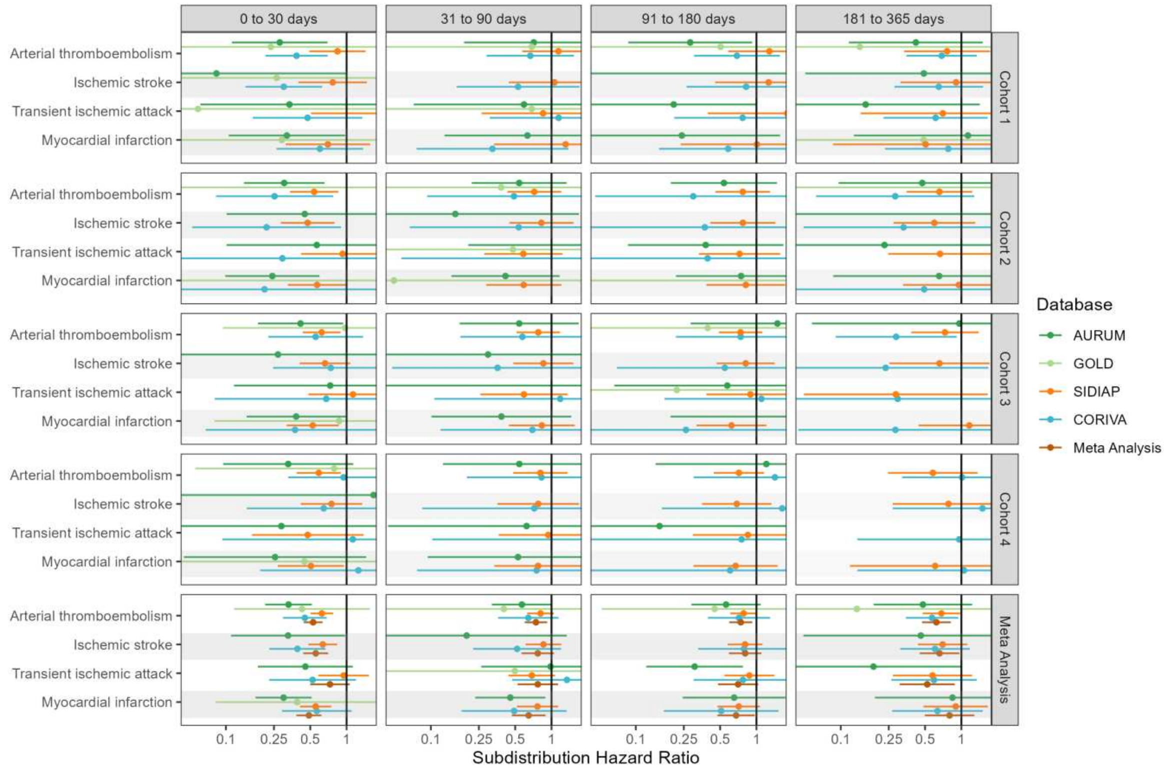

Figure S26: Forest plots for vaccine effect (ChAdOx1 vaccine) on preventing arterial thrombosis/thromboembolism complications, for each cohort and database (meta-analysis estimates across cohorts in the last panel). Dashed line represents a level of hetereogeneity I2 > 0.4.

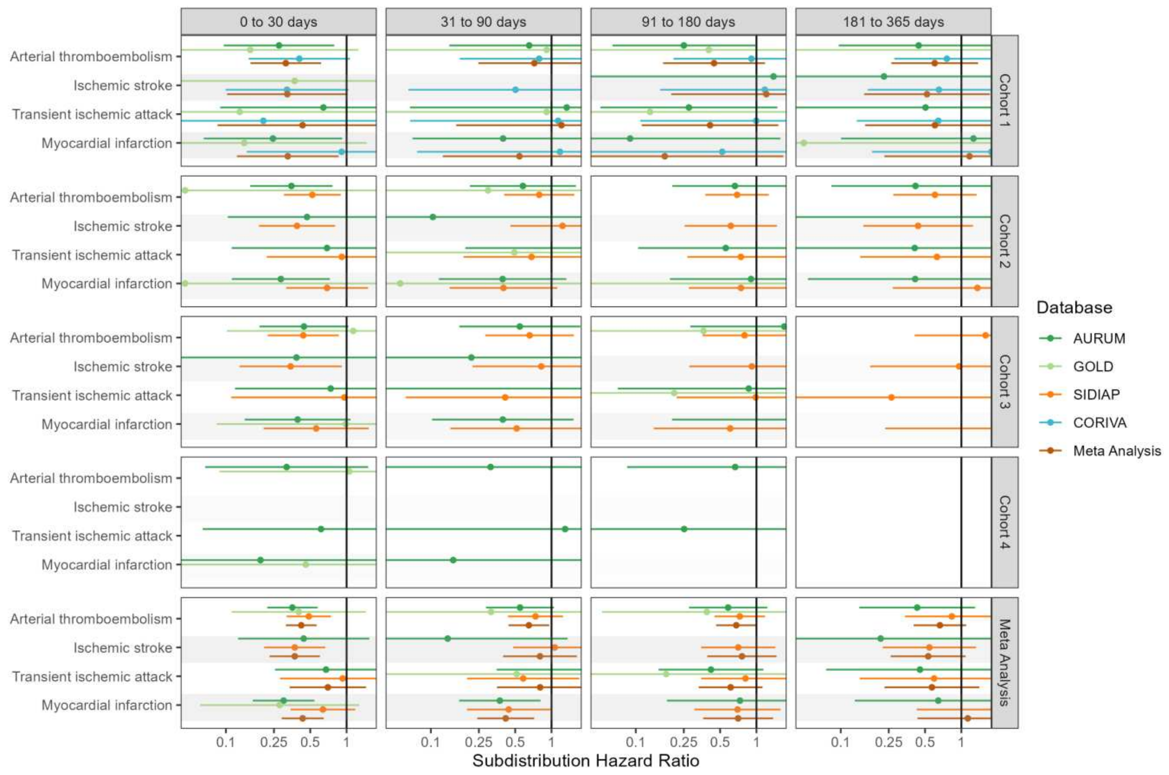

136

Figure S27: Forest plots for vaccine effect (ChAdOx1 vaccine) on preventing arterial thrombosis/thromboembolism complications, for each cohort and database (meta-analysis estimates across cohorts in the last panel).Follow-up ends at first vaccine dose after index date. Dashed line represents a level of hetereogeneity I2 > 0.4.

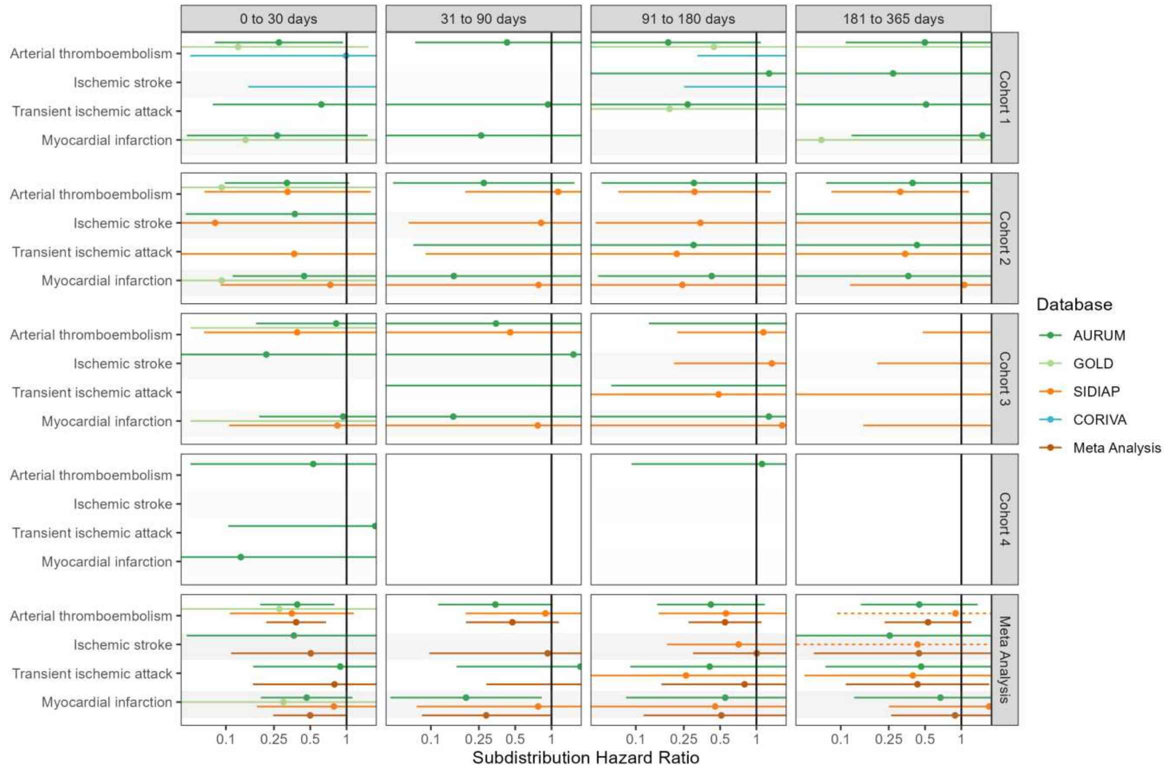

Mercadé-Besora N, *et al. Heart* 2024; 110:635–643. doi: 10.1136/heartjnl-2023-323483

Figure S28: Forest plots for vaccine effect (ChAdOx1 vaccine) on preventing arterial thrombosis/thromboembolism complications, for each cohort and database (meta-analysis estimates across cohorts in the last panel).Only first outcome after COVID-19 captured. Dashed line represents a level of hetereogeneity I2 > 0.4.

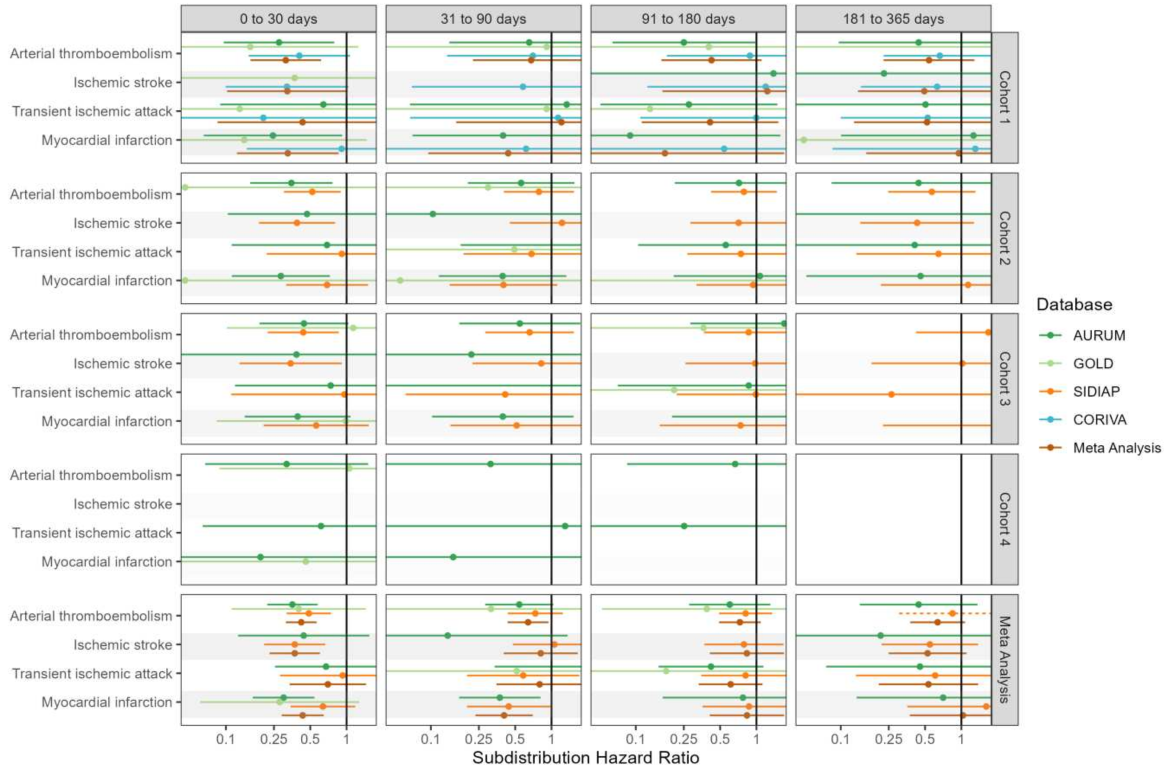

Mercadé-Besora N, *et al. Heart* 2024; 110:635–643. doi: 10.1136/heartjnl-2023-323483

Figure S29: Forest plots for vaccine effect (BNT162b2 vaccine) on preventing arterial thrombosis/thromboembolism complications, for each cohort and database (meta-analysis estimates across cohorts in the last panel). Dashed line represents a level of hetereogeneity I2 > 0.4.

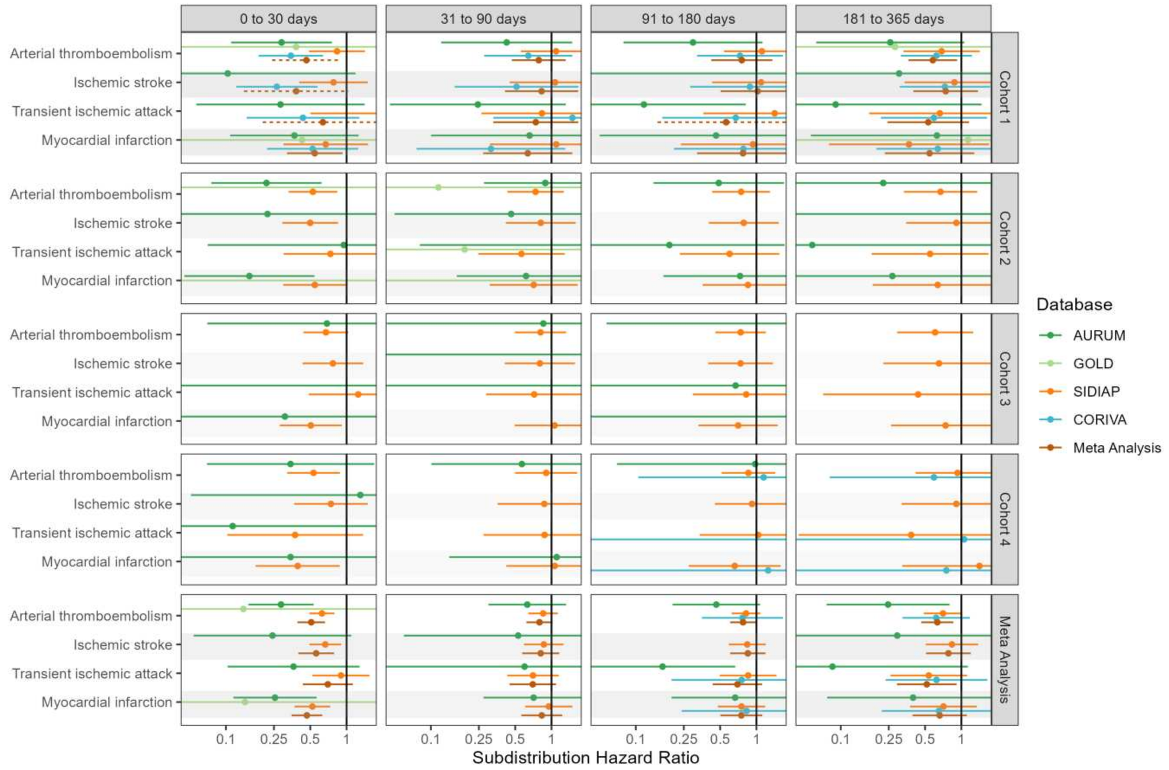

Mercadé-Besora N, *et al. Heart* 2024; 110:635–643. doi: 10.1136/heartjnl-2023-323483

Figure S30: Forest plots for vaccine effect (BNT162b2 vaccine) on preventing arterial thrombosis/thromboembolism complications, for each cohort and database (meta-analysis estimates across cohorts in the last panel).Follow-up ends at first vaccine dose after index date. Dashed line represents a level of hetereogeneity I2 > 0.4.

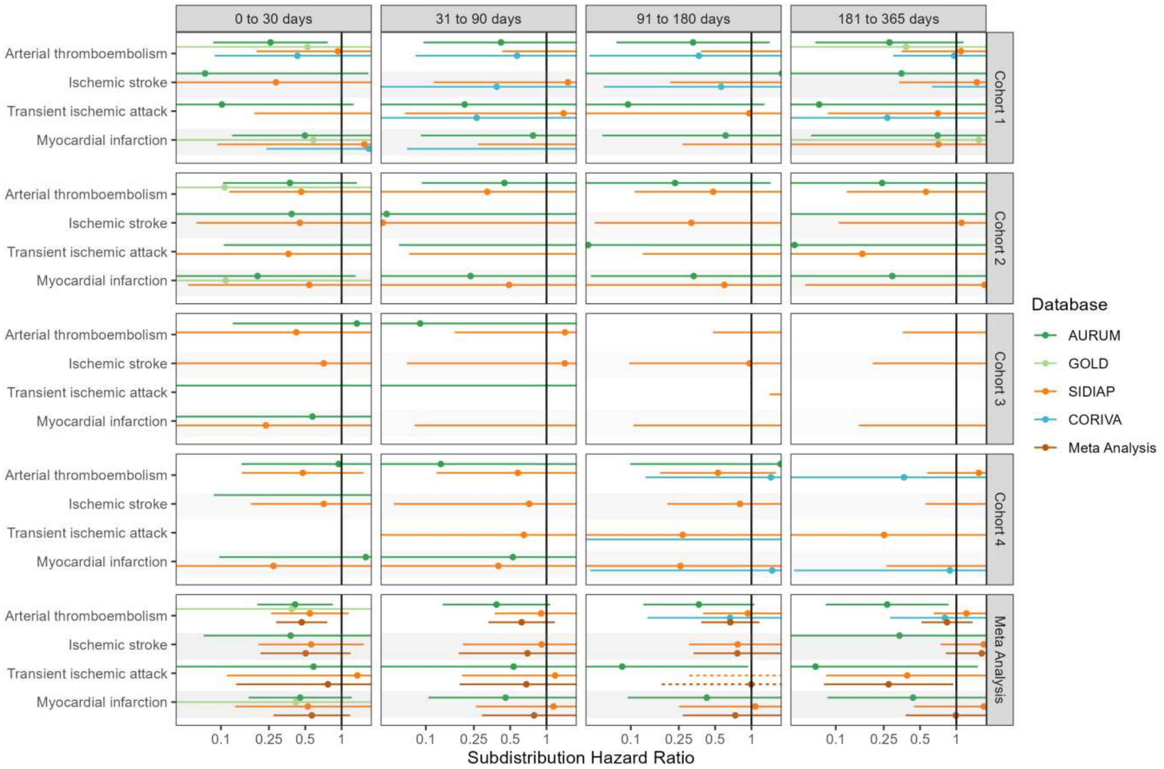

Figure S31: Forest plots for vaccine effect (BNT162b2 vaccine) on preventing arterial thrombosis/thromboembolism complications, for each cohort and database (meta-analysis estimates across cohorts in the last panel).Only first outcome after COVID-19 captured. Dashed line represents a level of hetereogeneity I2 > 0.4.

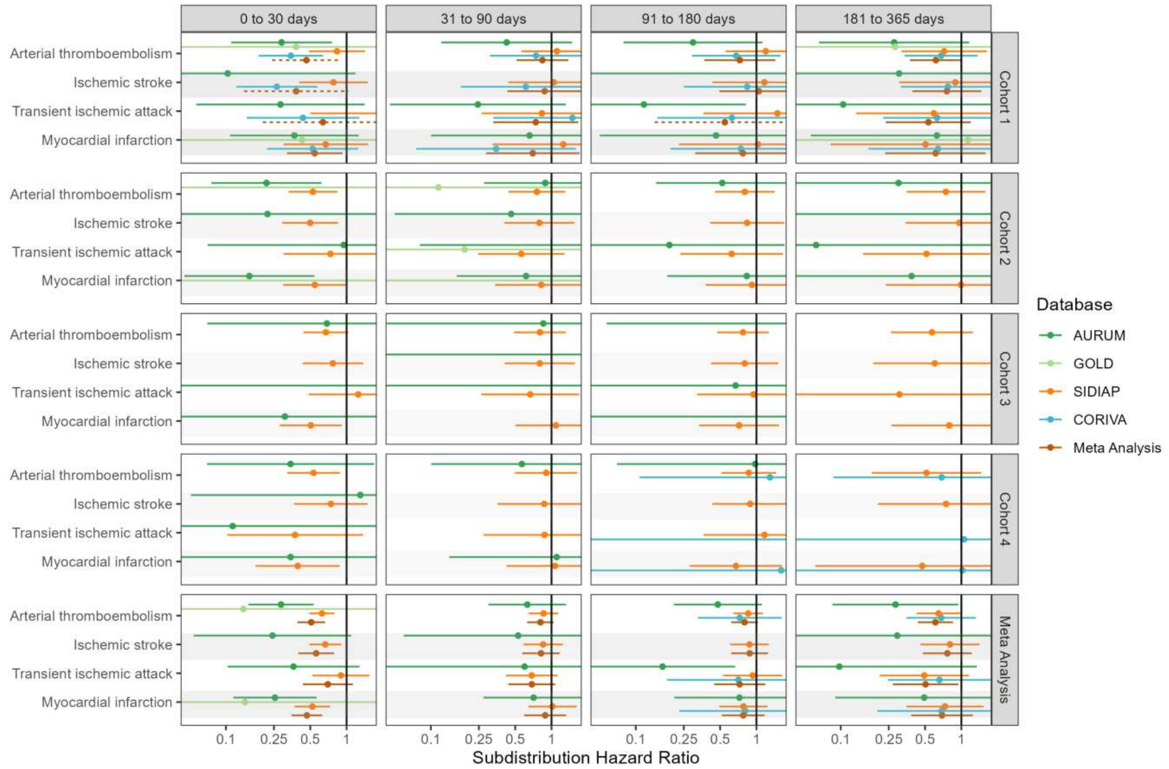

Figure S32: Forest plots for vaccine effect (any COVID-19 vaccine) on preventing cardiac diseases and hemorrhagic stroke, for each cohort and database (meta-analysis estimates across cohorts in the last panel). Dashed line represents a level of hetereogeneity I2 > 0.4.

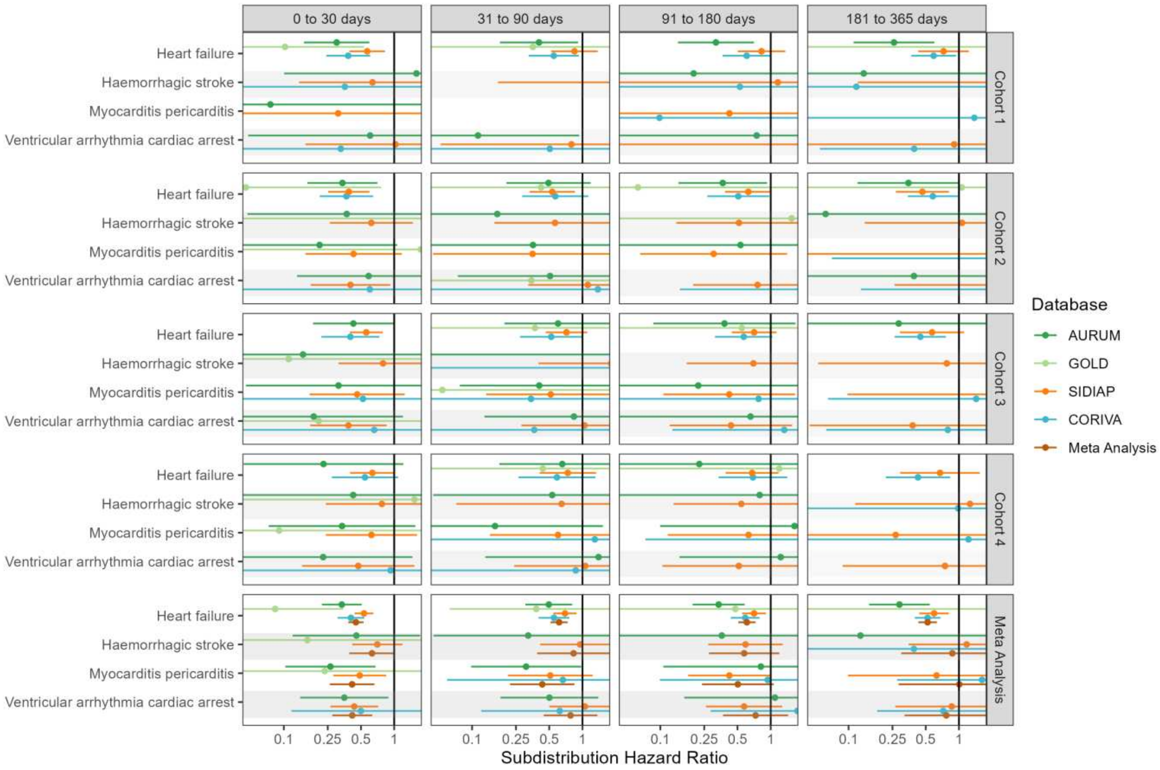

Figure S33: Forest plots for vaccine effect (any COVID-19 vaccine) on preventing cardiac diseases and hemorrhagic stroke, for each cohort and database (meta-analysis estimates across cohorts in the last panel).Follow-up ends at first vaccine dose after index date. Dashed line represents a level of hetereogeneity I2 > 0.4.

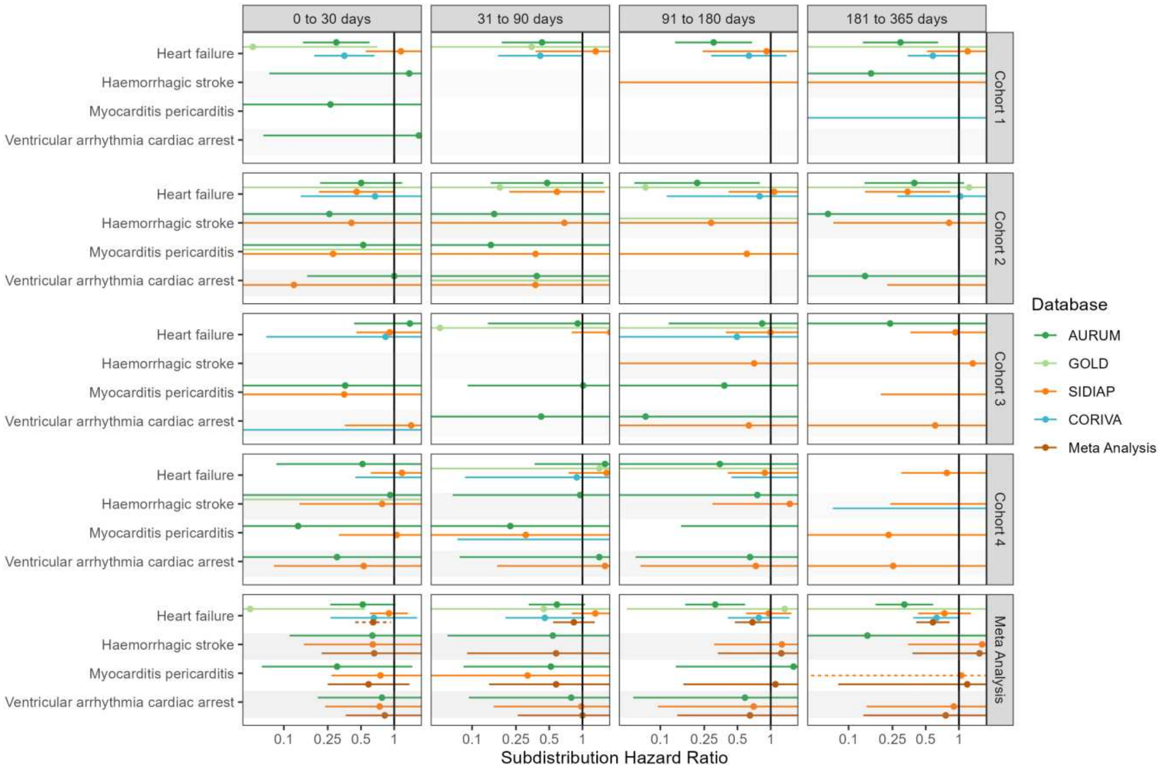

Mercadé-Besora N, *et al. Heart* 2024; 110:635–643. doi: 10.1136/heartjnl-2023-323483

Figure S34: Forest plots for vaccine effect (any COVID-19 vaccine) on preventing cardiac diseases and hemorrhagic stroke, for each cohort and database (meta-analysis estimates across cohorts in the last panel).Only first outcome after COVID-19 captured. Dashed line represents a level of hetereogeneity I2 > 0.4.

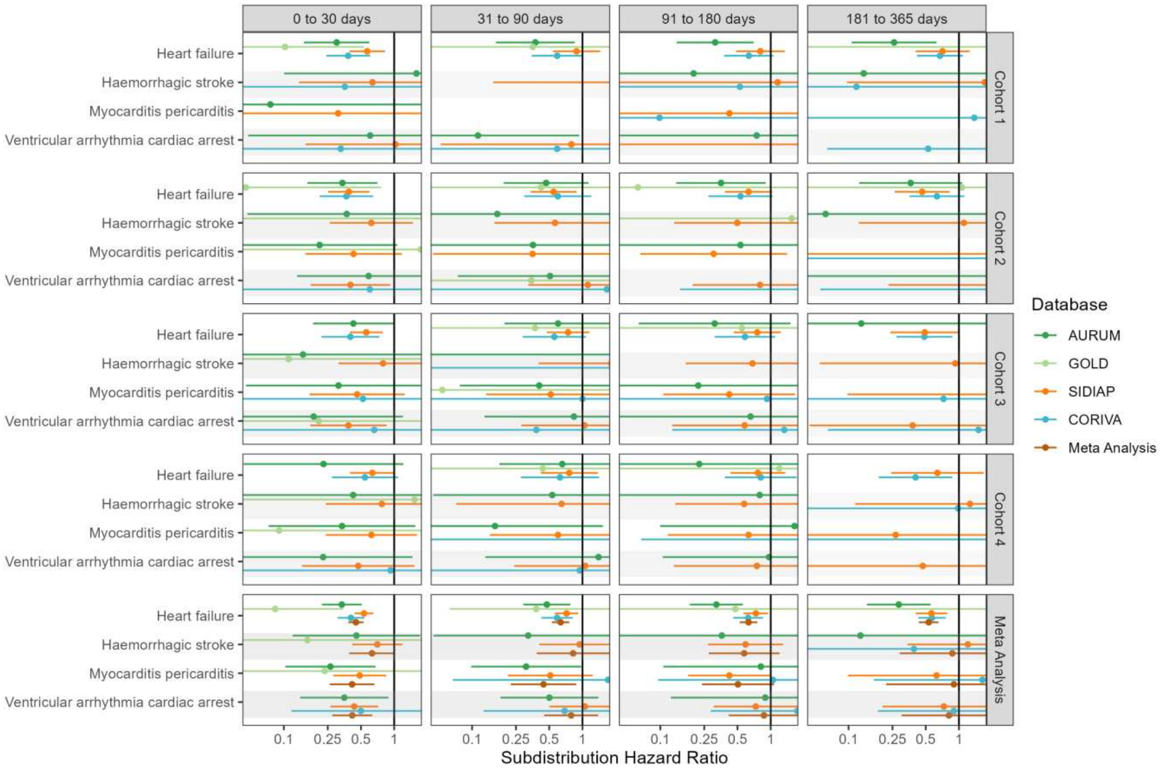

Figure S35: Forest plots for vaccine effect (ChAdOx1 vaccine) on preventing cardiac diseases and hemorrhagic stroke, for each cohort and database (meta-analysis estimates across cohorts in the last panel). Dashed line represents a level of hetereogeneity I2 > 0.4.

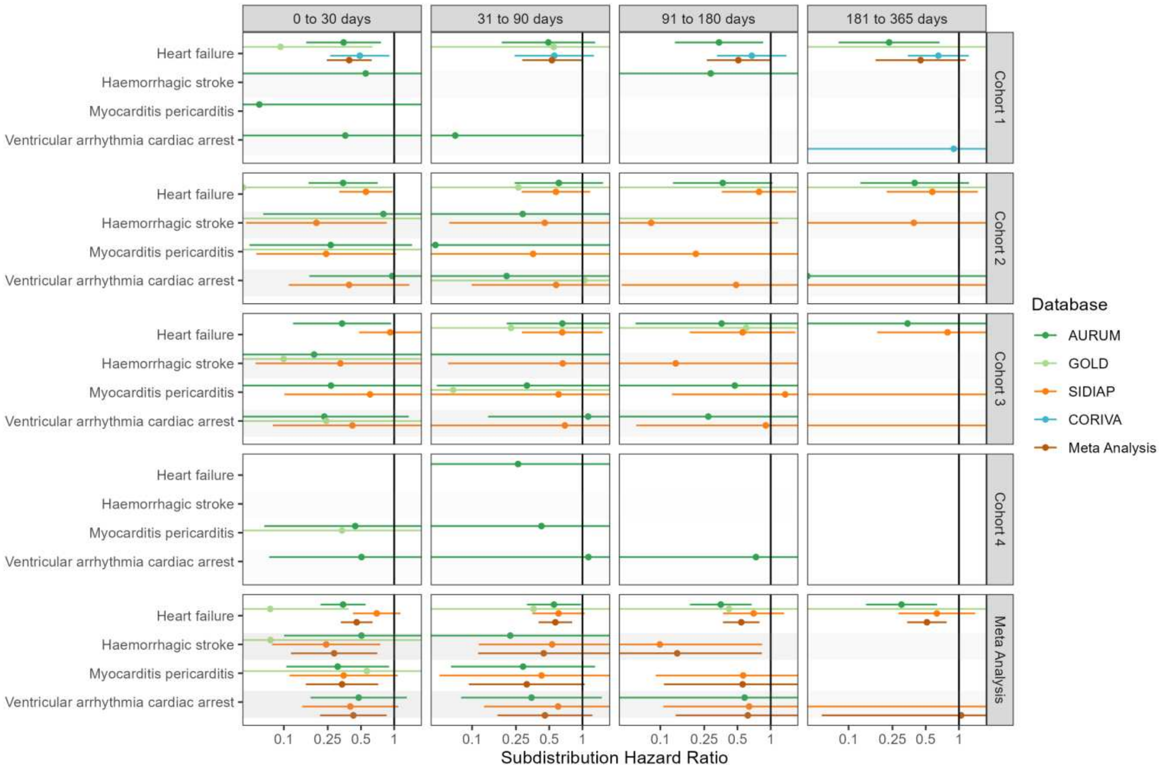

Mercadé-Besora N, *et al. Heart* 2024; 110:635–643. doi: 10.1136/heartjnl-2023-323483

Figure S36: Forest plots for vaccine effect (ChAdOx1 vaccine) on preventing cardiac diseases and hemorrhagic stroke, for each cohort and database (meta-analysis estimates across cohorts in the last panel).Follow-up ends at first vaccine dose after index date. Dashed line represents a level of hetereogeneity I2 > 0.4.

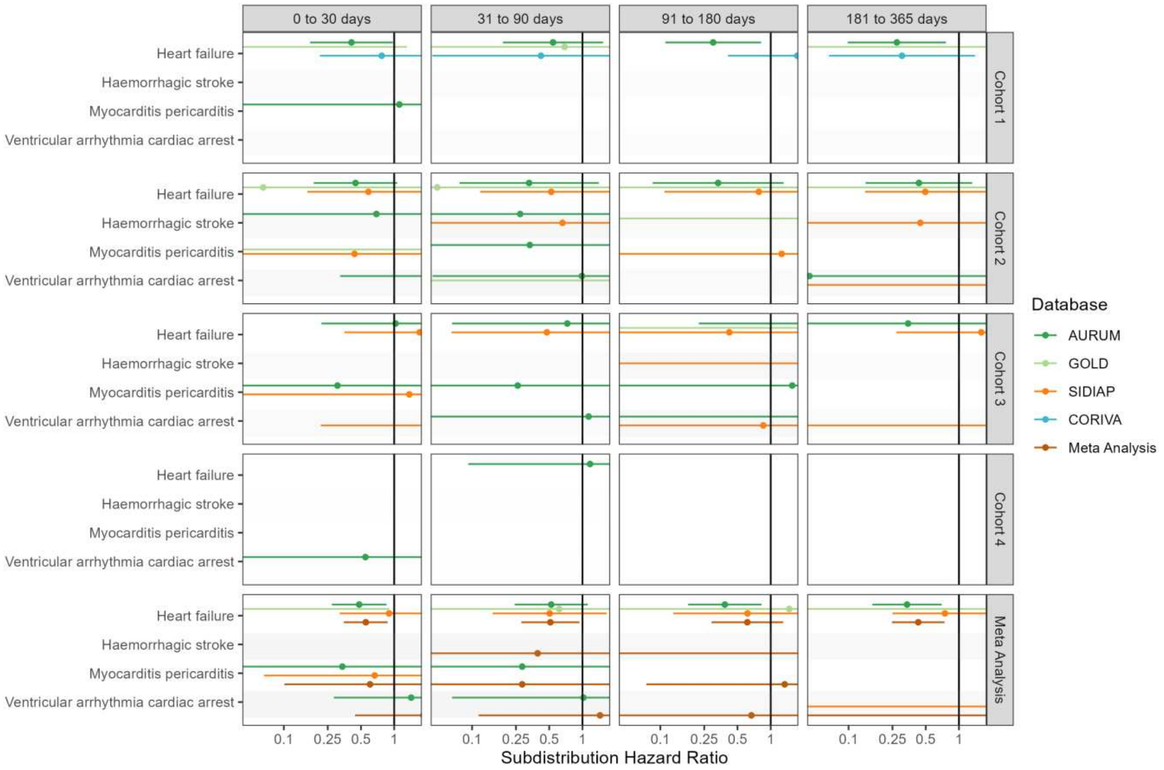

Mercadé-Besora N, *et al. Heart* 2024; 110:635–643. doi: 10.1136/heartjnl-2023-323483

Figure S37: Forest plots for vaccine effect (ChAdOx1 vaccine) on preventing cardiac diseases and hemorrhagic stroke, for each cohort and database (meta-analysis estimates across cohorts in the last panel).Only first outcome after COVID-19 captured. Dashed line represents a level of hetereogeneity I2 > 0.4.

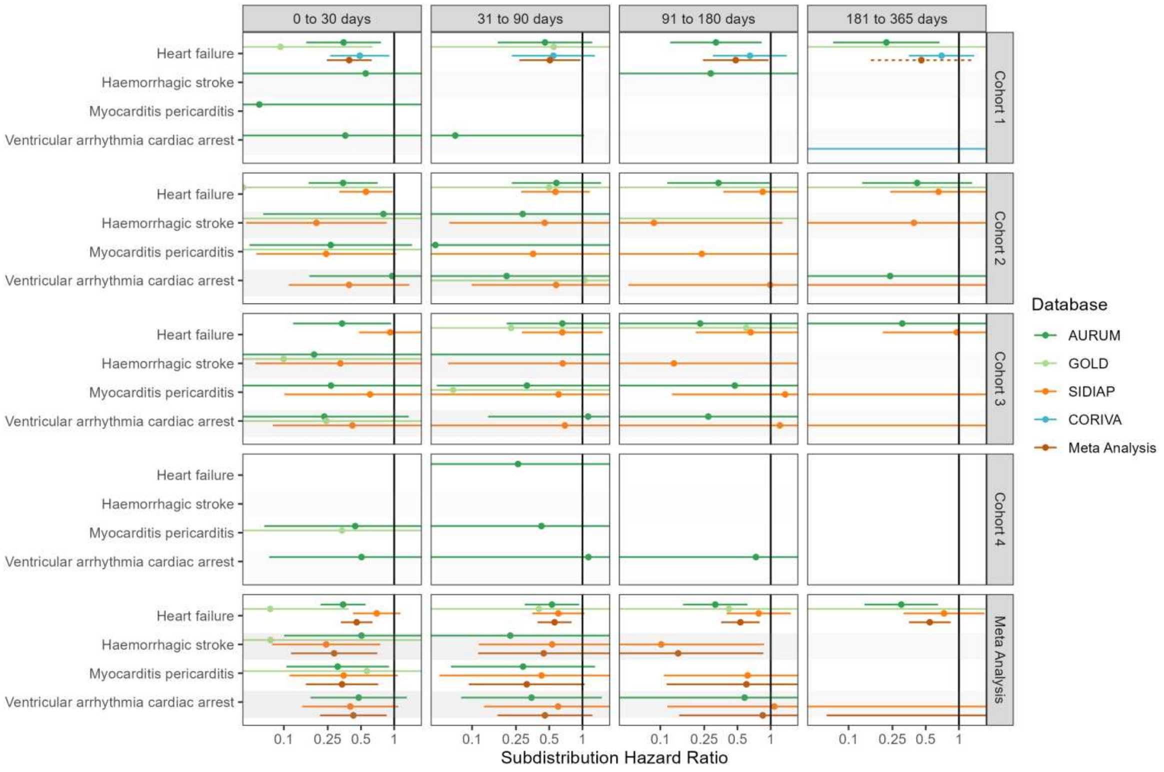

Mercadé-Besora N, *et al. Heart* 2024; 110:635–643. doi: 10.1136/heartjnl-2023-323483

Figure S38: Forest plots for vaccine effect (BNT162b2 vaccine) on preventing cardiac diseases and hemorrhagic stroke, for each cohort and database (meta-analysis estimates across cohorts in the last panel). Dashed line represents a level of hetereogeneity I2 > 0.4.

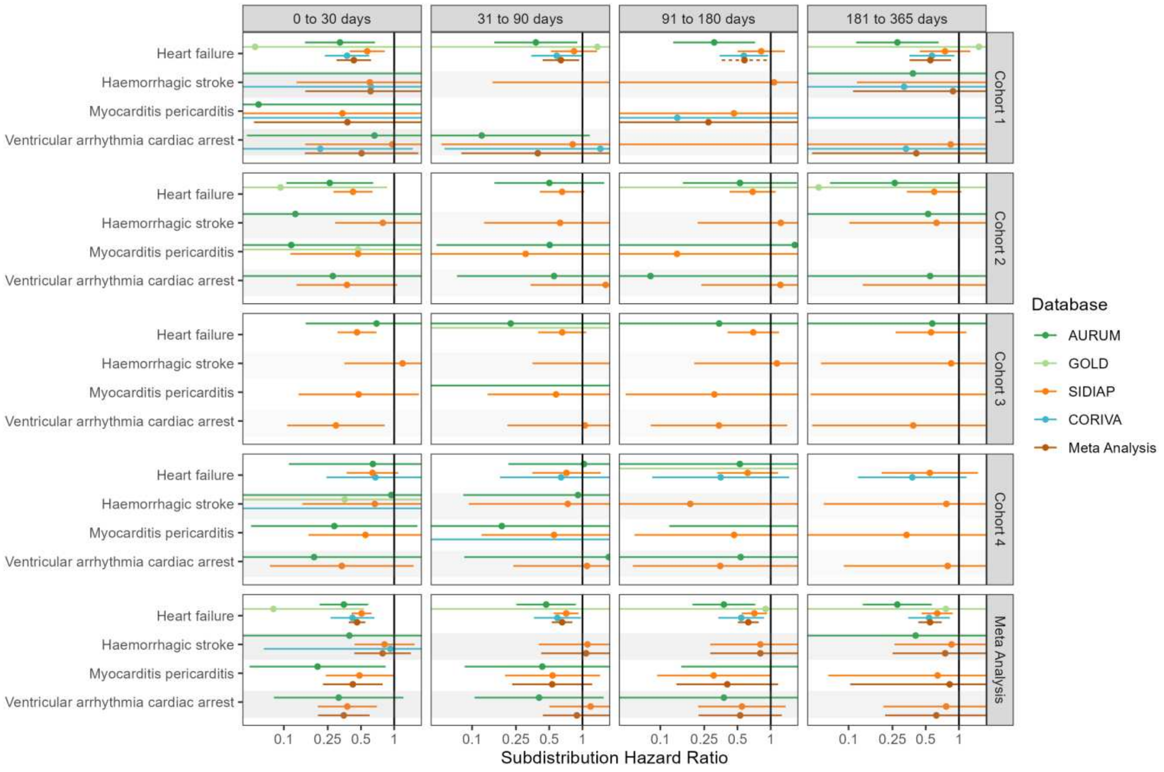

Mercadé-Besora N, *et al. Heart* 2024; 110:635–643. doi: 10.1136/heartjnl-2023-323483

Figure S39: Forest plots for vaccine effect (BNT162b2 vaccine) on preventing cardiac diseases and hemorrhagic stroke, for each cohort and database (meta-analysis estimates across cohorts in the last panel).Follow-up ends at first vaccine dose after index date. Dashed line represents a level of hetereogeneity I2 > 0.4.

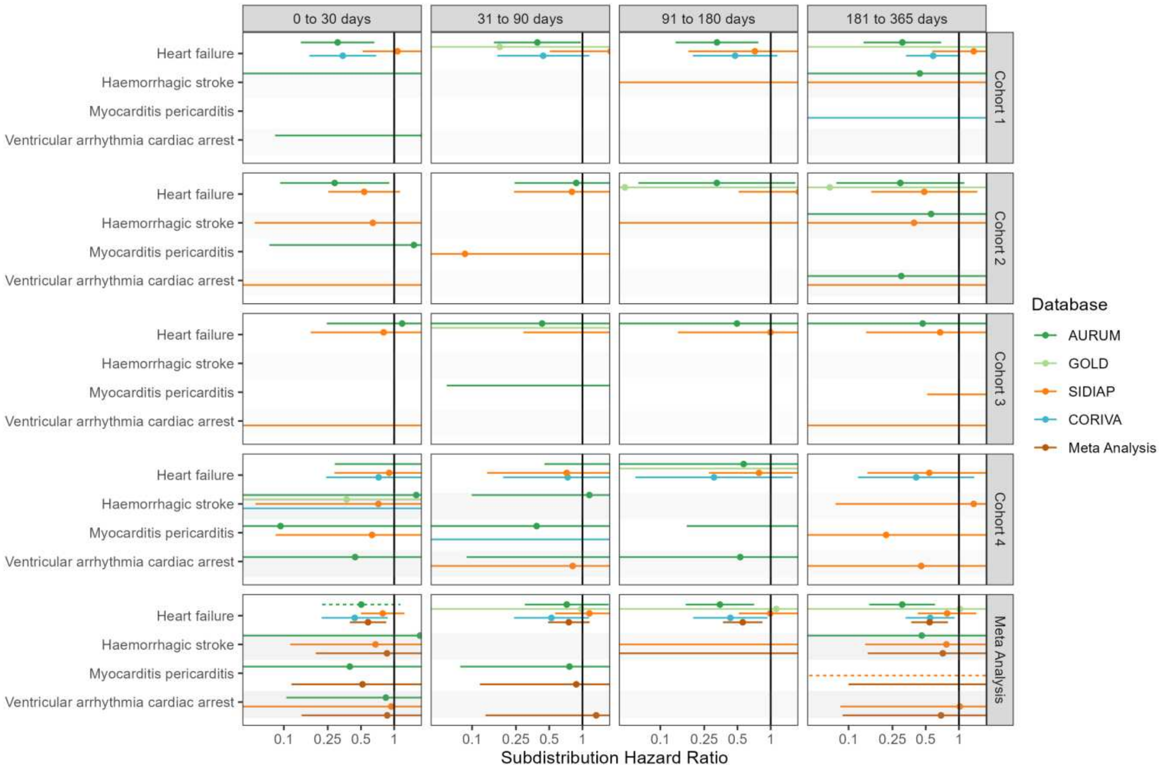

Mercadé-Besora N, *et al. Heart* 2024; 110:635–643. doi: 10.1136/heartjnl-2023-323483

Figure S40: Forest plots for vaccine effect (BNT162b2 vaccine) on preventing cardiac diseases and hemorrhagic stroke, for each cohort and database (meta-analysis estimates across cohorts in the last panel).Only first outcome after COVID-19 captured. Dashed line represents a level of hetereogeneity I2 > 0.4.

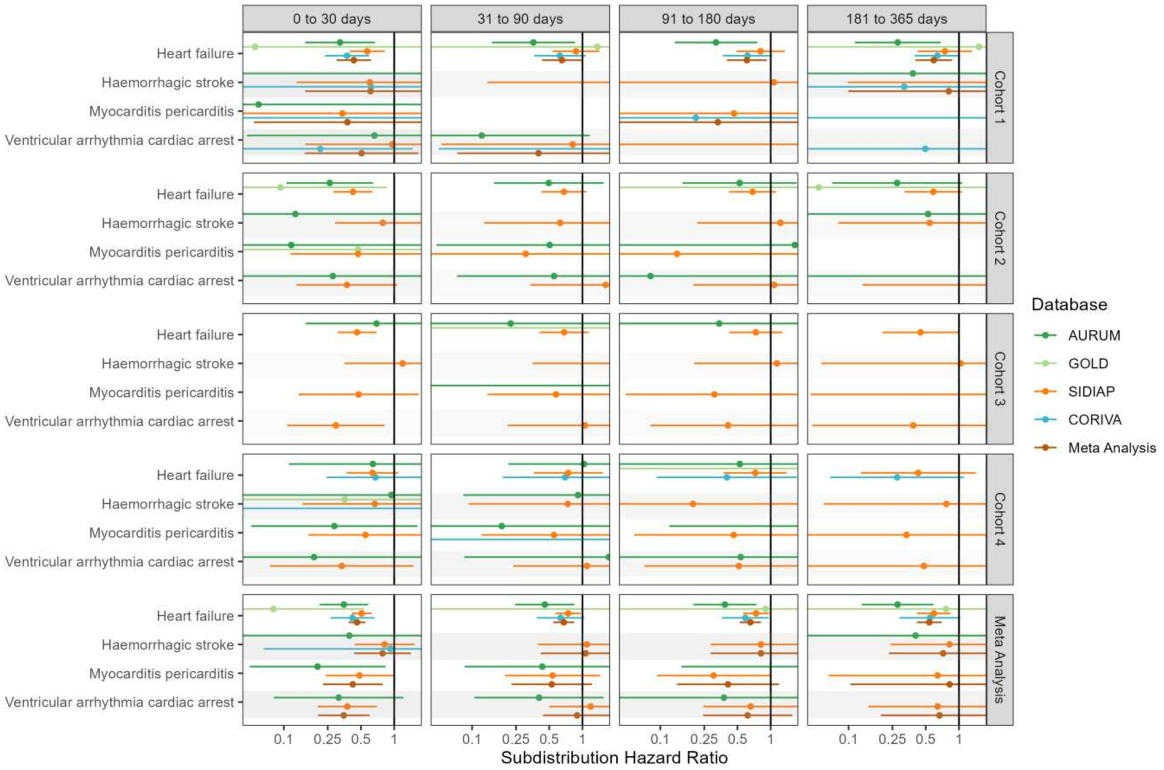

Figure S41: Forest plots for comparative effect, meta-analysis across cohorts and databases. Follow-up ends at first vaccine dose after index date. Dashed line represents a level of hetereogeneity I2 > 0.4.

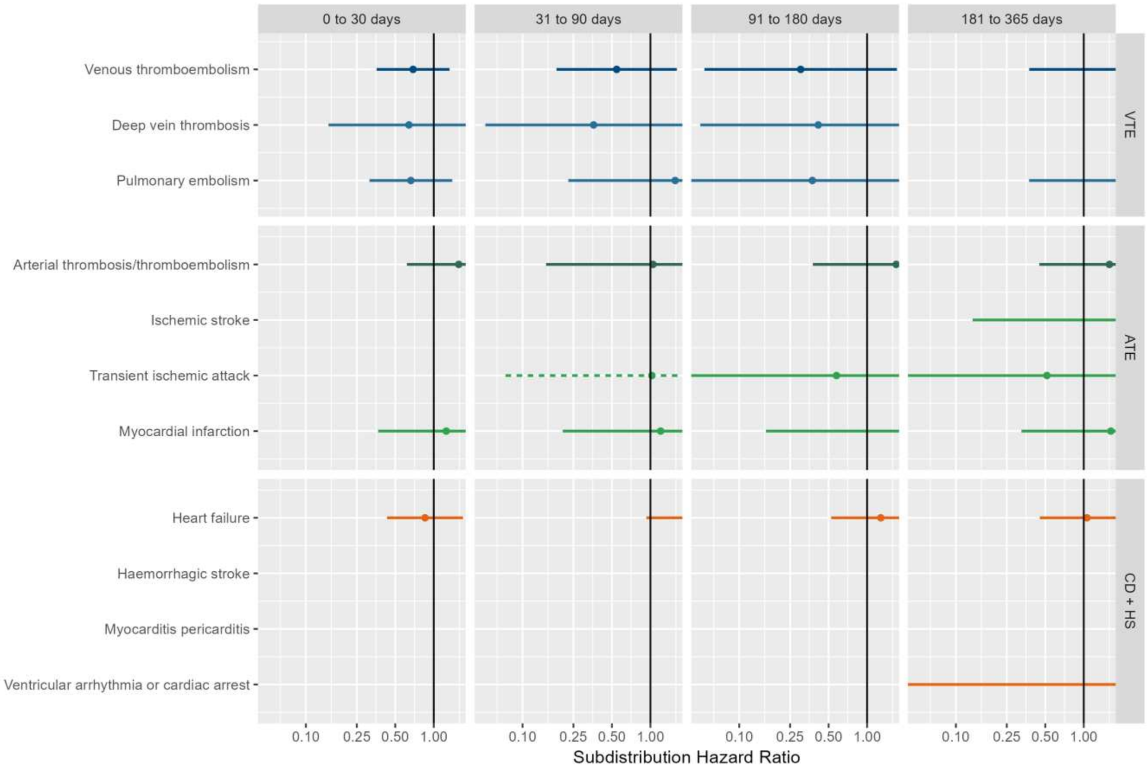

VTE = Venous thromboembolism, ATE = Arterial thrombosis/thromboembolism, CD + HS = Cardiac diseases and Hemorrhagic stroke

Figure S42: Forest plots for comparative effect, meta-analysis across cohorts and databases. Only first outcome after COVID-19 captured. Dashed line represents a level of hetereogeneity I2 > 0.4.

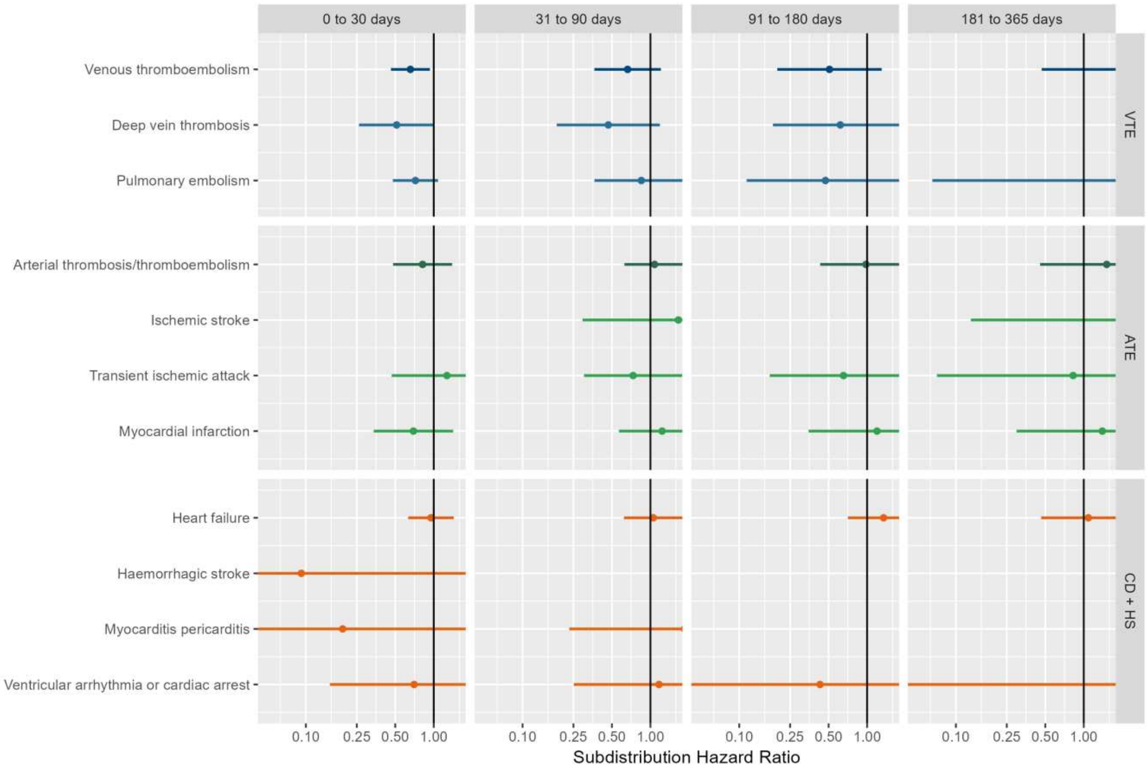

VTE = Venous thromboembolism, ATE = Arterial thrombosis/thromboembolism, CD + HS = Cardiac diseases and Hemorrhagic stroke

Figure S43: Forest plots for comparative effect on preventing venous thromboembolism complications, for each cohort and database (meta-analysis estimates across cohorts in the last panel). Dashed line represents a level of hetereogeneity I2 > 0.4.

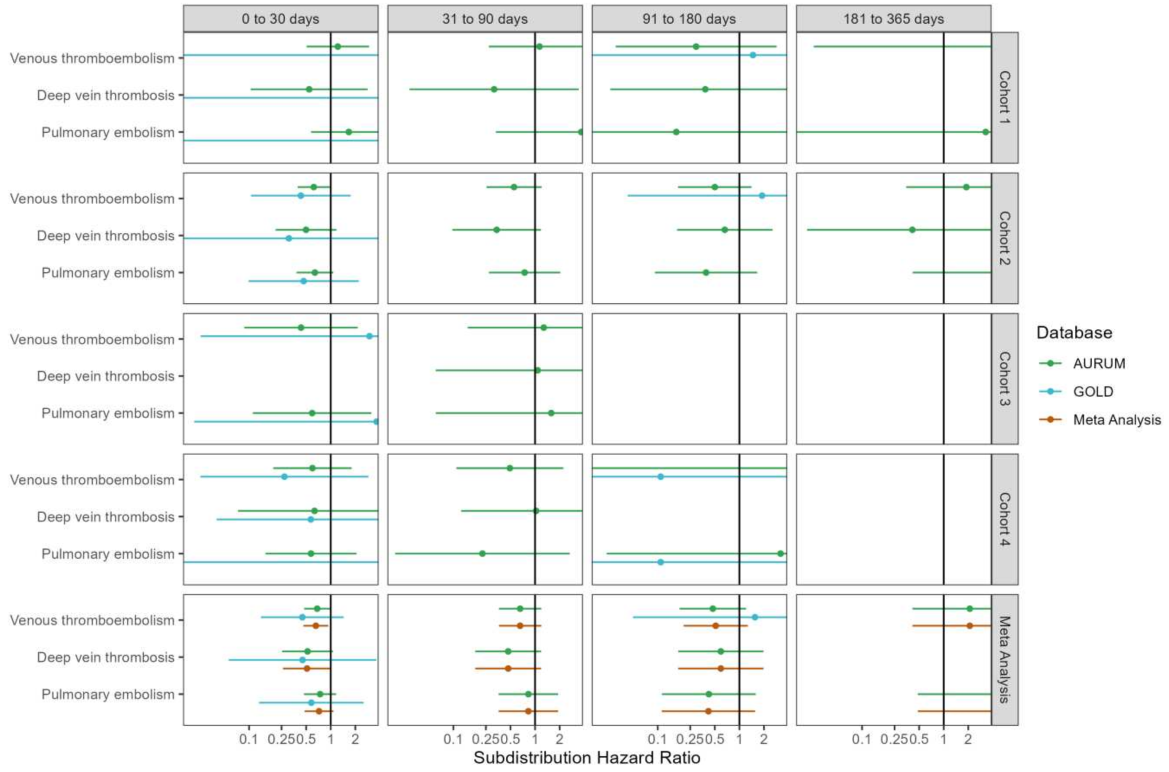

Mercadé-Besora N, *et al. Heart* 2024; 110:635–643. doi: 10.1136/heartjnl-2023-323483

Figure S44: Forest plots for comparative effect on preventing venous thromboembolism complications, for each cohort and database (meta-analysis estimates across cohorts in the last panel).Follow-up ends at first vaccine dose after index date. Dashed line represents a level of hetereogeneity I2 > 0.4.

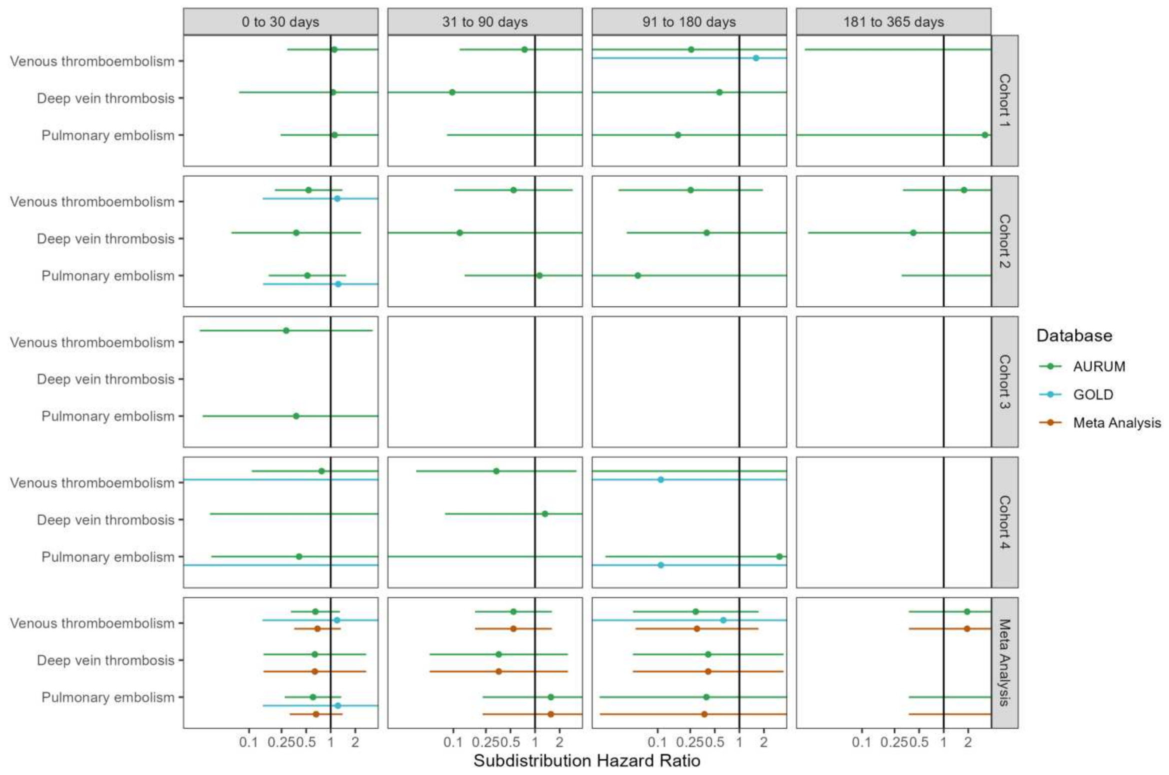

Figure S45: Forest plots for comparative effect on preventing venous thromboembolism complications, for each cohort and database (meta-analysis estimates across cohorts in the last panel).Only first outcome after COVID-19 captured. Dashed line represents a level of hetereogeneity I2 > 0.4.

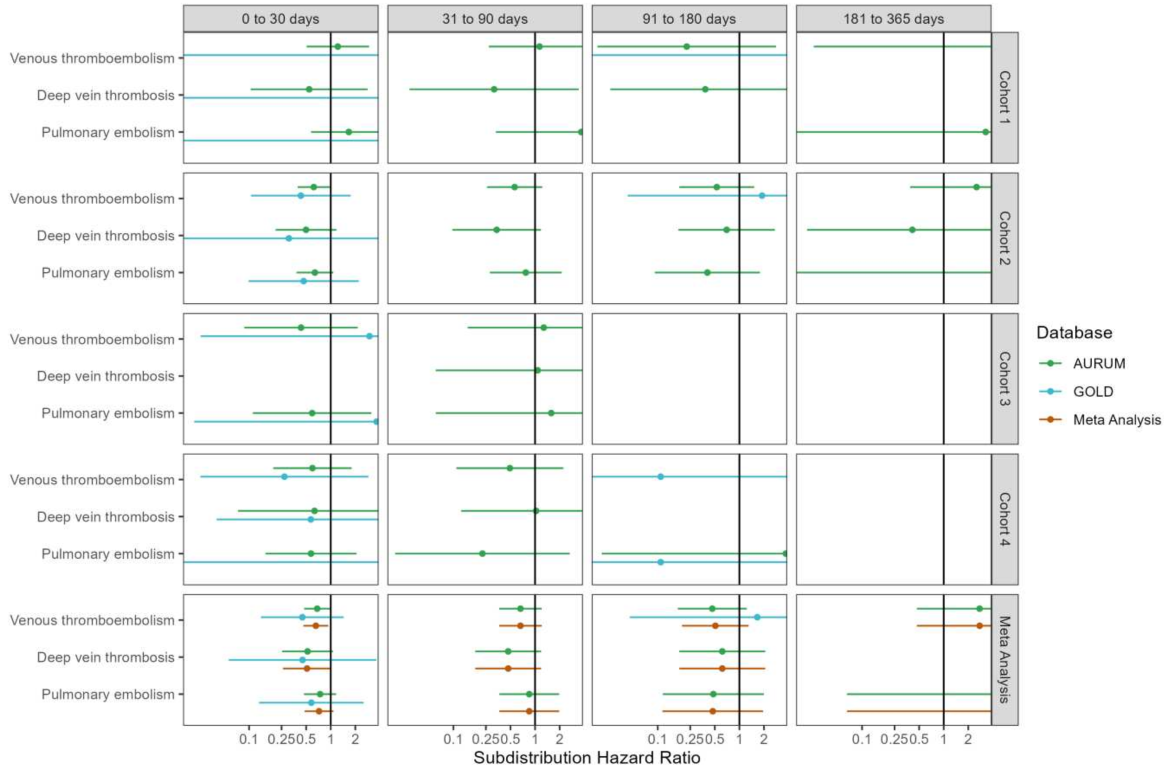

155

Figure S46: Forest plots for comparative effect on preventing arterial thrombosis/thromboembolism complications, for each cohort and database (meta-analysis estimates across cohorts in the last panel). Dashed line represents a level of hetereogeneity I2 > 0.4.

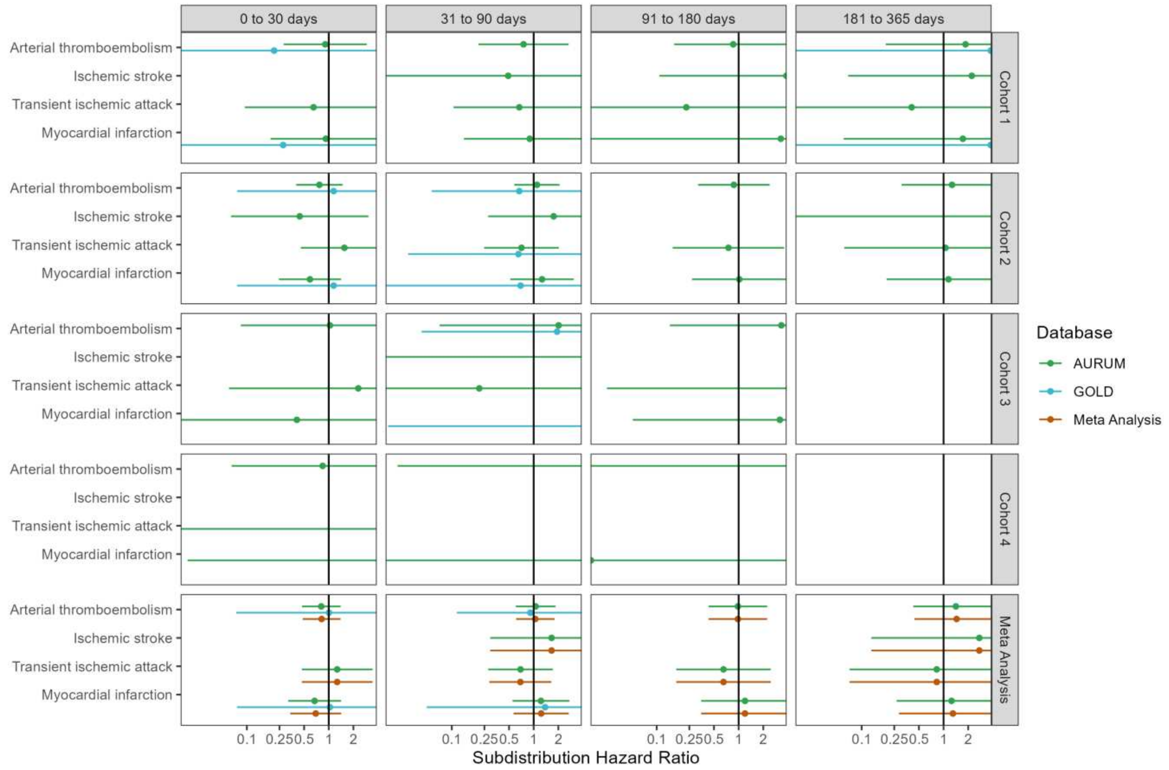

Mercadé-Besora N, *et al. Heart* 2024; 110:635–643. doi: 10.1136/heartjnl-2023-323483

Figure S47: Forest plots for comparative effect on preventing arterial thrombosis/thromboembolism complications, for each cohort and database (meta-analysis estimates across cohorts in the last panel).Follow-up ends at first vaccine dose after index date. Dashed line represents a level of hetereogeneity I2 > 0.4.

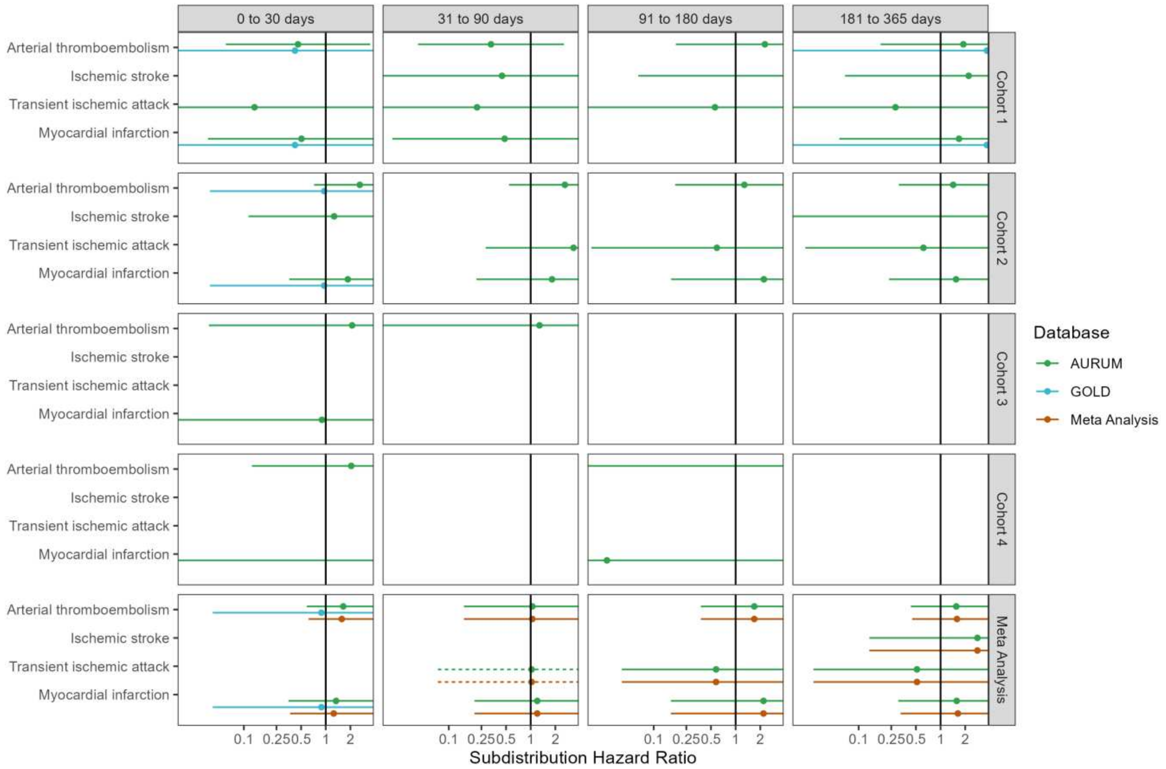

Mercadé-Besora N, *et al. Heart* 2024; 110:635–643. doi: 10.1136/heartjnl-2023-323483

Figure S48: Forest plots for comparative effect on preventing arterial thrombosis/thromboembolism complications, for each cohort and database (meta-analysis estimates across cohorts in the last panel).Only first outcome after COVID-19 captured. Dashed line represents a level of hetereogeneity I2 > 0.4.

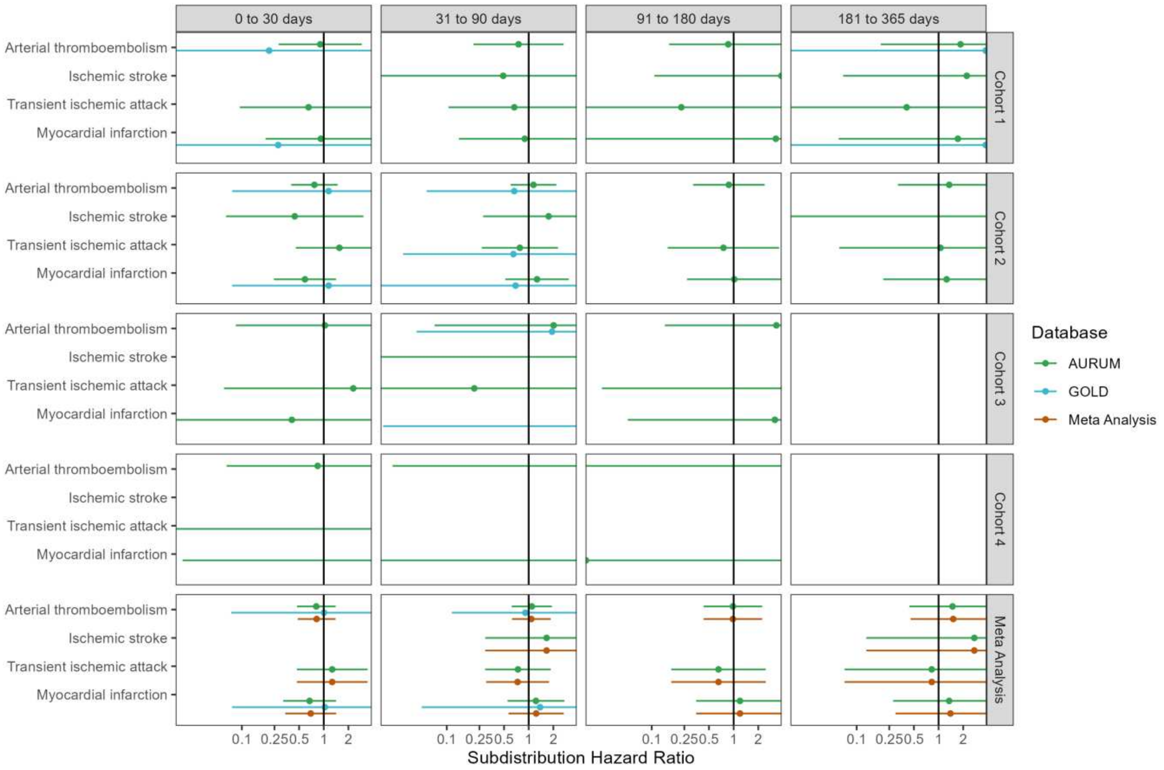

Figure S49: Forest plots for comparative effect on preventing cardiac diseases and hemorrhagic stroke, for each cohort and database (meta-analysis estimates across cohorts in the last panel). Dashed line represents a level of hetereogeneity I2 > 0.4.

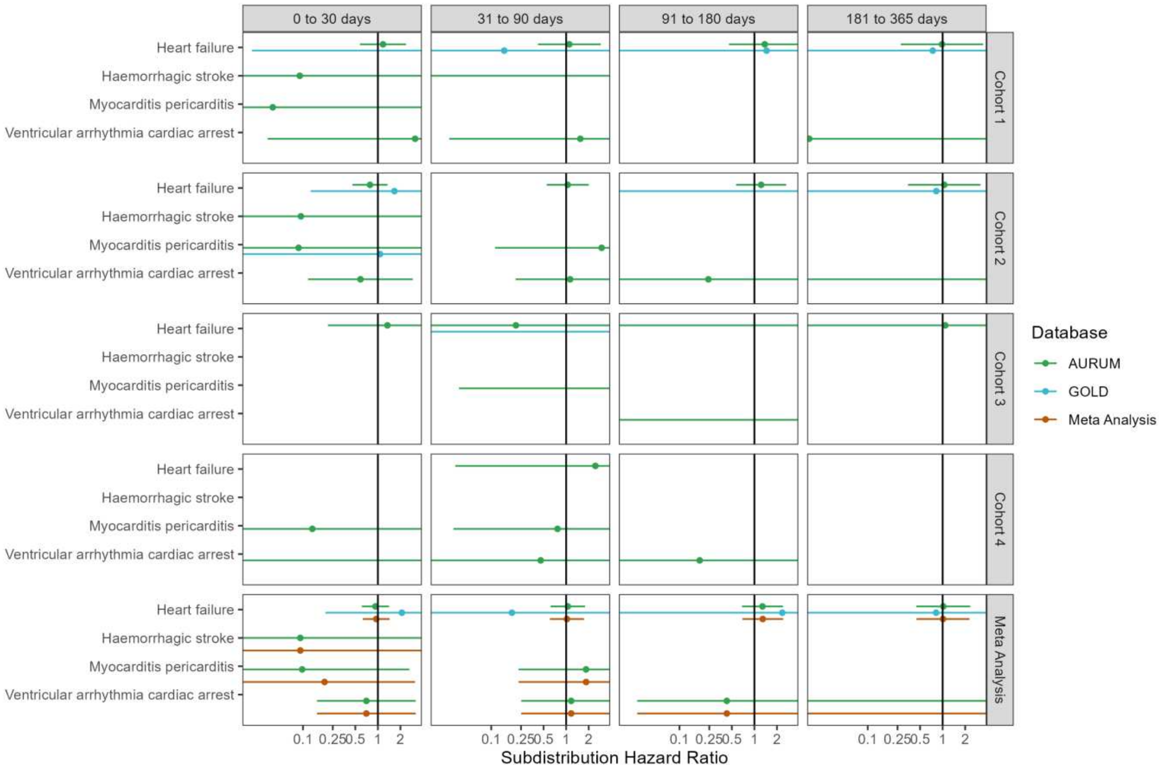

Mercadé-Besora N, *et al. Heart* 2024; 110:635–643. doi: 10.1136/heartjnl-2023-323483

Figure S50: Forest plots for comparative effect on preventing cardiac diseases and hemorrhagic stroke, for each cohort and database (meta-analysis estimates across cohorts in the last panel).Follow-up ends at first vaccine dose after index date. Dashed line represents a level of hetereogeneity I2 > 0.4.

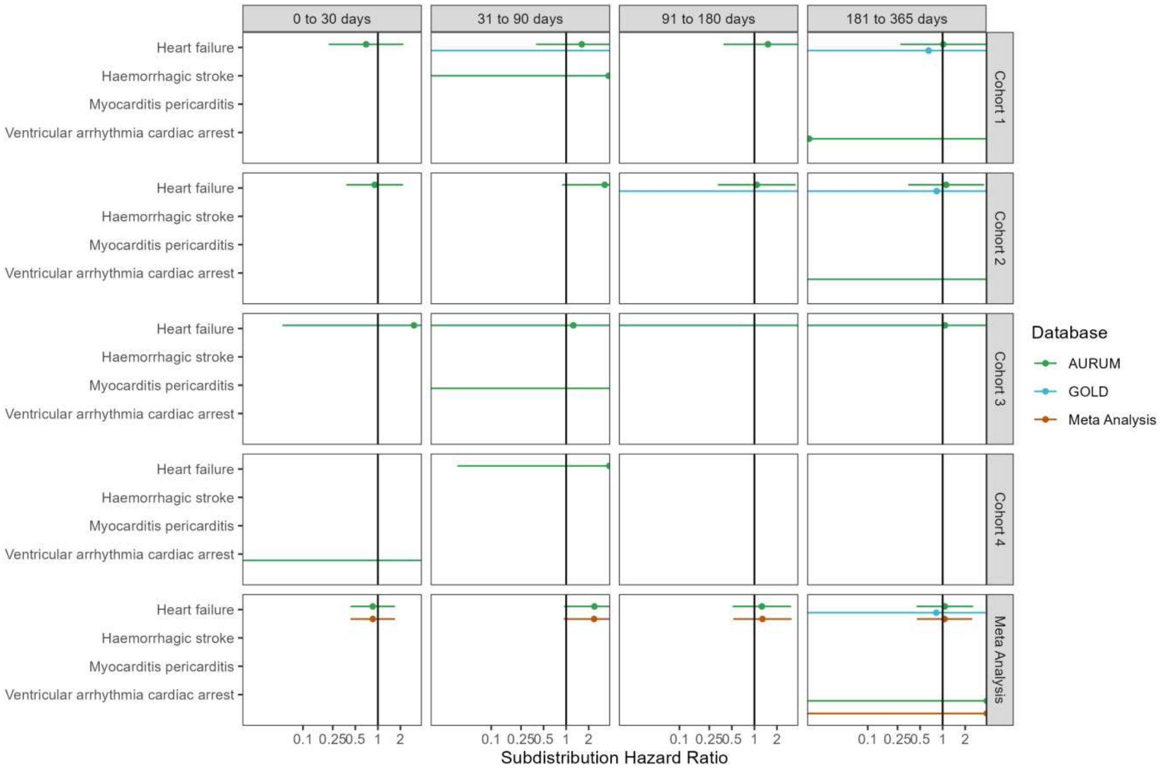

Figure S51: Forest plots for comparative effect on preventing cardiac diseases and hemorrhagic stroke, for each cohort and database (meta-analysis estimates across cohorts in the last panel).Only first outcome after COVID-19 captured. Dashed line represents a level of hetereogeneity I2 > 0.4.

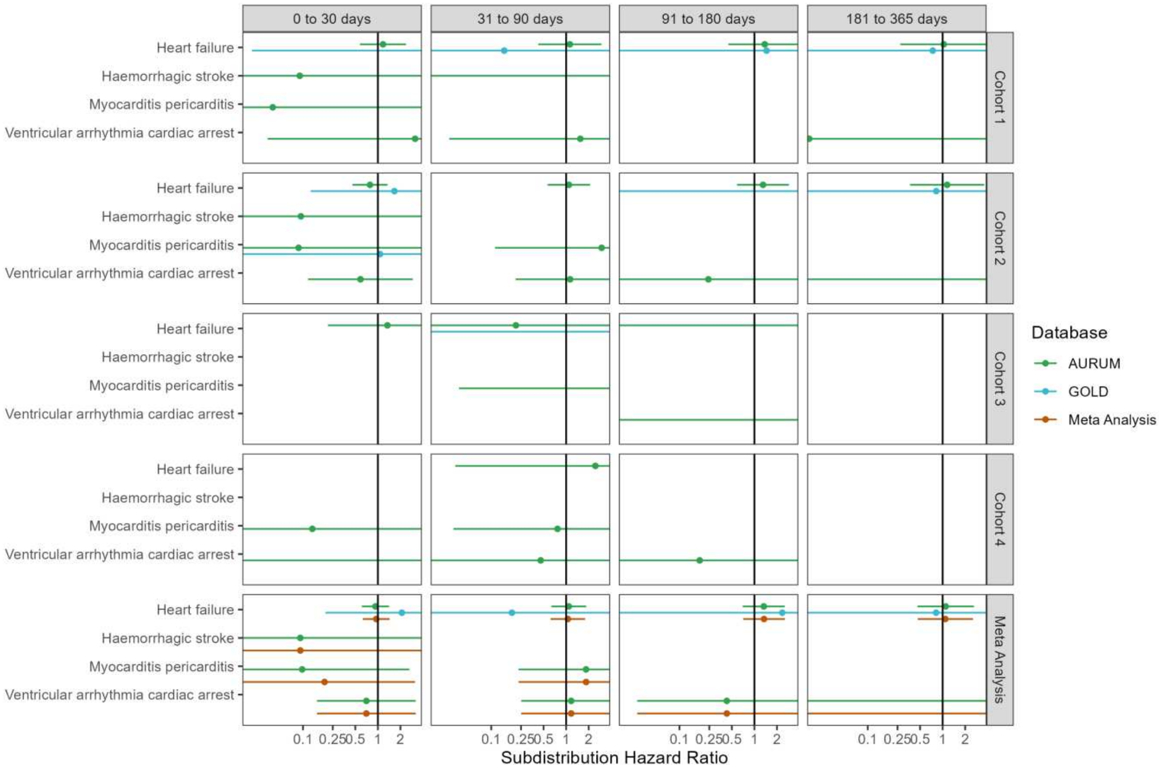

Mercadé-Besora N, *et al. Heart* 2024; 110:635–643. doi: 10.1136/heartjnl-2023-323483
